# The LUX Score: A Metric for Lipidome Homology

**DOI:** 10.1371/journal.pcbi.1004511

**Published:** 2015-09-22

**Authors:** Chakravarthy Marella, Andrew E. Torda, Dominik Schwudke

**Affiliations:** 1 Division of Bioanalytical Chemistry, Research Center Borstel, Leibniz-Center for Medicine and Biosciences, Borstel, SH, Germany; 2 National Centre for Biological Sciences, Tata Institute of Fundamental Research, Bangalore, KA, India; 3 Centre for Bioinformatics, University of Hamburg, Hamburg, Germany; 4 Airway Research Center North, German Center for Lung Research, Grosshansdorf, SH, Germany; 5 German Center for Infection Research, TTU-Tb, Borstel, SH, Germany; University of Wisconsin-Madison, UNITED STATES

## Abstract

A lipidome is the set of lipids in a given organism, cell or cell compartment and this set reflects the organism’s synthetic pathways and interactions with its environment. Recently, lipidomes of biological model organisms and cell lines were published and the number of functional studies of lipids is increasing. In this study we propose a homology metric that can quantify systematic differences in the composition of a lipidome. Algorithms were developed to 1. consistently convert lipids structure into SMILES, 2. determine structural similarity between molecular species and 3. describe a lipidome in a chemical space model. We tested lipid structure conversion and structure similarity metrics, in detail, using sets of isomeric ceramide molecules and chemically related phosphatidylinositols. Template-based SMILES showed the best properties for representing lipid-specific structural diversity. We also show that sequence analysis algorithms are best suited to calculate distances between such template-based SMILES and we adjudged the Levenshtein distance as best choice for quantifying structural changes. When all lipid molecules of the LIPIDMAPS structure database were mapped in chemical space, they automatically formed clusters corresponding to conventional chemical families. Accordingly, we mapped a pair of lipidomes into the same chemical space and determined the degree of overlap by calculating the Hausdorff distance. We named this metric the ‘Lipidome jUXtaposition (LUX) score’. First, we tested this approach for estimating the lipidome similarity on four yeast strains with known genetic alteration in fatty acid synthesis. We show that the LUX score reflects the genetic relationship and growth temperature better than conventional methods although the score is based solely on lipid structures. Next, we applied this metric to high-throughput data of larval tissue lipidomes of Drosophila. This showed that the LUX score is sufficient to cluster tissues and determine the impact of nutritional changes in an unbiased manner, despite the limited information on the underlying structural diversity of each lipidome. This study is the first effort to define a lipidome homology metric based on structures that will enrich functional association of lipids in a similar manner to measures used in genetics. Finally, we discuss the significance of the LUX score to perform comparative lipidome studies across species borders.

## Author Summary

Because of their role in health and disease, lipids are often the focus of biochemical studies. Advances in analytical biochemistry have made it possible to detect all the lipids from a cell, tissue or organism (termed lipidome). Much of this research is based on model organisms, but it is difficult to transfer results from a fruit fly or yeast to human biochemistry. A central problem is that there is no agreed-upon method for comparing lipidomes. We have developed the LUX score, which enables us to determine the homology between lipidomes. All constituent lipids are first embedded in a chemical space according to their similarity to each other. When we treat all lipids as points in such a space, one can overlay different lipidomes and estimate their differences. We expect that this kind of metric will be useful for translating findings from model organisms to human diseases and in understanding fundamental biological processes.

## Introduction

A lipidome can be an indicator of health, disease, stress or metabolic state. Using model organisms, the role of lipid metabolism has been studied in diseases such as diabetes, metabolic syndrome, neurodegeneration and cancer [1–5]. To this end, lipidomes from yeast and fruit fly have been characterised [6–10] enabling one to identify fundamental lipid metabolic processes [11,12]. However, a critical question remains: How relevant are lipidome changes in model organisms to human physiology, if these lipids are not present in humans?

For example, it would be a challenge to relate differences in lipid metabolism in *D*. *melanogaster* or *S*. *cerevisiae* to human biochemistry (S1 Table). One only has to consider their differing sphingolipid compositions [13]. In humans, sphingomyelins (SM) are highly abundant, but they are basically absent in the fruit fly. Furthermore, drosophila sphingolipids have shorter sphingoid alkyl chains, but their amide linked fatty acids are usually longer than those in humans.

The theme in this work is the development of metrics for lipidome similarity, largely based on established methods used on protein or gene sequences. We started by converting lipid structures to Simplified Molecular Input Line Entry Specification (SMILES) [14]. This representation is compact and allows one to use methods developed for fast string comparisons. One can also take advantage of the literature on SMILES-based methods in cheminformatics [15–17]. Given this structure representation, we used alignment and scoring methods such as Smith and Waterman [18] and the Levenshtein distance [19,20] and looked at the distances between lipids. Building on these distances, one can represent a whole lipidome as a dissimilarity matrix. This numerical representation can then be used for further analyses such as estimating the homology between lipidomes.

Analogous to chemical space models in the field of drug-discovery, the lipid similarity measures were used to define a high dimensional space [21]. This approach was evaluated on all lipids of the LIPIDMAPS Structure Database (LMSD) [22]. We then applied the chemical space model and determined the Hausdorff distance for four well characterized yeast strains [6] that enabled us to lay the basis for the ‘Lipidome jUXtaposition (LUX) score’. Finally, we characterized the LUX score properties on high-throughput lipidomics data of Drosophila larval tissue [8].

## Results

### Alignment based similarity scoring methods distinguish positional isomers

**Fig 1:**
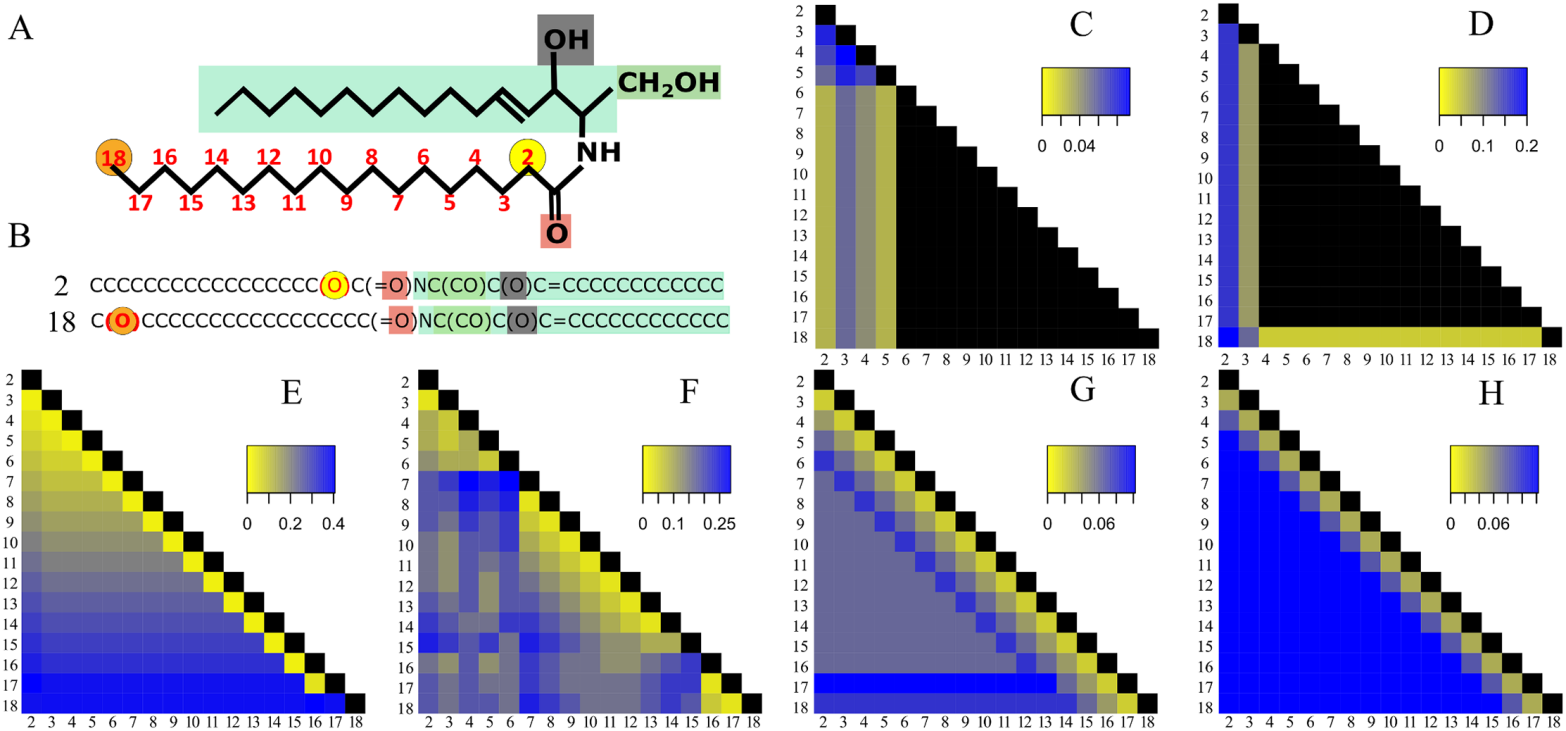
Alignment-based distance calculation algorithms can distinguish isomeric lipid molecules. (A) Structure of 17 ceramide molecules consisting of a *C-16* sphingoid base (light green) and an amide-linked hydroxy fatty acid. The carbon atom number of the hydroxyl group position at the fatty acid chain (red) is used for naming individual molecules. (B) SMILES representation of first and last molecules. Colour coding of atoms is identical in SMILES- and structure- representations. (C-H) Heat map of pairwise distances calculated using Open Babel’s FP2 Fingerprint (C) LINGO (D) Bioisosteric (E) SMILIGN (F) Smith-Waterman (G) and Levenshtein distance (H) algorithms. Bioisosteric method uses CACTVS canonical SMILES, whereas for all other methods template-based SMILES were used. Colour bars in each panel indicate range of distances values of the particular method. Black pixels denote a distance of zero, indicating identical molecules. Numbers in rows and columns represent 1) the molecule name and 2) the position of hydroxyl group in fatty acid moiety.

The first step was to establish a template-based method to generate SMILES strings for lipids. We were able to write SMILES in a consistent and predictable manner using template-based structure drawing tools [23,24] and the Open Babel default SMILES algorithm (S1 Protocol). Given these strings, we then tested alignment methods and distance metrics, analogous to those used for protein or nucleotide sequences. Our quality criterion was based on the methods' sensitivity to detect small structural differences commonly found in lipids. The first test dataset consisted of a set of 17 ceramide molecules with the chemical composition C_34_H_68_O_4_N_1_. The position of the hydroxyl group was successively changed from position 2 to 18 at the fatty acid moiety, resulting in 17 isomeric molecules (Fig 1A and 1B). The shift of the hydroxyl group can be easily recognized in the SMILES strings. We then tested six similarity scoring methods (Fig 1C–1H, S1 Dataset). Three from the literature were used as described under Methods: FP2 Fingerprint [16] LINGO [15] and Bioisosteric similarity [17]. Three methods were introduced here: the SMILIGN, Smith and Waterman [18] and Levenshtein distance [19,20].

The first clear result is that a large subset of isomeric structures cannot be distinguished by either Open Babel FP2 Fingerprint or LINGO (Fig 1C and 1D). The FP2 Fingerprint algorithm computed a distance of zero for 78 pairs of ceramide isomers (Fig 1C–black pixels). LINGO gave a zero distance for 91 pairs of isomers (Fig 1D). This would only be correct if the molecules were identical. Both methods segment SMILES into shorter sub-strings (1–7 characters in the Path-length Fingerprint and 4 characters by LINGO) and apply the Tanimoto coefficient for determining distances. This segmentation loses the information on the position of the hydroxyl group. In contrast, the Bioisosteric algorithm distinguished all 17 isomeric structures, even though it uses CACTVS Canonical SMILES. There are no zero distances off the diagonal (Fig 1E). The Bioisosteric method also segments SMILES into sub-strings, but in a hierarchical manner, preserving information on the position of the hydroxyl group [17]. There is a distinct pattern in the heat map of the Bioisosteric method characterized by a gradual increase in distance values for isomers having the hydroxyl group closer to the terminal methyl carbon. The Bioisosteric method returns a distance of 0.13 units for the shift of the hydroxyl group from position 5 to 7 (Fig 1E–yellow pixel), but returns 0.26 units for position 10 to 12 and for positions 16 to 18, a distance of 0.41 was calculated (Fig 1E—blue pixel). This dependence of the distance values on the position of the hydroxyl group leads to an unwanted weighting, which is a clear problem with the approach.

In the SMILIGN algorithm, SMILES strings are treated as if they were amino acid sequences and a multiple sequence alignment was calculated with MUSCLE [25]. Similarity within pairs of lipids was calculated using an identity matrix. The SMILIGN method distinguished all 17 ceramide isomers (Fig 1F), but we noticed an irregular distribution of distance values. For example, comparing molecule pairs where the hydroxyl group was shifted by one position 11–12, 12–13, 13–14 and 14–15 resulted in four different distance values of 0.03, 0.13, 0.25 and 0.06 units respectively. In this regard, we identified two problems with the algorithm. First, there were several misalignments that lead to incorrect distances (S1 Dataset, worksheet 5). Second, one needs 35 characters to represent all the structural details of all lipid molecules of the LMSD, but the software is limited to only 20 characters and too much information is lost. To overcome these two limitations of SMILIGN, we tested two pair-wise alignment methods that do not require conversion to amino acid sequences.

With the Smith-Waterman method, pair-wise alignments are carried out directly with the SMILES strings. All ceramide isomers were distinguished, but we noticed an anomaly in distance values for molecules 17 and 18 (Fig 1G). A closer examination of the pair-wise alignments revealed an inherent issue when applying local alignment procedure to lipids. In the aligned SMILES pairs 2–17 and 2–18, the hydroxyl groups in the fatty acid were ignored, while for the pairs 2–15 and 2–16 the characters were retained (S1 Dataset, worksheet 6). The Smith-Waterman algorithm is designed to find high scoring regions in strings, so differing ends are ignored by design and not by accident. This means that functional groups at the omega position are ignored, despite their role in biology [26]. Although one could try to adjust parameters, the Smith-Waterman method is fundamentally not appropriate for this kind of comparison.

Finally, we tested the Levenshtein distance for measuring similarities between lipid molecules (Fig 1H). Unlike Smith-Waterman, the Levenshtein approach always aligns all characters for a given pair of SMILES. This method was the most successful. It distinguished all ceramide molecules and for each molecule, it correctly scored and ranked distances up to the molecule’s third closest isomers. From the fourth closest isomer onwards, a fixed distance of 0.12 was determined. Unlike other methods, it guarantees a symmetric distance matrix with no unwanted weighting of groups due to their positions. These tests of structural similarity measures led to two conclusions. First, the alignment step is necessary. Second, the Levenshtein distance was most likely to be generally applicable for all molecules in a lipidome.

### From structural similarity to chemical space

A set of distances between *n* molecules defines an (*n* − 1) dimensional space. The coordinates of molecule *i* are simply the distances to all members of the set (including the zero distance to molecule *i* itself). This is formally a vector space, in which similar molecules will have similar coordinates. It is, however, not very compact and because of structural similarities, coordinates in some dimensions would be highly correlated with others. Principal component analysis (PCA) was then used to reduce the dimensionality and see how much information would be lost. The first test was performed on a set of 16 phosphatidylinositol molecules (S1 Protocol).

**Fig 2:**
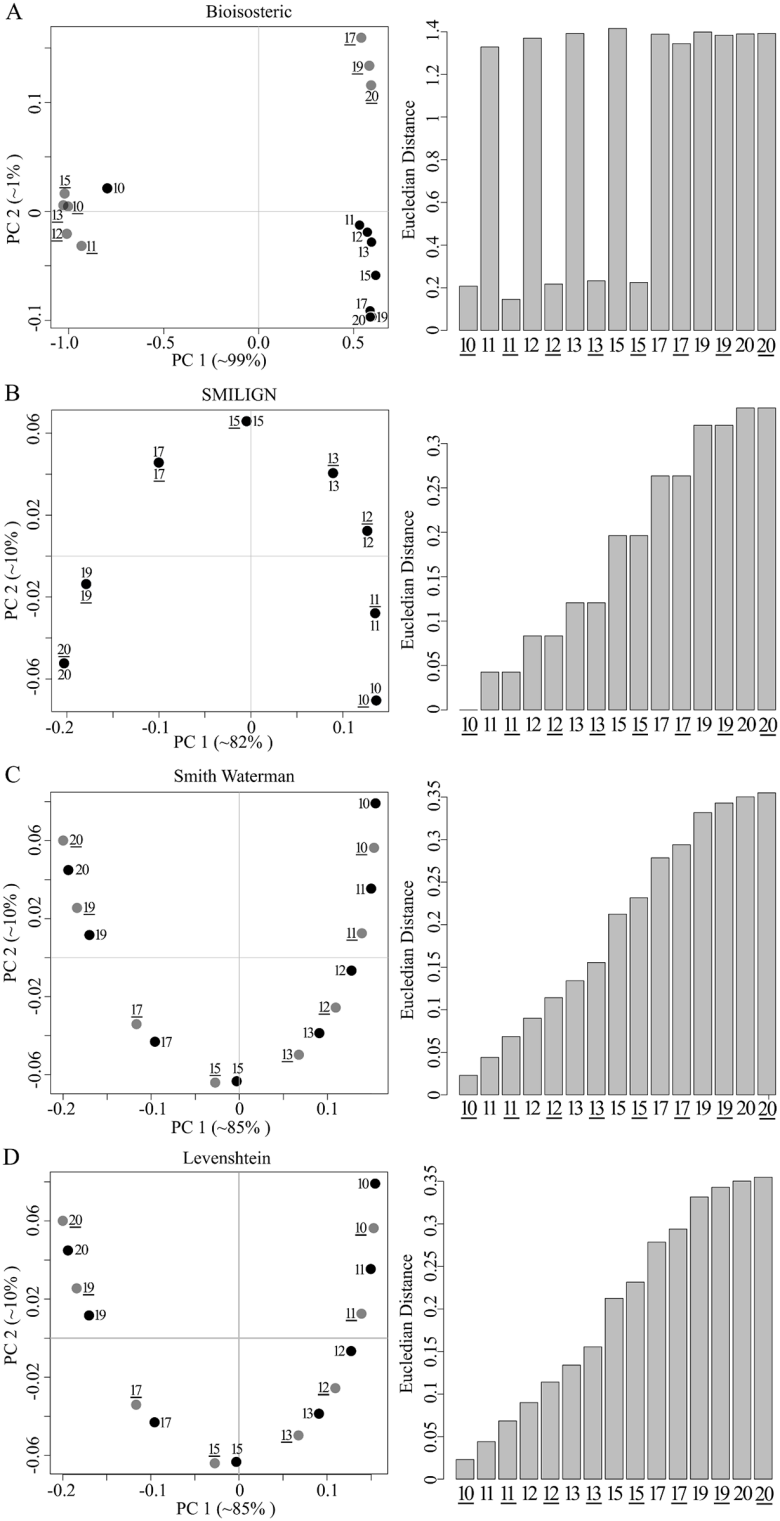
The relationship between phosphatidylinositol (PI) molecules is retained in a two dimensional structural space. Pairwise distances between 16 PI’s were calculated with Bioisosteric, SMILIGN, Smith-Waterman and Levenshtein methods (A-D). PCA was carried out on the distance matrices to generate two- and three- dimensional maps. The contribution of each principal component to the total variance is shown in brackets. Molecules with double bonds are in grey and without double bonds are in black. Euclidian distances between the first PI molecule and all others in the PC1-PC2 plane are shown as bar graphs on the right side of panels. Molecules were numbered according to the length of the *sn2* acyl chain length, wherein an underlined number *xx* indicate the presence of the double bond.

Considering just the first two principal components was sufficient to highlight problems with some of the distance measures. For example, the map in Fig 2A shows a clear weakness with the Bioisosteric method. The extension of the fatty acid chain at the *sn2* position and degree of unsaturation are not accurately represented (Fig 2A, scatter plot). We also computed the Euclidian distance between molecules in the plane of the first two components. This showed an inconsistent trend in the distance increase with each structural alteration (Fig 2A, bar graph). Principal components can often be interpreted in terms of the original descriptors and in the case of SMILIGN, the first two components are dominated by the extension of the acyl chain at the *sn2* position (Fig 2B). For SMILIGN, the first two principal components are not sufficient to distinguish molecules that differ only in the presence of a double bond, but the third principal component does capture it (S1 Fig).

In contrast, distances based on Smith-Waterman and Levenshtein algorithms reflected all gradual structural changes in the molecules (Fig 2C and 2D). In both cases, the projection leads to a set of points in a *U* shape and, if we take molecule 10 as a reference, stepwise changes to the chemistry are reflected in distinct shifts in the principal coordinates. We further recognized that the changes in coordinates, when the acyl chain is extended by two methylene groups (molecules 15–17, 17–19) are about twice as large as the difference between pairs differing by a single methylene group (Fig 2C and 2D). The first two principal components combined, accounted for 95% of the variability in the underlying data set for Smith-Waterman and Levenshtein. Summarizing the results, we see the Levenshtein method coupled with template-based SMILES as the best approach for calculating structural differences in small molecule sets. PCA is an appropriate way to reduce dimensionality and the relation between molecules can be depicted in a PCA map, which we treat as chemical space.

**Fig 3:**
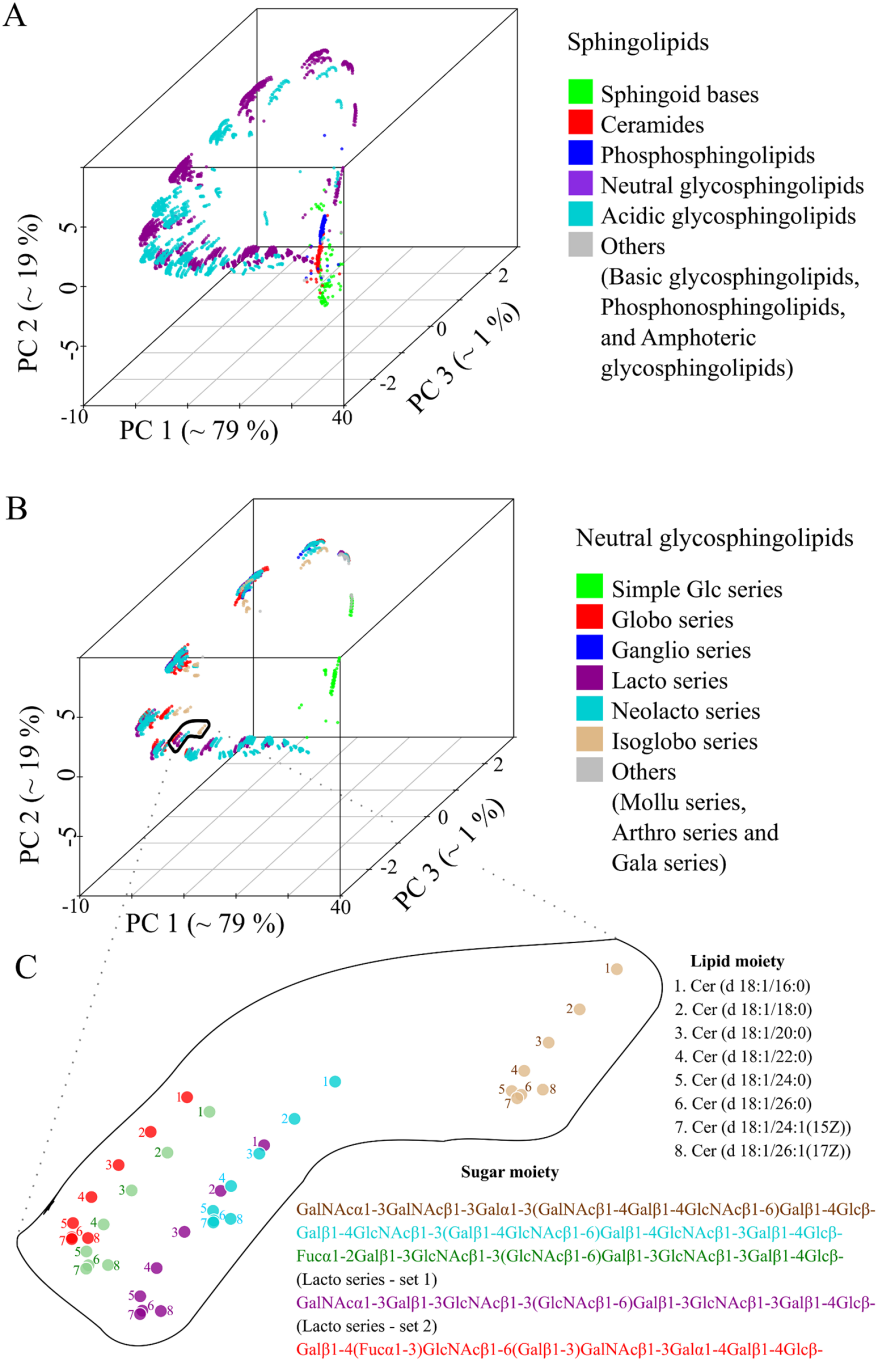
The structural space model clusters thousands of sphingolipids (SP) according to their chemical relationship. (A) Three-dimensional map of 30 510 SP obtained by PCA from a pair wise distance matrix calculated with Levenshtein distance. (B) Plot of all neutral SP within the same coordinate system as (A) indicating several associated glycosphingolipid series. (C) Complex glycosphingolipids are highlighted showing the influence of structural changes in the ceramide backbone and sugar moiety.

**Fig 4:**
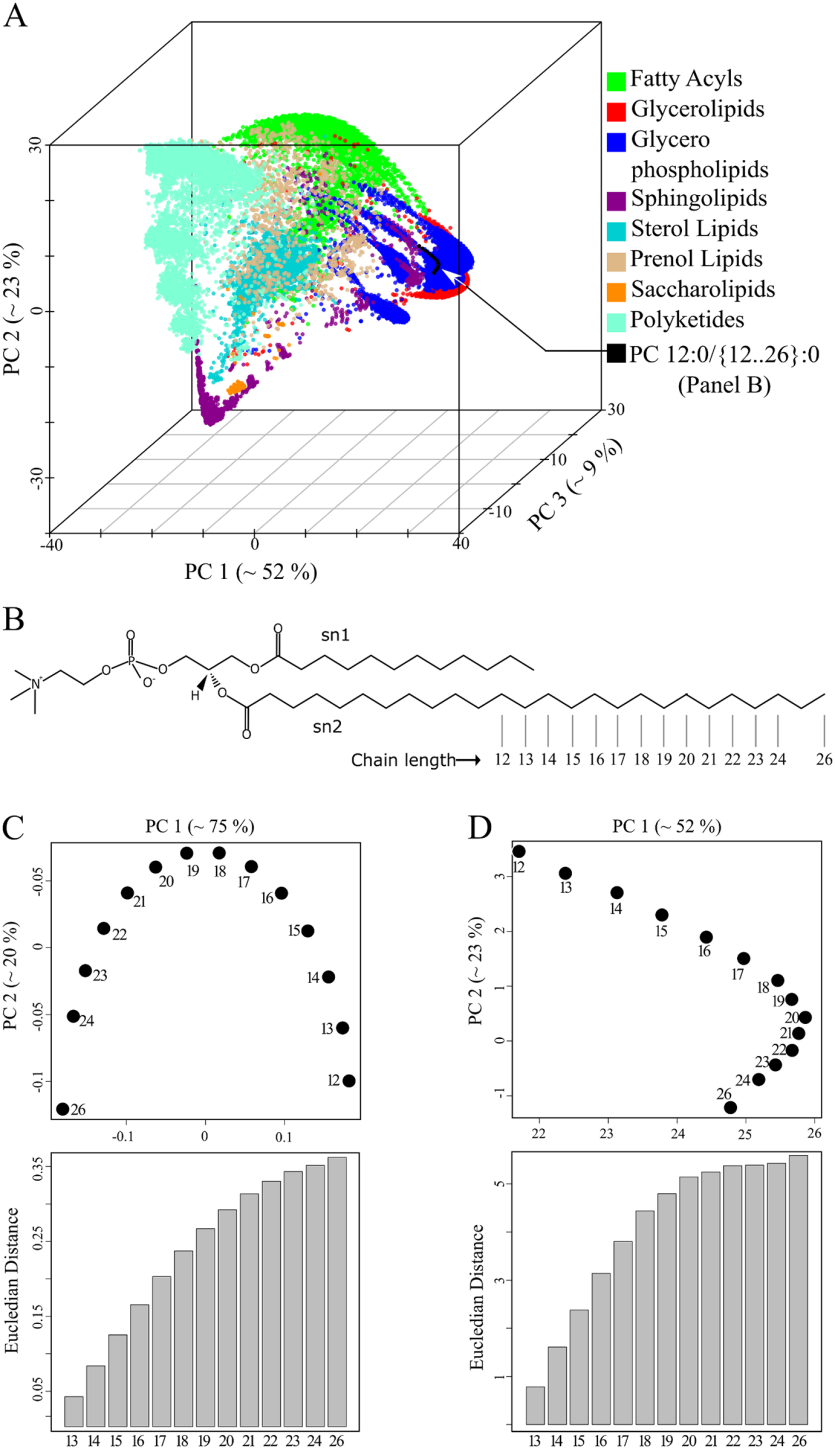
Spatial distribution of related phosphatidylcholines (PC) molecules remains stable in the background of large structure data sets. (A) Lipid map of 30 150 lipid molecules obtained from LMSD. Pairwise distances were calculated using the Levenshtein method of template-based SMILES. (B) Structures of the 14 PC molecules. Molecules are named based on the number of carbon atoms of the *sn2* acyl chain. (C) Two-dimensional map of the selected PC molecules displaying their chemical relation to each other. Euclidian distances in PC1-PC2 plane between the smallest molecule (*name 12*) and all others are shown in the bar graph. (D) Spatial distribution of 14 PC in the background of 30 136 lipids determined from three principal components and projected on the PC1-PC2 plane. Euclidian distances between first molecule (*name 12*) and all others were determined from the first two principal components and its trend is shown as bar graph.

The set of 16 phosphatidylinositol molecules is useful for highlighting details, but one is interested in using the method on much larger molecule sets. To this end, we used the 3510 sphingolipids (SP) from LMSD as a test dataset [22]. All lipid structures were converted into template-based SMILES and pair-wise distances were computed using the Levenshtein method. Fig 3A shows the position of all molecules in terms of the first three principal components. There are two clear observations. First, three principal components account for 99% of the total variance (Fig 3A) and no two SP's have the same coordinates (S2 Dataset, worksheet 1). Second, there was no biochemical knowledge put into the procedure, but the molecules naturally cluster into chemically similar groups (Fig 3A). Sphingosines, ceramides and phosphosphingolipids were clustered separately from the complex glycosphingolipids (GSL). Furthermore, the acidic and neutral GSL where placed in different clusters. Looking at the *globo*, *lacto*, *neolacto* and *isoglobo series* of neutral GSL, one can see changes in the sugar moiety and a clear separation from the simple *Glc series* (Fig 3B). This observation fits the intuitive expectation that the *Glc series* with simple sugar moieties (glucose, galactose or lactose) should be farther from lipids with complex sugars. We noted that changes in the sugar moiety of neutral GSL, which have a strong impact on biochemical behaviour, were separated by a larger distance compared to changes in the ceramide backbone (Fig 3B). In addition, we were intrigued by the recurring appearance of geometric patterns in the form of *I*, *C* and *L* shapes and investigated the structure within these clusters. Within each cluster, lipids were organized based on changes in the ceramide moiety (Fig 3C) so that, for example, eight molecules of the *isoglobo series* formed a twisted *L* shape with each successive lipid carrying a gradual change in the ceramide backbone (Fig 3C–light brown coloured points). Analogous geometric arrangements were observed for the *globo*, *lacto and neolacto series* (Fig 3C–red, violet and light-blue points). Next, we tested how all 30 150 lipids of the LMSD would be organized in a chemical space based on only structural similarity. All lipid molecules had unique coordinates in this space (S2 Dataset, worksheet 2), indicating that our approach can distinguish between all lipid structures within known, natural lipidomes. With no additional input, the method grouped lipids into clusters that correspond to the popular lipid classification of LIPIDMAPS (Fig 4A) [27].

Fatty acyls, glycerophospholipids (GPL), sphingolipids (SP) and polyketides occupied opposite ends of the chemical space (Fig 4A). In contrast, glycerolipids and GPL shared a common region because of their head group similarity. Sterol lipids formed a distinct cluster due to their unique four-ringed core structure. Prenol lipids were widely distributed in the chemical space reflecting their varying chemical composition. For GPL, we observed several distinct clusters, which on closer examination, could be recognized as spatially separated lipid classes like phosphatidylcholine (PC), phosphatidylserine (PS) and phosphatidylinositol (PI) (S2 Fig).

### The spatial organization of lipids is robust to changes in background molecule ensemble

As with the set of PI molecules described above (Fig 2D), the PC molecules in the two-dimensional representation form an inverted *U* pattern (Fig 4C). However, the PC molecules formed a flipped *L* pattern if all other 30 136 lipids of the LMSD were present (Fig 4D). In both cases (Fig 4C and 4D), the sequential arrangement of the PC molecules in the two-dimensional chemical space accurately represents the gradual increase in acyl chain length. We also observed a gradual increase of the Euclidian distance from the first PC molecule to the last (Fig 4C and 4D, bar graphs). When we gradually increased the complexity of the set of lipid molecules, we noticed that the PCA approach could disturb relationships between structurally similar molecules. In the case of a set consisting of only GPLs and only GPLs and SPs (S3 Fig), we noticed that the distances between molecule 12 and molecules 21–26 did not reflect the *sn2* chain length increase anymore. Interestingly, one can observe that the gradual addition of more diverse lipid structures spanning a broader chemical variety compensates for this bias. At the same time we recognized that for other lipid classes like cholesteryl esters and triacylglycerols (TAG) incremental changes in the acyl chains cluster together (S4 Fig). In case of TAG molecular species, a homologues series can be recognised due to the difference of one double bond on the *sn*3-linked fatty acid (S4C Fig). It seems that the Levenshtein distances and the projection to a chemical space automatically reconstructs conventional lipid class definitions while also clustering closely related molecules in accordance to chemical rules. The next natural step is to test these lipid coordinates, computed from template based SMILES and Levenshtein distances, for their suitability for analysing and comparing complete lipidomes.

### The Lipidome jUXtaposition (LUX) score, a single metric for calculating the similarity between lipidomes

**Fig 5:**
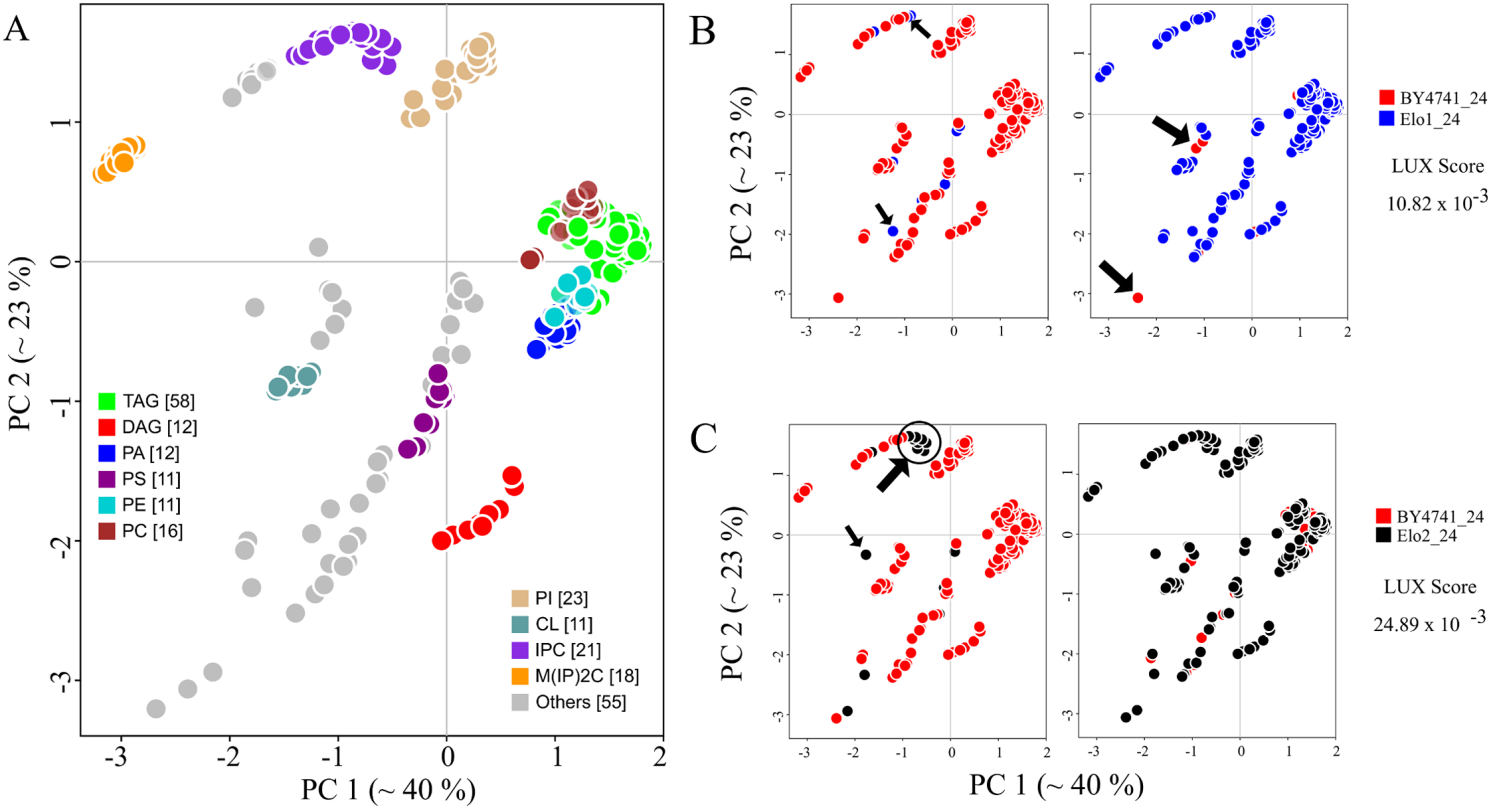
Lipidome maps highlight relationships between yeast strains. (A) All lipids from yeast strains, BY4741, Elo1, Elo2 and Elo3 cultured at 24°C and 37°C are combined to create a reference map of the yeast lipidome. Each coloured circle corresponds to a unique lipid. (B) Comparison of lipidomes from strains BY4741 and Elo1 (cultured at 24°C). Arrows in first plot indicate lipids that are present in Elo1, but not in BY4741 and vice versa in the second plot. (C) Comparison of BY4741 and Elo2 lipidomes. A two dimensional Lipidome jUXtaposition (LUX) score is calculated for a pair of lipidomes using reference-map coordinates (S3 Dataset, worksheet 3).

The approach to lipidome comparison was then tested on real data. All lipids from four yeast strains BY474, Elo1, Elo2 and Elo3 [6] were combined, yielding a reference lipidome with 248 members, each with unique coordinates (S3 Dataset, worksheet 3). For clarity, this is shown in a 2D map (Fig 5A), which is the basis of comparisons of the four strains and two culturing temperatures (24°C and 37°C). Triacylglycerols (TAG) occupy the largest area on the map in terms of the number and variety of structures. Mannose-bis(InositolPhospho)Ceramides [M(IP)_2_C] form a distinct cluster located in the top-left quadrant of the reference map. In the top right quadrant of the reference map, there is a cluster of GPLs consisting of phosphatidic acid (PA), phosphatidylethanolamines (PE), phosphatidylcholines (PC) and TAG. The reference lipidome map clearly shows temperature- and strain-specific changes. The lipidomes of the wild type yeast strains BY4741 and Elo1 grown at 24°C showed only minor differences (Fig 5B). In contrast, the lipidome of the Elo2 mutant is very different to the wild type strain BY4741 (Fig 5C). The mutation has led to dramatic changes amongst the inositol phosphorylceramides (IPC) seen in the top-left quadrant and the appearance of new species not present in the wild type. Using this well-defined lipidome map, one can determine the closest related lipid in the wild type strain. If one calculates the distances that lipids would have to move to make the members of each pair overlap, one can use the Hausdorff distance to compare lipidomes (Fig 5C and 5D, arrow marked lipids). For that, we chose the coordinates of all lipids in the two dimensional coordinate system of the first lipidome and determined the Euclidean distance to its closest structural neighbour in the second. Subsequently, the average of all distances was determined, including all distance values of zero for identical molecular species. Because the Hausdorff distance depends on the direction of the comparison, we used the maximum of the two values (max(*d*_*AB*_, *d*_*BA*_)). We named this measure as the ‘Lipidome jUXtaposition (LUX) score’. This score is a distance, so larger values indicate more dissimilarity and identity results in a LUX score of zero. From that perspective, one can see that the LUX score between BY4741 and the Elo2 strain is three-fold larger than the distance between BY4741 and Elo1 (Fig 5B and 5C).

**Fig 6:**
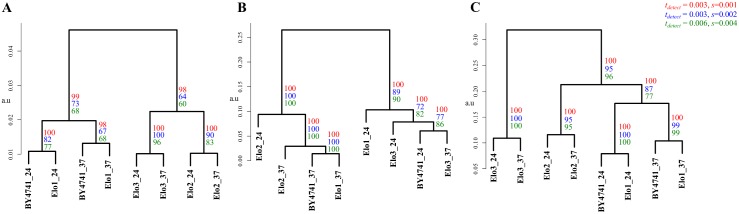
The LUX score accounts for genetic alteration of yeast strains. For the yeast strains, BY4741 (wild type), Elo1, Elo2 and Elo3 (*Elongase* mutants) cultured at 24°C and 37°C, dendrograms were computed from two-dimensional LUX scores (A) Comparing concentrations of common lipids (B) and counting the percentage of common lipids in a pair of lipidomes (C). All dendograms are based on complete linkage using Euclidean distance as the similarity metric (a.u—arbitrary units). The number of occurrences for each branch in 100 iterations is indicated with coloured numerals that correspond to the utilized parameter set for detection threshold *t*_*detect*_ and standard deviation *s* applied in the error model (see methods).

Next we evaluated the LUX score by computing a hierarchical clustered tree of all eight reported lipidomes of yeast (Fig 6A) and compared it to dendrograms based on the lipid concentrations (Fig 6B), and by simply counting common lipids (Fig 6C). This allowed us to test if our approach can correctly depict the genetic and phenotypic relationship between the yeast strains reported earlier [6]. A dendrogram based purely on correlation of lipid abundances would neglect the structural changes in the lipidome. This approach implies that the largest difference can be found between BY4741 and Elo1 mutants grown at 24°C and 37°C (Fig 6B). Alteration in lipid class profile as well as increased fatty acid length and less double bond content was reported in response to increased growth temperatures [9]. This response however, is well captured in a solely quantity-based lipidome comparison (Fig 6B) because most of the molecular species are present at different temperatures. The tree computed from the LUX score as well as common lipid count indicates that mutation of the Elo1 gene had less influence on the composition of the lipidome than the temperature shift. Both strains, BY4741 and Elo1 were closest neighbours to each other at the culturing temperature of 24°C and 37°C. The lipidomes from mutant strains Elo2 and Elo3 were clustered together using the LUX score (Fig 6A) but in counting common lipids, Elo2 clustered with BY4741 and Elo1 (Fig 6C). This marks the major difference between both metrics. It was reported that no aberrant phenotype for Elo1 was observed and that Elo2 and Elo3 had distinct alterations in their intracellular organization [6,28], which seems better represented with the LUX score. However, we verified this finding with an error model that modify only the presence and quantity for low abundant lipids to estimate a robustness for the observed clustering. One can recognize that the LUX score (Fig 6A) as well as the common lipid count (Fig 6C) comprise a sufficiently robust tree topology and groups Elo2 systematically different. We concluded from this experiment that compositional differences itself are useful to assign a phenotype while comparison purely based on quantities are dominated by changes of abundant lipids (Fig 6B). We also note that just counting of lipids is a simplistic, binary measure of compositional differences. In contrast, the LUX score provides a refined measure of lipidome structural diversity, which we recognize as an advantage.

The complexity of the yeast lipidome comparison is relatively small compared to higher organisms. Nevertheless, the two-dimensional structural space reflecting 63% of the overall variability of the dataset (Fig 5A) is sufficient to determine lipidome similarities based upon the LUX score. We also note that the tree topology does not change whether one uses just three principal components (covering 83% of the variability) or the original pairwise distance matrix (S4 Dataset).

### The LUX score enables tissue lipidome comparison

**Fig 7:**
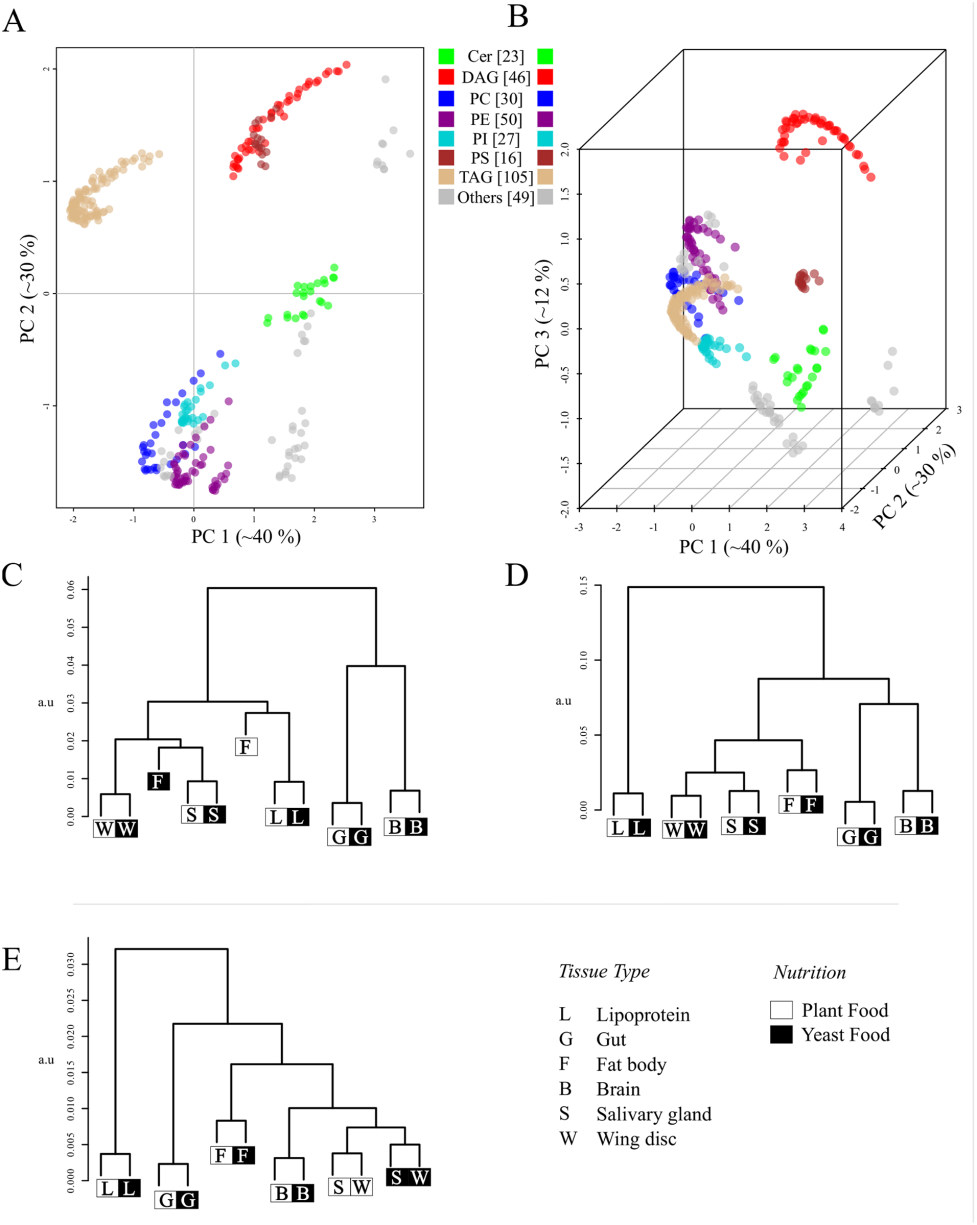
Hierarchical clustering of Drosophila tissue lipidomes based on the LUX score. (A) Two dimensional reference lipidome map of 6 Drosophila larval tissues (gut, brain, wing disc, salivary glands, fat bodies and lipoprotein) grown on Yeast Food (YF) and Protein Food (PF). (B) The same reference lipidome depicted in the three dimensions. The PC1/PC2 plane is situated on the bottom of the three dimensional representation. Dendrogram were computed on the basis of the pairwise LUX derived from the two dimensional (C) and three dimensional chemical space model (D). (E) Hierarchical cluster tree based on the matrix of pairwise Levenshtein distances of all 346 lipid molecular species. All dendrograms are based on complete linkage using Euclidean distance as the similarity metric (a.u—arbitrary units).

Next, we tested the LUX score on the more complex tissue lipidome of Drosophila [8]. In this experiment the lipidome composition of six different larval tissues (gut, lipoprotein, fat body, salivary gland, wing, disc, and brain) were determined in conjunction with two nutrition regimens (yeast food and plant food). In contrast to the yeast dataset, the fatty acid composition and sphingosine structures were not defined, so lipid structures were compiled based on prior knowledge detailed in S3 Protocol. In our analysis, we included 346 lipids structures of 12 classes, which corresponds to 97.2% coverage of the reported lipidome. The structural repertoire of the larval tissue lipidomes is shown in Fig 7A and 7B. For visualization purposes, one is obviously bound to two and three dimensional representation, but because of the set's complexity, we prefer the LUX score based on the original high-dimensional distances for biological interpretation. As one would expect, the dendrogram topology is somewhat sensitive to the dimensionality of the LUX score (Fig 7C–7E). Nevertheless, interesting properties of the larval tissue lipidome are recognizable in all dendrograms. The fat body shows the strongest influence regarding the nutrition regimen of all tissues (Fig 7C–7E). This is consistent with the expectation that the primary storage organ of the larvae reflects the nutritional differences more strongly than other tissues. All other tissues exhibit a systematic compositional shift, but with less than half the LUX score value. Interestingly, the gut lipidome was the least affected by the nutrition, which points to its function as barrier organ, where the lipid composition is tightly controlled to maintain cell membrane integrity. We further recognize that the salivary glands and wing discs lipidomes are clustered together. Using the complete distance matrix, these are the only tissue where the compositional shift induced by the nutrition is scored slightly higher than tissue lipidome similarity (Fig 7E). That might point to the lower structural specificity of the LUX score, that has to be expected because fatty acid composition of the phospholipids and sphingolipids were not experimentally defined. However, even with this limitation, one can cluster lipidomes in a manner similar to gene expression analysis.

## Discussion

Our study offers a general approach to characterizing and comparing lipidomes based on the structures of their constituents. It is certainly useful for making function/phenotype associations and allows one to correlate changes with habitat, genetic relationships and environmental stresses. The approach is dependent on the initial SMILES strings which is both an advantage and possible weakness. One can compare the issue with small molecule classification. There, the problem is sometimes easier, especially when one is dealing with derivatives which are closely related, but even in small molecule cheminformatics, there is no universally accepted scheme [29]. Optimization of such structures often depends on the interaction sites of a protein and pharmaceutical requirements for administration of drugs [30,31]. In this study, the analysis does not stop after comparing the details of individual structures. The aim is whole lipidome comparison and these are sets of structures whose members are functionally related. In this work, we took advantage of a SMILES generation scheme, which works well on large sets, but there will probably be pathological examples where it does not perform well. It definitely seems useful when working with lipids where it reflects 1) chain length 2) double bond position and 3) bond frequency. However, lipids are special with regards to their structural diversity, and some better similarity metric might be available in future. Here, a combination of SMILES with SMARTS will allow a weighting of structural alteration of lipids similar to usage of substitution matrices in sequence analysis. In this regard, lipidome research is at an infancy stage where appropriate weights cannot yet be automatically predicted on basis of experimental data.

The definition of structural similarity and the chemical space model concisely depict the complexity of a lipidome. The projection down to two- and three-dimensional maps lead to clusters that fit standard lipid nomenclature, so one can quickly see qualitative differences between lipidomes. The reference map for multiple comparisons also shows changes in the overall organization of a lipidome, which can support functional association related to membrane organization and metabolic adaptations. Consequently, the LUX score enables the standardization of lipidome comparisons outside of conventional correlation based approaches. The yeast lipidome comparison can be seen as a model for an evolutionary change in lipid biosynthesis where the mutations in Elo2 and Elo3 induce new structural variants. With the chemical space model such molecules are placed in relation to the complete lipidome (Fig 5C). In this way, alterations in the structural space can be objectively calculated and findings for interspecies, cell type and cell compartment lipidome analyses can be depicted in a well-defined graphical illustration. At the same time, the LUX score workflow is customizable in regards to the complexity of the lipidome study (S5 Fig). We further show that the LUX score approach is compatible with high-throughput lipidomics. However, we note that it is preferable to utilize lipidomics data where fatty acid and sphingosine compositions are experimentally determined. For the analysis of compositional differences between lipidomes and its interpretation, we recommend to apply an error model as introduced in this study. We recognized that clustering approaches are often not verified with an error model, which negatively affects the value of subsequently derived biological and/or medical interpretations.

The LUX score based lipidome comparison is based solely upon an identity matrix for exchange values, which does not account for quantitative changes. This is parsimonious, but obviously not optimal for capturing the complexity of lipidomes. In future work, we will test how quantitative changes should be weighted with respect to changes in the structural composition of a lipidome. We will estimate such weight measures from well understood model systems based on larger data sets that are now becoming available [32,33]. However, this study shows that the structural arrangement of a lipidome is sufficient to recognize the degree of genetic alteration and temperature dependence in yeast in an unsupervised manner. We further show that approach is applicable for high-throughput lipidomics.

In summary, we see potential in the LUX score to identify evolutionarily conserved compositional constraints that are linked to cellular functions. This is of special importance for studying the lipid metabolism in animal models of human diseases, where inherent lipidome differences should be considered for developing new therapeutic strategies. To utilize the full potential of lipidome homology metrics in a biological context, improvements in lipidomics technology and reporting standards have to be made in analogy to the present wealth of available genomics data [37]. In this regard, our approach enables to estimate and compare the structural complexity of lipidomes, which can nurture systems-biology approaches. The chemical space model of a lipidome and the LUX score will facilitate inter-species functional association that are applied in comparative genomics.

## Methods

Details of SMILES generation, principal component analysis (PCA), structural similarity methods and annotation of lipids are given in supplementary methods (S2 Protocol).

### Lipid structure datasets

Lipid structures for Figs 1 and 2 were drawn and SDF files were generated using PubChem Sketcher [34]. The complete LIPIDMAPS Structure Database (LMSD) in SDF format was downloaded on Nov 9, 2011 from www.lipidmaps.org (LMSDFDownload9Nov11.zip) [22].

Lipidome data of yeast mutants was taken from Ejsing et.al. [6]. LIPIDMAPS structure drawing tools were customized to draw all required lipid structures for yeast. For ergosterol and ergosta-5,7-dien-3β-ol, SDF files were obtained from the LMSD. SMILES for phytosphingosine 1-phosphate was made by hand from the corresponding phytosphingosine. For some molecules, the number of hydroxylations and double bonds was known, but their position was not. In these cases, a list of possible isomers was generated. The position of double bonds and hydroxylations in yeast fatty acids were taken from previous studies [35]. Pairwise distances between all isomers were calculated using the Levenshtein distance method [19,20]. The isomer with smallest average distance to other isomers was chosen as representative molecule (S2 Protocol).

Lipidome data for Drosophila is based on Carvahlo et al. [8] described in detail in S3 Protocol.

### Template-based SMILES

LIPIDMAPS perl scripts were modified to generate a wider spectrum of lipid structures [23,24]. These scripts are provided in supplementary information (S5 Dataset) and also available at http://lux.fz-borstel.de. Molecular structures in SDF format were converted to SMILES using the OpenBabel molecular conversion tool with the default algorithm [16]. Characters indicating chirality, cis–trans isomerism and charges were removed automatically for the yeast lipidome analysis.

### Structural similarity scoring methods

Six similarity scoring methods were tested 1) OpenBabel FP2 Fingerprint 2) LINGO 3) Bioisosteric similarity 4) SMILIGN 5) Smith Waterman Local Alignment 6) Levenshtein distance. Details are given in supplementary methods (S1 Methods). The Levenshtein method was applied for analysing the LMSD, yeast and Drosphila lipidome (Figs 4–7). This algorithm was originally designed for correcting spelling errors, but the principle can be applied to compare any pair of strings including SMILES [19,20]. The source code used in this study is provided in supplementary information (S5 Dataset) and also at the website http://lux.fz-borstel.de.

### Lipidome Juxtaposition Score (LUX) calculation

The LUX score is based on the Hausdorff distance [36] and summarizes the similarity between lipidomes. In pseudocode, the distance from lipidome *A* to *B* is calculated from:

for each lipid in A

 find distance *d* to most similar lipid in B

*d*_sum_ ∶ = *d*_sum_ + *d*

*n* = *n* +1

return *d*_sum_/*n*

This yields the average shortest distance *d*_*AB*_ from A to B. The larger of *d*_*AB*_ and *d*_*BA*_ was used as the lipidome homology score (*AB*).

### Hierarchical cluster analysis

Complete linkage clustering was performed with R, version 2.14.1, library–‘stats’ and function ‘hclust’ using the LUX score, Pearson and common lipid count as similarity metrics. An error model for the yeast lipidome analysis was computed by iterating all lipid quantities *x* of the original data set according to:

for each lipid with abundance *x*

*x'* = *x* + rnorm(1,0,*s*)

 if *x'* > *t*_*detect*_

return *x'*

The detection limit *t*_*detect*_ and standard deviation *s* were defined so that only low abundant lipids were significantly affected. We chose the following three parameter sets: 1) *t*_*detect*_ = 0.003 mol %- 4.3% of all reported quantities, *s* = 0.001mol %- 11.4% of all reported standard deviations 2) *t*_*detect*_ = 0.003 mol %, *s* = 0.002 mol%- 20.3% of all reported standard deviations and 3) *t*_*detect*_ = 0.006 mol%- 8.7% of all reported quantities, *s* = 0.004 mol%- 34.7% of all reported standard deviations. The number of occurrences for each branch was counted after 100 iterations using the R library, ape::boot.phylo::prop.part.

## Supporting Information

S1 Table Main structural features of abundant membrane lipids in mammals, drosophila and yeast. (PDF)

S1 Protocol Procedure for template-based SMILES generation. (DOCX)

S2 Protocol Supplementary methods. Includes computer configuration, annotations and abbreviations used in the manuscript.
(DOCX)

S3 Protocol Compilation of the Drosophila larvae tissue lipidome data. (DOCX)

S1 Dataset SMILES, distance matrices and alignments for Fig 1. Excel worksheets are organized as follows: (1) Input SMILES. Pairwise distances between for input SMILES calculated with FP2 Fingerprint (2) LINGO (3) Bioisosteric distance (4) SMILIGN (5) Smith-Waterman (6) and Levenshtein (7). Problems associated with Multiple Sequence Alignment approach used in SMILIGN method are highlighted in sheet (5) and problems associated with local alignment approach of Smith-Waterman in sheet (6).
(XLSX)

S2 Dataset SMILES and coordinates for Figs 3 and 4. Worksheet 1 contains *x*, *y* and *z* coordinates of 3510 Sphingolipids and worksheet 2 for all 30 150 lipids of LIPIDMAPS Structure Database.
(XLSX)

S3 Dataset SMILES and coordinates for Figs 5 and 6. Worksheet 1: Representative isomer for all lipids in yeast lipidome. Worksheet 2: Template-based SMILES for yeast lipids. Worksheet 3: PCA coordinates for all lipids in yeast lipidome.
(XLSX)

S4 Dataset The relation between the yeast lipidomes is not influenced by the number of dimensions used (matrices for Fig 6.) Use the files Yeast_Lipidome_Homology_Scores.htm (Firefox Browser suggested) or Yeast_Lipidome_Homology_Scores.xlsx (Excel 2013) to navigate through the complete yeast lipidome dataset. Plots and additional distance matrices are linked.
(ZIP)

S5 Dataset Source code. Includes scripts, README files and data files for Figs 1, 2, 6, 7 and S6.
(ZIP)

S1 Fig Three-dimensional representation of 16 phosphatidylinositol molecules in chemical space (Fig 2A) using SMILIGN method. Underlined molecules indicate the presence of the double bond.
(TIF)

S2 Fig Spatial distribution of glycerophospholipid classes. Pairwise distances between 30 150 lipid molecules of LMSD were determined using the Levenshtein method. Only glycerophospholipids are depicted.
(TIF)

S3 Fig Relative position of 14 PC molecules in the background of large and diverse lipid structure sets. (A) Map of 14 phosphatidylcholine (PC) molecules (Fig 4B) in the background of 7744 Glycerophospholipids. (B) Distribution of the 14 PC molecules in the background of 11 268 Glycerophospholipids and Sphingolipids. (C) Map of 14 PC molecules in the background of 15 304 Glycerophospholipids, Sphingolipids and Fatty acyl molecules. All structures were obtained from LMSD and converted to non-canonical SMILES. Pair wise distances were calculated with Levenshtein method. First row of plots show 14 PC molecules along with indicated lipid structure classes. Second row plots show only 14 PC molecules maintaining the coordinates of corresponding first row plots. Third row of plots show Euclidean distance between first molecule (name 12) and other 13 PCs. Euclidean distance is calculated in Principal Component 1 and Principal Component 2 plane as shown in second row of plots.
(TIF)

S4 Fig Spatial distribution of selected triacylglycerol and cholesteryl esters. (A) Pairwise distances between 30 150 molecules from LIPIDMAPS Structure Database were determined using Levenshtein distance. Principal Component Analysis was carried out on pairwise distance matrix and the first three components were plotted. Selected triacylglycerols (TAG) and cholesteryl esters (CE) are highlighted while the remaining 30 131 lipids were shown as grey dots. (B) Spatial distribution of 9 CEs differing in the fatty acid chain length (12–22 carbon atoms). Molecule coordinates are the same in Panels A and B. (C). Spatial distribution of 10 TAGs with C16:0 fatty acid in *sn*1 and *sn*2 position and third fatty acid chain length varying from 16 to 22 carbon atoms and one double bond. (D) and (E) Manually rendered plots based on the screen-capture of panels B and C respectively (Original screen-captures are shown as inserts). Please note that panels D and E are manually rendered, hence, slight differences in the coordinates might occur.
(TIF)

S5 Fig Workflow to determine homology between lipidomes. (A) All identified lipid molecular species of a lipidome are combined into a non-redundant list (B). (C) If the lipidomics data is not sufficient to describe the molecular species, all possible isomer structures are inferred from literature and a representative isomer structure is chosen for each lipid. (D) Template-based SMILES are generated for each chosen lipid isomeric structure as the basis for the determination of the structural similarity between all pairs of molecules using Levenshtein distance method (E). (E-F) Pair-wise LUX scores are determined for all lipidome pairs. (G) Hierarchical clustering of LUX scores is performed to depict homology between lipidomes.
(TIF)
