# Supplementary material for: The LUX Score: A Metric for Lipidome Homology
Source: PLoS Comput Biol. 2015 Sep 22;11(9):e1004511. doi: 10.1371/journal.pcbi.1004511 (PMC4578897; doi:10.1371/journal.pcbi.1004511)
Supplement: S1 Table — (PDF) [file pcbi.1004511.s001.pdf]

Supplementary Table 1.
Main structural features of abundant membrane lipids in mammals, drosophila and yeast.

	**S. Cerevisiae**	**D. melanogaster**	**Mammalian**
GPL<br>FA double bonds<br>FA length	PI, PC, PE, PA, PS<br>1 DB maximum<br>C10-C18	PE, PC, PI, PS, PG, PA<br>3 DB maximum<br>C14-C20	PC, PE, PI, PS, PG, PA<br>6 DB maximum<br>C14-C22
main membrane sterol	Ergosterol	Ergosterol	Cholesterol
main sphingolipid<br>sphingoid base length<br>sphingoid base DB	M(IP)2C<br>(C18, C20)<br>no double bond	CerPE<br>short (C14, C16)<br>1-2 DB<br>(conjugated DB)	SM<br>(C18)<br>1 DB

DB – double bond, **PL** main abundand glycerophospholipid class (GPL)

Ref: Ejsing CS et al. PNAS 2009; Guan XL et al. Methods Enzymol. 2010; Carvalho et al. Mol. Syst. Biol. 2012;
