# Supplementary material for: The LUX Score: A Metric for Lipidome Homology
Source: PLoS Comput Biol. 2015 Sep 22;11(9):e1004511. doi: 10.1371/journal.pcbi.1004511 (PMC4578897; doi:10.1371/journal.pcbi.1004511)
Supplement: S1 Protocol — (DOCX) [file pcbi.1004511.s002.docx]

**Template-based structure drawing enables consistent SMILES generation.**

We tested how SMILES can be consistently generated for lipid structures. In the first experiment (Fig. 1) we drew isomeric structure for one ceramide molecule [Cer (d16:1 / 18:0 – OH)] using PubChem Sketcher. Structure in SDF format were then converted into SMILES using the default OpenBabel SDF to SMILES conversion algorithm. Using the SMILES of the ceramide molecule as a template, we generated SMILES for 16 isomers – differing in the position of hydroxyl group (Fig. 1A-B). Next, we converted template-based SMILES into canonical SMILES in two ways 1) using OpenBabel’s SMILES to canonical SMILES conversion algorithm 2) using CACTVS SMILES to canonical SMILES writing algorithm (Ihlenfeldt *et al.*, 1994). In Table 1 (below), we show template-based SMILES and CACTVS canonical SMILES of 17 isomers of a ceramide molecule [Cer (d16:1 / 18:0 - OH)]. For molecules **2-6**, the template-based SMILES and CACTVS canonical SMILES are the same, but from molecules **7** until **16,** the chains are swapped. Furthermore, for CACTVS canonical SMILES of the molecules **17** and **18,** the hydroxyl group is not indicated as a side chain anymore. Table I shows that canonicalization algorithms interfere in the writing SMILES in a consistent way.

**Table I: Canonicalization algorithm changes order of ASCII characters in SMILES**

| **Hydroxyl-positions / Ceramide-isomer** | **Template-based SMILES** | **CACTVS canonical SMILES** |
| --- | --- | --- |
| **2** | CCCCCCCCCCCCCCCCC(**O**)C(=O)NC(CO)C(O)C=CCCCCCCCCCCC | CCCCCCCCCCCCCCCCC(**O**)C(=O)NC(CO)C(O)C=CCCCCCCCCCCC |
| **3** | CCCCCCCCCCCCCCCC(**O**)CC(=O)NC(CO)C(O)C=CCCCCCCCCCCC | CCCCCCCCCCCCCCCC(**O**)CC(=O)NC(CO)C(O)C=CCCCCCCCCCCC |
| **4** | CCCCCCCCCCCCCCC(**O**)CCC(=O)NC(CO)C(O)C=CCCCCCCCCCCC | CCCCCCCCCCCCCCC(**O**)CCC(=O)NC(CO)C(O)C=CCCCCCCCCCCC |
| **5** | CCCCCCCCCCCCCC(**O**)CCCC(=O)NC(CO)C(O)C=CCCCCCCCCCCC | CCCCCCCCCCCCCC(**O**)CCCC(=O)NC(CO)C(O)C=CCCCCCCCCCCC |
| **6** | CCCCCCCCCCCCC(**O**)CCCCC(=O)NC(CO)C(O)C=CCCCCCCCCCCC | CCCCCCCCCCCCC(**O**)CCCCC(=O)NC(CO)C(O)C=CCCCCCCCCCCC |
| **7** | CCCCCCCCCCCC(**O**)CCCCCC(=O)NC(CO)C(O)C=CCCCCCCCCCCC | CCCCCCCCCCCC=CC(O)C(CO)NC(=O)CCCCCC(**O**)CCCCCCCCCCC |
| **8** | CCCCCCCCCCC(**O**)CCCCCCC(=O)NC(CO)C(O)C=CCCCCCCCCCCC | CCCCCCCCCCCC=CC(O)C(CO)NC(=O)CCCCCCC(**O**)CCCCCCCCCC |
| **9** | CCCCCCCCCC(**O**)CCCCCCCC(=O)NC(CO)C(O)C=CCCCCCCCCCCC | CCCCCCCCCCCC=CC(O)C(CO)NC(=O)CCCCCCCC(**O**)CCCCCCCCC |
| **10** | CCCCCCCCC(**O**)CCCCCCCCC(=O)NC(CO)C(O)C=CCCCCCCCCCCC | CCCCCCCCCCCC=CC(O)C(CO)NC(=O)CCCCCCCCC(**O**)CCCCCCCC |
| **11** | CCCCCCCC(**O**)CCCCCCCCCC(=O)NC(CO)C(O)C=CCCCCCCCCCCC | CCCCCCCCCCCC=CC(O)C(CO)NC(=O)CCCCCCCCCC(**O**)CCCCCCC |
| **12** | CCCCCCC(**O**)CCCCCCCCCCC(=O)NC(CO)C(O)C=CCCCCCCCCCCC | CCCCCCCCCCCC=CC(O)C(CO)NC(=O)CCCCCCCCCCC(**O**)CCCCCC |
| **13** | CCCCCC(**O**)CCCCCCCCCCCC(=O)NC(CO)C(O)C=CCCCCCCCCCCC | CCCCCCCCCCCC=CC(O)C(CO)NC(=O)CCCCCCCCCCCC(**O**)CCCCC |
| **14** | CCCCC(**O**)CCCCCCCCCCCCC(=O)NC(CO)C(O)C=CCCCCCCCCCCC | CCCCCCCCCCCC=CC(O)C(CO)NC(=O)CCCCCCCCCCCCC(**O**)CCCC |
| **15** | CCCC(**O**)CCCCCCCCCCCCCC(=O)NC(CO)C(O)C=CCCCCCCCCCCC | CCCCCCCCCCCC=CC(O)C(CO)NC(=O)CCCCCCCCCCCCCC(**O**)CCC |
| **16** | CCC(**O**)CCCCCCCCCCCCCCC(=O)NC(CO)C(O)C=CCCCCCCCCCCC | CCCCCCCCCCCC=CC(O)C(CO)NC(=O)CCCCCCCCCCCCCCC(**O**)CC |
| **17** | CC(**O**)CCCCCCCCCCCCCCCC(=O)NC(CO)C(O)C=CCCCCCCCCCCC | CCCCCCCCCCCC=CC(O)C(CO)NC(=O)CCCCCCCCCCCCCCCC(C)**O** |
| **18** | C(**O**)CCCCCCCCCCCCCCCCC(=O)NC(CO)C(O)C=CCCCCCCCCCCC | CCCCCCCCCCCC=CC(O)C(CO)NC(=O)CCCCCCCCCCCCCCCCC**O** |

We then performed second experiment using a set 16 phosphatidylinositol (PI) molecules (Figure IA). We followed the procedure similar to the set of ceramides isomers described above, generating template-based SMILES manually using PubChem Sketcher and OpenBabel for the first PI molecule [PI (10:0 / 10:0)]. Using this SMILES as a template, we stepwise extended the acyl chain at the sn2 position and added a double bond, creating a set of 16 SMILES. Next, we generated CACTVS canonical SMILES and the OpenBabel canonical SMILES as described previously for the ceramide molecules.

**
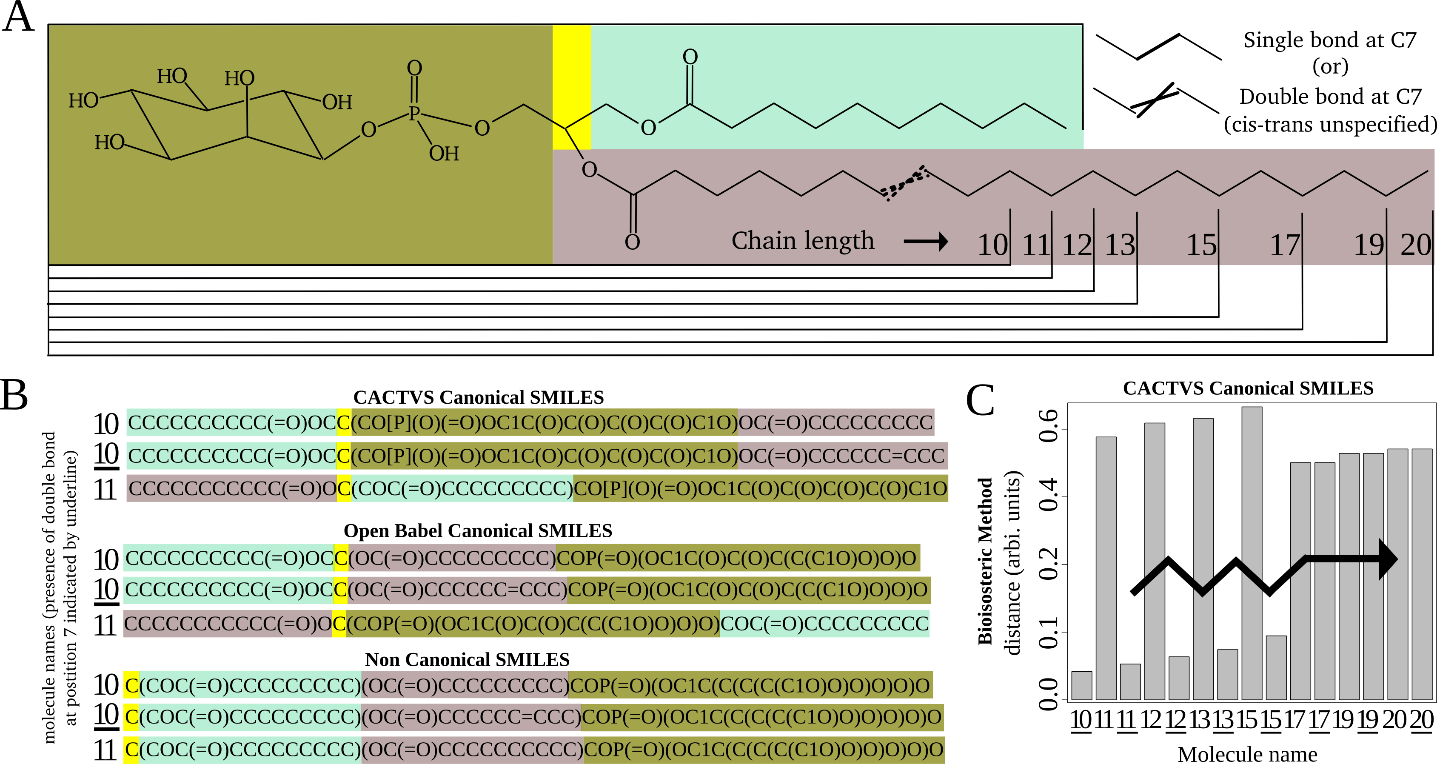
**

**Figure I - Conversion of lipid structures into SMILES must be performed without canonicalization.**

A. Structural representation of 16 phosphatidylinositol (PI) molecules varying in sn2 acyl chain length and degree of unsaturation. B. CACTVS canonical, Open Babel canonical and non-canonical SMILES representation of first three molecules. C. Distances between first molecule, PI (10:0/10:0) and others using the Bioisosteric method. Molecules were numbered according to the length of the sn2 acyl chain length and an underlined number XX indicates the presence of the double bond.

We observed rearrangements within acyl chain strings in CACTVS and OpenBabel canonical SMILES of molecules 10 and 11 (Figure I B). In this respect, results from the Bioisosteric method utilizing CACTVS canonical SMILES clearly show the problem with this approach (Figure IC). Between the closely related molecules 10 and 11 a distance of 0.39 units was determined, but only 0.05 between 10 and 11. That is, adding a double bond to molecule 11 leads to a 7-fold change in the distance to the reference molecule 10. For the molecule pairs 10/17 and 10/17 the same distance of 0.36 units was determined (Figure IC). Furthermore, rearrangements were observed throughout the dataset for both OpenBabel and CACTVS canonical SMILES (Figure II).

Canonicalization algorithms were originally designed for storing large ensembles of chemical structures in a database by generating one unique SMILES for a given molecule (Morgan, 1965; O’Boyle, 2012). Our results suggest that the canonicalization algorithms are not appropriate for writing consistent SMILES. From these two experiments we concluded that template-based SMILES display structural difference(s) between closely related lipids meaningfully. At this point, we came across template-based structure generation tool by LIPIDMAPS consortium (Sud *et al.*, 2012). Here, we recognized that template-based structures should be the basis for writing SMILES to overcome the problems brought about by canonicalization algorithms. Consequently, we utilized template-based SMILES as basis for our experiments concerning lipid similarity metrics and development of a lipidome homology score.


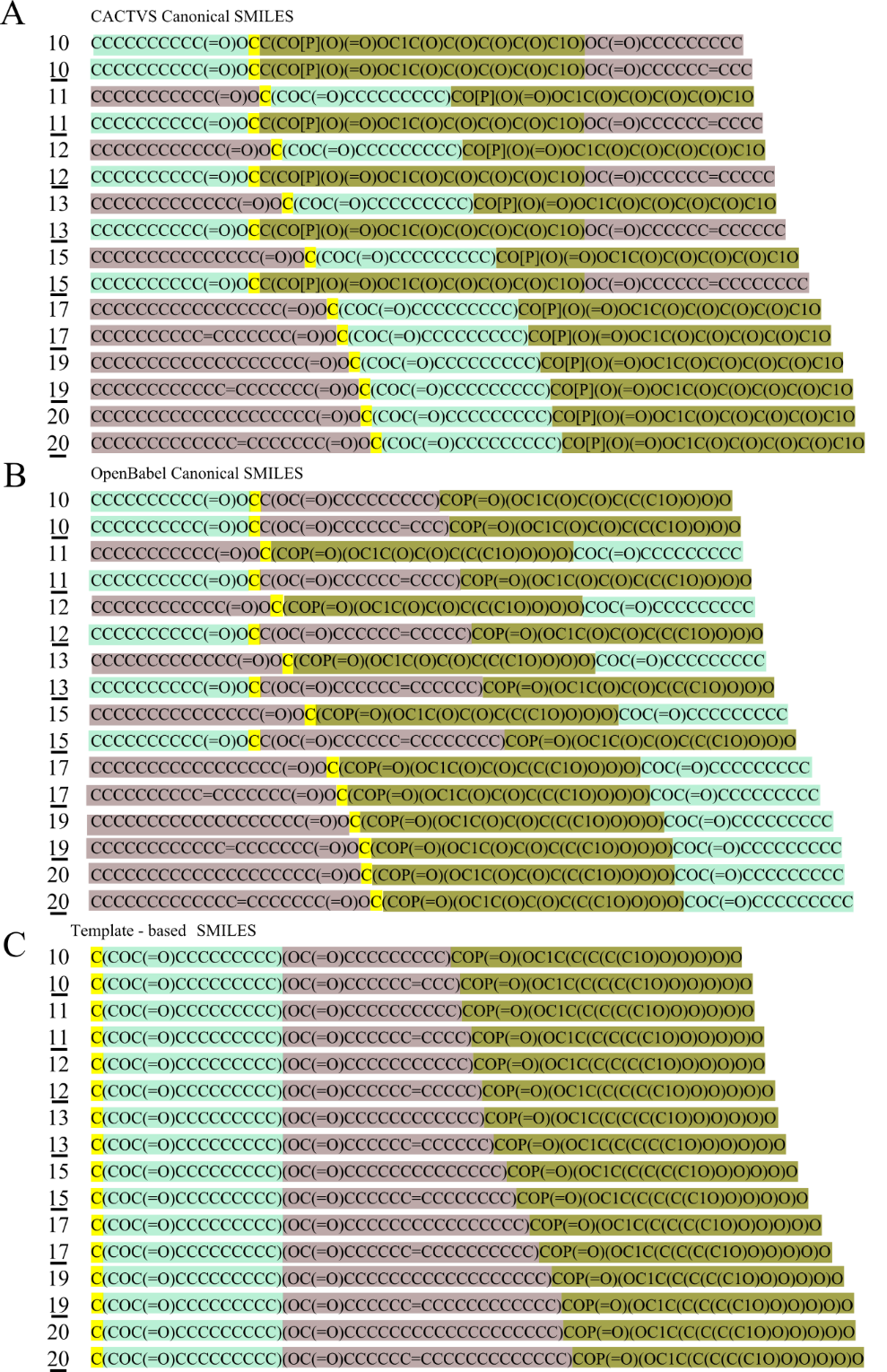


**Figure II – Comparison of SMILES.**

A) CACTVS canonical SMILES, B) OpenBabel canonical SMILES and C) Template-based SMILES of 16 phosphatidylinositols (PI). Structure of first molecule is shown in Figure IIA (below). Color coding of atoms is the same as presented Fig IIA.

References

Ihlenfeldt,W.D. *et al.* (1994) Computation and management of chemical properties in CACTVS: An extensible networked approach toward modularity and compatibility. *J. Chem. Inf. Comput. Sci.*, **34**, 109–116.

Morgan,H.L. (1965) The Generation of a Unique Machine Description for Chemical Structures-A Technique Developed at Chemical Abstracts Service. *J. Chem. Doc.*, **5**, 107–113.

O’Boyle,N.M. (2012) Towards a Universal SMILES representation - A standard method to generate canonical SMILES based on the InChI. *J. Cheminformatics*, **4**, 22.

Sud,M. *et al.* (2012) Template-based combinatorial enumeration of virtual compound libraries for lipids. *J. Cheminformatics*, **4**, 23.
