# Supplementary material for: The LUX Score: A Metric for Lipidome Homology
Source: PLoS Comput Biol. 2015 Sep 22;11(9):e1004511. doi: 10.1371/journal.pcbi.1004511 (PMC4578897; doi:10.1371/journal.pcbi.1004511)
Supplement: S2 Protocol — Includes computer configuration, annotations and abbreviations used in the manuscript. (DOCX) [file pcbi.1004511.s003.docx]

**Supplementary Methods:**

*1.0 SMILES Conversion:*

Chemical table files (SDF, Dalby *et al.*, 1992) were used as input for:

1) OpenBabel’s default SMILES generation algorithm,

2) OpenBabel’s canonical SMILES algorithm (command line option ‘-c’; O’Boyle *et al.*, 2011),

3) CACTVS canonical SMILES tool (Oellien).

*1.1 Structural similarity scoring methods*

Scaled similarity scores were obtained using six methods. 1) OpenBabel FP2 Fingerprint 2) LINGO 3) Bioisosteric similarity 4) SMILIGN 5) Smith Waterman Local Alignment 6) Levenshtein distance.

The distance *d* between a pair of molecules was computed from the similarity score as *d*=1−*S*; where *S* is the similarity score. Range: 0 to 1, ‘0’ – identical, ‘1’ – dissimilar.

1) OpenBabel FP2 Fingerprint (command line option ‘-ofpt’; O’Boyle *et al.*, 2011) is applied on SMILES strings to obtain Tanimoto coefficient. Tanimoto coefficient (S) is converted to distance *d* as described above. Perl script to carryout this procedure is provided as supplementary file, ‘S5_Dataset.zip > Lipid_Similarity_Testing > bin > 111212_m1.pl’.

2) LINGO algorithm as described in the original paper (Vidal *et al.*, 2005) was used to colcualte. Tanimoto coefficient (S). S is converted to distance *d* as described above. Python script for this procedure is provided as supplementary file, ‘S5_Dataset.zip > Lipid_Similarity_Testing > bin > 111206_m2.py’.

3) For the Bioisosteric method, the script ‘query_smiles.pl’ (Krier and Hutter, 2009) was used to calculate the similarity score (S) which is converted to distance *d*. The source code for calculating Bioisosteric similarity score was obtained from Dr. Michael Hutter (personal communication). We cannot share the source code due to license restrictions.

4) SMILIGN is a new method we developed as part of this study. After reducing SMILES character set to 20 symbols, MUSCLE algorithm (Edgar, 2004) was used to calculate alignments based on an identity substitution matrix. The reduction to 20 symbols was achieved by removing the following: a) Positive and negative charges (+,-) b) Sodium symbol (Na) c) atom delimiters ([,]) and d) carbon atom numbers in cyclic structures (1 to 9 numbers). An identity matrix was used as the substitution matrix. Gap opening and gap extension penalties were set to -100. No limit was set to the number of iterations, so alignments were optimized until they converged. The similarity score was calculated as *S*=*n*_mismatch_/*n*_aln_len_; Where *n*_mismatch_ is the number of gaps and *n*_aln_len_ is the length of the alignment. Perl script to carry out this procedure is provided as supplementary file, ‘S5_Dataset.zip > Lipid_Similarity_Testing > bin > 111219_m6.pl’.

5) For the Smith-Waterman alignment method, SMILES were first aligned and the similarity *S* was calculated as the fraction of matching symbols of the longer sequence (*S*=*n_match_*/*n*_longer_). The algorithm by Forrest Bao (open source license) is implemented in this study. Source code is provided as supplementary file, ‘S5_Dataset.zip > Lipid_Similarity_Testing > bin > 111206_m5.py’.

6) The Levenshtein algorithm (Levenshtein, 1966) by Martin Schimmels (open source license) was implemented in this study. Python script is provided in the supplementary file, ‘S5_Dataset.zip > Lipid_Similarity_Testing > bin > 111206_m4.py’.

*1.2 Computer Configuration and Implementation*

Scripts were written for tasks such as 1) Conversion of lipid structures from MDL MOL format to SMILES 2) Calculation of similarity score between a pair of lipids 3) Performing principal component analysis using external packages and libraries 4) Generating plots and interactive lipidome maps and 5) Calculation of lipidome distance. Two software packages are provided at {{www.lux.fz-borstel.de}}. A brief description how to use these programs is provided as a ‘README’ file inside the packages. First package, ‘S5_Dataset.zip > Lipid_Similarity_Testing’ scontains scripts for testing lipid similarity scoring methods on any other dataset of the reader’s choice. Second package, ‘S5_Dataset.zip > Lipidome_Testing.zip’ contains scripts for carrying out lipidome homology analysis. Refer to ‘README’ files and the yeast example inside the package for further details.

Distance calculations for large lipid structure sets (LMSD) were executed on a machine with Intel Xeon processor (2.57 GHz) as single thread and 64GB DDR3 RAM, running on Debian operating system. Smaller datasets (Figure1, Figure2 and Yeast lipidome) were executed on a machine with Intel Xeon processor (3.07 GHz) as single thread and 4GB DDR3 RAM, running on Debian operating system.

*1.3 Annotation of lipid species*

Glycerophospholipids (GPL), DAG and TAG lipids were annotated as: <lipid class> <no. of carbons in all fatty acids> : <no. of double bonds in all fatty acids>. If the sn1 and sn2 position for the fatty acids moieties is known for a GPL, lipids were annotated as: : <lipid class> <no. of carbons of sn1 fatty acid> : <no. of double bonds> / <no. of carbons of sn2 fatty acid> : <no. of double bonds>. Sphingolipids (SP) species were annotated as: <lipid class> <no. of carbons in the long-chain base and fatty acid moieties> : <no. of double bonds in the long-chain base and fatty acid moieties> ; <no. of hydroxyl groups in the long-chain base and fatty acid moieties>. Sterols were annotated as: <ST> <no. of carbons additional to cholesterol> : <no. of double bonds> : <no. of hydroxyl groups additional to the hydroxyl group at position 3>.

*1.3.1 Lipid names*

Cardioplipins (CL), Ceramides (Cer), Diacylglycerol (DAG), Glycosphingolipids (GSL), Inositol phosphorylceramides (IPC), Mannose-bis(inositolphospho)ceramide (M(IP)2C), Phosphatidic acids (PA), Phosphatidylcholines (PC), Phosphatidylethanolamines (PE), Phosphatidylglycerols (PG), Phosphatidylinositols (PI), Phosphatidylserines (PS), Phosphorylethanolamine ceramides (CerPE), Triacylglycerol (TAG),

*1.4 Abbreviations*

Chemical Algorithms Construction, Threading and Verification System (CACTVS), LIPID Metabolites and Pathways Strategy (LIPIDMAPS), LIPIDMAPS Structure Database (LMSD), Structure Data File (SDF), Simplified Molecular Input Line Entry Specification (SMILES), Principal Component Analysis (PCA), Principal Component 1 (PC1), Principal Component 2 (PC2), SMILES Multiple Sequence Alignment (SMILIGN), Lipidome jUXtaposition (LUX) score

*1.5 Principal Component Analysis (PCA)*

Principal Component Analysis was performed using ‘gdata’ library in R (R Core Team, 2014). Principal components were plotted using R ‘scatterplot3d’ module. Interactive plots were generated using ‘RSVGTipsDevice’ package.

*1.5 References*

Dalby,A. *et al.* (1992) Description of several chemical structure file formats used by computer programs developed at Molecular Design Limited. *J. Chem. Inf. Comput. Sci.*, **32**, 244–255.

Edgar,R.C. (2004) MUSCLE: multiple sequence alignment with high accuracy and high throughput. *Nucleic Acids Res.*, **32**, 1792–1797.

Krier,M. and Hutter,M.C. (2009) Bioisosteric similarity of molecules based on structural alignment and observed chemical replacements in drugs. *J. Chem. Inf. Model.*, **49**, 1280–1297.

Levenshtein,V.I. (1966) Binary Codes Capable of Correcting Deletions, Insertions and Reversals. *Sov. Phys. Dokl.*, **10**, 707.

O’Boyle,N.M. *et al.* (2011) Open Babel: An open chemical toolbox. *J. Cheminformatics*, **3**, 33.

Oellien,F. Online SMILES Translator. *Online SMILES Transl.*

R Core Team (2014) R: A Language and Environment for Statistical Computing R Foundation for Statistical Computing, Vienna, Austria.

Vidal,D. *et al.* (2005) LINGO, an efficient holographic text based method to calculate biophysical properties and intermolecular similarities. *J. Chem. Inf. Model.*, **45**, 386–393.
