# Supplementary material for: The LUX Score: A Metric for Lipidome Homology
Source: PLoS Comput Biol. 2015 Sep 22;11(9):e1004511. doi: 10.1371/journal.pcbi.1004511 (PMC4578897; doi:10.1371/journal.pcbi.1004511)
Supplement: S3 Protocol — (DOCX) [file pcbi.1004511.s004.docx]

**S3 Protocol. Compilation of the Drosophila larvae tissue lipidome data**

The lipid composition of six larval tissues of Drosophila (1. Gut, 2. Brain, 3. Wing disc, 4. Salivary Glands, 5. Fat body and 6. Lipoprotein) grown in either yeast food (YF) or plant food (PF) were obtained from Carvalho. et. al. 2012 (Table 1).

Table 1) Overview of all identified lipids in Drosophila larvae tissues (2).

| S. No | Name of the Lipidome Sample | Larval Tissue | Food | No. of Lipids  (Carvalho. et. al.) |
| --- | --- | --- | --- | --- |
| 1 | DmGutYF | Gut | Yeast | 267 |
| 2 | DmGutPF | Gut | Plant | 261 |
| 3 | DmBraiYF | Brain | Yeast | 198 |
| 4 | DmBraiPF | Brain | Plant | 208 |
| 5 | DmWidsYF | Wing disc | Yeast | 209 |
| 6 | DmWidsPF | Wing disc | Plant | 204 |
| 7 | DmSaglYF | Salivary glands | Yeast | 205 |
| 8 | DmSaglPF | Salivary glands | Plant | 196 |
| 9 | DmFaboYF | Fat body | Yeast | 182 |
| 10 | DmFaboPF | Fat body | Plant | 162 |
| 11 | DmLipoYF | Lipoprotein | Yeast | 164 |
| 12 | DmLipoPF | Lipoprotein | Plant | 165 |

All tissue lipidomes combined, resulted in an initial dataset comprising 356 unique lipids. For 288 glycerolipids and glycerophospholipids, structures were generated using the LIPIDMAPS Structure Drawing Tools as explained in Methods section and S2 Protocol. For that, we used known fatty acids of Drosophila (1) and fatty acids that might have been incorporated from the food source or from the larval gut microbiome (3). Accordingly, we made a master list of 29 fatty acids (Tab 2.), which is used as input to computationally generate lipid structures. In this regard, we aimed to explain most of the reported lipids with a minimal set of fatty acids. In this way only 8 lipids (DAG 28:4, PC 38:7, PE 40:7, PE 40:8, PE 40:9, PI 38:7, PS 38:7, TAG 55:8) were omitted containing an unusual high number of double bonds.

For 58 lipids belonging to sterols and sphingolipids, SMILES were manually curated to ensure correct structure selection. Sphingolipids of Drosophila comprise a conserved ceramide structure that contains a long chain bases with chain length of 14 or 16 carbon atoms (4, 5). In Drosophila delta(4,6)-sphingadienes are found, which could not be drawn with the LIPIDMAPS Structure Drawing Tools. We further placed the additional hydroxyl group at the alpha position of the fatty acids as was described earlier (4). With the above assumptions, for Cer 39:1:2, no feasible combination of fatty acid and know sphingosine structure could be assigned.

SMILES for all sterols were derived from cholesterol as basic structure. Here, we omitted one sterol (ST 1:4:0), for which we could not assign a structure. In summary, we incorporated 346 lipids that refers to an overall coverage of 97.2 % for further lipidome analysis. All SMILES are provided within the program package (S5 Dataset).

**Table 2 Putative fatty acids composition for Drosophila lipdiome.**

| No. of Carbon atoms | No. of Double bonds | Position of double bond |
| --- | --- | --- |
| 10 | 0 |  |
| 12 | 0 |  |
| 12 | 1 | (9Z) |
| 13* | 0 |  |
| 13* | 1 | (9Z) |
| 14 | 0 |  |
| 14 | 1 | (9Z) |
| 15* | 0 |  |
| 15* | 1 | (9Z) |
| 16 | 0 |  |
| 16 | 1 | (9Z) |
| 16 | 2 | (9Z,12Z) |
| 17* | 0 |  |
| 17* | 1 | (9Z) |
| 18 | 0 |  |
| 18 | 1 | (9Z) |
| 18 | 2 | (9Z,12Z) |
| 18 | 3 | (9Z,12Z,15Z) |
| 19* | 0 |  |
| 19* | 1 | (9Z) |
| 20 | 1 | (9Z) |
| 20 | 2 | (9Z,12Z) |
| 20 | 3 | (9Z,12Z,15Z) |
| 22 | 1 | (9Z) |
| 22 | 2 | (9Z,12Z) |
| 22 | 3 | (9Z,12Z,15Z) |
| 24 | 1 | (9Z) |
| 24 | 2 | (9Z,12Z) |
| 24 | 3 | (9Z,12Z,15Z) |

^* Fatty acids, which are associated with the food source and microbial contribution.^

**References**

1. Shen LR, Lai CQ, Feng X, Parnell LD, Wan JB, Wang JD, Li D, Ordovas JM, Kang JX. Drosophila lacks C20 and C22 PUFAs. J Lipid Res. 2010 Oct;51(10):2985-92
2. Carvalho M, Sampaio JL, Palm W, Brankatschk M, Eaton S, Shevchenko A. Effects of diet and development on the Drosophila lipidome. Mol Syst Biol. 2012;8:600.
3. Shin SC, Kim SH, You H, Kim B, Kim AC, Lee KA, Yoon JH, Ryu JH, Lee WJ.Drosophila microbiome modulates host developmental and metabolic homeostasis via insulin signaling. Science. 2011;334(6056):670-4.
4. Carvalho M, Schwudke D, Sampaio JL, Palm W, Riezman I, Dey G, Gupta GD, Mayor S, Riezman H, Shevchenko A, Kurzchalia TV, Eaton S. Survival strategies of a sterol auxotroph. Development. 2010 Nov;137(21):3675-85.
5. Fyrst H, Zhang X, Herr DR, Byun HS, Bittman R, Phan VH, Harris GL, Saba JD. Identification and characterization by electrospray mass spectrometry of endogenous Drosophila sphingadienes. J Lipid Res. 2008 Mar;49(3):597-606
