# Supplementary material for: The LUX Score: A Metric for Lipidome Homology
Source: PLoS Comput Biol. 2015 Sep 22;11(9):e1004511. doi: 10.1371/journal.pcbi.1004511 (PMC4578897; doi:10.1371/journal.pcbi.1004511)
Supplement: S4 Dataset — Use the files Yeast_Lipidome_Homology_Scores.htm (Firefox Browser suggested) or Yeast_Lipidome_Homology_Scores.xlsx (Excel 2013) to navigate through the complete yeast lipidome dataset. Plots and additional distance matrices are linked. (ZIP) [file pcbi.1004511.s008.zip › Supplementary_Result_9/Yeast_Lipidome_Homology_Scores.htm]

| two dimensional structural space of the yeat lipidome - PC1, PC2 | | | | | | | | |  | |  | | --- | |  | |  |  |  |  |  |  |  |  |  |  |  |
| BY4741\_24 | 0 |  |  |  |  |  |  |  |  |  |  |  |  |  |  |  |  |  |  |  |  |
| BY4741\_37 | 0.01658 | 0 |  |  |  |  |  |  |  |  |  |  |  |  |  |  |  |  |  |  |  |
| Elo1\_24 | 0.01082 | 0.01534 | 0 |  |  |  |  |  |  |  |  |  |  |  |  |  |  |  |  |  |  |
| Elo1\_37 | 0.01974 | 0.01315 | 0.01458 | 0 |  |  |  |  |  |  |  |  |  |  |  |  |  |  |  |  |  |
| Elo2\_24 | 0.02489 | 0.03188 | 0.03245 | 0.03352 | 0 |  |  |  |  |  |  |  |  |  |  |  |  |  |  |  |  |
| Elo2\_37 | 0.0303 | 0.02764 | 0.03573 | 0.03243 | 0.01025 | 0 |  |  |  |  |  |  |  |  |  |  |  |  |  |  |  |
| Elo3\_24 | 0.03523 | 0.04348 | 0.04247 | 0.04614 | 0.01891 | 0.02241 | 0 |  |  |  |  |  |  |  |  |  |  |  |  |  |  |
| Elo3\_37 | 0.037 | 0.03933 | 0.04068 | 0.04101 | 0.02098 | 0.01715 | 0.01013 | 0 |  |  |  |  |  |  |  |  |  |  |  |  |  |
|  |  |  |  |  |  |  |  |  |  |  |  |  |  |  |  |  |  |  |  |  |  |
| three dimensional structural space of the yeast lipidome - PC1, PC2, PC3 | | | | | | | | |  |  |  |  |  |  |  |  |  |  |  |  |  |
| BY4741\_24 | 0 |  |  |  |  |  |  |  |  |  |  |  |  |  |  |  |  |  |  |  |  |
| BY4741\_37 | 0.02861 | 0 |  |  |  |  |  |  |  |  |  |  |  |  |  |  |  |  |  |  |  |
| Elo1\_24 | 0.01355 | 0.03139 | 0 |  |  |  |  |  |  |  |  |  |  |  |  |  |  |  |  |  |  |
| Elo1\_37 | 0.03433 | 0.01911 | 0.03516 | 0 |  |  |  |  |  |  |  |  |  |  |  |  |  |  |  |  |  |
| Elo2\_24 | 0.04187 | 0.06015 | 0.04806 | 0.06055 | 0 |  |  |  |  |  |  |  |  |  |  |  |  |  |  |  |  |
| Elo2\_37 | 0.04886 | 0.05012 | 0.04996 | 0.0574 | 0.01747 | 0 |  |  |  |  |  |  |  |  |  |  |  |  |  |  |  |
| Elo3\_24 | 0.0565 | 0.07762 | 0.05884 | 0.08052 | 0.03386 | 0.04095 | 0 |  |  |  |  |  |  |  |  |  |  |  |  |  |  |
| Elo3\_37 | 0.05379 | 0.05943 | 0.05064 | 0.06268 | 0.039 | 0.0305 | 0.02398 | 0 |  |  |  |  |  |  |  |  |  |  |  |  |  |
|  |  |  |  |  |  |  |  |  |  |  |  |  |  |  |  |  |  |  |  |  |  |
| |  | | --- | |  | |  |  |  |  |  |  |  |  |  |  |  |  |  |  |  |  |  |  |  |  |  |
|  |  |  |  |  |  |  |  |  |  |  |  |  |  |  |  |  |  |  |  |  |  |
|  |  |  |  |  |  |  |  |  |  |  |  |  |  |  |  |  |  |  |  |  |  |
|  |  |  |  |  |  |  |  |  |  |  |  |  |  |  |  |  |  |  |  |  |  |
|  |  |  |  |  |  |  |  |  |  |  |  |  |  |  |  |  |  |  |  |  |  |
|  |  |  |  |  |  |  |  |  |  |  |  |  |  |  |  |  |  |  |  |  |  |
|  |  |  |  |  |  |  |  |  |  |  |  |  |  |  |  |  |  |  |  |  |  |
|  |  |  |  |  |  |  |  |  |  |  |  |  |  |  |  |  |  |  |  |  |  |
|  |  |  |  |  |  |  |  |  |  |  |  |  |  |  |  |  |  |  |  |  |  |
|  |  |  |  |  |  |  |  |  |  | Dendograms derived by hierachical clustering using two dimensional,� three dimensional structural space and the complete lipid� distance matrix as input for the determination of the pairwise LUX score shown on the right. | | | | | | | | | |  |  |
|  |  |  |  |  |  |  |  |  |  |  |  |
|  |  |  |  |  |  |  |  |  |  |  |  |
|  |  |  |  |  |  |  |  |  |  |  |  |  |  |  |  |  |  |  |  |  |  |
|  |  |  |  |  |  |  |  |  |  |  |  |  |  |  |  |  |  |  |  |  |  |
|  |  |  |  |  |  |  |  |  |  |  |  |  |  |  |  |  |  |  |  |  |  |
|  |  |  |  |  |  |  |  |  |  |  |  |  |  |  |  |  |  |  |  |  |  |
|  |  |  |  |  |  |  |  |  |  |  |  |  |  |  |  |  |  |  |  |  |  |
|  |  |  |  |  |  |  |  |  |  |  |  |  |  |  |  |  |  |  |  |  |  |
|  |  |  |  |  |  |  |  |  |  |  |  |  |  |  |  |  |  |  |  |  |  |
| Distribution of captured variance of the PCA in prinicpal components 1-10. The structural space of lipidome reference map can be depicted in two and three dimensional maps. | | | | | | | | |  |  |  |  |  |  |  |  |  |  |  |  |  |
|  |  |  |  |  |  |  |  |  |  |  |  |  |
|  |  |  |  |  |  |  |  |  |  |  |  |  |
| Yeast reference lipidome distance matrix | | | | | | | | |  |  |  |  |  |  |  |  |  |  |  |  |  |
| BY4741\_24 | 0 |  |  |  |  |  |  |  |  |  |  |  |  |  |  |  |  |  |  |  |  |
| BY4741\_37 | 0.007295 | 0 |  |  |  |  |  |  |  |  |  |  |  |  |  |  |  |  |  |  |  |
| Elo1\_24 | 0.003603 | 0.007192 | 0 |  |  |  |  |  |  |  |  |  |  |  |  |  |  |  |  |  |  |
| Elo1\_37 | 0.007602 | 0.005005 | 0.00768 | 0 |  |  |  |  |  |  |  |  |  |  |  |  |  |  |  |  |  |
| Elo2\_24 | 0.009174 | 0.01284 | 0.01157 | 0.01483 | 0 |  |  |  |  |  |  |  |  |  |  |  |  |  |  |  |  |
| Elo2\_37 | 0.01009 | 0.009856 | 0.01187 | 0.01411 | 0.004691 | 0 |  |  |  |  |  |  |  |  |  |  |  |  |  |  |  |
| Elo3\_24 | 0.01186 | 0.01735 | 0.0143 | 0.01912 | 0.007858 | 0.01033 | 0 |  |  |  |  |  |  |  |  |  |  |  |  |  |  |
| Elo3\_37 | 0.01089 | 0.01261 | 0.01212 | 0.01595 | 0.009331 | 0.007394 | 0.004737 | 0 |  |  |  |  |  |  |  |  |  |  |  |  |  |
|  |  |  |  |  |  |  |  |  |  |  |  |  |  |  |  |  |  |  |  |  |  |
