# Supplementary figures and images for: The LUX Score: A Metric for Lipidome Homology
Source: PLoS Comput Biol. 2015 Sep 22;11(9):e1004511. doi: 10.1371/journal.pcbi.1004511 (PMC4578897; doi:10.1371/journal.pcbi.1004511)

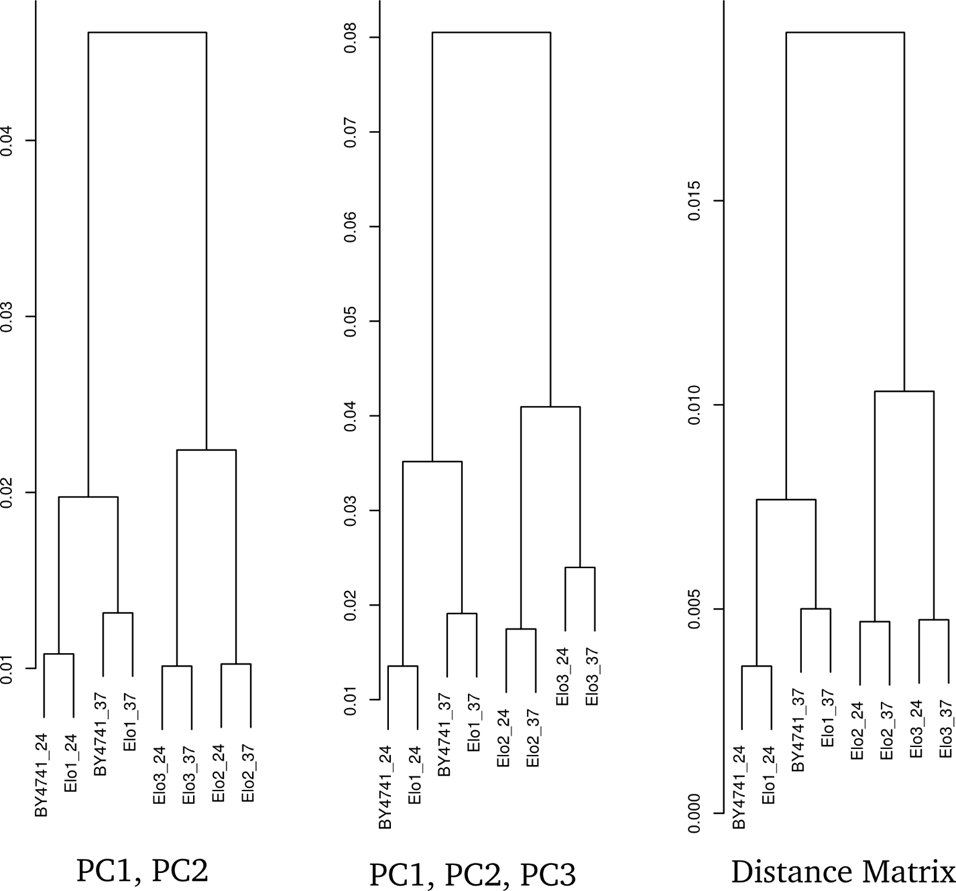

Supplement: S4 Dataset — Use the files Yeast_Lipidome_Homology_Scores.htm (Firefox Browser suggested) or Yeast_Lipidome_Homology_Scores.xlsx (Excel 2013) to navigate through the complete yeast lipidome dataset. Plots and additional distance matrices are linked. (ZIP) [file pcbi.1004511.s008.zip › Supplementary_Result_9/Yeast_Lipidome_Homology_Scores-Dateien/Yeast_Lipidome_Homology_Scores_16667_image001.png]

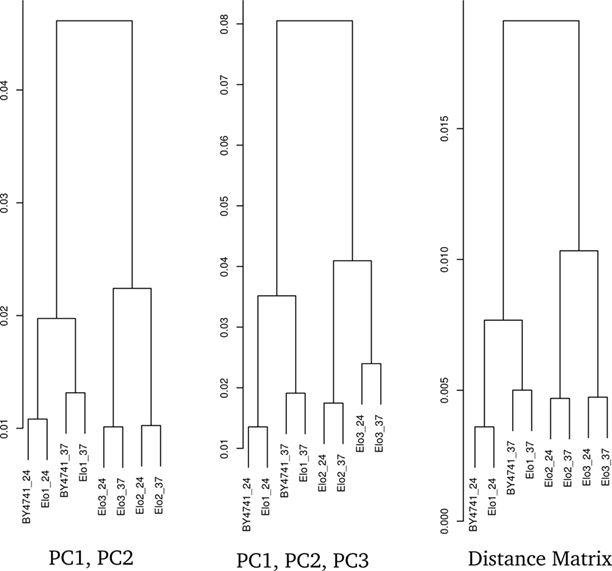

Supplement: S4 Dataset — Use the files Yeast_Lipidome_Homology_Scores.htm (Firefox Browser suggested) or Yeast_Lipidome_Homology_Scores.xlsx (Excel 2013) to navigate through the complete yeast lipidome dataset. Plots and additional distance matrices are linked. (ZIP) [file pcbi.1004511.s008.zip › Supplementary_Result_9/Yeast_Lipidome_Homology_Scores-Dateien/Yeast_Lipidome_Homology_Scores_16667_image002.png]

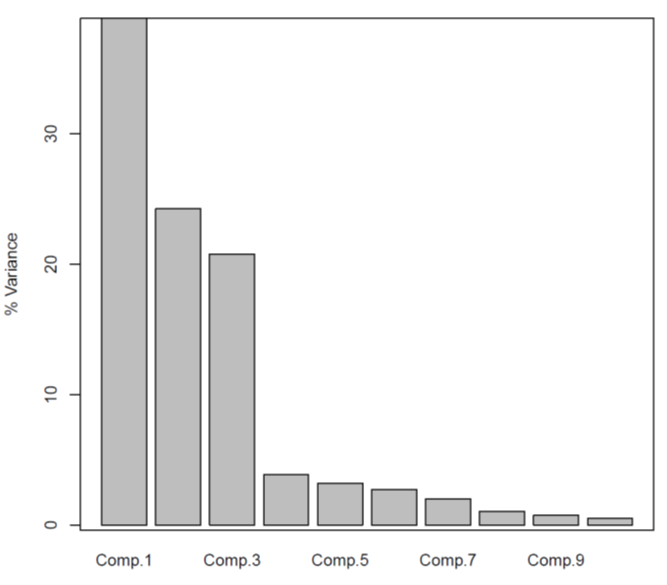

Supplement: S4 Dataset — Use the files Yeast_Lipidome_Homology_Scores.htm (Firefox Browser suggested) or Yeast_Lipidome_Homology_Scores.xlsx (Excel 2013) to navigate through the complete yeast lipidome dataset. Plots and additional distance matrices are linked. (ZIP) [file pcbi.1004511.s008.zip › Supplementary_Result_9/Yeast_Lipidome_Homology_Scores-Dateien/Yeast_Lipidome_Homology_Scores_16667_image003.png]

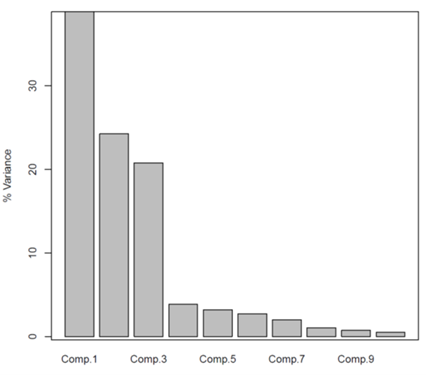

Supplement: S4 Dataset — Use the files Yeast_Lipidome_Homology_Scores.htm (Firefox Browser suggested) or Yeast_Lipidome_Homology_Scores.xlsx (Excel 2013) to navigate through the complete yeast lipidome dataset. Plots and additional distance matrices are linked. (ZIP) [file pcbi.1004511.s008.zip › Supplementary_Result_9/Yeast_Lipidome_Homology_Scores-Dateien/Yeast_Lipidome_Homology_Scores_16667_image004.png]

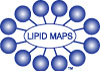

Supplement: S5 Dataset — Includes scripts, README files and data files for Figs 1, 2, 6, 7 and S6. (ZIP) [file pcbi.1004511.s009.zip › S5_Dataset/Lipidome_Homology_Testing/bin/121010_lipidmapstools/docs/html/LipidMAPSLogo.jpg]

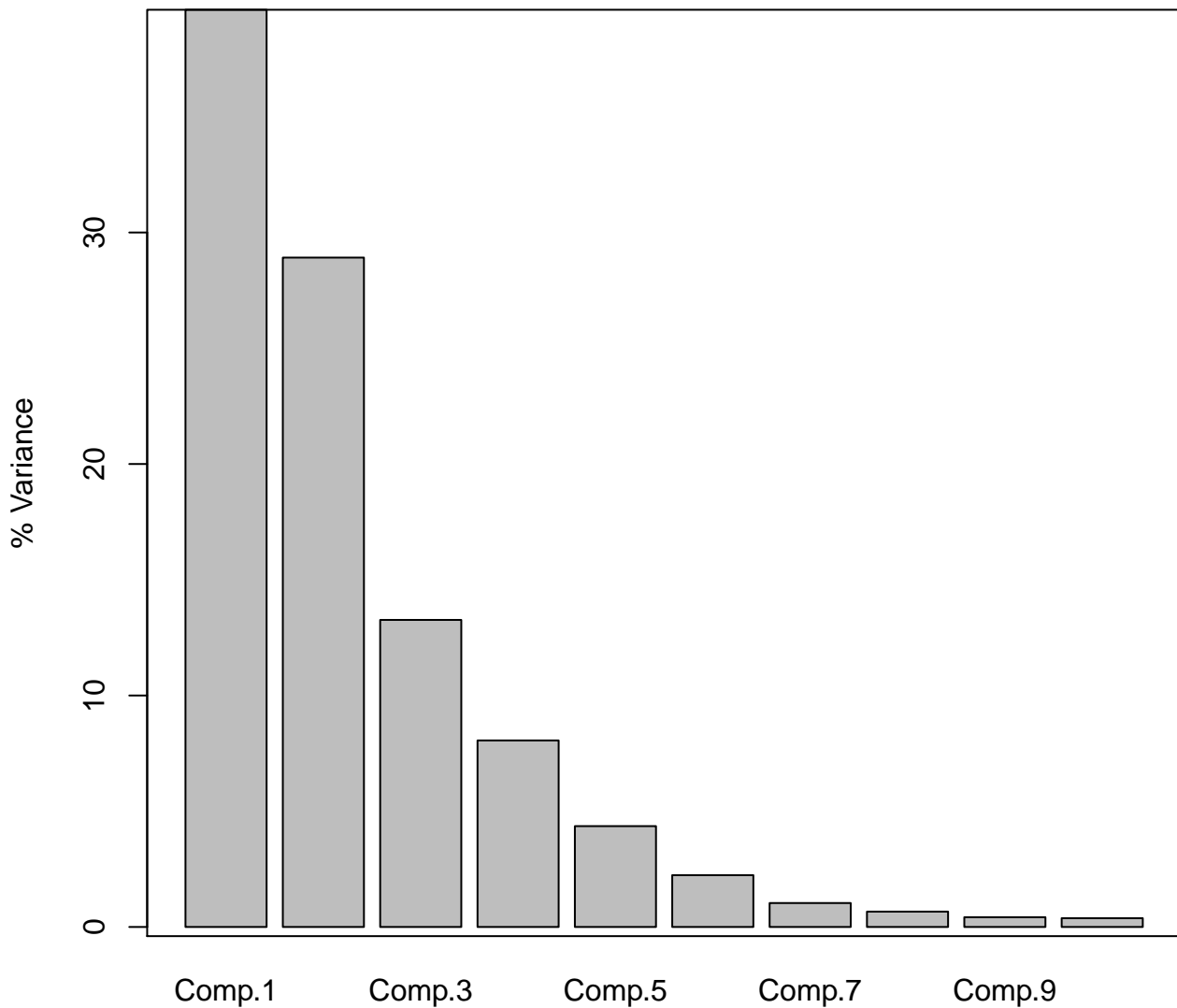

Supplement: S5 Dataset — Includes scripts, README files and data files for Figs 1, 2, 6, 7 and S6. (ZIP) [file pcbi.1004511.s009.zip › S5_Dataset/Lipidome_Homology_Testing/DM_Result/DM_Result_VariancePlot.pdf]

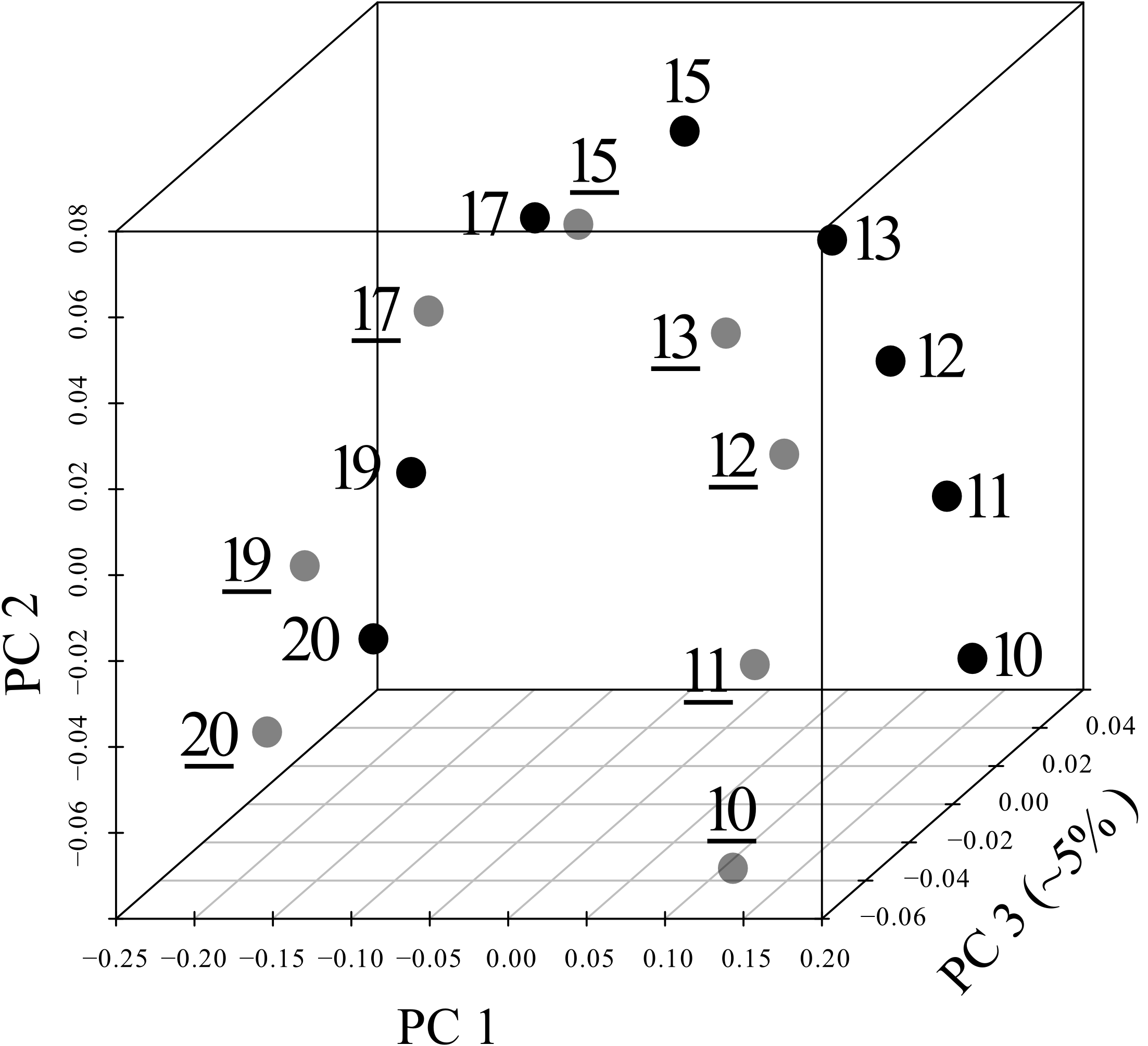

Supplement: S1 Fig — Underlined molecules indicate the presence of the double bond. (TIF) [file pcbi.1004511.s010.tif]

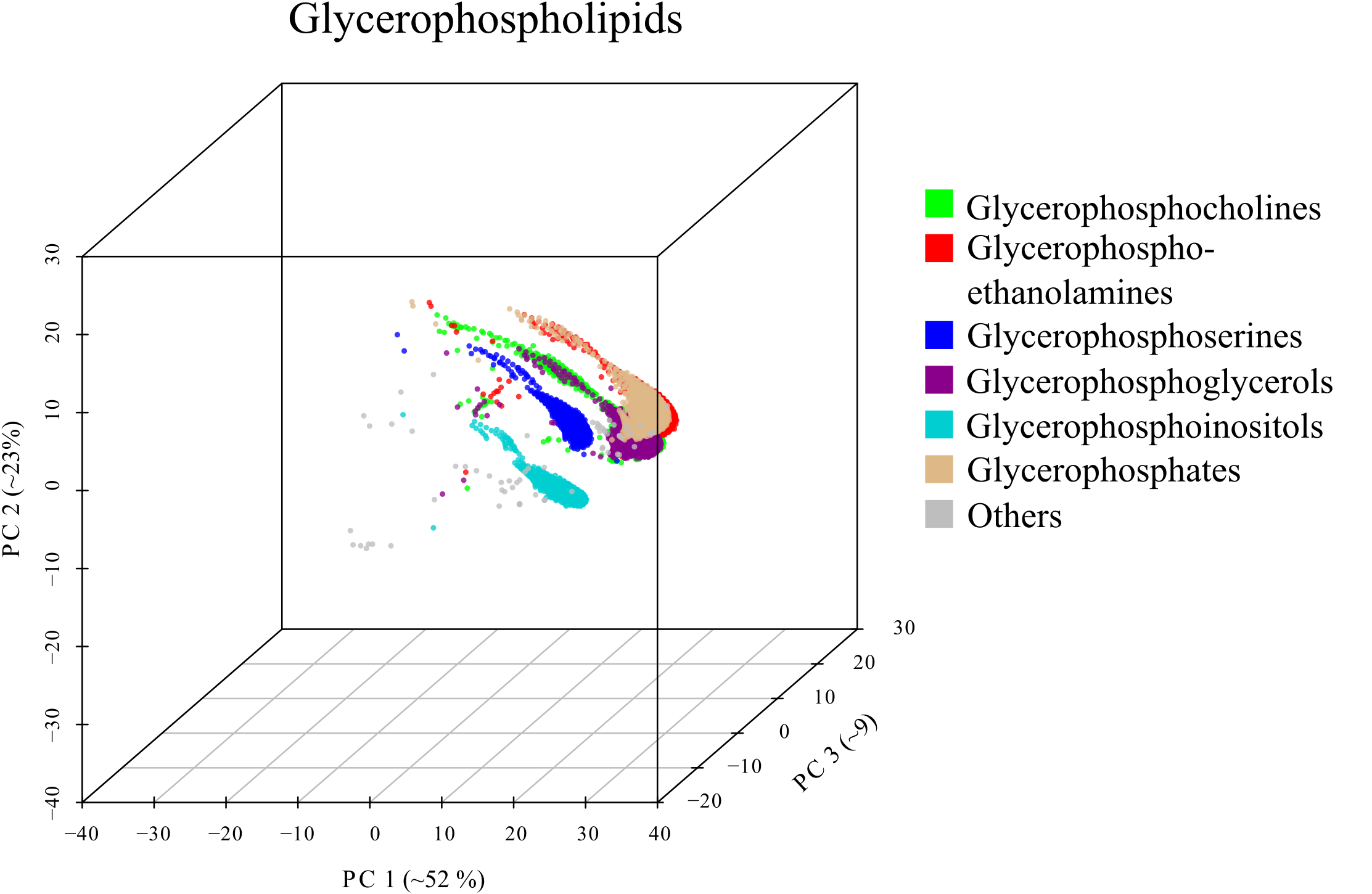

Supplement: S2 Fig — Pairwise distances between 30 150 lipid molecules of LMSD were determined using the Levenshtein method. Only glycerophospholipids are depicted. (TIF) [file pcbi.1004511.s011.tif]

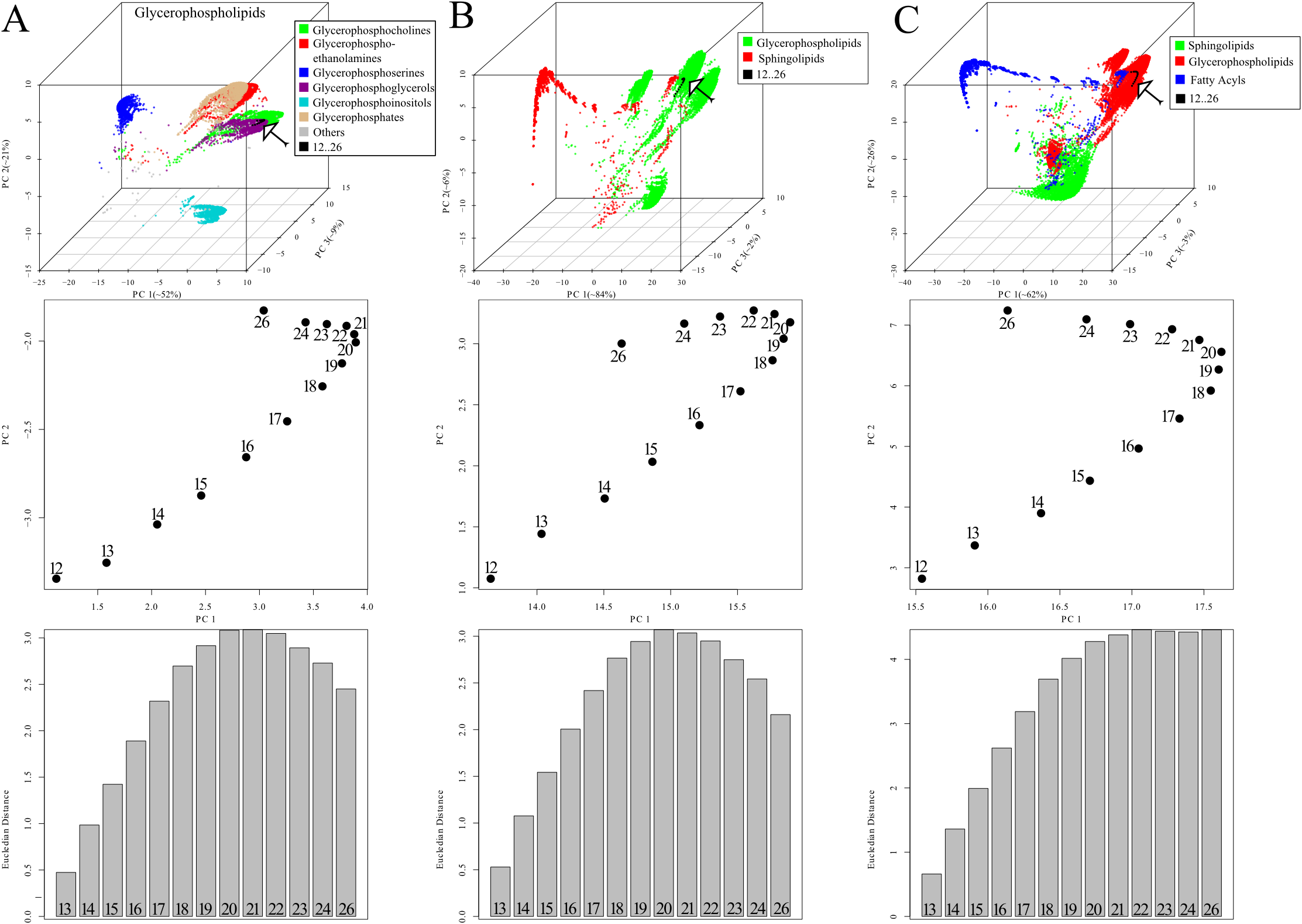

Supplement: S3 Fig — (A) Map of 14 phosphatidylcholine (PC) molecules (Fig 4B) in the background of 7744 Glycerophospholipids. (B) Distribution of the 14 PC molecules in the background of 11 268 Glycerophospholipids and Sphingolipids. (C) Map of 14 PC molecules in the background of 15 304 Glycerophospholipids, Sphingolipids and Fatty acyl molecules. All structures were obtained from LMSD and converted to non-canonical SMILES. Pair wise distances were calculated with Levenshtein method. First row of plots show 14 PC molecules along with indicated lipid structure classes. Second row plots show only 14 PC molecules maintaining the coordinates of corresponding first row plots. Third row of plots show Euclidean distance between first molecule (name 12) and other 13 PCs. Euclidean distance is calculated in Principal Component 1 and Principal Component 2 plane as shown in second row of plots. (TIF) [file pcbi.1004511.s012.tif]

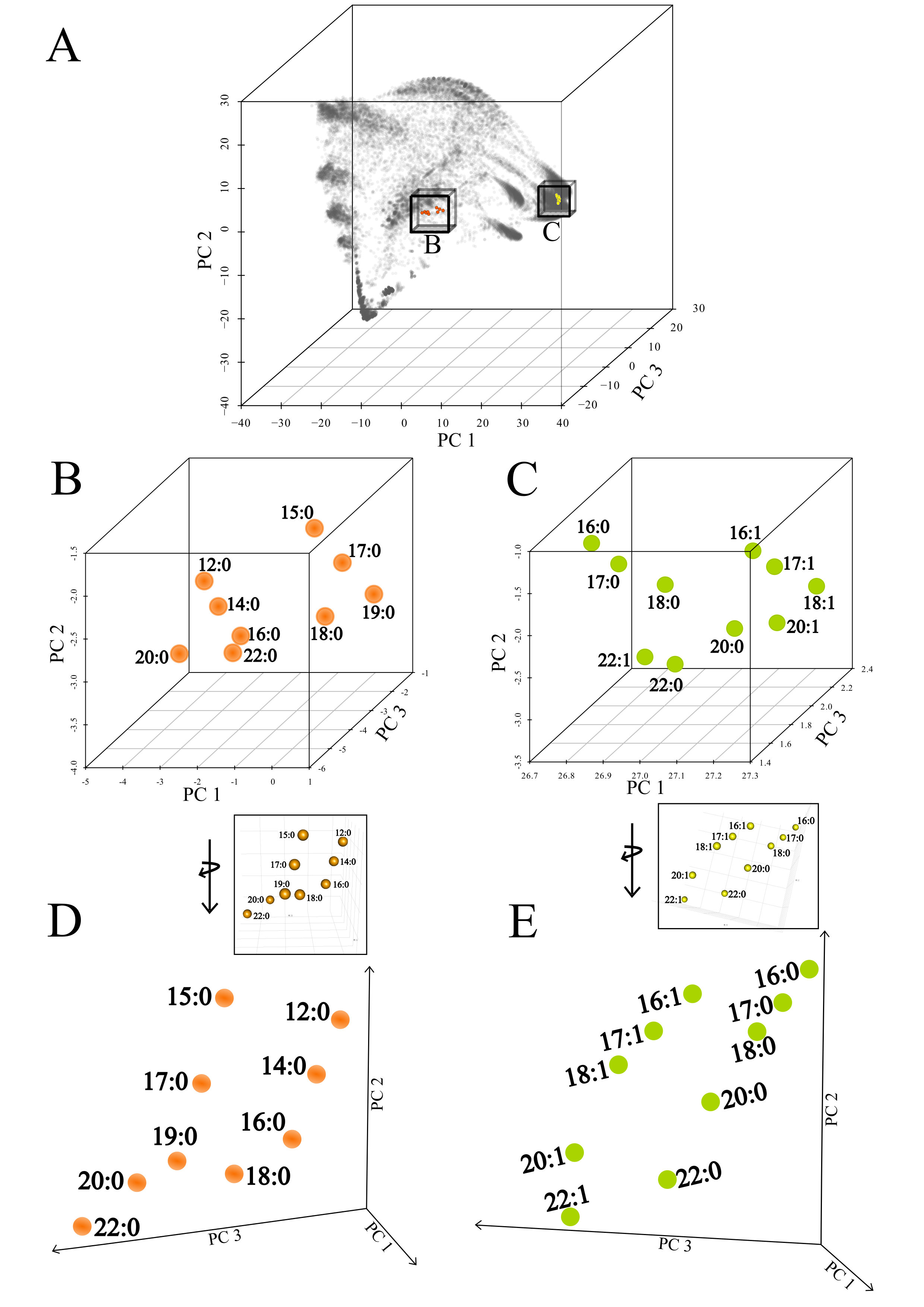

Supplement: S4 Fig — (A) Pairwise distances between 30 150 molecules from LIPIDMAPS Structure Database were determined using Levenshtein distance. Principal Component Analysis was carried out on pairwise distance matrix and the first three components were plotted. Selected triacylglycerols (TAG) and cholesteryl esters (CE) are highlighted while the remaining 30 131 lipids were shown as grey dots. (B) Spatial distribution of 9 CEs differing in the fatty acid chain length (12–22 carbon atoms). Molecule coordinates are the same in Panels A and B. (C). Spatial distribution of 10 TAGs with C16:0 fatty acid in sn1 and sn2 position and third fatty acid chain length varying from 16 to 22 carbon atoms and one double bond. (D) and (E) Manually rendered plots based on the screen-capture of panels B and C respectively (Original screen-captures are shown as inserts). Please note that panels D and E are manually rendered, hence, slight differences in the coordinates might occur. (TIF) [file pcbi.1004511.s013.tif]

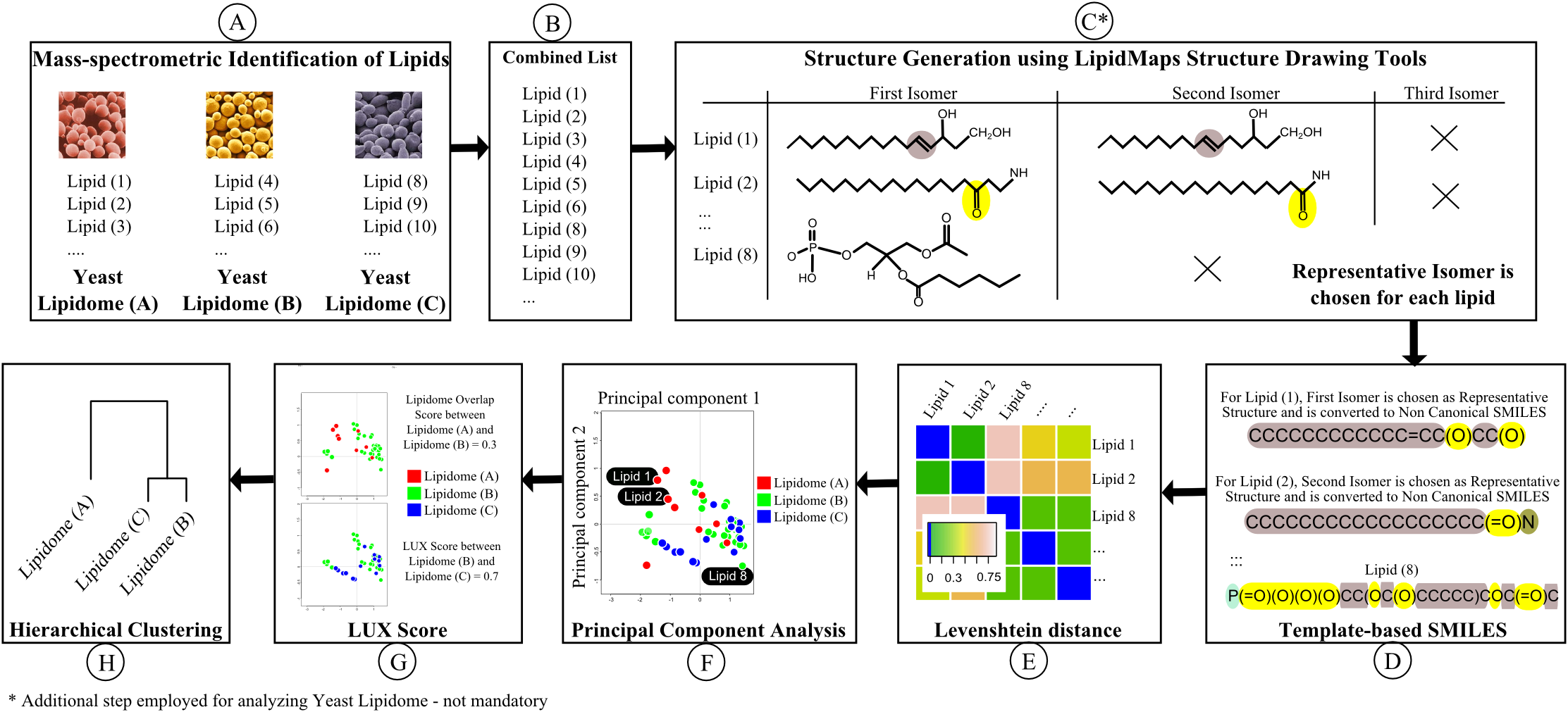

Supplement: S5 Fig — (A) All identified lipid molecular species of a lipidome are combined into a non-redundant list (B). (C) If the lipidomics data is not sufficient to describe the molecular species, all possible isomer structures are inferred from literature and a representative isomer structure is chosen for each lipid. (D) Template-based SMILES are generated for each chosen lipid isomeric structure as the basis for the determination of the structural similarity between all pairs of molecules using Levenshtein distance method (E). (E-F) Pair-wise LUX scores are determined for all lipidome pairs. (G) Hierarchical clustering of LUX scores is performed to depict homology between lipidomes. (TIF) [file pcbi.1004511.s014.tif]
