# Supplementary material for: The LUX Score: A Metric for Lipidome Homology
Source: PLoS Comput Biol. 2015 Sep 22;11(9):e1004511. doi: 10.1371/journal.pcbi.1004511 (PMC4578897; doi:10.1371/journal.pcbi.1004511)
Supplement: S5 Dataset — Includes scripts, README files and data files for Figs 1, 2, 6, 7 and S6. (ZIP) [file pcbi.1004511.s009.zip › S5_Dataset/Lipidome_Homology_Testing/bin/121010_lipidmapstools/docs/html/ChainAbbrev.html]

LIPID MAPS Tools Documentation: ChainAbbrev.pm


|  |  |
| --- | --- |
|  | LIPID Metabolites And Pathways Strategy |

  

|  |
| --- |
| PDF  PDFA4 |

## NAME

ChainAbbrev - Methods for processing chain abbreviations

## SYNOPSIS

use ChainAbbrev;

use ChainAbbrev qw(:all);

## DESCRIPTION

ChainAbbrev module provides these methods:

ChainAbbrevNameExists - Is it a supported chain abbreviation
  
 ExpandChainAbbrev - Expand wild cards in chain abbreviation
  
 GetChainAbbrevToNameMap - Get chain name
  
 GetChainLenAbbrevSupportedMap - Get reference to supported chain
abbreviations data
  
 GetChainLenAbbrevDbleBondGeometryDataMap - Get reference to supported
double bond geometry data
  
 GetChainLengthAndMultipleBondCount - Get chain length and number of
double and triple bonds
  
 GetChainLenToNamePrefixMap - Get chain name prefix
  
 GetCountToNamePrefixMap - Get count prefix
  
 GetSubstituentsAbbrevToNameMap - Get substituents name
  
 GetSubstituentBondOrder - Get substituent bond order
  
 GetSupportedChainLenList - Get supported chain lengths
  
 IsAlkylChainAbbrev - Is it a alkyl chain abbreviation
  
 IsAlkenylChainAbbrev - Is it a alkenyl chain abbreviation
  
 IsChainAbbrevOkay - Is it a valid chain abbreviation
  
 IsDoubleBondsAbbrevOkay - Is it a valid double bond abbreviation
  
 IsRingsAbbrevOkay - Is it a valid ring abbreviation
  
 IsSubstituentsAbbrevOkay - Is it a valid substituent abbreviation
  
 IsWildCardInChainAbbrev - Does chain abbreviation contains a wild card
  
 ParseChainAbbrev - Parse chain abbreviation
  
 ParseRingAbbrev - Parse ring abbreviation
  
 ParseSubstituentAbbrev - Parse substituent abbreviation
  
 SetupChainSubstituentsName - Set up substituent name

## METHODS

**ChainAbbrevNameExists**
:   $Status = ChainAbbrevNameExists($ChainAbbrev);

    Return 1 or 0 based on whether it's a supported chain name.

**ExpandChainAbbrev**
:   $AbbrevArrayRef = ExpandChainAbbrev($Abbrev);

    Return a reference to an array containing complete chain abbreviations. Wild card
    characters in chain abbreviation name are expanded to generate fully qualified
    chain abbreviations.

**GetChainAbbrevToNameMap**
:   $AbbrevNameHashRef = GetChainAbbrevToNameMap();

    Return a reference to hash with chain abbreviation/name as key/value pair.

**GetChainLenAbbrevSupportedMap**
:   $ChainLenHashRef = GetChainLenAbbrevSupportedMap();

    Return a reference to hash with supported chain length as hash key.

**GetChainLenAbbrevDbleBondGeometryDataMap**
:   $ChainLenDblBondHashRef =
    GetChainLenAbbrevDbleBondGeometryDataMap();

    Return a reference to hash containing information about chain length, number of
    double bonds and geometry of double bonds.

**GetChainLengthAndMultipleBondCount**
:   ($ChainLength, $DoubleBondCount, $TripleBondCount) =
    GetChainLengthAndMultipleBondCount($ChainAbbrev);

    Parse chain abbreviation and return these values: chain length; number of
    double and triple bonds.

**GetChainLenToNamePrefixMap**
:   $ChainNameHashRef = GetChainLenToNamePrefixMap();

    Return a reference to hash with chain length/name prefix as key/value pair.

**GetCountToNamePrefixMap**
:   $CountHashRef = GetCountToNamePrefixMap();

    Return a reference to hash with count/name prefix as key/value pair.

**GetSubstituentsAbbrevToNameMap**
:   $AbbrevNameHashRef = GetSubstituentsAbbrevToNameMap();

    Return a reference to hash with substituents abbreviation/name as key/value pair.

**GetSubstituentBondOrder**
:   $BondOrder = GetSubstituentBondOrder($SubstituentAbbrev);

    Return bond order for a sustituent.

**GetSupportedChainLenList**
:   $ChainLengthListRef = GetSupportedChainLenList();

    Return a reference to a sorted list containing supported chain lengths.

**IsAlkylChainAbbrev**
:   $Status = IsAlkylChainAbbrev($ChainAbbrev);

    Return 1 or 0 based on whether it's a alkyl chain abbreviation.

**IsAlkenylChainAbbrev**
:   $Status = IsAlkenylChainAbbrev($ChainAbbrev);

    Return 1 or 0 based on whether it's a alkenyl chain abbreviation.

**IsChainAbbrevOkay**
:   $Status = IsChainAbbrevOkay($ChainAbbrev);

    Return 1 or 0 based on whether chain abbreviation is valid.

**IsDoubleBondsAbbrevOkay**
:   $Status = IsDoubleBondsAbbrevOkay($ChainAbbrev, $ChainLength,
    $DoubleBondCount, $DoubleBondGeometry);

    Return 1 or 0 based on whether chain abbreviation contains a valid multiple bond specification.

**IsRingsAbbrevOkay**
:   $Status = IsRingsAbbrevOkay($ChainAbbrev, $ChainLength, $Rings);

    Return 1 or 0 based on whether chain abbreviation contains a valid ring specification.

**IsSubstituentsAbbrevOkay**
:   $Status = IsSubstituentsAbbrevOkay($ChainAbbrev, $ChainLength,
    $DoubleBondCount, $DoubleBondGeometry, $Substituents);

    Return 1 or 0 based on whether chain abbreviation contains a valid substituents specification.

**IsWildCardInChainAbbrev**
:   $Status = IsWildCardInChainAbbrev($ChainAbbrev);

    Return 1 or 0 based on whether chain abbreviation contains any wild card character.

**ParseChainAbbrev**
:   ($ChainLength, $DoubleBondCount, $DoubleBondGeometry) =
    ParseChainAbbrev($ChainAbbrev);

    Parse chain abbreviation and return these values: chain length, number of double bonds,
    and geometry of double bonds.

**ParseRingAbbrev**
:   ($Pos, $StereoChemistry) = ParseRingAbbrev($ChainAbbrev);

    Parse chain abbreviation and return these values: ring position and stereochemistry
    specificaton at the ring.

**ParseSubstituentAbbrev**
:   ($Pos, $Name, $StereoChemistry) =
    ParseSubstituentAbbrev($SubstituentAbbrev);

    Parse substituent abbreviation and return these values: position of the substituent
    on the chain, name, and stereochemistry of the substituent.

**SetupChainSubstituentsName**
:   $SubstituentsName = SetupChainSubstituentsName(
    $CmpdAbbrevTemplateDataMapRef, $ChainIndex);

    Return systematic name for substituents after ordering and grouping substituents by their
    position.

## AUTHOR

Manish Sud

## CONTRIBUTOR

Eoin Fahy

## SEE ALSO

LMAPSStr.pm, ChainStr.pm

## COPYRIGHT

Copyright (C) 2006-2012. The Regents of the University of California. All Rights Reserved.

## LICENSE

Modified BSD License
