# Supplementary material for: The LUX Score: A Metric for Lipidome Homology
Source: PLoS Comput Biol. 2015 Sep 22;11(9):e1004511. doi: 10.1371/journal.pcbi.1004511 (PMC4578897; doi:10.1371/journal.pcbi.1004511)
Supplement: S5 Dataset — Includes scripts, README files and data files for Figs 1, 2, 6, 7 and S6. (ZIP) [file pcbi.1004511.s009.zip › S5_Dataset/Lipidome_Homology_Testing/bin/121010_lipidmapstools/docs/html/ChainStr.html]

LIPID MAPS Tools Documentation: ChainStr.pm


|  |  |
| --- | --- |
|  | LIPID Metabolites And Pathways Strategy |

  

|  |
| --- |
| PDF  PDFA4 |

## NAME

ChainStr - Fatty Acyls (FA), Glycerolipids (GL) and Glycerophospholipids (GP) structure data generation methods

## SYNOPSIS

use ChainStr;

use ChainStr qw(:all);

## DESCRIPTION

ChainStr module provides these methods:

AssignSubstituentStereoChemistry - Assign stereochemistry to substituents
  
 GenerateAtomBlockLines - Generate SD file atom data lines
  
 GenerateBondBlockLines - Generate SD file bond data lines
  
 GenerateChainStrData - Generate structure data for chains
  
 GenerateCmpdCountsLine - Generate SD file count data line
  
 IsAnySubstituentSpecifiedWithStereoChemistry - Check stereochemistry of
substituents
  
 SetupTemplateDataMap - Set up template data for a compound abbreviation

## METHODS

**AssignSubstituentStereoChemistry**
:   AssignSubstituentStereoChemistry($CmpdAbbrevTemplateDataMapRef,
    $CmdDataLinesRef);

    Assign stereochemistry to substituents using structure and stereochemistry data available via
    $CmpdAbbrevTemplateDataMapRef and $CmdDataLinesRef. And add new lines to existing
    structure data using $CmdDataLinesRef.

**GenerateAtomBlockLines**
:   $AtomDataLines = GenerateAtomBlockLines($CmpdAbbrevTemplateDataMapRef,
    $Sn1AtomLinesRef, $Sn2AtomLinesRef, $Sn3AtomLinesRef);

    Return atom data lines suitable for writing to SD file. Atom data for all approrpriate chains is
    merged into a single string using new line character as delimiter.

**GenerateBondBlockLines**
:   $BondDataLines = GenerateBondBlockLines($CmpdAbbrevTemplateDataMapRef,
    $Sn1BondLinesRef, $Sn2AtomLinesRef, $Sn3AtomLinesRef);

    Return bond data lines suitable for writing to SD file. Bond data for all approrpriate chains is
    merged into a single string using new line character as delimiter.

**GenerateChainStrData**
:   ($AtomLinesArrayRef, $BondLinesArrayRef) = GenerateChainStrData($ChainType,
    $CmpdAbbrevTemplateDataMapRef);

    Return array references containing atom and bond data lines for SD file. Appropriate atom
    and bond data lines are generated using chain type and abbreviation template data.

**GenerateCmpdCountsLine**
:   $RetValue = GenerateCmpdCountsLine($CmpdAbbrevTemplateDataMapRef);

    Return a formatted count data line for SD file.

**IsAnySubstituentSpecifiedWithStereoChemistry**
:   $Status = IsAnySubstituentSpecifiedWithStereoChemistry(
    $CmpdAbbrevTemplateDataMapRef);

    Return 1 or 0 based on whether stereochemistry is specifed for any substituent.

**SetupTemplateDataMap**
:   SetupTemplateDataMap($TemplateType, $AbbrevTemplateDataMapRef,
    $TemplateData);

    Setup compound abbreviation template data using a supported template.

## AUTHOR

Manish Sud

## CONTRIBUTOR

Eoin Fahy

## SEE ALSO

FAStr.pm, GLStr.pm, GPStr.pm, LMAPSStr.pm, SPStr.pm

## COPYRIGHT

Copyright (C) 2006-2012. The Regents of the University of California. All Rights Reserved.

## LICENSE

Modified BSD License
