# Supplementary material for: The LUX Score: A Metric for Lipidome Homology
Source: PLoS Comput Biol. 2015 Sep 22;11(9):e1004511. doi: 10.1371/journal.pcbi.1004511 (PMC4578897; doi:10.1371/journal.pcbi.1004511)
Supplement: S5 Dataset — Includes scripts, README files and data files for Figs 1, 2, 6, 7 and S6. (ZIP) [file pcbi.1004511.s009.zip › S5_Dataset/Lipidome_Homology_Testing/bin/121010_lipidmapstools/docs/html/CLStrGen.html]

LIPID MAPS Tools Documentation: CLStrGen.pl


|  |  |
| --- | --- |
|  | LIPID Metabolites And Pathways Strategy |

  

|  |
| --- |
| PDF  PDFA4 |

## NAME

CLStrGen.pl - Generate structures for Glycerophosphoglycerophosphoglycerols (Cardiolipins)

## SYNOPSIS

CLStrGen.pl CLAbbrev|CLAbbrevFileName ...

CLStrGen.pl [**-c, --ChainAbbrevMode** *MostLikely | Arbitrary*]
[**-h, --help**] [**-m, --mode** *Abbrev | AbbrevFileName*]
[**-p, --ProcessMode** *WriteSDFile | CountOnly*] [**-o, --overwrite**]
[**-r, --root** rootname] [**-w, --workingdir** dirname] <arguments>...

## DESCRIPTION

Generate Cardiolipins (CL) structures using compound abbreviations specified on
a command line or in a CSV/TSV Text file. All the command line arguments represent either
compound abbreviations or file name containing abbreviations. Use mode option to control
the type of command line arguments.

A SD file, containing structures for all CL abbreviations along with ontological information, is
generated as an output.

## SUPPORTED ABBREVIATIONS

Current support for CL structure generation include these main classes and sub classes:

o Glycerophosphoglycerophosphoglycerols (Cardiolipins)

. Diacylglycerophosphoglycerophosphodiradylglycerols
  
 . Diacylglycerophosphoglycerophosphomonoradylglycerols
  
 . 1-alkyl,2-acylglycerophosphoglycerophosphodiradylglycerols
  
 . 1-alkyl,2-acylglycerophosphoglycerophosphomonoradylglycerols
  
 . 1Z-alkenyl,2-acylglycerophosphoglycerophosphodiradylglycerols
  
 . 1Z-alkenyl,2-acylglycerophosphoglycerophosphomonoradylglycerols
  
 . Monoacylglycerophosphoglycerophosphomonoradylglycerols
  
 . 1-alkyl glycerophosphoglycerophosphodiradylglycerols
  
 . 1-alkyl glycerophosphoglycerophosphomonoradylglycerols
  
 . 1Z-alkenylglycerophosphoglycerophosphodiradylglycerols
  
 . 1Z-alkenylglycerophosphoglycerophosphomonoradylglycerols

## OPTIONS

**-c, --ChainAbbrevMode** *MostLikely|Arbitrary*
:   Specify what types of acyl chain abbreviations are allowed during processing of complete
    abbreviations: allow most likely chain abbreviations containing specific double bond geometry
    specifications; allow any acyl chain abbreviation with valid chain length and double bond
    geometry specificatios. Possible values: *MostLikely or Arbitrary*. Default value: *MostLikely*.

    *Arbitrary* value of **-c, --ChainAbbrevMode** option is not allowed during processing of
    abbreviations containing wild cards.

    During *MostLikely* value of **-c, --ChainAbbrevMode** option, only the most likely acyl chain
    abbreviations specified in ChainAbbrev.pm module are allowed. However, during *Arbitrary* value
    of **-c, --ChainAbbrevMode** option, any acyl chain abbreviations with valid chain length and
    double bond geometry can be specified. The current release of lipidmapstools support chain
    lengths from 2 to 50 as specified in ChainAbbev.pm module.

    In addition to double bond geometry specifications, valid substituents can be specified for in the acyl
    chain abbreviations.

**-h, --help**
:   Print this help message

**-m, --mode** *Abbrev|AbbrevFileName*
:   Controls interpretation of command line arguments. Two different methods are provided:
    specify compound abbreviations or a file name containing compound abbreviations. Possible
    values: *Abbrev or AbbrevFileName*. Default: *Abbrev*

    In *AbbrevFileName* mode, a single line in CSV/TSV files can contain multiple compound
    abbreviations. The file extension determines delimiter used to process data lines: comma for
    CSV and tab for TSV. For files with TXT extension, only one compound abbreviation per line
    is allowed.

    Wild card character, \*, is also supported in compound abbreviations.

    Examples:

    Specific structures: CL(1'-[18:2(9Z,12Z)/18:2(9Z,12Z)],
    3'-[18:2(9Z,12Z)/18:2(9Z,12Z)])
      
     All possibilites: \*(1'-[\*:\*/\*:\*],3'-[\*:\*/\*:\*]) or
    \*(1'-[\*/\*],3'-[\*/\*])

    With wild card character, +/- can also be used for chain lengths to indicate even and odd lengths at
    sn1/sn2/sn3 positions; additionally > and < qualifiers are also allowed to specify length
    requirements. Examples:

    Odd/even number chains at sn1/sn3 and sn2/sn4: \*(1'-[\*+:\*/\*-:\*],
    3'-[\*+:\*/\*-:\*])
      
     Odd/even number chains at sn1/sn3 and sn2/sn4 with length longer
      
     than 20 and 22: \*(1'-[\*+>20:\*/\*->22:\*],3'-[\*+>20:\*/\*->22:\*])

**-p, --ProcessMode** *WriteSDFile|CountOnly*
:   Specify how abbreviations are processed: generate structures for specified abbreviations along
    with generating a SD file or just count the number of structures corresponding to specified
    abbreviations without generating any SD file. Possible values: *WriteSDFile or CountOnly*.
    Default: *WriteSDFile*.

    It can take substantial amount of time for generating all the structures and writing out a SD file
    for abbreviations containing wild cards. *CountOnly* value of **--ProcessMode** option can
    be used to get a quick count of number of structures to be generated without writing out any
    SD file.

**-o, --overwrite**
:   Overwrite existing files

**-r, --root** *rootname*
:   New file name is generated using the root: <Root>.sdf. Default for new file names: CLAbbrev.sdf,
    <AbbrevFilenName>.sdf, or <FirstAbbrevFileName>1To<Count>.sdf.

**-w, --workingdir** *dirname*
:   Location of working directory. Default: current directory

## EXAMPLES

On some systems, command line scripts may need to be invoked using
*perl -s GLStrGen.pl*; however, all the examples assume direct invocation
of command line script works.

To generate a CLStructures.sdf file containing a structure specified by a command line
CL abbreviation for Diacylglycerophosphoglycerophosphodiradylglycerols, type:

% CLStrGen.pl -r CLStructures -o "CL(1'-[18:2(9Z,12Z)/18:2(9Z,12Z)],
3'-[18:2(9Z,12Z)/18:2(9Z,12Z)])"

To generate a CLStructures.sdf file containing a structure specified by a command line
CL abbreviation for Diacylglycerophosphoglycerophosphomonoradylglycerols, type:

% CLStrGen.pl -r CLStructures -o "CL(1'-[18:2(9Z,12Z)/18:2(9Z,12Z)],
3'-[18:2(9Z,12Z)/0:0])"

To generate a CLStructures.sdf file containing a structure specified by a command line
CL abbreviation for 1-alkyl,2-acylglycerophosphoglycerophosphodiradylglycerols, type:

% CLStrGen.pl -r CLStructures -o "CL(1'-[O-16:0/18:2(9Z,12Z)],
3'-[18:2(9Z,12Z)/18:2(9Z,12Z)])"

To generate a CLStructures.sdf file containing a structure specified by a command line
CL abbreviation for 1-alkyl,2-acylglycerophosphoglycerophosphomonoradylglycerols, type:

% CLStrGen.pl -r CLStructures -o "CL(1'-[O-16:0/18:2(9Z,12Z)],
3'-[18:2(9Z,12Z)/0:0])"

To generate a CLStructures.sdf file containing a structure specified by a command line
CL abbreviation for 1Z-alkenyl,2-acylglycerophosphoglycerophosphodiradylglycerols, type:

% CLStrGen.pl -r CLStructures -o "CL(1'-[P-16:0/18:2(9Z,12Z)],
3'-[18:2(9Z,12Z)/18:2(9Z,12Z)])"

To generate a CLStructures.sdf file containing a structure specified by a command line
CL abbreviation for 1Z-alkenyl,2-acylglycerophosphoglycerophosphomonoradylglycerols, type:

% CLStrGen.pl -r CLStructures -o "CL(1'-[P-16:0/18:2(9Z,12Z)],
3'-[18:2(9Z,12Z)/0:0])"

To generate a CLStructures.sdf file containing a structure specified by a command line
CL abbreviation for Monoacylglycerophosphoglycerophosphomonoradylglycerols, type:

% CLStrGen.pl -r CLStructures -o "CL(1'-[18:2(9Z,12Z)/0:0],
3'-[18:2(9Z,12Z)/0:0])"

To generate a CLStructures.sdf file containing a structure specified by a command line
CL abbreviation for 1-alkyl glycerophosphoglycerophosphodiradylglycerols, type:

% CLStrGen.pl -r CLStructures -o "CL(1'-[O-16:0/0:0],
3'-[18:2(9Z,12Z)/18:2(9Z,12Z)])"

To generate a CLStructures.sdf file containing a structure specified by a command line
CL abbreviation for 1-alkyl glycerophosphoglycerophosphomonoradylglycerols, type:

% CLStrGen.pl -r CLStructures -o "CL(1'-[O-16:0/0:0],
3'-[18:2(9Z,12Z)/0:0])"

To generate a CLStructures.sdf file containing a structure specified by a command line
CL abbreviation for 1Z-alkenylglycerophosphoglycerophosphodiradylglycerols, type:

% CLStrGen.pl -r CLStructures -o "CL(1'-[P-16:0/0:0],
3'-[18:2(9Z,12Z)/18:2(9Z,12Z)])"

To generate a CLStructures.sdf file containing a structure specified by a command line
CL abbreviation for 1Z-alkenylglycerophosphoglycerophosphomonoradylglycerols, type:

% CLStrGen.pl -r CLStructures -o "CL(1'-[P-16:0/0:0],
3'-[18:2(9Z,12Z)/0:0])"

To enumerate all possible CL structures and generate a CLStructures.sdf
file, type:

% CLStrGen.pl -r CLStructures -o "\*(1'-[\*/\*],3'-[\*/\*])"

or

% CLStrGen.pl -r CLStructures -o "\*(1'-[\*:\*/\*:\*],3'-[\*:\*/\*:\*])"

or

% CLStrGen.pl -r CLStructures -o "\*(1'-[\*:\*(\*)/\*:\*(\*)],
3'-[\*:\*(\*)/\*:\*(\*)])"

## AUTHOR

Manish Sud

## CONTRIBUTOR

Eoin Fahy

## SEE ALSO

FAStrGen.pl, GLStrGen.pl, GPStrGen.pl, SPStrGen.pl, STStrGen.pl

## COPYRIGHT

Copyright (C) 2006-2012. The Regents of the University of California. All Rights Reserved.

## LICENSE

Modified BSD License
