# Supplementary material for: The LUX Score: A Metric for Lipidome Homology
Source: PLoS Comput Biol. 2015 Sep 22;11(9):e1004511. doi: 10.1371/journal.pcbi.1004511 (PMC4578897; doi:10.1371/journal.pcbi.1004511)
Supplement: S5 Dataset — Includes scripts, README files and data files for Figs 1, 2, 6, 7 and S6. (ZIP) [file pcbi.1004511.s009.zip › S5_Dataset/Lipidome_Homology_Testing/bin/121010_lipidmapstools/docs/html/FAStrGen.html]

LIPID MAPS Tools Documentation: FAStrGen.pl


|  |  |
| --- | --- |
|  | LIPID Metabolites And Pathways Strategy |

  

|  |
| --- |
| PDF  PDFA4 |

## NAME

FAStrGen.pl - Generate structures for Fatty Acyls (FA)

## SYNOPSIS

FAStrGen.pl FAAbbrev|FAAbbrevFileName ...

FAStrGen.pl [**-h, --help**] [**-m, --mode** *Abbrev | AbbrevFileName*]
[**-p, --ProcessMode** *WriteSDFile | CountOnly*] [**-o, --overwrite**]
[**-r, --root** rootname] [**-w, --workingdir** dirname] <arguments>...

## DESCRIPTION

Generate Fatty Acyls (FA) structures using compound abbreviations specified on
a command line or in a CSV/TSV Text file. All the command line arguments represent either
compound abbreviations or file name containing abbreviations. Use mode option to control
the type of command line arguments.

A SD file, containing structures for all SP abbreviations along with ontological information, is
generated as an output.

## SUPPORTED ABBREVIATIONS

Current support for FA structure generation include these main classes and sub classes:

o Fatty Acids and Conjugates

. Straight chain fatty acids
  
 . Methyl branched fatty acids
  
 . Unsaturated fatty acids
  
 . Hydroperoxy fatty acids
  
 . Hydroxy fatty acids
  
 . Oxo fatty acids
  
 . Epoxy fatty acids
  
 . Methoxy fatty acids
  
 . Halogenated fatty acids
  
 . Amino fatty acids
  
 . Cyano fatty acids
  
 . Nitro fatty acids
  
 . Thia fatty acids

o Eicosanoids

. Prostaglandins

o Fatty alcohols

o Fatty aldehydes

o Fatty amides

. Primary amides

## OPTIONS

**-h, --help**
:   Print this help message

**-m, --mode** *Abbrev|AbbrevFileName*
:   Controls interpretation of command line arguments. Two different methods are provided:
    specify compound abbreviations or a file name containing compound abbreviations. Possible
    values: *Abbrev or AbbrevFileName*. Default: *Abbrev*

    In *AbbrevFileName* mode, a single line in CSV/TSV files can contain multiple compound
    abbreviations. The file extension determines delimiter used to process data lines: comma for
    CSV and tab for TSV. For files with TXT extension, only one compound abbreviation per line
    is allowed.

    Wild card character, \*, is also supported in compound abbreviations to generate straight
    chain and unsaturated fatty acids.

    Examples:

    Specific structures: "18:0" "20:4(5Z,8Z,11Z,14Z)"
    "28:1(12Z)(2Me,4Me,6Me)"
    "18:3(6Z,9Z,11E)(13OOH[S])"
    "18:2(9E,11E)(13OH)"
    "18:1(10E)(9Ke,10Ep)"
    "16:1(5Z)(2OMe)" "7:1(2Z)(3Br)"
    "18:2(9Z,12Z)(10NO2)" "16:2(10E,12Z)(1OH)"
    "6:0(1CHO)" "12:0(1NH2)"
    "20:2(5Z,13E)(9OH[S],11OH[R],15OH[S]){8a,12b}"
      
     All possibilites: \*:\* or \*

    With wild card character, +/- can also be used for chain lengths to indicate even and odd lengths;
    additionally > and < qualifiers are also allowed to specify length requirements. Examples:

    Odd number chains: "\*-:\*"
      
     Even number chains: "\*+:\*"
      
     Odd number chains with chain length longer than 18: "\*->18:\*"
      
     Even number chains with chain length longer than 14: "\*+>14:\*"

**-o, --overwrite**
:   Overwrite existing files

**-r, --root** *rootname*
:   New file name is generated using the root: <Root>.sdf. Default for new file names: FAAbbrev.sdf,
    <AbbrevFilenName>.sdf, or <FirstAbbrevFileName>1To<Count>.sdf.

**-p, --ProcessMode** *WriteSDFile|CountOnly*
:   Specify how abbreviations are processed: generate structures for specified abbreviations along
    with generating a SD file or just count the number of structures corresponding to specified
    abbreviations without generating any SD file. Possible values: *WriteSDFile or CountOnly*.
    Default: *WriteSDFile*.

    It can take substantial amount of time for generating all the structures and writing out a SD file
    for abbreviations containing wild cards. *CountOnly* value of **--ProcessMode** option can
    be used to get a quick count of number of structures to be generated without writing out any
    SD file.

**-w, --workingdir** *dirname*
:   Location of working directory. Default: current directory

## EXAMPLES

On some systems, command line scripts may need to be invoked using
*perl -s FAStrGen.pl*; however, all the examples assume direct invocation
of command line script works.

To generate a FAStructures.sdf file containing a structure specified
by a command line FA abbreviation for straight chain fatty acids, type:

% FAStrGen.pl -r FAStructures -o "18:0" "9:0"

To generate a FAStructures.sdf file containing a structure specified
by a command line FA abbreviation for methyl branched fatty acids, type:

% FAStrGen.pl -r FAStructures -o "8:0(6Me)" "18:1(6Z)(17Me)"
"28:1(12Z)(2Me,4Me,6Me)"

To generate a FAStructures.sdf file containing a structure specified
by a command line FA abbreviation for unsaturated fatty acids, type:

% FAStrGen.pl -r FAStructures -o "20:4(5Z,8Z,11Z,14Z)" "8:1(5E)"

To generate a FAStructures.sdf file containing a structure specified
by a command line FA abbreviation for hydroperoxy fatty acids, type:

% FAStrGen.pl -r FAStructures -o "18:2(9E,11E)(13OOH)"
"18:3(6Z,9Z,11E)(13OOH[S])"

To generate a FAStructures.sdf file containing a structure specified
by a command line FA abbreviation for hydroxy fatty acids, type:

% FAStrGen.pl -r FAStructures -o "10:0(10OH)" "15:0(2OH,15OH)"
"18:2(9E,11E)(13OH)" "4:0(3OH[R])"

To generate a FAStructures.sdf file containing a structure specified
by a command line FA abbreviation for oxo fatty acids, type:

% FAStrGen.pl -r FAStructures -o "10:0(2Ke)" "18:1(10E)(9Ke,10Ep)"

To generate a FAStructures.sdf file containing a structure specified
by a command line FA abbreviation for epoxy fatty acids, type:

% FAStrGen.pl -r FAStructures -o "18:0(6Ep)"

To generate a FAStructures.sdf file containing a structure specified
by a command line FA abbreviation for methoxy fatty acids, type:

% FAStrGen.pl -r FAStructures -o "18:1(9E)(12OH,13OH,11OMe)"
"16:1(5Z)(2OMe)"

To generate a FAStructures.sdf file containing a structure specified
by a command line FA abbreviation for halogenated fatty acids, type:

% FAStrGen.pl -r FAStructures -o "7:1(2Z)(3Br)" "26:2(5Z,9Z)(2Br)"

To generate a FAStructures.sdf file containing a structure specified
by a command line FA abbreviation for amino fatty acids, type:

% FAStrGen.pl -r FAStructures -o "13:0(2NH2[S])" "4:0(2NH2,4CN)"

To generate a FAStructures.sdf file containing a structure specified
by a command line FA abbreviation for Cyano fatty acids, type:

% FAStrGen.pl -r FAStructures -o "4:0(4CN)"

To generate a FAStructures.sdf file containing a structure specified
by a command line FA abbreviation for nitro fatty acids, type:

% FAStrGen.pl -r FAStructures -o "18:2(9Z,12Z)(10NO2)"

To generate a FAStructures.sdf file containing a structure specified
by a command line FA abbreviation for prostaglanins, type:

% FAStrGen.pl -r FAStructures -o "20:2(5Z,13E)(9OH[S],11OH[R],
15OH[S]){8a,12b}"

To generate a FAStructures.sdf file containing a structure specified
by a command line FA abbreviation for fatty alcohols, type:

% FAStrGen.pl -r FAStructures -o "26:0(1OH)" "16:2(10E,12Z)(1OH)"
"11:0(1OH,2Me,2Me,9Me,9Me,10OH)"

To generate a FAStructures.sdf file containing a structure specified
by a command line FA abbreviation for fatty aldehydes, type:

% FAStrGen.pl -r FAStructures -o "6:0(1CHO)" "16:2(2E,4E)(1CHO,6OH)"

To generate a FAStructures.sdf file containing a structure specified
by a command line FA abbreviation for primary amides, type:

% FAStrGen.pl -r FAStructures -o "12:0(1NH2)"

To enumerate straight chain and unsaturated fatty acids with commonly occuring
chain lengths and generate FAStructures.sdf file, type:

% FAStrGen.pl -r FAStructures -o "\*"

or

% FAStrGen.pl -r SPStructures -o "\*:\*"

## AUTHOR

Manish Sud

## CONTRIBUTOR

Eoin Fahy

## SEE ALSO

CLStrGen.pl, GLStrGen.pl, GPStrGen.pl, SPStrGen.pl, STStrGen.pl

## COPYRIGHT

Copyright (C) 2006-2012. The Regents of the University of California. All Rights Reserved.

## LICENSE

Modified BSD License
