# Supplementary material for: The LUX Score: A Metric for Lipidome Homology
Source: PLoS Comput Biol. 2015 Sep 22;11(9):e1004511. doi: 10.1371/journal.pcbi.1004511 (PMC4578897; doi:10.1371/journal.pcbi.1004511)
Supplement: S5 Dataset — Includes scripts, README files and data files for Figs 1, 2, 6, 7 and S6. (ZIP) [file pcbi.1004511.s009.zip › S5_Dataset/Lipidome_Homology_Testing/bin/121010_lipidmapstools/docs/html/GLStr.html]

LIPID MAPS Tools Documentation: GLStr.pm


|  |  |
| --- | --- |
|  | LIPID Metabolites And Pathways Strategy |

  

|  |
| --- |
| PDF  PDFA4 |

## NAME

GLStr - Glycerolipids (GL) structure generation methods

## SYNOPSIS

use GLStr;

use GLStr qw(:all);

## DESCRIPTION

GLStr module provides these methods:

GenerateCmpdOntologyData - Generate ontology data
  
 GenerateCmpdOntologySDDataLines - Generate ontology data lines for SD file
  
 GenerateGLChainStrData - Generate chain structure data
  
 GenerateSDFile - Generate SD file
  
 IsGLAbbrevSupported - Is it a supported GL abbreviation
  
 ParseGLAbrev - Parse GL abbreviation
  
 ProcessGLCmpdAbbrevs - Process GL abbreviation
  
 SetupGLCmpdAbbrevTemplateDataMap - Setup template structure data map
  
 ValidateGLAbbrev - Validate GL abbreviation

## METHODS

**ExpandGLCmpdAbbrevs**
:   $ExpandedAbbrevArrayRef = ExpandGLCmpdAbbrevs($CmpdAbbrev);

    Return a reference to an array containing complete GL abbreviations. Wild card
    characters in GL abbreviation name are expanded to generate fully qualified
    GL abbreviations.

**GenerateCmpdOntologyData**
:   $DataHashRef = GenerateCmpdOntologyData($CmpdDataRef);

    Return a reference to a hash containing ontology data with hash keys and values
    corresponding to property names and values.

**GenerateCmpdOntologySDDataLines**
:   $DataLinesArrayRef = GenerateCmpdOntologySDDataLines($CmpDataRef);

    Return a reference to an array containing ontology data lines suitable for
    generate SD file data block.

**GenerateGLChainStrData**
:   ($AtomLinesArrayRef, $BondLinesArrayRef) =
    GenerateGLChainStrData($ChainType, $CmpdDataRef);

    Return array references containing atom and bond data lines for SD file. Appropriate atom
    and bond data lines are generated using chain type and abbreviation template data.

**GenerateSDFile**
:   GenerateSDFile($SDFileName, $CmdAbbrevsRef);

    Generate a SD file for compound abbreviations. Structure data for specified abbreviation
    is generated sequentially and written to SD file.

**IsGLAbbrevSupported**
:   $Status = IsGLAbbrevSupported($Abbrev, $PrintWarning);

    Return 1 or 0 based on whether GL abbreviated is supported. For unsupported GL abbreviations,
    a warning is printed unless PrintWarning flag is set.

**ParseGLAbrev**
:   ($GLType, $ChainsAbbrev, $AbbrevModifier) = ParseGLAbrev($Abbrev);

    Parse GL abbreviation and return these values: GLType, ChainsAbbrev,
    AbbrevModifier.

**ProcessGLCmpdAbbrevs**
:   ProcessGLCmpdAbbrevs($CmpdAbbrevsRef, $AllowArbitraryChainAbbrev,
    $WriteSDFile, $SDFileName);

    Process specified GL abbreviations to generate structures and write them out either
    a SD file or simply report number of valid abbreviations.

**SetupGLCmpdAbbrevTemplateDataMap**
:   $AbbrevTemplateDataMapRef =
    SetupGLCmpdAbbrevTemplateDataMap($Abbrev);

    Return a reference to a hash containing template data for compound abbreviation. The
    template data is used to generate SD file for compound abbreviation.

**ValidateGLAbbrev**
:   $Status = ValidateGLAbbrev($Abbrev);

    Return 1 or 0 based on whether a GL abbreviation is valid.

## AUTHOR

Manish Sud

## CONTRIBUTOR

Eoin Fahy

## SEE ALSO

ChainAbbrev.pm, ChainStr.pm, LMAPSStr.pm

## COPYRIGHT

Copyright (C) 2006-2012. The Regents of the University of California. All Rights Reserved.

## LICENSE

Modified BSD License
