# Supplementary material for: The LUX Score: A Metric for Lipidome Homology
Source: PLoS Comput Biol. 2015 Sep 22;11(9):e1004511. doi: 10.1371/journal.pcbi.1004511 (PMC4578897; doi:10.1371/journal.pcbi.1004511)
Supplement: S5 Dataset — Includes scripts, README files and data files for Figs 1, 2, 6, 7 and S6. (ZIP) [file pcbi.1004511.s009.zip › S5_Dataset/Lipidome_Homology_Testing/bin/121010_lipidmapstools/docs/html/GLStrGen.html]

LIPID MAPS Tools Documentation: GLStrGen.pl


|  |  |
| --- | --- |
|  | LIPID Metabolites And Pathways Strategy |

  

|  |
| --- |
| PDF  PDFA4 |

## NAME

GLStrGen.pl - Generate structures for Glycerolipids (GL)

## SYNOPSIS

GLStrGen.pl GLAbbrev|GLAbbrevFileName ...

GLStrGen.pl [**-c, --ChainAbbrevMode** *MostLikely | Arbitrary*]
[**-h, --help**] [**-m, --mode** *Abbrev | AbbrevFileName*]
[**-p, --ProcessMode** *WriteSDFile | CountOnly*] [**-o, --overwrite**]
[**-r, --root** rootname] [**-w, --workingdir** dirname] <arguments>...

## DESCRIPTION

Generate Glycerolipids (GL) structures using compound abbreviations specified on a command line
or in a CSV/TSV Text file. All the command line arguments represent either compound
abbreviations or file name containing abbreviations. Use *-m, --mode* option to control the type
of command line arguments.

A SD file, containing structures for all GL abbreviations along with ontological information, is
generated as an output.

## SUPPORTED ABBREVIATIONS

Current support for GL structure generation include these main classes and sub classes:

o Monoradylglycerols

. Monoacylglycerols
  
 . Monoalkylglycerols
  
 . Mono-(1Z-alkenyl)-glycerol

o Diradylglycerols

. Diacylglycerols
  
 . Alkyl, acylglycerols
  
 . Dialkylglycerols
  
 . 1Z-alkenyl, acylglycerols

o Triradylglycerols

. Triacylglycerols
  
 . Alkyl, diacylglycerols
  
 . Dialkyl, monoacylglycerols
  
 . 1Z-alkenyl, diacylglycerols

## OPTIONS

**-c, --ChainAbbrevMode** *MostLikely|Arbitrary*
:   Specify what types of acyl chain abbreviations are allowed during processing of complete
    abbreviations: allow most likely chain abbreviations containing specific double bond geometry
    specifications; allow any acyl chain abbreviation with valid chain length and double bond
    geometry specificatios. Possible values: *MostLikely or Arbitrary*. Default value: *MostLikely*.

    *Arbitrary* value of **-c, --ChainAbbrevMode** option is not allowed during processing of
    abbreviations containing wild cards.

    During *MostLikely* value of **-c, --ChainAbbrevMode** option, only the most likely acyl chain
    abbreviations specified in ChainAbbrev.pm module are allowed. However, during *Arbitrary* value
    of **-c, --ChainAbbrevMode** option, any acyl chain abbreviations with valid chain length and
    double bond geometry can be specified. The current release of lipidmapstools support chain
    lengths from 2 to 50 as specified in ChainAbbev.pm module.

    In addition to double bond geometry specifications, valid substituents can be specified for in the acyl
    chain abbreviations.

**-h, --help**
:   Print this help message

**-m, --mode** *Abbrev|AbbrevFileName*
:   Controls interpretation of command line arguments. Two different methods are provided:
    specify compound abbreviations or a file name containing compound abbreviations. Possible
    values: *Abbrev or AbbrevFileName*. Default: *Abbrev*

    In *AbbrevFileName* mode, a single line in CSV/TSV files can contain multiple compound
    abbreviations. The file extension determines delimiter used to process data lines: comma for
    CSV and tab for TSV. For files with TXT extension, only one compound abbreviation per line
    is allowed.

    Wild card character, \*, is also supported in compound abbreviations.

    Examples:

    Specific structures: MG(16:0/0:0/0:0) DG(18:1(11E)/16:0/0:0)
    TG(16:0/16:0/18:1(9Z))
      
     Specific structures: MG(O-16:0/0:0/0:0) DG(P-16:0/16:0/0:0)
    TG(O-20:0/16:0/18:1(9Z))
      
     Specific possibilities: DG(18:\*/16:0/0:0) DG(18:1(\*)/16:0/0:0)
    DG(\*:\*(9Z)/16:0/0:0)
    DG(\*:\*(9Z)/\*:\*(11E)/0:0)
      
     All TG possibilities: \*(\*:\*/\*:\*/\*:\*) or \*(\*/\*/\*)
      
     All MG, DG and TG possibilities: "MG(\*:\*/0:0/0:0)" "DG(\*:\*/\*:\*/0:0)"
    "DG(\*.\*/0:0/\*:\*)" "TG(\*:\*/\*:\*/\*:\*)"

    Along with wild card character, +/- can also be used for chain lengths to indicate even and odd lengths at
    sn1/sn2/sn3 positions; additionally > and < qualifiers are also allowed to specify length requirements.
    Examples:

    Odd and even number chains at sn1 and sn2: TG(\*+:\*/\*-:\*/\*:\*)
      
     Odd and even number chains at sn1 and sn2 with length longer than
      
     10 and 20: TG(\*+>10:\*/\*->20:\*/\*:\*)

    Default sn2 stereochemistry is R. However, abbreviation format also supports these additional stereochemistry
    specifications for sn2 position: S; U - unknown; rac - racemic mixture. Examples:

    MG(16:0/0:0/0:0)[rac] - racemic mixture
      
     DG(18:1(11E)/16:0/0:0)[S] - sn2 stereochemistry is S instead of default R
      
     TG(16:0/16:0/18:1(9Z))[U] - sn2 stereochemistry is unknown

    To generate all isomers for specific chains in DG and TG, use of iso designation is also supported.
    Stereochemistry specification support is not available with isomeric structure generation.
    Examples:

    DG(18:1(11E)/16:0/0:0)[iso2] - Two isomeric structures
      
     TG(16:0/16:0/18:1(9Z))[iso3] - Three isomeric structures
      
     TG(16:0/18:0/18:1(9Z))[iso6] - Six isomeric structures

    Additionally, all isomeric structures can also be generated by explicit specification of chains at
    different positions:

    DG(18:1(11E)/16:0/0:0) DG(16:0/18:1(11E)/0:0)
      
     TG(16:0/16:0/18:1(9Z)) TG(16:0/18:1(9Z)/16:0)
      
     TG(18:1(9Z)/16:0/16:0/)

    Wild card chain abbreviations are supported with sn2 stereochemistry but not with
    isomer abbreviation.

**-p, --ProcessMode** *WriteSDFile|CountOnly*
:   Specify how abbreviations are processed: generate structures for specified abbreviations along
    with generating a SD file or just count the number of structures corresponding to specified
    abbreviations without generating any SD file. Possible values: *WriteSDFile or CountOnly*.
    Default: *WriteSDFile*.

    It can take substantial amount of time for generating all the structures and writing out a SD file
    for abbreviations containing wild cards. *CountOnly* value of **--ProcessMode** option can
    be used to get a quick count of number of structures to be generated without writing out any
    SD file.

**-o, --overwrite**
:   Overwrite existing files

**-r, --root** *rootname*
:   New file name is generated using the root: <Root>.sdf. Default for new file names: GLAbbrev.sdf,
    <AbbrevFilenName>.sdf, or <FirstAbbrevFileName>1To<Count>.sdf.

**-w, --workingdir** *dirname*
:   Location of working directory. Default: current directory

## EXAMPLES

On some systems, command line scripts may need to be invoked using
*perl -s GLStrGen.pl*; however, all the examples assume direct invocation
of command line script works.

To generate a GLStructures.sdf file containing a structure specified
by a command line GL abbreviation, type:

% GLStrGen.pl -r GLStructures -o "MG(16:0/0:0/0:0)"

To generate a GLStructures.sdf file containing structures specified
by a command line GL abbreviations, type:

% GLStrGen.pl -r GLStructures -o "MG(16:0/0:0/0:0)"
"DG(18:1(11E)/16:0/0:0)" "TG(16:0/16:0/18:1(9Z))"

To generate a GLStructures.sdf file containing structures specified
by a command line GL abbreviations with specific stereochemistry, type:

% GLStrGen.pl -r GLStructures -o "MG(16:0/0:0/0:0)[rac]"
"DG(18:1(11E)/16:0/0:0)[S]" "TG(16:0/16:0/18:1(9Z))[U]"

To generate a GLStructures.sdf file containing all isomeric structures specified
by a command line GL abbreviations, type:

% GLStrGen.pl -r GLStructures -o "DG(18:1(11E)/16:0/0:0)[iso2]"
"TG(16:0/16:0/18:1(9Z))[iso3]" "TG(16:0/17:0/18:1(9Z))[iso6]"

To enumerate all possible GL structures and generate a GLStructures.sdf
file, type:

% GLStrGen.pl -r GLStructures -o "\*(\*/\*/\*)"

or

% GLStrGen.pl -r GLStructures -o "\*(\*:\*/\*:\*/\*:\*)"

or

% GLStrGen.pl -r GLStructures -o "\*(\*:\*(\*)/\*:\*(\*)/\*:\*(\*))"

To enumerate all possible Monoradylglycerols structures and generate a
MonoGLStructures.sdf file, type:

% GLStrGen.pl -r MonoGLStructures -o "MG(\*/0:0/0:0)"

To enumerate all possible Diradylglycerols structures and generate a
DiGLStructures.sdf file, type:

% GLStrGen.pl -r DiGLStructures -o "DG(\*/\*/0:0)"

To enumerate all possible Monoradylglycerols structures with one double
bond on acyl chain and generate a GLStructures.sdf file, type:

% GLStrGen.pl -r MonoGLStructures -o "MG(\*:1/0:0/0:0)"

To enumerate all possible Monoradylglycerols structures with even chain
lengths and generate a GLStructures.sdf file, type:

% GLStrGen.pl -r MonoGLStructures -o "MG(\*+:\*/0:0/0:0)"

To enumerate all possible Diradylglycerols structures with odd chains longer
than 10 at sn1 and even chains longer than 18 at sn2, and generate a
DiGLStructures.sdf file, type:

% GLStrGen.pl -r DiGLStructures -o "DG(\*->10:\*/\*+>18:\*/0:0)"

## AUTHOR

Manish Sud

## CONTRIBUTOR

Eoin Fahy

## SEE ALSO

CLStrGen.pl, FAStrGen.pl, GPStrGen.pl, SPStrGen.pl, STStrGen.pl

## COPYRIGHT

Copyright (C) 2006-2012. The Regents of the University of California. All Rights Reserved.

## LICENSE

Modified BSD License
