# Supplementary material for: The LUX Score: A Metric for Lipidome Homology
Source: PLoS Comput Biol. 2015 Sep 22;11(9):e1004511. doi: 10.1371/journal.pcbi.1004511 (PMC4578897; doi:10.1371/journal.pcbi.1004511)
Supplement: S5 Dataset — Includes scripts, README files and data files for Figs 1, 2, 6, 7 and S6. (ZIP) [file pcbi.1004511.s009.zip › S5_Dataset/Lipidome_Homology_Testing/bin/121010_lipidmapstools/docs/html/GPStrGen.html]

LIPID MAPS Tools Documentation: GPStrGen.pl


|  |  |
| --- | --- |
|  | LIPID Metabolites And Pathways Strategy |

  

|  |
| --- |
| PDF  PDFA4 |

## NAME

GPStrGen.pl - Generate structures for Glycerophospholipids (GP)

## SYNOPSIS

GPStrGen.pl GPAbbrev|GPAbbrevFileName ...

GPStrGen.pl [**-c, --ChainAbbrevMode** *MostLikely | Arbitrary*]
[**-h, --help**] [**-m, --mode** *Abbrev | AbbrevFileName*]
[**-p, --ProcessMode** *WriteSDFile | CountOnly*] [**-o, --overwrite**]
[**-r, --root** rootname] [**-w, --workingdir** dirname] <arguments>...

## DESCRIPTION

Generate Glyceriphospholipids (GP) structures using compound abbreviations specified on
a command line or in a CSV/TSV Text file. All the command line arguments represent either
compound abbreviations or file name containing abbreviations. Use mode option to control
the type of command line arguments.

A SD file, containing structures for all GP abbreviations along with ontological information, is
generated as an output.

## SUPPORTED ABBREVIATIONS

Current support for GP structure generation include these main classes and sub classes:

o Glycerophosphocholines (PC)

. Diacylglycerophosphocholines
  
 . 1-alkyl,2-acylglycerophosphocholines
  
 . 1Z-alkenyl,2-acylglycerophosphocholines
  
 . Dialkylglycerophosphocholines
  
 . Monoacylglycerophosphocholines
  
 . 1-alkyl glycerophosphocholines
  
 . 1Z-alkenylglycerophosphocholines

o Glycerophosphoethanolamines (PE)

. Diacylglycerophosphoethanolamines
  
 . 1-alkyl,2-acylglycerophosphoethanolamines
  
 . 1Z-alkenyl,2-acylglycerophosphoethanolamines
  
 . Dialkylglycerophosphoethanolamines
  
 . Monoacylglycerophosphoethanolamines
  
 . 1-alkyl glycerophosphoethanolamines
  
 . 1Z-alkenylglycerophosphoethanolamines

o Glycerophosphoserines (PS)

. Diacylglycerophosphoserines
  
 . 1-alkyl,2-acylglycerophosphoserines
  
 . 1Z-alkenyl,2-acylglycerophosphoserines
  
 . Dialkylglycerophosphoserines
  
 . Monoacylglycerophosphoserines
  
 . 1-alkyl glycerophosphoserines
  
 . 1Z-alkenylglycerophosphoserines

o Glycerophosphoglycerols (PG)

. Diacylglycerophosphoglycerols
  
 . 1-alkyl,2-acylglycerophosphoglycerols
  
 . 1Z-alkenyl,2-acylglycerophosphoglycerols
  
 . Dialkylglycerophosphoglycerols
  
 . Monoacylglycerophosphoglycerols
  
 . 1-alkyl glycerophosphoglycerols
  
 . 1Z-alkenylglycerophosphoglycerols

o Glycerophosphoglycerophosphates (PGP)

. Diacylglycerophosphoglycerophosphates
  
 . 1-alkyl,2-acylglycerophosphoglycerophosphates
  
 . 1Z-alkenyl,2-acylglycerophosphoglycerophosphates
  
 . Dialkylglycerophosphoglycerophosphates
  
 . Monoacylglycerophosphoglycerophosphates
  
 . 1-alkyl glycerophosphoglycerophosphates
  
 . 1Z-alkenylglycerophosphoglycerophosphates

o Glycerophosphoinositols (PI)

. Diacylglycerophosphoinositols
  
 . 1-alkyl,2-acylglycerophosphoinositols
  
 . 1Z-alkenyl,2-acylglycerophosphoinositols
  
 . Dialkylglycerophosphoinositols
  
 . Monoacylglycerophosphoinositols
  
 . 1-alkyl glycerophosphoinositols
  
 . 1Z-alkenylglycerophosphoinositols

o Glycerophosphoinositol monophosphates (PIP)

. Diacylglycerophosphoinositol monophosphates
  
 . 1-alkyl,2-acylglycerophosphoinositol monophosphates
  
 . 1Z-alkenyl,2-acylglycerophosphoinositol monophosphates
  
 . Dialkylglycerophosphoinositol monophosphates
  
 . Monoacylglycerophosphoinositol monophosphates
  
 . 1-alkyl glycerophosphoinositol monophosphates
  
 . 1Z-alkenylglycerophosphoinositol monophosphates

o Glycerophosphates (PA)

. Diacylglycerophosphates
  
 . 1-alkyl,2-acylglycerophosphates
  
 . 1Z-alkenyl,2-acylglycerophosphates
  
 . Dialkylglycerophosphates
  
 . Monoacylglycerophosphates
  
 . 1-alkyl glycerophosphates
  
 . 1Z-alkenylglycerophosphates

o Glyceropyrophosphates (PPA)

. Diacylglyceropyrophosphates
  
 . Monoacylglyceropyrophosphates

o Glycerophosphonocholines (PnC)

. Diacylglycerophosphonocholines
  
 . 1-alkyl,2-acylglycerophosphonocholines
  
 . 1Z-alkenyl,2-acylglycerophosphonocholines
  
 . Dialkylglycerophosphonocholines
  
 . Monoacylglycerophosphonocholines
  
 . 1-alkyl glycerophosphonocholines
  
 . 1Z-alkenylglycerophosphonocholines

o Glycerophosphonoethanolamines (PnE)

. Diacylglycerophosphonoethanolamines
  
 . 1-alkyl,2-acylglycerophosphonoethanolamines
  
 . 1Z-alkenyl,2-acylglycerophosphonoethanolamines
  
 . Dialkylglycerophosphonoethanolamines
  
 . Monoacylglycerophosphonoethanolamines
  
 . 1-alkyl glycerophosphonoethanolamines
  
 . 1Z-alkenylglycerophosphonoethanolamines

## OPTIONS

**-c, --ChainAbbrevMode** *MostLikely|Arbitrary*
:   Specify what types of acyl chain abbreviations are allowed during processing of complete
    abbreviations: allow most likely chain abbreviations containing specific double bond geometry
    specifications; allow any acyl chain abbreviation with valid chain length and double bond
    geometry specificatios. Possible values: *MostLikely or Arbitrary*. Default value: *MostLikely*.

    *Arbitrary* value of **-c, --ChainAbbrevMode** option is not allowed during processing of
    abbreviations containing wild cards.

    During *MostLikely* value of **-c, --ChainAbbrevMode** option, only the most likely acyl chain
    abbreviations specified in ChainAbbrev.pm module are allowed. However, during *Arbitrary* value
    of **-c, --ChainAbbrevMode** option, any acyl chain abbreviations with valid chain length and
    double bond geometry can be specified. The current release of lipidmapstools support chain
    lengths from 2 to 50 as specified in ChainAbbev.pm module.

    In addition to double bond geometry specifications, valid substituents can be specified for in the acyl
    chain abbreviations.

**-h, --help**
:   Print this help message

**-m, --mode** *Abbrev|AbbrevFileName*
:   Controls interpretation of command line arguments. Two different methods are provided:
    specify compound abbreviations or a file name containing compound abbreviations. Possible
    values: *Abbrev or AbbrevFileName*. Default: *Abbrev*

    In *AbbrevFileName* mode, a single line in CSV/TSV files can contain multiple compound
    abbreviations. The file extension determines delimiter used to process data lines: comma for
    CSV and tab for TSV. For files with TXT extension, only one compound abbreviation per line
    is allowed.

    Wild card character, \*, is also supported in compound abbreviations.

    Examples:

    Specific structures: PC(12:0/13:0) PC(17:1(9Z)/0:0)
    PA(13:0/0:0)
      
     Specific structures: PC(O-16:0/13:0) PC(P-16:0/0:0)
      
     Specific possibilities: PC(21:0/22:\*) PA(17:\*/0:0)
    PE(O-18:0/\*:\*)
      
     All possibilites: \*(\*:\*/\*:\*) or \*(\*/\*)

    With wild card character, +/- can also be used for chain lengths to indicate even and odd lengths at
    sn1/sn2/sn3 positions; additionally > and < qualifiers are also allowed to specify length
    requirements. Examples:

    Odd and even number chains at sn1 and sn2: \*(\*+:\*/\*-:\*)
      
     Odd and even number chains at sn1 and sn2 with length longer than 10
    and 20: \*(\*+>10:\*/\*->20:\*)

    Default sn2 stereochemistry is R. However, abbreviation format also supports these additional stereochemistry
    specifications for sn2 position: S; U - unknown; rac - racemic mixture. Examples:

    PC(12:0/13:0)[rac]
      
     PC(17:1(9Z)/14:0)[S]
      
     PA(13:0/12:0)[U]

**-p, --ProcessMode** *WriteSDFile|CountOnly*
:   Specify how abbreviations are processed: generate structures for specified abbreviations along
    with generating a SD file or just count the number of structures corresponding to specified
    abbreviations without generating any SD file. Possible values: *WriteSDFile or CountOnly*.
    Default: *WriteSDFile*.

    It can take substantial amount of time for generating all the structures and writing out a SD file
    for abbreviations containing wild cards. *CountOnly* value of **--ProcessMode** option can
    be used to get a quick count of number of structures to be generated without writing out any
    SD file.

**-o, --overwrite**
:   Overwrite existing files

**-r, --root** *rootname*
:   New file name is generated using the root: <Root>.sdf. Default for new file names: GPAbbrev.sdf,
    <AbbrevFilenName>.sdf, or <FirstAbbrevFileName>1To<Count>.sdf.

**-w, --workingdir** *dirname*
:   Location of working directory. Default: current directory

## EXAMPLES

On some systems, command line scripts may need to be invoked using
*perl -s GLStrGen.pl*; however, all the examples assume direct invocation
of command line script works.

To generate a GPStructures.sdf file containing a structure specified
by a command line GP abbreviation, type:

% GPStrGen.pl -r GPStructures -o "PC(16:0/0:0)"

To generate a GPStructures.sdf file containing structures specified
by a command line GL abbreviations, type:

% GPStrGen.pl -r GPStructures -o "PC(16:0/0:0)" "PE(18:1(11E)/16:0)"

To generate a GPStructures.sdf file containing structures specified
by a command line GP abbreviations with specific stereochemistry, type:

% GPStrGen.pl -r GPStructures -o "PC(16:0/0:0)[U]"
"PE(18:1(11E)/16:0)[S]"

To enumerate all possible GP structures and generate a GPStructures.sdf
file, type:

% GPStrGen.pl -r GPStructures -o "\*(\*/\*)"

or

% GPStrGen.pl -r GPStructures -o "\*(\*:\*/\*:\*)"

or

% GPStrGen.pl -r GPStructures -o "\*(\*:\*(\*)/\*:\*(\*))"

To enumerate all possible GP structures with a sn1 chain, and generate a
GPStructures.sdf file, type:

% GPStrGen.pl -r GPStructures -o "\*(\*/0:0)"

To enumerate all possible GP structures with a sn1 chain containing one
double bond, and generate a GPStructures.sdf file, type:

% GPStrGen.pl -r GPStructures -o "\*(\*:1/0:0)"

To enumerate all possible GP structures with even chain length larger than
10 at sn1 position, and generate and generate a GPStructures.sdf file, type:

% GPStrGen.pl -r GPStructures -o "\*(\*+>10:\*/0:0)"

To enumerate all possible GP structures with odd chains longer
than 10 at sn1 and even chains longer than 18 at sn2, and generate a
GPStructures.sdf file, type:

% GPStrGen.pl -r GPStructures -o "\*(\*->10:\*/\*+>18:\*)"

## AUTHOR

Manish Sud

## CONTRIBUTOR

Eoin Fahy

## SEE ALSO

CLStrGen.pl, FAStrGen.pl, GLStrGen.pl, SPStrGen.pl, STStrGen.pl

## COPYRIGHT

Copyright (C) 2006-2012. The Regents of the University of California. All Rights Reserved.

## LICENSE

Modified BSD License
