# Supplementary material for: The LUX Score: A Metric for Lipidome Homology
Source: PLoS Comput Biol. 2015 Sep 22;11(9):e1004511. doi: 10.1371/journal.pcbi.1004511 (PMC4578897; doi:10.1371/journal.pcbi.1004511)
Supplement: S5 Dataset — Includes scripts, README files and data files for Figs 1, 2, 6, 7 and S6. (ZIP) [file pcbi.1004511.s009.zip › S5_Dataset/Lipidome_Homology_Testing/bin/121010_lipidmapstools/docs/html/LMAPSStr.html]

LIPID MAPS Tools Documentation: LMAPSStr.pm


|  |  |
| --- | --- |
|  | LIPID Metabolites And Pathways Strategy |

  

|  |
| --- |
| PDF  PDFA4 |

## NAME

LMAPSStr - Glycerolipids (GL) structure generation methods

## SYNOPSIS

use LMAPSStr;

use LMAPSStr qw(:all);

## DESCRIPTION

LMAPSStr module provides these methods:

GenerateCmpdAtomLine - Generate SD file atom data line
  
 GenerateCmpdBondLine - Generate SD file bond data line
  
 GenerateCmpdCountsLine - Generate SD file count data line
  
 GenerateCmpdMiscInfoLine - Generate SD file misc data line
  
 ParseCmpdAtomLine - Parse SD file atom data line
  
 ParseCmpdBondLine - Parse SD file bond data line
  
 ParseCmpdCountsLine - Parse SD file count data line
  
 RoundToNextInteger - Round up to next integer
  
 SetupCmpdAbbrevs - Setup lipid abbreviations
  
 SetupSDFileName - Setup SD file name
  
 StandardizeStereochemistrySpec - Standardize stereochemistry
  
 StandardizeStereochemistrySpec - Standardize ring stereochemistry

## METHODS

**GenerateCmpdAtomLine**
:   $Line = GenerateCmpdAtomLine($AtomX, $AtomY, $AtomZ, $AtomSymbol);

    Return a formatted atom data line for SD file.

**GenerateCmpdBondLine**
:   $Line = GenerateCmpdBondLine($FirstAtomNum, $SecondAtomNum,
    $BondType, [$BondStereo]);

    Return a formatted bond data line for SD file.

**GenerateCmpdCountsLine**
:   $Line = GenerateCmpdCountsLine($AtomCount, $BondCount,
    [$ChiralFlag, $PropertyCount, $Version]);

    Return a formatted count data line for SD file.

**GenerateCmpdMiscInfoLine**
:   $Line = GenerateCmpdMiscInfoLine();

    Return a formatted miscellaneous data line for SD file. In addition to a time stamp, LipdMAPS
    name is used as the program name.

**ParseCmpdAtomLine**
:   ($AtomX, $AtomY, $AtomZ, $AtomSymbol) = ParseCmpdAtomLine($Line);

    Parse SD file atom data line and return a list with these values: atom coordinates and
    element symbol.

**ParseCmpdBondLine**
:   ($FirstAtomNum, $SecondAtomNum, $BondType, $BondStereo) =
    ParseCmpdBondLine($Line);

    Parse SD file atom bond data line and return a list containing these values: bond atom numbers and
    bond type.

**ParseCmpdCountsLine**
:   ($AtomCount, $BondCount, $ChiralFlag, $PropertyCount, $Version) =
    ParseCmpdCountsLine($Line);

    Parse SD file count data line and return a list containing these values: atom/bond count and other
    miscellaneous count information.

**RoundToNextInteger**
:   $IntegerValue = RoundToNextInteger($Number);

    Return an integer by rounding the number off to next integer.

**SetupCmpdAbbrevs**
:   $AbbrebArrayRef = SetupCmpdAbbrevs($CmdLineOptionsRef);

    Return a reference to an array containing specified compound abbreviations by parsing
    command line arguments or processing files containing specified abbreviations.

**SetupSDFileName**
:   $SDFilename = SetupSDFileName($LipidCategory, $CmdLineOptionsRef);

    Return a SD file name by processing a specified *-r, --root* option or using default
    values.

**StandardizeStereochemistrySpec**
:   $StandardizeSpec = StandardizeStereochemistrySpec($StereochemistrySpec);

    Return a standardize stereochemistry specification containg R/S instead of a/b or
    alpha/beta.

**StandardizeRingStereochemistrySpec**
:   $StandardizeSpec = StandardizeRingStereochemistrySpec($StereochemistrySpec);

    Return a standardize stereochemistry specification containg alpha/beta instead of
    a/b or alpha/beta.

## AUTHOR

Manish Sud

## CONTRIBUTOR

Eoin Fahy

## SEE ALSO

ChainAbbrev.pm, ChainStr.pm

## COPYRIGHT

Copyright (C) 2006-2012. The Regents of the University of California. All Rights Reserved.

## LICENSE

Modified BSD License
