# Supplementary material for: The LUX Score: A Metric for Lipidome Homology
Source: PLoS Comput Biol. 2015 Sep 22;11(9):e1004511. doi: 10.1371/journal.pcbi.1004511 (PMC4578897; doi:10.1371/journal.pcbi.1004511)
Supplement: S5 Dataset — Includes scripts, README files and data files for Figs 1, 2, 6, 7 and S6. (ZIP) [file pcbi.1004511.s009.zip › S5_Dataset/Lipidome_Homology_Testing/bin/121010_lipidmapstools/docs/html/LMStr.html]

LIPID MAPS Tools Documentation: LMStr.pm


|  |  |
| --- | --- |
|  | LIPID Metabolites And Pathways Strategy |

  

|  |
| --- |
| PDF  PDFA4 |

## NAME

LMStr - LIPID MAPS arbitrary structure generation methods

## SYNOPSIS

use LMStr;

use LMStr qw(:all);

## DESCRIPTION

LMStr module provides these methods:

ExpandLMCmpdAbbrevs - Expand abbreviation
  
 GenerateCmpdOntologyData - Generate ontology data
  
 GenerateCmpdOntologySDDataLines - Generate ontology data lines for SD file
  
 GenerateLMChainStrData - Generate chain structure data
  
 GenerateSDFile - Generate SD file
  
 GetLMTemplatesData - Get templates data
  
 GetLMSupportedHeadGroupMap - Get supported headgroups data
  
 GetLMTemplateID - Get templates ID
  
 IsLMChainsAbbrevSupported - Is it a supported abbreviation
  
 ParseLMAbbrev - Parse abbreviation
  
 SetupLMCmpdAbbrevTemplateDataMap - Setup template structure data map
  
 ValidateLMAbbrev - Validate abbreviation

## METHODS

**ExpandLMCmpdAbbrevs**
:   $ExpandedAbbrevArrayRef = ExpandLMCmpdAbbrevs($CmpdAbbrev);

    Return a reference to an array containing complete LM abbreviations. Wild card
    characters in LM abbreviation name are expanded to generate fully qualified
    LM abbreviations.

**GenerateCmpdOntologyData**
:   $DataHashRef = GenerateCmpdOntologyData($CmpDataRef);

    Return a reference to a hash containing ontology data with hash keys and values
    corresponding to property names and values.

**GenerateCmpdOntologySDDataLines**
:   $DataLinesArrayRef = GenerateCmpdOntologySDDataLines($CmpdDataRef);

    Return a reference to an array containing ontology data lines suitable for
    generate SD file data block.

**GenerateLMChainStrData**
:   ($AtomLinesArrayRef, $BondLinesArrayRef) =
    GenerateLMChainStrData($ChainType, $CmpdDataRef);

    Return array references containing atom and bond data lines for SD file. Appropriate atom
    and bond data lines are generated using chain type and abbreviation template data.

**GenerateSDFile**
:   GenerateSDFile($SDFileName, $CmdAbbrevsRef);

    Generate a SD file for compound abbreviations. Structure data for specified abbreviation
    is generated sequentially and written to SD file.

**GetLMTemplatesData**
:   $TemplatesDataRef = GetLMTemplatesData();

    Return a reference to a hash containing LM templates data

**GetLMSupportedHeadGroupMap**
:   $SupportedHeadGroupDataRef = GetLMSupportedHeadGroupMap();

    Return a reference to a hash containing supported head groups data.

**GetLMTemplateID**
:   $HeadGroupID = GetLMTemplateID($HeadGroupAbbrev, $ChainsAbbrev);

    Return a supported template ID for compound abbreviation.

**IsLMChainsAbbrevSupported**
:   $Status = IsLMChainsAbbrevSupported($Abbrev, $PrintWarning);

    Return 1 or 0 based on whether LM abbreviated is supported. For unsupported LM abbreviations,
    a warning is printed unless PrintWarning flag is set.

**ParseLMAbbrev**
:   ($HeadGroup, $ChainsAbbrev, $AbbrevModifier) =
    ParseLMAbbrev($Abbrev);

    Parse LM abbreviation and return these values: HeadGroup, ChainsAbbrev,
    AbbrevModifier.

**SetupLMCmpdAbbrevTemplateDataMap**
:   $AbbrevTemplateDataMapRef =
    SetupLMCmpdAbbrevTemplateDataMap($Abbrev);

    Return a reference to a hash containing template data for compound abbreviation. The
    template data is used to generate SD file for compound abbreviation.

**ValidateLMAbbrev**
:   $Status = ValidateLMAbbrev($Abbrev);

    Return 1 or 0 based on whether a LM abbreviation is valid.

## AUTHOR

Manish Sud

## CONTRIBUTOR

Eoin Fahy

## SEE ALSO

ChainAbbrev.pm, ChainStr.pm, LMAPSStr.pm

## COPYRIGHT

Copyright (C) 2006-2012. The Regents of the University of California. All Rights Reserved.

## LICENSE

Modified BSD License
