# Supplementary material for: The LUX Score: A Metric for Lipidome Homology
Source: PLoS Comput Biol. 2015 Sep 22;11(9):e1004511. doi: 10.1371/journal.pcbi.1004511 (PMC4578897; doi:10.1371/journal.pcbi.1004511)
Supplement: S5 Dataset — Includes scripts, README files and data files for Figs 1, 2, 6, 7 and S6. (ZIP) [file pcbi.1004511.s009.zip › S5_Dataset/Lipidome_Homology_Testing/bin/121010_lipidmapstools/docs/html/LMStrGen.html]

LIPID MAPS Tools Documentation: LMStrGen.pl


|  |  |
| --- | --- |
|  | LIPID Metabolites And Pathways Strategy |

  

|  |
| --- |
| PDF  PDFA4 |

## NAME

LMStrGen.pl - Generate arbitrary LIPID MAPS structures

## SYNOPSIS

LMStrGen.pl LMAbbrev|LMAbbrevFileName ...

LMStrGen.pl [**-h, --help**] [**-o, --overwrite**] [**-r, --root** rootname]
[**-w, --workingdir** dirname] <arguments>...

## DESCRIPTION

Generate arbitrary LIPID MAPS (LM) structures using compound abbreviations specified on
a command line or in a CSV/TSV Text file. All the command line arguments represent either
compound abbreviations or file name containing abbreviations. Use mode option to control
the type of command line arguments.

A SD file, containing structures for all LM abbreviations along with ontological information, is
generated as an output.

## SUPPORTED ABBREVIATIONS

Current support for LM structure generation include these headgroups and acyl chains:

o DIMAXX - sn1, sn2 and sn3
o DIMA20 - sn1 and sn2
o DIMA22 - sn1 and sn2
o DIMA20Me - sn1 and sn2
o DIMA22Me - sn1 and sn2
o DIMB20 - sn1 and sn2
o DIMB22 - sn1 and sn2
o DIB20Me - sn1 and sn2
o DIB22Me - sn1 and sn2
o PIM1 - sn1 and sn2
o PIM2 - sn1 and sn2
o PIM3 - sn1 and sn2
o PIM4 - sn1 and sn2
o PIM5 - sn1 and sn2
o PIM6 - sn1 and sn2
o DAT - sn1 and sn2
o CoA - sn1

## OPTIONS

**-h, --help**
:   Print this help message

**-m, --mode** *Abbrev|AbbrevFileName*
:   Controls interpretation of command line arguments. Two different methods are provided:
    specify compound abbreviations or a file name containing compound abbreviations. Possible
    values: *Abbrev or AbbrevFileName*. Default: *Abbrev*

    In *AbbrevFileName* mode, a single line in CSV/TSV files can contain multiple compound
    abbreviations. The file extension determines delimiter used to process data lines: comma for
    CSV and tab for TSV. For files with TXT extension, only one compound abbreviation per line
    is allowed.

    Wild card character, \*, is not supported in compound abbreviations.

    Examples:

    Specific structures: DIMA20(12:0/13:0) DIMB20(17:1(9Z)/18:0)
    CoA(16:0) PIM1(21:0/22:0)

**-o, --overwrite**
:   Overwrite existing files

**-r, --root** *rootname*
:   New file name is generated using the root: <Root>.sdf. Default for new file names: LMAbbrev.sdf,
    <AbbrevFilenName>.sdf, or <FirstAbbrevFileName>1To<Count>.sdf.

**-w, --workingdir** *dirname*
:   Location of working directory. Default: current directory

## EXAMPLES

On some systems, command line scripts may need to be invoked using
*perl -s LMStrGen.pl*; however, all the examples assume direct invocation
of command line script works.

To generate a LMStructures.sdf file containing a structure specified
by a command line LM abbreviation, type:

% LMStrGen.pl -r LMStructures -o "DIMA20(12:0/13:0)"

To generate a LMStructures.sdf file containing structures specified
by a command line LM abbreviations, type:

% LMStrGen.pl -r LMStructures -o "DIMA22(16:0/18:0)" "DIMA22Me(18:1(11E)/16:0)"

## AUTHOR

Manish Sud

## CONTRIBUTOR

Eoin Fahy

## SEE ALSO

CLStrGen.pl, FAStrGen.pl, GLStrGen.pl, SPStrGen.pl, STStrGen.pl

## COPYRIGHT

Copyright (C) 2006-2012. The Regents of the University of California. All Rights Reserved.

## LICENSE

Modified BSD License
