# Supplementary material for: The LUX Score: A Metric for Lipidome Homology
Source: PLoS Comput Biol. 2015 Sep 22;11(9):e1004511. doi: 10.1371/journal.pcbi.1004511 (PMC4578897; doi:10.1371/journal.pcbi.1004511)
Supplement: S5 Dataset — Includes scripts, README files and data files for Figs 1, 2, 6, 7 and S6. (ZIP) [file pcbi.1004511.s009.zip › S5_Dataset/Lipidome_Homology_Testing/bin/121010_lipidmapstools/docs/html/README.html]

LIPID MAPS Tools: README


|  |  |
| --- | --- |
|  | LIPID Metabolites And Pathways Strategy |

  

## Introduction

LIPID MAPS Tools package provides various command line Perl scripts to generate SD files containing structure and ontological data for various lipid categories.

## Installation

1. Add <YOUR LIPID MAPS TOOLS DIR>/bin to your PATH environment variable.

C shell example:

set path = "$path <YOUR LIPID MAPS TOOLS DIR>/bin"

Bourne shell example:

PATH = "$PATH:<YOUR LIPID MAPS TOOLS DIR>/bin"

Windows:

o Right click on "My Computer" icon and select properties. And Select "Environment Variables" button under Advanced tab.

o Select path under "System Variables" and click on Edit button. And system variable "path" to include LIPID MAPS TOOLS bin directory.

2. And you're all set to try out the command line scripts.

## Caveats

On some systems, command line scripts may need to be invoked using "perl -S <ScriptName.pl>" instead of "<ScriptName.pl>". Example:

% perl -S GLStrGen.pl

## Feedback

LIPID MAPS Webmaster

## Author

Manish Sud

## Contributor(s)

Eoin Fahy

COPYRIGHT

Copyright (C) 2006-2012. The Regents of the University of California. All Rights Reserved.

LICENSE

Modified BSD License
