# Supplementary material for: The LUX Score: A Metric for Lipidome Homology
Source: PLoS Comput Biol. 2015 Sep 22;11(9):e1004511. doi: 10.1371/journal.pcbi.1004511 (PMC4578897; doi:10.1371/journal.pcbi.1004511)
Supplement: S5 Dataset — Includes scripts, README files and data files for Figs 1, 2, 6, 7 and S6. (ZIP) [file pcbi.1004511.s009.zip › S5_Dataset/Lipidome_Homology_Testing/bin/121010_lipidmapstools/docs/html/ScriptsIndex.html]

LIPID MAPS Tools Documentation


|  |  |
| --- | --- |
|  | LIPID Metabolites And Pathways Strategy |

  

# LipidMAPSTools Documentation

## Table of Contents

- CLStrGen.pl
- FAStrGen.pl
- GLStrGen.pl
- GPStrGen.pl
- LMStrGen.pl
- SPStrGen.pl
- STStrGen.pl
