# Supplementary material for: The LUX Score: A Metric for Lipidome Homology
Source: PLoS Comput Biol. 2015 Sep 22;11(9):e1004511. doi: 10.1371/journal.pcbi.1004511 (PMC4578897; doi:10.1371/journal.pcbi.1004511)
Supplement: S5 Dataset — Includes scripts, README files and data files for Figs 1, 2, 6, 7 and S6. (ZIP) [file pcbi.1004511.s009.zip › S5_Dataset/Lipidome_Homology_Testing/bin/121010_lipidmapstools/docs/html/SPChainAbbrev.html]

LIPID MAPS Tools Documentation: SPChainAbbrev.pm


|  |  |
| --- | --- |
|  | LIPID Metabolites And Pathways Strategy |

  

|  |
| --- |
| PDF  PDFA4 |

## NAME

SPChainAbbrev - Methods for processing SP chain abbreviations

## SYNOPSIS

use SPChainAbbrev;

use SPChainAbbrev qw(:all);

## DESCRIPTION

SPChainAbbrev module provides these methods:

ChainAbbrevNameExists - Is it a supported chain abbreviation
  
 ExpandChainAbbrev - Expand wild cards in chain abbreviation
  
 GetChainAbbrevToNameMap - Get chain name
  
 GetChainLenAbbrevSupportedMap - Get reference to supported chain
abbreviations data
  
 GetChainLenAbbrevDbleBondGeometyDataMap - Get reference to supported chain
double bond geometry data
  
 GetSupportedChainLenList - Get supported chain lengths
  
 IsSphingosineChainAbbrev - Is it a sphingosine chain abbreviation
  
 IsSphingosineC18ChainAbbrev - Is it a sphingosine C18 abbreviation
  
 IsSphinganineC18ChainAbbrev - Is it a sphinganine C18 abbreviation

## METHODS

**ChainAbbrevNameExists**
:   $Status = ChainAbbrevNameExists($ChainAbbrev, $ChainType);

    Return 1 or 0 based on whether it's a supported chain name.

**ExpandChainAbbrev**
:   $AbbrevArrayRef = ExpandChainAbbrev($Abbrev);

    Return a reference to an array containing complete chain abbreviations. Wild card
    characters in chain abbreviation name are expanded to generate fully qualified
    chain abbreviations.

**GetChainAbbrevToNameMap**
:   $AbbrevNameHashRef = GetChainAbbrevToNameMap();

    Return a reference to hash with chain abbreviation/name as key/value pair.

**GetChainLenAbbrevSupportedMap**
:   $ChainLenHashRef = GetChainLenAbbrevSupportedMap();

    Return a reference to hash with supported chain length as hash key.

**GetChainLenAbbrevDbleBondGeometyDataMap**
:   $ChainLenDblBondHashRef = GetChainLenAbbrevDbleBondGeometyDataMap();

    Return a reference to hash containing information about chain length, number of
    double bonds and geometry of double bonds.

**GetSupportedChainLenList**
:   $ChainLengthListRef = GetSupportedChainLenList();

    Return a reference to a sorted list containing supported chain lengths.

**IsChainAbbrevOkay**
:   $Status = IsChainAbbrevOkay($ChainAbbrev);

    Return 1 or 0 based on whether chain abbreviation is valid.

**IsSphingosineChainAbbrev**
:   $Status = IsSphingosineChainAbbrev($ChainAbbrev);

    Return 1 or 0 based on whether it's a sphingosine chain abbreviation.

**IsSphingosineC18ChainAbbrev**
:   $Status = IsSphingosineC18ChainAbbrev($ChainAbbrev);

    Return 1 or 0 based on whether it's a sphingosine abbreviation with chain length
    of 18.

**IsSphinganineC18ChainAbbrev**
:   $Status = IsSphinganineC18ChainAbbrev($ChainAbbrev);

    Return 1 or 0 based on whether it's a sphinganine abbreviation with chain length
    of 18.

## AUTHOR

Manish Sud

## CONTRIBUTOR

Eoin Fahy

## SEE ALSO

LMAPSStr.pm, ChainStr.pm

## COPYRIGHT

Copyright (C) 2006-2012. The Regents of the University of California. All Rights Reserved.

## LICENSE

Modified BSD License
