# Supplementary material for: The LUX Score: A Metric for Lipidome Homology
Source: PLoS Comput Biol. 2015 Sep 22;11(9):e1004511. doi: 10.1371/journal.pcbi.1004511 (PMC4578897; doi:10.1371/journal.pcbi.1004511)
Supplement: S5 Dataset — Includes scripts, README files and data files for Figs 1, 2, 6, 7 and S6. (ZIP) [file pcbi.1004511.s009.zip › S5_Dataset/Lipidome_Homology_Testing/bin/121010_lipidmapstools/docs/html/SPStr.html]

LIPID MAPS Tools Documentation: SPStr.pm


|  |  |
| --- | --- |
|  | LIPID Metabolites And Pathways Strategy |

  

|  |
| --- |
| PDF  PDFA4 |

## NAME

SPStr - Sphingolipids (SP) structure generation methods

## SYNOPSIS

use SPStr;

use SPStr qw(:all);

## DESCRIPTION

SPStr module provides these methods:

GenerateCmpdOntologyData - Generate ontology data
  
 GenerateCmpdOntologySDDataLines - Generate ontology data lines for SD file
  
 GenerateSPChainStrData - Generate chain structure data
  
 GenerateSDFile - Generate SD file
  
 GetSPTemplatesData - Get templates data
  
 GetSPSupportedHeadGroupMap - Get supported headgroups data
  
 GetSPTemplateID - Get templates ID
  
 IsSPChainsAbbrevSupported - Is it a supported SP abbreviation
  
 ParseSPAbbrev - Parse SP abbreviation
  
 ProcessSPCmpdAbbrevs - Process SP abbreviation
  
 SetupSPCmpdAbbrevTemplateDataMap - Setup template structure data map
  
 ValidateSPAbbrev - Validate SP abbreviation

## METHODS

**GenerateCmpdOntologyData**
:   $DataHashRef = GenerateCmpdOntologyData($CmpDataRef);

    Return a reference to a hash containing ontology data with hash keys and values
    corresponding to property names and values.

**GenerateCmpdOntologySDDataLines**
:   $DataLinesArrayRef = GenerateCmpdOntologySDDataLines($CmpdDataRef);

    Return a reference to an array containing ontology data lines suitable for
    generate SD file data block.

**GenerateSPChainStrData**
:   ($AtomLinesArrayRef, $BondLinesArrayRef) =
    GenerateSPChainStrData($ChainType, $CmpdDataRef);

    Return array references containing atom and bond data lines for SD file. Appropriate atom
    and bond data lines are generated using chain type and abbreviation template data.

**GenerateSDFile**
:   GenerateSDFile($SDFileName, $CmdAbbrevsRef);

    Generate a SD file for compound abbreviations. Structure data for specified abbreviation
    is generated sequentially and written to SD file.

**GetSPTemplatesData**
:   $TemplatesDataRef = GetSPTemplatesData();

    Return a reference to a hash containing SP templates data

**GetSPSupportedHeadGroupMap**
:   $SupportedHeadGroupDataRef = GetSPSupportedHeadGroupMap();

    Return a reference to a hash containing supported head groups data.

**GetSPTemplateID**
:   $HeadGroupID = GetSPTemplateID($HeadGroupAbbrev, $ChainsAbbrev);

    Return a supported template ID for compound abbreviation.

**IsSPChainsAbbrevSupported**
:   $Status = IsSPChainsAbbrevSupported($Abbrev, $PrintWarning);

    Return 1 or 0 based on whether SP abbreviated is supported. For unsupported SP abbreviations,
    a warning is printed unless PrintWarning flag is set.

**ParseSPAbbrev**
:   ($HeadGroup, $ChainsAbbrev, $AbbrevModifier) =
    ParseSPAbbrev($Abbrev);

    Parse SP abbreviation and return these values: HeadGroup, ChainsAbbrev,
    AbbrevModifier.

**ProcessSPCmpdAbbrevs**
:   ProcessSPCmpdAbbrevs($CmpdAbbrevsRef, $AllowArbitraryChainAbbrev,
    $WriteSDFile, $SDFileName);

    Process specified SP abbreviations to generate structures and write them out either
    a SD file or simply report number of valid abbreviations.

**SetupSPCmpdAbbrevTemplateDataMap**
:   $AbbrevTemplateDataMapRef =
    SetupSPCmpdAbbrevTemplateDataMap($Abbrev);

    Return a reference to a hash containing template data for compound abbreviation. The
    template data is used to generate SD file for compound abbreviation.

**ValidateSPAbbrev**
:   $Status = ValidateSPAbbrev($Abbrev);

    Return 1 or 0 based on whether a SP abbreviation is valid.

## AUTHOR

Manish Sud

## CONTRIBUTOR

Eoin Fahy

## SEE ALSO

ChainStr.pm

## COPYRIGHT

Copyright (C) 2006-2012. The Regents of the University of California. All Rights Reserved.

## LICENSE

Modified BSD License
