# Supplementary material for: The LUX Score: A Metric for Lipidome Homology
Source: PLoS Comput Biol. 2015 Sep 22;11(9):e1004511. doi: 10.1371/journal.pcbi.1004511 (PMC4578897; doi:10.1371/journal.pcbi.1004511)
Supplement: S5 Dataset — Includes scripts, README files and data files for Figs 1, 2, 6, 7 and S6. (ZIP) [file pcbi.1004511.s009.zip › S5_Dataset/Lipidome_Homology_Testing/bin/121010_lipidmapstools/docs/html/SPStrGen.html]

LIPID MAPS Tools Documentation: SPStrGen.pl


|  |  |
| --- | --- |
|  | LIPID Metabolites And Pathways Strategy |

  

|  |
| --- |
| PDF  PDFA4 |

## NAME

SPStrGen.pl - Generate structures for Sphingophospholipids (SP)

## SYNOPSIS

SPStrGen.pl SPAbbrev|SPAbbrevFileName ...

SPStrGen.pl [**-c, --ChainAbbrevMode** *MostLikely | Arbitrary*]
[**-h, --help**] [**-m, --mode** *Abbrev | AbbrevFileName*]
[**-p, --ProcessMode** *WriteSDFile | CountOnly*] [**-o, --overwrite**]
[**-r, --root** rootname] [**-w, --workingdir** dirname] <arguments>...

## DESCRIPTION

Generate Sphingophospholipids (SP) structures using compound abbreviations specified on
a command line or in a CSV/TSV Text file. All the command line arguments represent either
compound abbreviations or file name containing abbreviations. Use mode option to control
the type of command line arguments.

A SD file, containing structures for all SP abbreviations along with ontological information, is
generated as an output.

## SUPPORTED ABBREVIATIONS

Current support for SP structure generation include these main classes and sub classes:

o Sphingoid bases

. Sphing-4-enines (Sphingosines)
  
 . Sphinganines
  
 . 4-Hydroxysphinganines (Phytosphingosines)
  
 . Sphingoid base homologs and variants
  
 . Sphingoid base 1-phosphates
  
 . Lysosphingomyelins and lysoglycosphingolipids

o Sphingoid bases

. Sphing-4-enines (Sphingosines)
  
 . Sphinganines
  
 . 4-Hydroxysphinganines (Phytosphingosines)
  
 . Sphingoid base homologs and variants
  
 . Sphingoid base 1-phosphates
  
 . Lysosphingomyelins and lysoglycosphingolipids
  
 . Sphingoid base analogs

o Ceramides

. N-acylsphingosines (ceramides)
  
 . N-acylsphinganines (dihydroceramides)
  
 . N-acyl-4-hydroxysphinganines (phytoceramides)
  
 . Ceramide 1-phosphates

o Phosphosphingolipids

. Ceramide phosphocholines (sphingomyelins)
  
 . Ceramide phosphoethanolamines
  
 . Ceramide phosphoinositols

o Neutral glycosphingolipids

. Simple Glc series (GlcCer, LacCer, etc)
  
 . GalNAcb1-3Gala1-4Galb1-4Glc- (Globo series)
  
 . GalNAcb1-4Galb1-4Glc- (Ganglio series)
  
 . Galb1-3GlcNAcb1-3Galb1-4Glc- (Lacto series)
  
 . Galb1-4GlcNAcb1-3Galb1-4Glc- (Neolacto series)
  
 . GalNAcb1-3Gala1-3Galb1-4Glc- (Isoglobo series)
  
 . GlcNAcb1-2Mana1-3Manb1-4Glc- (Mollu series)
  
 . GalNAcb1-4GlcNAcb1-3Manb1-4Glc- (Arthro series)
  
 . Gal- (Gala series)

o Acidic glycosphingolipids

. Gangliosides

## OPTIONS

**-c, --ChainAbbrevMode** *MostLikely|Arbitrary*
:   Specify what types of acyl chain abbreviations are allowed during processing of complete
    abbreviations: allow most likely chain abbreviations containing specific double bond geometry
    specifications; allow any acyl chain abbreviation with valid chain length and double bond
    geometry specificatios. Possible values: *MostLikely or Arbitrary*. Default value: *MostLikely*.

    *Arbitrary* value of **-c, --ChainAbbrevMode** option is not allowed during processing of
    abbreviations containing wild cards.

    During *MostLikely* value of **-c, --ChainAbbrevMode** option, only the most likely acyl chain
    abbreviations specified in ChainAbbrev.pm module are allowed. However, during *Arbitrary* value
    of **-c, --ChainAbbrevMode** option, any acyl chain abbreviations with valid chain length and
    double bond geometry can be specified. The current release of lipidmapstools support chain
    lengths from 2 to 50 as specified in ChainAbbev.pm module.

    In addition to double bond geometry specifications, valid substituents can be specified for in the acyl
    chain abbreviations.

**-h, --help**
:   Print this help message

**-m, --mode** *Abbrev|AbbrevFileName*
:   Controls interpretation of command line arguments. Two different methods are provided:
    specify compound abbreviations or a file name containing compound abbreviations. Possible
    values: *Abbrev or AbbrevFileName*. Default: *Abbrev*

    In *AbbrevFileName* mode, a single line in CSV/TSV files can contain multiple compound
    abbreviations. The file extension determines delimiter used to process data lines: comma for
    CSV and tab for TSV. For files with TXT extension, only one compound abbreviation per line
    is allowed.

    Wild card character, \*, is also supported in compound abbreviations.

    Examples:

    Specific structures: Cer(d18:0/0:0) Cer(d18:1(4E)/0:0)
    Cer(d19:1(4E)/24:4(5Z,8Z,11Z,14Z))
      
     Specific structures: SM(d18:0/16:0) SM(d19:0/24:1(15Z))
      
     Specific possibilities: Cer(\*/0:0) Cer(d18:1(4E)/\*)
      
     All possibilites: \*(\*:\*/\*:\*) or \*(\*/\*)

    With wild card character, +/- can also be used for chain lengths to indicate even and odd lengths at
    sn1/sn2/sn3 positions; additionally > and < qualifiers are also allowed to specify length
    requirements. Examples:

    Odd and even number chains at sn1 and sn2: \*(\*-:\*/\*+:\*)
      
     Odd and even number chains at sn1 and sn2 with length longer than 18
    and 22: \*(\*->18:\*/\*+>22:\*)

**-p, --ProcessMode** *WriteSDFile|CountOnly*
:   Specify how abbreviations are processed: generate structures for specified abbreviations along
    with generating a SD file or just count the number of structures corresponding to specified
    abbreviations without generating any SD file. Possible values: *WriteSDFile or CountOnly*.
    Default: *WriteSDFile*.

    It can take substantial amount of time for generating all the structures and writing out a SD file
    for abbreviations containing wild cards. *CountOnly* value of **--ProcessMode** option can
    be used to get a quick count of number of structures to be generated without writing out any
    SD file.

**-o, --overwrite**
:   Overwrite existing files

**-r, --root** *rootname*
:   New file name is generated using the root: <Root>.sdf. Default for new file names: SPAbbrev.sdf,
    <AbbrevFilenName>.sdf, or <FirstAbbrevFileName>1To<Count>.sdf.

**-w, --workingdir** *dirname*
:   Location of working directory. Default: current directory

## EXAMPLES

On some systems, command line scripts may need to be invoked using
*perl -s SPStrGen.pl*; however, all the examples assume direct invocation
of command line script works.

To generate a SPStructures.sdf file containing a structure specified
by a command line SP abbreviation, type:

% SPStrGen.pl -r SPStructures -o "Cer(d18:0/0:0)"

To generate a SPStructures.sdf file containing structures specified
by a command line SP abbreviations, type:

% SPStrGen.pl -r SPStructures -o "SM(d18:0/16:0)" "SM(d19:0/24:1(15Z))"

To enumerate all possible SP structures and generate a SPStructures.sdf
file, type:

% SPStrGen.pl -r SPStructures -o "\*(\*/\*)"

or

% SPStrGen.pl -r SPStructures -o "\*(\*:\*/\*:\*)"

## AUTHOR

Manish Sud

## CONTRIBUTOR

Eoin Fahy

## SEE ALSO

CLStrGen.pl, FAStrGen.pl, GLStrGen.pl, GPStrGen.pl, STStrGen.pl

## COPYRIGHT

Copyright (C) 2006-2012. The Regents of the University of California. All Rights Reserved.

## LICENSE

Modified BSD License
