# Supplementary material for: The LUX Score: A Metric for Lipidome Homology
Source: PLoS Comput Biol. 2015 Sep 22;11(9):e1004511. doi: 10.1371/journal.pcbi.1004511 (PMC4578897; doi:10.1371/journal.pcbi.1004511)
Supplement: S5 Dataset — Includes scripts, README files and data files for Figs 1, 2, 6, 7 and S6. (ZIP) [file pcbi.1004511.s009.zip › S5_Dataset/Lipidome_Homology_Testing/bin/121010_lipidmapstools/docs/html/STStr.html]

LIPID MAPS Tools Documentation: STStr.pm


|  |  |
| --- | --- |
|  | LIPID Metabolites And Pathways Strategy |

  

|  |
| --- |
| PDF  PDFA4 |

## NAME

STStr - Sterol (ST) structure generation methods

## SYNOPSIS

use STStr;

use STStr qw(:all);

## DESCRIPTION

STStr module provides these methods:

ExpandSTCmpdAbbrevs - Expand ST abbreviation
  
 GenerateCmpdOntologyData - Generate ontology data
  
 GenerateCmpdOntologySDDataLines - Generate ontology data lines for
SD file
  
 GenerateSTStrData - Generate structure data
  
 GenerateSDFile - Generate SD file
  
 IsSTAbbrevSupported - Is it a supported ST abbreviation
  
 IsSTSubstituentsNameSupported - Is it a supported ST substituent name
  
 IsSTDoubleBondsAbbrevOkay - Is it a valid ST double bond abbreviation
  
 IsSTSubstituentsAbbrevOkay - Is it a valid ST substituent abbreviation
  
 IsWildCardInSTAbbrev - Does ST abbreviatio contains a wild card
  
 ParseSTAbrev - Parse ST abbreviation
  
 ParseSTDoubleBondAbbrev - Parse ST double bond abbreviation
  
 ParseSTSubstituentAbbrev - Parse ST substituent abbreviation
  
 SetupSTCmpdAbbrevTemplateDataMap - Setup template structure data map
  
 ValidateSTAbbrev - Validate ST abbreviation

## METHODS

**ExpandSTCmpdAbbrevs**
:   $ExpandedAbbrevArrayRef = ExpandSTCmpdAbbrevs($CmpdAbbrev);

    Return a reference to an array containing complete ST abbreviations. Wild card
    characters in ST abbreviation name are expanded to generate fully qualified
    ST abbreviations.

**GenerateCmpdOntologyData**
:   $DataHashRef = GenerateCmpdOntologyData($CmpdDataRef);

    Return a reference to a hash containing ontology data with hash keys and values
    corresponding to property names and values.

**GenerateCmpdOntologySDDataLines**
:   $DataLinesArrayRef =
    GenerateCmpdOntologySDDataLines($CmpDataRef);

    Return a reference to an array containing ontology data lines suitable for
    generate SD file data block.

**GenerateSTStrData**
:   ($AtomLinesArrayRef, $BondLinesArrayRef) =
    GenerateSTStrData($CmpdDataRef);

    Return array references containing atom and bond data lines for SD file. Appropriate atom
    and bond data lines are generated using abbreviation template data.

**GenerateSDFile**
:   GenerateSDFile($SDFileName, $CmdAbbrevsRef);

    Generate a SD file for compound abbreviations. Structure data for specified abbreviation
    is generated sequentially and written to SD file.

**IsSTAbbrevSupported**
:   $Status = IsSTAbbrevSupported($Abbrev);

    Return 1 or 0 based on whether ST abbreviation is supported.

**IsSTSubstituentsNameSupported**
:   $Status = IsSTSubstituentsNameSupported($SubstituentAbbrev);

    Return 1 or 0 based on whether ST substituent abbreviation is supported.

**IsSTDoubleBondsAbbrevOkay**
:   $Status = IsSTDoubleBondsAbbrevOkay($STAbbrev, $STType,
    $SubstituentsAbbrev, $DoubleBondsAbbrev);

    Return 1 or 0 based on whether ST double bond abbreviation is valid.

**IsSTSubstituentsAbbrevOkay**
:   $Status = IsSTSubstituentsAbbrevOkay($STAbbrev, $STType,
    $SubstituentsAbbrev, $DoubleBondsAbbrev);

    Return 1 or 0 based on whether ST substituent abbreviation is valid.

**IsWildCardInSTAbbrev**
:   $Status = IsSTAbbrevSupported($Abbrev);

    Return 1 or 0 based on whether ST abbreviation contains wild card.

**ParseSTAbbrev**
:   ($STType, $SubstituentsAbbrev, $DoubleBondsAbbrev) =
    ParseSTAbrev($Abbrev);

    Parse ST abbreviation and return these values: STType, SubstituentsAbbrev, and
    DoubleBondsAbbrev.

**ParseSTDoubleBondAbbrev**
:   ($BondPos1, $BondPos1) = ParseSTDoubleBondAbbrev($Abbrev);

    Parse ST double bond abbreviation and return these values: BondPos1 and BondPos2.

**ParseSTSubstituentAbbrev**
:   ($SubstituentPos, $SubstituentAbbrev, $StereoChemistry) =
    ParseSTSubstituentAbbrev($Abbrev);

    Parse ST substituents abbreviation and return these values: SubstituentPos, SubstituentAbbrev,
    and SubstituentStereoChemistry.

**SetupSTCmpdAbbrevTemplateDataMap**
:   $AbbrevTemplateDataMapRef =
    SetupSTCmpdAbbrevTemplateDataMap($Abbrev);

    Return a reference to a hash containing template data for compound abbreviation. The
    template data is used to generate SD file for compound abbreviation.

**ValidateSTAbbrev**
:   $Status = ValidateSTAbbrev($Abbrev);

    Return 1 or 0 based on whether a ST abbreviation is valid.

## AUTHOR

Manish Sud

## CONTRIBUTOR

Eoin Fahy

## SEE ALSO

ChainStr.pm, LMAPSStr.pm

## COPYRIGHT

Copyright (C) 2006-2012. The Regents of the University of California. All Rights Reserved.

## LICENSE

Modified BSD License
