# Supplementary material for: The LUX Score: A Metric for Lipidome Homology
Source: PLoS Comput Biol. 2015 Sep 22;11(9):e1004511. doi: 10.1371/journal.pcbi.1004511 (PMC4578897; doi:10.1371/journal.pcbi.1004511)
Supplement: S5 Dataset — Includes scripts, README files and data files for Figs 1, 2, 6, 7 and S6. (ZIP) [file pcbi.1004511.s009.zip › S5_Dataset/Lipidome_Homology_Testing/bin/121010_lipidmapstools/docs/html/STStrGen.html]

LIPID MAPS Tools Documentation: STStrGen.pl


|  |  |
| --- | --- |
|  | LIPID Metabolites And Pathways Strategy |

  

|  |
| --- |
| PDF  PDFA4 |

## NAME

STStrGen.pl - Generate structures for Sterols (ST)

## SYNOPSIS

STStrGen.pl FAAbbrev|FAAbbrevFileName ...

STStrGen.pl [**-h, --help**] [**-o, --overwrite**] [**-r, --root** rootname]
[**-w, --workingdir** dirname] <arguments>...

## DESCRIPTION

Generate Strerol (ST) structures using compound abbreviations specified on
a command line or in a CSV/TSV Text file. All the command line arguments represent either
compound abbreviations or file name containing abbreviations. Use mode option to control
the type of command line arguments.

A SD file, containing structures for all SP abbreviations along with ontological information, is
generated as an output.

## SUPPORTED ABBREVIATIONS

Current support for ST structure generation include these main classes and sub classes:

o Sterols

. Cholesterol and derivatives
  
 . Ergosterols and C24-methyl derivatives
  
 . Stigmasterols and C24-ethyl derivatives

## OPTIONS

**-h, --help**
:   Print this help message

**-m, --mode** *Abbrev|AbbrevFileName*
:   Controls interpretation of command line arguments. Two different methods are provided:
    specify compound abbreviations or a file name containing compound abbreviations. Possible
    values: *Abbrev or AbbrevFileName*. Default: *Abbrev*

    In *AbbrevFileName* mode, a single line in CSV/TSV files can contain multiple compound
    abbreviations. The file extension determines delimiter used to process data lines: comma for
    CSV and tab for TSV. For files with TXT extension, only one compound abbreviation per line
    is allowed.

    Examples:

    cholesterol: "CHOLESTANE(3,b,OH:5(6))"
      
     5alpha-cholestane: "CHOLESTANE(5,a,H)"
      
     25-hydroxy-cholesterol: "CHOLESTANE(3,b,OH/25,,OH:5(6))"
      
     5,6beta-epoxy-cholesterol: "CHOLESTANE(3,b,OH/5,b,Ep)"
      
     cholestenone: "CHOLESTANE(3,,Ke:4)"

    ergost-5-en-3beta-ol: "ERGOSTANE(3,b,OH:5)"

    campest-5-en-3beta-ol: "CAMPESTANE(3,b,OH:5)"

    stigmast-5,25-dien-3beta-ol: "STIGMASTANE(3,b,OH:5/25(26))"

**-o, --overwrite**
:   Overwrite existing files

**-r, --root** *rootname*
:   New file name is generated using the root: <Root>.sdf. Default for new file names: STAbbrev.sdf,
    <AbbrevFilenName>.sdf, or <FirstAbbrevFileName>1To<Count>.sdf.

**-w, --workingdir** *dirname*
:   Location of working directory. Default: current directory

## EXAMPLES

On some systems, command line scripts may need to be invoked using
*perl -s STStrGen.pl*; however, all the examples assume direct invocation
of command line script works.

To generate a STStructures.sdf file containing a structure specified
by a command line abbreviation for cholesterol, type:

% STStrGen.pl -r STStructures -o "CHOLESTANE(3,b,OH:5(6))"

To generate a STStructures.sdf file containing a structure specified
by a command line abbreviation for 5alpha-cholestane, type:

% STStrGen.pl -r STStructures -o "CHOLESTANE(5,a,H)"

To generate a STStructures.sdf file containing a structure specified
by a command line abbreviation for 25-hydroxy-cholesterol, type:

% STStrGen.pl -r STStructures -o "CHOLESTANE(3,b,OH/25,,OH:5(6))"

To generate a STStructures.sdf file containing a structure specified
by a command line abbreviation for 24S-hydroxy-cholesterol, type:

% STStrGen.pl -r STStructures -o "CHOLESTANE(3,b,OH/24,a,OH:5(6))"

To generate a STStructures.sdf file containing a structure specified
by a command line abbreviation for 5,6beta-epoxy-cholesterol, type:

% STStrGen.pl -r STStructures -o "CHOLESTANE(3,b,OH/5,b,Ep)"

To generate a STStructures.sdf file containing a structure specified
by a command line abbreviation for cholestenone, type:

% STStrGen.pl -r STStructures -o "CHOLESTANE(3,,Ke:4)"

To generate a STStructures.sdf file containing a structure specified
by a command line abbreviation for desmostero;, type:

% STStrGen.pl -r STStructures -o "CHOLESTANE(3,b,OH:5/24)"

## AUTHOR

Manish Sud

## CONTRIBUTOR

Eoin Fahy

## SEE ALSO

CLStrGen.pl, FAStrGen.pl, GLStrGen.pl, GPStrGen.pl, SPStrGen.pl

## COPYRIGHT

Copyright (C) 2006-2012. The Regents of the University of California. All Rights Reserved.

## LICENSE

Modified BSD License
