# Supplementary material for: The LUX Score: A Metric for Lipidome Homology
Source: PLoS Comput Biol. 2015 Sep 22;11(9):e1004511. doi: 10.1371/journal.pcbi.1004511 (PMC4578897; doi:10.1371/journal.pcbi.1004511)
Supplement: S5 Dataset — Includes scripts, README files and data files for Figs 1, 2, 6, 7 and S6. (ZIP) [file pcbi.1004511.s009.zip › S5_Dataset/Lipidome_Homology_Testing/bin/121010_lipidmapstools/docs/pdfa4/CLStr.pdf]

NAME

CLStr - Cardiolipins (CL) structure generation methods

SYNOPSIS

use CLStr;

use CLStr qw(:all);

DESCRIPTION

CLStr module provides these methods:

```
GenerateCmpdOntologyData - Generate ontology data
GenerateCmpdOntologySDDataLines - Generate ontology data lines for
                                  SD file
GenerateCLChainStrData - Generate chain structure data
GenerateSDFile - Generate SD file
GetCLTemplatesData - Get templates data
GetCLSupportedHeadGroupMap - Get supported headgroups data
GetCLTemplateID - Get templates ID
IsCLChainsAbbrevSupported - Is it a supported CL abbreviation
ParseCLAbbrev - Parse CL abbreviation
ProcessCLCmpdAbbrevs - Process CL abbreviation
SetupCLCmpdAbbrevTemplateDataMap - Setup template structure data map
ValidateCLAbbrev - Validate CL abbreviation
```

METHODS

GenerateCmpdOntologyData

```
$DataHashRef = GenerateCmpdOntologyData($CmpDataRef);
```

Return a reference to a hash containing ontology data with hash keys and values corresponding to property names and values.

GenerateCmpdOntologySDDataLines

```
$DataLinesArrayRef =
   GenerateCmpdOntologySDDataLines($CmpdDataRef);
```

Return a reference to an array containing ontology data lines suitable for generate SD file data block.

GenerateCLChainStrData

```
($AtomLinesArrayRef, $BondLinesArrayRef) =
   GenerateCLChainStrData($ChainType, $CmpdDataRef);
```

Return array references containing atom and bond data lines for SD file. Appropriate atom and bond data lines are generated using chain type and abbreviation template data.

GenerateSDFile

```
GenerateSDFile($SDFileName, $CmdAbbrevsRef);
```

Generate a SD file for compound abbreviations. Structure data for specified abbreviation is generated sequentially and written to SD file.

GetCLTemplatesData

```
$TemplatesDataRef = GetCLTemplatesData();
```

Return a reference to a hash containing CL templates data

GetCLSupportedHeadGroupMap

```
$SupportedHeadGroupDataRef = GetCLSupportedHeadGroupMap();
```

Return a reference to a hash containing supported head groups data.

GetCLTemplateID

```
$HeadGroupID = GetCLTemplateID($HeadGroupAbbrev, $ChainsAbbrev);
```

Return a supported template ID for compound abbreviation.

IsCLChainsAbbrevSupported

```
$Status = IsCLChainsAbbrevSupported($Abbrev, $PrintWarning);
```

Return 1 or 0 based on whether CL abbreviated is supported. For unsupported CL abbreviations, a warning is printed unless PrintWarning flag is set.

ParseCLAbbrev

```
($HeadGroup, $ChainsAbbrev, $AbbrevModifier) =
   ParseCLAbbrev($Abbrev);
```

Parse CL abbreviation and return these values: HeadGroup, ChainsAbbrev, AbbrevModifier.

ProcessCLCmpdAbbrevs

```
ProcessCLCmpdAbbrevs($CmpdAbbrevsRef, $AllowArbitraryChainAbbrev,
                     $WriteSDFile, $SDFileName);
```

Process specified CL abbreviations to generate structures and write them out either a SD file or simply report number of valid abbreviations.

SetupCLCmpdAbbrevTemplateDataMap

```
$AbbrevTemplateDataMapRef =
   SetupCLCmpdAbbrevTemplateDataMap($Abbrev);
```

Return a reference to a hash containing template data for compound abbreviation. The template data is used to generate SD file for compound abbreviation.

ValidateCLAbbrev

```
$Status = ValidateCLAbbrev($Abbrev);
```

Return 1 or 0 based on whether a CL abbreviation is valid.

AUTHOR

Manish Sud

CONTRIBUTOR

Eoin Fahy

SEE ALSO

ChainAbbrev.pm, ChainStr.pm, LMAPSStr.pm

COPYRIGHT

Copyright (C) 2006-2012. The Regents of the University of California. All Rights Reserved.

LICENSE

Modified BSD License
