# Supplementary material for: The LUX Score: A Metric for Lipidome Homology
Source: PLoS Comput Biol. 2015 Sep 22;11(9):e1004511. doi: 10.1371/journal.pcbi.1004511 (PMC4578897; doi:10.1371/journal.pcbi.1004511)
Supplement: S5 Dataset — Includes scripts, README files and data files for Figs 1, 2, 6, 7 and S6. (ZIP) [file pcbi.1004511.s009.zip › S5_Dataset/Lipidome_Homology_Testing/bin/121010_lipidmapstools/docs/pdfa4/FAStr.pdf]

NAME

FAStr - Fatty Acyls (FA) structure generation methods

SYNOPSIS

use FAStr;

use FAStr qw(:all);

DESCRIPTION

FAStr module provides these methods:

```
    GenerateCmpdOntologyData - Generate ontology data
    GenerateCmpdOntologySDDataLines - Generate ontology data lines for SD
file
    GenerateFAChainStrData - Generate chain structure data
    GenerateSDFile - Generate SD file
    IsFAAbbrevSupported - Is it a supported FA abbreviation
    ParseFAAbrev - Parse FA abbreviation
    SetupFACmpdAbbrevTemplateDataMap - Setup template structure data map
    ValidateFAAbbrev - Validate FA abbreviation
```

METHODS

GenerateCmpdOntologyData

```
    $DataHashRef = GenerateCmpdOntologyData($CmpdDataRef);
```

Return a reference to a hash containing ontology data with hash keys and values corresponding to property names and values.

GenerateCmpdOntologySDDataLines

```
    $DataLinesArrayRef =
GenerateCmpdOntologySDDataLines($CmpDataRef);
```

Return a reference to an array containing ontology data lines suitable for generate SD file data block.

GenerateFAChainStrData

```
    ($AtomLinesArrayRef, $BondLinesArrayRef) =
       GenerateFAChainStrData($ChainType, $CmpdDataRef);
```

Return array references containing atom and bond data lines for SD file. Appropriate atom and bond data lines are generated using chain type and abbreviation template data.

GenerateSDFile

```
    GenerateSDFile($SDFileName, $CmdAbbrevsRef);
```

Generate a SD file for compound abbreviations. Structure data for specified abbreviation is generated sequentially and written to SD file.

IsFAAbbrevSupported

```
    $Status = IsFAAbbrevSupported($Abbrev, $PrintWarning);
```

Return 1 or 0 based on whether FA abbreviated is supported. For unsupported FA abbreviations, a warning is printed unless PrintWarning flag is set.

ParseFAAbrev

```
    ($ChainAbbrev, $AbbrevModifier) = ParseFAAbrev($Abbrev);
```

Parse FA abbreviation and return these values: ChainsAbbrev and AbbrevModifier.

ProcessFACmpdAbbrevs

```
ProcessFACmpdAbbrevs($CmpdAbbrevsRef, $WriteSDFile, $SDFileName);
```

Process specified FA abbreviations to generate structures and write them out either a SD file or simply report number of valid abbreviations.

SetupFACmpdAbbrevTemplateDataMap

```
$AbbrevTemplateDataMapRef =
   SetupFACmpdAbbrevTemplateDataMap($Abbrev);
```

Return a reference to a hash containing template data for compound abbreviation. The template data is used to generate SD file for compound abbreviation.

ValidateFAAbbrev

```
$Status = ValidateFAAbbrev($Abbrev);
```

Return 1 or 0 based on whether a FA abbreviation is valid.

AUTHOR

Manish Sud

CONTRIBUTOR

Eoin Fahy

SEE ALSO

ChainAbbrev.pm, ChainStr.pm, LMAPSStr.pm

COPYRIGHT

Copyright (C) 2006-2012. The Regents of the University of California. All Rights Reserved.

LICENSE

Modified BSD License
