# Supplementary material for: The LUX Score: A Metric for Lipidome Homology
Source: PLoS Comput Biol. 2015 Sep 22;11(9):e1004511. doi: 10.1371/journal.pcbi.1004511 (PMC4578897; doi:10.1371/journal.pcbi.1004511)
Supplement: S5 Dataset — Includes scripts, README files and data files for Figs 1, 2, 6, 7 and S6. (ZIP) [file pcbi.1004511.s009.zip › S5_Dataset/Lipidome_Homology_Testing/bin/121010_lipidmapstools/docs/pdfa4/FAStrGen.pdf]

NAME

FAStrGen.pl - Generate structures for Fatty Acyls (FA)

SYNOPSIS

FAStrGen.pl FAAbbrev|FAAbbrevFileName ...

FAStrGen.pl [**-h, --help**] [**-m, --mode** *Abbrev* **|** *AbbrevFileName*] [**-p, --ProcessMode** *WriteSDFile* **|** *CountOnly*] [**-o, --overwrite**] [**-r, --root** rootname] [**-w, --workingdir** dirname] <arguments>...

DESCRIPTION

Generate Fatty Acyls (FA) structures using compound abbreviations specified on a command line or in a CSV/TSV Text file. All the command line arguments represent either compound abbreviations or file name containing abbreviations. Use mode option to control the type of command line arguments.

A SD file, containing structures for all SP abbreviations along with ontological information, is generated as an output.

SUPPORTED ABBREVIATIONS

Current support for FA structure generation include these main classes and sub classes:

o Fatty Acids and Conjugates

```
. Straight chain fatty acids
. Methyl branched fatty acids
. Unsaturated fatty acids
. Hydroperoxy fatty acids
. Hydroxy fatty acids
. Oxo fatty acids
. Epoxy fatty acids
. Methoxy fatty acids
. Halogenated fatty acids
. Amino fatty acids
. Cyano fatty acids
. Nitro fatty acids
. Thia fatty acids
```

o Eicosanoids

```
. Prostaglandins
```

o Fatty alcohols

o Fatty aldehydes

o Fatty amides

```
. Primary amides
```

OPTIONS

-h, --help

Print this help message

-m, --mode *Abbrev|AbbrevFileName*

Controls interpretation of command line arguments. Two different methods are provided: specify compound abbreviations or a file name containing compound abbreviations. Possible values: *Abbrev or AbbrevFileName*. Default: *Abbrev*

In *AbbrevFileName* mode, a single line in CSV/TSV files can contain multiple compound abbreviations. The file extension determines delimiter used to process data lines: comma for CSV and tab for TSV. For files with TXT extension, only one compound abbreviation per line is allowed.

Wild card character, *, is also supported in compound abbreviations to generate straight chain and unsaturated fatty acids.

Examples:

```
    Specific structures: "18:0" "20:4(5Z,8Z,11Z,14Z)"
                         "28:1(12Z)(2Me,4Me,6Me)"
                         "18:3(6Z,9Z,11E)(13OOH[S])"
                         "18:2(9E,11E)(13OH)"
                         "18:1(10E)(9Ke,10Ep)"
                         "16:1(5Z)(2OMe)" "7:1(2Z)(3Br)"
                         "18:2(9Z,12Z)(10NO2)" "16:2(10E,12Z)(1OH)"
                         "6:0(1CHO)" "12:0(1NH2)"

"20:2(5Z,13E)(9OH[S],11OH[R],15OH[S]){8a,12b}"
    All possibilites: *:* or *
```

With wild card character, +/- can also be used for chain lengths to indicate even and odd lengths; additionally > and < qualifiers are also allowed to specify length requirements. Examples:

```
    Odd number chains: "*-:*"
    Even number chains: "*+:*"
    Odd number chains with chain length longer than 18: "*->18:*"
    Even number chains with chain length longer than 14: "*+>14:*"
```

-o, --overwrite

Overwrite existing files

-r, --root *rootname*

New file name is generated using the root: <Root>.sdf. Default for new file names: FAAbbrev.sdf, <AbbrevFilenName>.sdf, or <FirstAbbrevFileName>1To<Count>.sdf.

-p, --ProcessMode *WriteSDFile|CountOnly*

Specify how abbreviations are processed: generate structures for specified abbreviations along with generating a SD file or just count the number of structures corresponding to specified abbreviations without generating any SD file. Possible values: *WriteSDFile or CountOnly*. Default: *WriteSDFile*.

It can take substantial amount of time for generating all the structures and writing out a SD file for abbreviations containing wild cards. *CountOnly* value of **--ProcessMode** option can be used to get a quick count of number of structures to be generated without writing out any SD file.

-w, --workingdir *dirname*

Location of working directory. Default: current directory

EXAMPLES

On some systems, command line scripts may need to be invoked using *perl -s FAStrGen.pl*; however, all the examples assume direct invocation of command line script works.

To generate a FAStructures.sdf file containing a structure specified by a command line FA abbreviation for straight chain fatty acids, type:

```
% FAStrGen.pl -r FAStructures -o "18:0" "9:0"
```

To generate a FAStructures.sdf file containing a structure specified by a command line FA abbreviation for methyl branched fatty acids, type:

```
% FAStrGen.pl -r FAStructures -o "8:0(6Me)" "18:1(6Z)(17Me)"
"28:1(12Z)(2Me,4Me,6Me)"
```

To generate a FAStructures.sdf file containing a structure specified by a command line FA abbreviation for unsaturated fatty acids, type:

```
% FAStrGen.pl -r FAStructures -o "20:4(5Z,8Z,11Z,14Z)" "8:1(5E)"
```

To generate a FAStructures.sdf file containing a structure specified by a command line FA abbreviation for hydroperoxy fatty acids, type:

```
% FAStrGen.pl -r FAStructures -o "18:2(9E,11E)(13OOH)"
"18:3(6Z,9Z,11E)(13OOH[S])"
```

To generate a FAStructures.sdf file containing a structure specified by a command line FA abbreviation for hydroxy fatty acids, type:

```
% FAStrGen.pl -r FAStructures -o "10:0(10OH)" "15:0(2OH,15OH)"
 "18:2(9E,11E)(13OH)" "4:0(3OH[R])"
```

To generate a FAStructures.sdf file containing a structure specified by a command line FA abbreviation for oxo fatty acids, type:

```
% FAStrGen.pl -r FAStructures -o "10:0(2Ke)" "18:1(10E)(9Ke,10Ep)"
```

To generate a FAStructures.sdf file containing a structure specified by a command line FA abbreviation for epoxy fatty acids, type:

```
% FAStrGen.pl -r FAStructures -o "18:0(6Ep)"
```

To generate a FAStructures.sdf file containing a structure specified by a command line FA abbreviation for methoxy fatty acids, type:

```
% FAStrGen.pl -r FAStructures -o "18:1(9E)(12OH,13OH,11OMe)"
"16:1(5Z)(2OMe)"
```

To generate a FAStructures.sdf file containing a structure specified by a command line FA abbreviation for halogenated fatty acids, type:

```
% FAStrGen.pl -r FAStructures -o "7:1(2Z)(3Br)" "26:2(5Z,9Z)(2Br)"
```

To generate a FAStructures.sdf file containing a structure specified by a command line FA abbreviation for amino fatty acids, type:

```
% FAStrGen.pl -r FAStructures -o "13:0(2NH2[S])" "4:0(2NH2,4CN)"
```

To generate a FAStructures.sdf file containing a structure specified by a command line FA abbreviation for Cyano fatty acids, type:

```
% FAStrGen.pl -r FAStructures -o "4:0(4CN)"
```

To generate a FAStructures.sdf file containing a structure specified by a command line FA abbreviation for nitro fatty acids, type:

```
% FAStrGen.pl -r FAStructures -o "18:2(9Z,12Z)(10NO2)"
```

To generate a FAStructures.sdf file containing a structure specified by a command line FA abbreviation for prostaglanins, type:

```
% FAStrGen.pl -r FAStructures -o "20:2(5Z,13E)(9OH[S],11OH[R],
15OH[S]){8a,12b}"
```

To generate a FAStructures.sdf file containing a structure specified by a command line FA abbreviation for fatty alcohols, type:

```
% FAStrGen.pl -r FAStructures -o "26:0(1OH)" "16:2(10E,12Z)(1OH)"
"11:0(1OH,2Me,2Me,9Me,9Me,10OH)"
```

To generate a FAStructures.sdf file containing a structure specified by a command line FA abbreviation for fatty aldehydes, type:

```
% FAStrGen.pl -r FAStructures -o "6:0(1CHO)" "16:2(2E,4E)(1CHO,6OH)"
```

To generate a FAStructures.sdf file containing a structure specified by a command line FA abbreviation for primary amides, type:

```
% FAStrGen.pl -r FAStructures -o "12:0(1NH2)"
```

To enumerate straight chain and unsaturated fatty acids with commonly occuring chain lengths and generate FAStructures.sdf file, type:

```
% FAStrGen.pl -r FAStructures -o "*"
```

or

```
% FAStrGen.pl -r SPStructures -o "*:*"
```

AUTHOR

Manish Sud

CONTRIBUTOR

Eoin Fahy

SEE ALSO

CLStrGen.pl, GLStrGen.pl, GPStrGen.pl, SPStrGen.pl, STStrGen.pl

COPYRIGHT

Copyright (C) 2006-2012. The Regents of the University of California. All Rights Reserved.

LICENSE

Modified BSD License
