# Supplementary material for: The LUX Score: A Metric for Lipidome Homology
Source: PLoS Comput Biol. 2015 Sep 22;11(9):e1004511. doi: 10.1371/journal.pcbi.1004511 (PMC4578897; doi:10.1371/journal.pcbi.1004511)
Supplement: S5 Dataset — Includes scripts, README files and data files for Figs 1, 2, 6, 7 and S6. (ZIP) [file pcbi.1004511.s009.zip › S5_Dataset/Lipidome_Homology_Testing/bin/121010_lipidmapstools/docs/pdfa4/GPStrGen.pdf]

NAME

GPStrGen.pl - Generate structures for Glycerophospholipids (GP)

SYNOPSIS

GPStrGen.pl GPAbbrev|GPAbbrevFileName ...

GPStrGen.pl [**-c, --ChainAbbrevMode** *MostLikely* **|** *Arbitrary*] [**-h, --help**] [**-m, --mode** *Abbrev* **|** *AbbrevFileName*] [**-p, --ProcessMode** *WriteSDFile* **|** *CountOnly*] [**-o, --overwrite**] [**-r, --root** rootname] [**-w, --workingdir** dirname] <arguments>...

DESCRIPTION

Generate Glyceriphospholipids (GP) structures using compound abbreviations specified on a command line or in a CSV/TSV Text file. All the command line arguments represent either compound abbreviations or file name containing abbreviations. Use mode option to control the type of command line arguments.

A SD file, containing structures for all GP abbreviations along with ontological information, is generated as an output.

SUPPORTED ABBREVIATIONS

Current support for GP structure generation include these main classes and sub classes:

o Glycerophosphocholines (PC)

```
. Diacylglycerophosphocholines
. 1-alkyl,2-acylglycerophosphocholines
. 1Z-alkenyl,2-acylglycerophosphocholines
. Dialkylglycerophosphocholines
. Monoacylglycerophosphocholines
. 1-alkyl glycerophosphocholines
. 1Z-alkenylglycerophosphocholines
```

o Glycerophosphoethanolamines (PE)

```
. Diacylglycerophosphoethanolamines
. 1-alkyl,2-acylglycerophosphoethanolamines
. 1Z-alkenyl,2-acylglycerophosphoethanolamines
. Dialkylglycerophosphoethanolamines
. Monoacylglycerophosphoethanolamines
. 1-alkyl glycerophosphoethanolamines
. 1Z-alkenylglycerophosphoethanolamines
```

o Glycerophosphoserines (PS)

```
. Diacylglycerophosphoserines
. 1-alkyl,2-acylglycerophosphoserines
. 1Z-alkenyl,2-acylglycerophosphoserines
. Dialkylglycerophosphoserines
. Monoacylglycerophosphoserines
. 1-alkyl glycerophosphoserines
. 1Z-alkenylglycerophosphoserines
```

o Glycerophosphoglycerols (PG)

```
. Diacylglycerophosphoglycerols
. 1-alkyl,2-acylglycerophosphoglycerols
. 1Z-alkenyl,2-acylglycerophosphoglycerols
```

- . Dialkylglycerophosphoglycerols
- . Monoacylglycerophosphoglycerols
- . 1-alkyl glycerophosphoglycerols
- . 1Z-alkenylglycerophosphoglycerols

o Glycerophosphoglycerophosphates (PGP)

- . Diacylglycerophosphoglycerophosphates
- . 1-alkyl,2-acylglycerophosphoglycerophosphates
- . 1Z-alkenyl,2-acylglycerophosphoglycerophosphates
- . Dialkylglycerophosphoglycerophosphates
- . Monoacylglycerophosphoglycerophosphates
- . 1-alkyl glycerophosphoglycerophosphates
- . 1Z-alkenylglycerophosphoglycerophosphates

o Glycerophosphoinositols (PI)

- . Diacylglycerophosphoinositols
- . 1-alkyl,2-acylglycerophosphoinositols
- . 1Z-alkenyl,2-acylglycerophosphoinositols
- . Dialkylglycerophosphoinositols
- . Monoacylglycerophosphoinositols
- . 1-alkyl glycerophosphoinositols
- . 1Z-alkenylglycerophosphoinositols

o Glycerophosphoinositol monophosphates (PIP)

- . Diacylglycerophosphoinositol monophosphates
- . 1-alkyl,2-acylglycerophosphoinositol monophosphates
- . 1Z-alkenyl,2-acylglycerophosphoinositol monophosphates
- . Dialkylglycerophosphoinositol monophosphates
- . Monoacylglycerophosphoinositol monophosphates
- . 1-alkyl glycerophosphoinositol monophosphates
- . 1Z-alkenylglycerophosphoinositol monophosphates

o Glycerophosphates (PA)

- . Diacylglycerophosphates
- . 1-alkyl,2-acylglycerophosphates
- . 1Z-alkenyl,2-acylglycerophosphates
- . Dialkylglycerophosphates
- . Monoacylglycerophosphates
- . 1-alkyl glycerophosphates
- . 1Z-alkenylglycerophosphates

o Glyceropyrophosphates (PPA)

- . Diacylglyceropyrophosphates
- . Monoacylglyceropyrophosphates

o Glycerophosphonocholines (PnC)

- . Diacylglycerophosphonocholines
- . 1-alkyl,2-acylglycerophosphonocholines
- . 1Z-alkenyl,2-acylglycerophosphonocholines
- . Dialkylglycerophosphonocholines
- . Monoacylglycerophosphonocholines

```
. 1-alkyl glycerophosphonocholines
. 1Z-alkenylglycerophosphonocholines
```

o Glycerophosphonoethanolamines (PnE)

```
. Diacylglycerophosphonoethanolamines
. 1-alkyl,2-acylglycerophosphonoethanolamines
. 1Z-alkenyl,2-acylglycerophosphonoethanolamines
. Dialkylglycerophosphonoethanolamines
. Monoacylglycerophosphonoethanolamines
. 1-alkyl glycerophosphonoethanolamines
. 1Z-alkenylglycerophosphonoethanolamines
```

OPTIONS

-c, --ChainAbbrevMode *MostLikely|Arbitrary*

Specify what types of acyl chain abbreviations are allowed during processing of complete abbreviations: allow most likely chain abbreviations containing specific double bond geometry specifications; allow any acyl chain abbreviation with valid chain length and double bond geometry specificatios. Possible values: *MostLikely or Arbitrary*. Default value: *MostLikely*.

Arbitrary value of **-c, --ChainAbbrevMode** option is not allowed during processing of abbreviations containing wild cards.

During *MostLikely* value of **-c, --ChainAbbrevMode** option, only the most likely acyl chain abbreviations specified in ChainAbbrev.pm module are allowed. However, during *Arbitrary* value of **-c, --ChainAbbrevMode** option, any acyl chain abbreviations with valid chain length and double bond geometry can be specified. The current release of lipidmapstools support chain lengths from 2 to 50 as specified in ChainAbbev.pm module.

In addition to double bond geometry specifications, valid substituents can be specified for in the acyl chain abbreviations.

-h, --help

Print this help message

-m, --mode *Abbrev|AbbrevFileName*

Controls interpretation of command line arguments. Two different methods are provided: specify compound abbreviations or a file name containing compound abbreviations. Possible values: *Abbrev or AbbrevFileName*. Default: *Abbrev*

In *AbbrevFileName* mode, a single line in CSV/TSV files can contain multiple compound abbreviations. The file extension determines delimiter used to process data lines: comma for CSV and tab for TSV. For files with TXT extension, only one compound abbreviation per line is allowed.

Wild card character, *, is also supported in compound abbreviations.

Examples:

```
Specific structures: PC(12:0/13:0) PC(17:1(9Z)/0:0)
                     PA(13:0/0:0)
Specific structures: PC(O-16:0/13:0) PC(P-16:0/0:0)
Specific possibilities: PC(21:0/22:*) PA(17:*/0:0)
                        PE(O-18:0/*:*)
All possibilites: *(*:*/*:*) or *(*/*)
```

With wild card character, +/- can also be used for chain lengths to indicate even and odd lengths at sn1/sn2/sn3 positions; additionally > and < qualifiers are also allowed to specify length requirements. Examples:

```
Odd and even number chains at sn1 and sn2: *(*+:*/*-:*)
```

```
Odd and even number chains at sn1 and sn2 with length longer than
10
    and 20: *(*+>10:*/*->20:*)
```

Default sn2 stereochemistry is R. However, abbreviation format also supports these additional stereochemistry specifications for sn2 position: S; U - unknown; rac - racemic mixture. Examples:

```
PC(12:0/13:0)[rac]
PC(17:1(9Z)/14:0)[S]
PA(13:0/12:0)[U]
```

-p, --ProcessMode *WriteSDFile|CountOnly*

Specify how abbreviations are processed: generate structures for specified abbreviations along with generating a SD file or just count the number of structures corresponding to specified abbreviations without generating any SD file. Possible values: *WriteSDFile or CountOnly*. Default: *WriteSDFile*.

It can take substantial amount of time for generating all the structures and writing out a SD file for abbreviations containing wild cards. *CountOnly* value of **--ProcessMode** option can be used to get a quick count of number of structures to be generated without writing out any SD file.

-o, --overwrite

Overwrite existing files

-r, --root *rootname*

New file name is generated using the root: <Root>.sdf. Default for new file names: GPAbbrev.sdf, <AbbrevFilenName>.sdf, or <FirstAbbrevFileName>1To<Count>.sdf.

-w, --workingdir *dirname*

Location of working directory. Default: current directory

EXAMPLES

On some systems, command line scripts may need to be invoked using *perl -s GLStrGen.pl*; however, all the examples assume direct invocation of command line script works.

To generate a GPStructures.sdf file containing a structure specified by a command line GP abbreviation, type:

```
% GPStrGen.pl -r GPStructures -o "PC(16:0/0:0)"
```

To generate a GPStructures.sdf file containing structures specified by a command line GL abbreviations, type:

```
% GPStrGen.pl -r GPStructures -o "PC(16:0/0:0)" "PE(18:1(11E)/16:0)"
```

To generate a GPStructures.sdf file containing structures specified by a command line GP abbreviations with specific stereochemistry, type:

```
% GPStrGen.pl -r GPStructures -o "PC(16:0/0:0)[U]"
"PE(18:1(11E)/16:0)[S]"
```

To enumerate all possible GP structures and generate a GPStructures.sdf file, type:

```
% GPStrGen.pl -r GPStructures -o "*(*/*)"
```

or

```
% GPStrGen.pl -r GPStructures -o "*(*:*/*:*)"
```

or

```
% GPStrGen.pl -r GPStructures -o "*(*:*(*)/*:*(*))"
```

To enumerate all possible GP structures with a sn1 chain, and generate a GPStructures.sdf file, type:

```
% GPStrGen.pl -r GPStructures -o "*(*/0:0)"
```

To enumerate all possible GP structures with a sn1 chain containing one double bond, and generate a GPStructures.sdf file, type:

```
% GPStrGen.pl -r GPStructures -o "*(*:1/0:0)"
```

To enumerate all possible GP structures with even chain length larger than 10 at sn1 position, and generate and generate a GPStructures.sdf file, type:

```
% GPStrGen.pl -r GPStructures -o "*(*+>10:*/0:0)"
```

To enumerate all possible GP structures with odd chains longer than 10 at sn1 and even chains longer than 18 at sn2, and generate a GPStructures.sdf file, type:

```
% GPStrGen.pl -r GPStructures -o "*(*->10:*/*+>18:*)"
```

AUTHOR

Manish Sud

CONTRIBUTOR

Eoin Fahy

SEE ALSO

CLStrGen.pl, FAStrGen.pl, GLStrGen.pl, SPStrGen.pl, STStrGen.pl

COPYRIGHT

Copyright (C) 2006-2012. The Regents of the University of California. All Rights Reserved.

LICENSE

Modified BSD License
