# Supplementary material for: The LUX Score: A Metric for Lipidome Homology
Source: PLoS Comput Biol. 2015 Sep 22;11(9):e1004511. doi: 10.1371/journal.pcbi.1004511 (PMC4578897; doi:10.1371/journal.pcbi.1004511)
Supplement: S5 Dataset — Includes scripts, README files and data files for Figs 1, 2, 6, 7 and S6. (ZIP) [file pcbi.1004511.s009.zip › S5_Dataset/Lipidome_Homology_Testing/bin/121010_lipidmapstools/docs/pdfa4/LMStr.pdf]

NAME

LMStr - LIPID MAPS arbitrary structure generation methods

SYNOPSIS

use LMStr;

use LMStr qw(:all);

DESCRIPTION

LMStr module provides these methods:

```
    ExpandLMCmpdAbbrevs - Expand abbreviation
    GenerateCmpdOntologyData - Generate ontology data
    GenerateCmpdOntologySDDataLines - Generate ontology data lines for SD
file
    GenerateLMChainStrData - Generate chain structure data
    GenerateSDFile - Generate SD file
    GetLMTemplatesData - Get templates data
    GetLMSupportedHeadGroupMap - Get supported headgroups data
    GetLMTemplateID - Get templates ID
    IsLMChainsAbbrevSupported - Is it a supported abbreviation
    ParseLMAbbrev - Parse abbreviation
    SetupLMCmpdAbbrevTemplateDataMap - Setup template structure data map
    ValidateLMAbbrev - Validate abbreviation
```

METHODS

ExpandLMCmpdAbbrevs

```
$ExpandedAbbrevArrayRef = ExpandLMCmpdAbbrevs($CmpdAbbrev);
```

Return a reference to an array containing complete LM abbreviations. Wild card characters in LM abbreviation name are expanded to generate fully qualified LM abbreviations.

GenerateCmpdOntologyData

```
$DataHashRef = GenerateCmpdOntologyData($CmpDataRef);
```

Return a reference to a hash containing ontology data with hash keys and values corresponding to property names and values.

GenerateCmpdOntologySDDataLines

```
    $DataLinesArrayRef =
GenerateCmpdOntologySDDataLines($CmpdDataRef);
```

Return a reference to an array containing ontology data lines suitable for generate SD file data block.

GenerateLMChainStrData

```
($AtomLinesArrayRef, $BondLinesArrayRef) =
   GenerateLMChainStrData($ChainType, $CmpdDataRef);
```

Return array references containing atom and bond data lines for SD file. Appropriate atom and bond data lines are generated using chain type and abbreviation template data.

GenerateSDFile

```
GenerateSDFile($SDFileName, $CmdAbbrevsRef);
```

Generate a SD file for compound abbreviations. Structure data for specified abbreviation is

generated sequentially and written to SD file.

GetLMTemplatesData

```
$TemplatesDataRef = GetLMTemplatesData();
```

Return a reference to a hash containing LM templates data

GetLMSupportedHeadGroupMap

```
$SupportedHeadGroupDataRef = GetLMSupportedHeadGroupMap();
```

Return a reference to a hash containing supported head groups data.

GetLMTemplateID

```
$HeadGroupID = GetLMTemplateID($HeadGroupAbbrev, $ChainsAbbrev);
```

Return a supported template ID for compound abbreviation.

IsLMChainsAbbrevSupported

```
$Status = IsLMChainsAbbrevSupported($Abbrev, $PrintWarning);
```

Return 1 or 0 based on whether LM abbreviated is supported. For unsupported LM abbreviations, a warning is printed unless PrintWarning flag is set.

ParseLMAbbrev

```
($HeadGroup, $ChainsAbbrev, $AbbrevModifier) =
   ParseLMAbbrev($Abbrev);
```

Parse LM abbreviation and return these values: HeadGroup, ChainsAbbrev, AbbrevModifier.

SetupLMCmpdAbbrevTemplateDataMap

```
$AbbrevTemplateDataMapRef =
   SetupLMCmpdAbbrevTemplateDataMap($Abbrev);
```

Return a reference to a hash containing template data for compound abbreviation. The template data is used to generate SD file for compound abbreviation.

ValidateLMAbbrev

```
$Status = ValidateLMAbbrev($Abbrev);
```

Return 1 or 0 based on whether a LM abbreviation is valid.

AUTHOR

Manish Sud

CONTRIBUTOR

Eoin Fahy

SEE ALSO

ChainAbbrev.pm, ChainStr.pm, LMAPSStr.pm

COPYRIGHT

Copyright (C) 2006-2012. The Regents of the University of California. All Rights Reserved.

LICENSE

Modified BSD License
