# Supplementary material for: The LUX Score: A Metric for Lipidome Homology
Source: PLoS Comput Biol. 2015 Sep 22;11(9):e1004511. doi: 10.1371/journal.pcbi.1004511 (PMC4578897; doi:10.1371/journal.pcbi.1004511)
Supplement: S5 Dataset — Includes scripts, README files and data files for Figs 1, 2, 6, 7 and S6. (ZIP) [file pcbi.1004511.s009.zip › S5_Dataset/Lipidome_Homology_Testing/bin/121010_lipidmapstools/docs/pdfa4/README.pdf]

## Introduction

LIPID MAPS Tools package provides various command line Perl scripts to generate SD files containing structure and ontological data for various lipid categories.

## Installation

1. Add <YOUR LIPID MAPS TOOLS DIR>/bin to your PATH environment variable.

```
C shell example:

    set path = "$path <YOUR LIPID MAPS TOOLS DIR>/bin"

Bourne shell example:

    PATH = "$PATH:<YOUR LIPID MAPS TOOLS DIR>/bin"

Windows:

    o Right click on "My Computer" icon and select properties. And
       Select "Environment Variables" button under Advanced tab.

    o Select path under "System Variables" and click on Edit button.
And
       system variable "path" to include LIPID MAPS TOOLS bin
directory.
```

2. And you're all set to try out the command line scripts.

## Caveats

On some systems, command line scripts may need to be invoked using "perl -S <ScriptName.pl>" instead of "<ScriptName.pl>". Example:

```
% perl -S GLStrGen.pl
```

## Feedback

webmaster@lipidmaps.org

## Author

Manish Sud

## Contributor(s)

Eoin Fahy

COPYRIGHT

Copyright (C) 2006-2012. The Regents of the University of California. All Rights Reserved.

LICENSE

Modified BSD License
