# Supplementary material for: The LUX Score: A Metric for Lipidome Homology
Source: PLoS Comput Biol. 2015 Sep 22;11(9):e1004511. doi: 10.1371/journal.pcbi.1004511 (PMC4578897; doi:10.1371/journal.pcbi.1004511)
Supplement: S5 Dataset — Includes scripts, README files and data files for Figs 1, 2, 6, 7 and S6. (ZIP) [file pcbi.1004511.s009.zip › S5_Dataset/Lipidome_Homology_Testing/bin/121010_lipidmapstools/docs/pdfa4/SPChainAbbrev.pdf]

NAME

SPChainAbbrev - Methods for processing SP chain abbreviations

SYNOPSIS

use SPChainAbbrev;

use SPChainAbbrev qw(:all);

DESCRIPTION

SPChainAbbrev module provides these methods:

```
    ChainAbbrevNameExists - Is it a supported chain abbreviation
    ExpandChainAbbrev - Expand wild cards in chain abbreviation
    GetChainAbbrevToNameMap - Get chain name
    GetChainLenAbbrevSupportedMap - Get reference to supported chain
                                    abbreviations data
    GetChainLenAbbrevDbleBondGeometyDataMap - Get reference to supported
chain
                                              double bond geometry data
    GetSupportedChainLenList - Get supported chain lengths
    IsSphingosineChainAbbrev - Is it a sphingosine chain abbreviation
    IsSphingosineC18ChainAbbrev - Is it a sphingosine C18 abbreviation
    IsSphinganineC18ChainAbbrev - Is it a sphinganine C18 abbreviation
```

METHODS

ChainAbbrevNameExists

```
$Status = ChainAbbrevNameExists($ChainAbbrev, $ChainType);
```

Return 1 or 0 based on whether it's a supported chain name.

ExpandChainAbbrev

```
$AbbrevArrayRef = ExpandChainAbbrev($Abbrev);
```

Return a reference to an array containing complete chain abbreviations. Wild card characters in chain abbreviation name are expanded to generate fully qualified chain abbreviations.

GetChainAbbrevToNameMap

```
$AbbrevNameHashRef = GetChainAbbrevToNameMap();
```

Return a reference to hash with chain abbreviation/name as key/value pair.

GetChainLenAbbrevSupportedMap

```
$ChainLenHashRef = GetChainLenAbbrevSupportedMap();
```

Return a reference to hash with supported chain length as hash key.

GetChainLenAbbrevDbleBondGeometyDataMap

```
    $ChainLenDblBondHashRef =
GetChainLenAbbrevDbleBondGeometyDataMap();
```

Return a reference to hash containing information about chain length, number of double bonds and geometry of double bonds.

GetSupportedChainLenList

```
$ChainLengthListRef = GetSupportedChainLenList();
```

Return a reference to a sorted list containing supported chain lengths.

IsChainAbbrevOkay

```
$Status = IsChainAbbrevOkay($ChainAbbrev);
```

Return 1 or 0 based on whether chain abbreviation is valid.

IsSphingosineChainAbbrev

```
$Status = IsSphingosineChainAbbrev($ChainAbbrev);
```

Return 1 or 0 based on whether it's a sphingosine chain abbreviation.

IsSphingosineC18ChainAbbrev

```
$Status = IsSphingosineC18ChainAbbrev($ChainAbbrev);
```

Return 1 or 0 based on whether it's a sphingosine abbreviation with chain length of 18.

IsSphinganineC18ChainAbbrev

```
$Status = IsSphinganineC18ChainAbbrev($ChainAbbrev);
```

Return 1 or 0 based on whether it's a sphinganine abbreviation with chain length of 18.

AUTHOR

Manish Sud

CONTRIBUTOR

Eoin Fahy

SEE ALSO

LMAPSStr.pm, ChainStr.pm

COPYRIGHT

Copyright (C) 2006-2012. The Regents of the University of California. All Rights Reserved.

LICENSE

Modified BSD License
