# Supplementary material for: The LUX Score: A Metric for Lipidome Homology
Source: PLoS Comput Biol. 2015 Sep 22;11(9):e1004511. doi: 10.1371/journal.pcbi.1004511 (PMC4578897; doi:10.1371/journal.pcbi.1004511)
Supplement: S5 Dataset — Includes scripts, README files and data files for Figs 1, 2, 6, 7 and S6. (ZIP) [file pcbi.1004511.s009.zip › S5_Dataset/Lipidome_Homology_Testing/bin/121010_lipidmapstools/docs/pdfa4/SPStr.pdf]

NAME

SPStr - Sphingolipids (SP) structure generation methods

SYNOPSIS

use SPStr;

use SPStr qw(:all);

DESCRIPTION

SPStr module provides these methods:

```
    GenerateCmpdOntologyData - Generate ontology data
    GenerateCmpdOntologySDDataLines - Generate ontology data lines for SD
file
    GenerateSPChainStrData - Generate chain structure data
    GenerateSDFile - Generate SD file
    GetSPTemplatesData - Get templates data
    GetSPSupportedHeadGroupMap - Get supported headgroups data
    GetSPTemplateID - Get templates ID
    IsSPChainsAbbrevSupported - Is it a supported SP abbreviation
    ParseSPAbbrev - Parse SP abbreviation
    ProcessSPCmpdAbbrevs - Process SP abbreviation
    SetupSPCmpdAbbrevTemplateDataMap - Setup template structure data map
    ValidateSPAbbrev - Validate SP abbreviation
```

METHODS

GenerateCmpdOntologyData

```
    $DataHashRef = GenerateCmpdOntologyData($CmpDataRef);
```

Return a reference to a hash containing ontology data with hash keys and values corresponding to property names and values.

GenerateCmpdOntologySDDataLines

```
    $DataLinesArrayRef =
GenerateCmpdOntologySDDataLines($CmpdDataRef);
```

Return a reference to an array containing ontology data lines suitable for generate SD file data block.

GenerateSPChainStrData

```
    ($AtomLinesArrayRef, $BondLinesArrayRef) =
       GenerateSPChainStrData($ChainType, $CmpdDataRef);
```

Return array references containing atom and bond data lines for SD file. Appropriate atom and bond data lines are generated using chain type and abbreviation template data.

GenerateSDFile

```
    GenerateSDFile($SDFileName, $CmdAbbrevsRef);
```

Generate a SD file for compound abbreviations. Structure data for specified abbreviation is generated sequentially and written to SD file.

GetSPTemplatesData

```
    $TemplatesDataRef = GetSPTemplatesData();
```

Return a reference to a hash containing SP templates data

GetSPSupportedHeadGroupMap

```
$SupportedHeadGroupDataRef = GetSPSupportedHeadGroupMap();
```

Return a reference to a hash containing supported head groups data.

GetSPTemplateID

```
$HeadGroupID = GetSPTemplateID($HeadGroupAbbrev, $ChainsAbbrev);
```

Return a supported template ID for compound abbreviation.

IsSPChainsAbbrevSupported

```
$Status = IsSPChainsAbbrevSupported($Abbrev, $PrintWarning);
```

Return 1 or 0 based on whether SP abbreviated is supported. For unsupported SP abbreviations, a warning is printed unless PrintWarning flag is set.

ParseSPAbbrev

```
($HeadGroup, $ChainsAbbrev, $AbbrevModifier) =
   ParseSPAbbrev($Abbrev);
```

Parse SP abbreviation and return these values: HeadGroup, ChainsAbbrev, AbbrevModifier.

ProcessSPCmpdAbbrevs

```
ProcessSPCmpdAbbrevs($CmpdAbbrevsRef, $AllowArbitraryChainAbbrev,
                     $WriteSDFile, $SDFileName);
```

Process specified SP abbreviations to generate structures and write them out either a SD file or simply report number of valid abbreviations.

SetupSPCmpdAbbrevTemplateDataMap

```
$AbbrevTemplateDataMapRef =
   SetupSPCmpdAbbrevTemplateDataMap($Abbrev);
```

Return a reference to a hash containing template data for compound abbreviation. The template data is used to generate SD file for compound abbreviation.

ValidateSPAbbrev

```
$Status = ValidateSPAbbrev($Abbrev);
```

Return 1 or 0 based on whether a SP abbreviation is valid.

AUTHOR

Manish Sud

CONTRIBUTOR

Eoin Fahy

SEE ALSO

ChainStr.pm

COPYRIGHT

Copyright (C) 2006-2012. The Regents of the University of California. All Rights Reserved.

LICENSE

Modified BSD License
