# Supplementary material for: The LUX Score: A Metric for Lipidome Homology
Source: PLoS Comput Biol. 2015 Sep 22;11(9):e1004511. doi: 10.1371/journal.pcbi.1004511 (PMC4578897; doi:10.1371/journal.pcbi.1004511)
Supplement: S5 Dataset — Includes scripts, README files and data files for Figs 1, 2, 6, 7 and S6. (ZIP) [file pcbi.1004511.s009.zip › S5_Dataset/Lipidome_Homology_Testing/bin/121010_lipidmapstools/docs/pdfa4/SPStrGen.pdf]

NAME

SPStrGen.pl - Generate structures for Sphingophospholipids (SP)

SYNOPSIS

SPStrGen.pl SPAbbrev|SPAbbrevFileName ...

SPStrGen.pl [**-c, --ChainAbbrevMode** *MostLikely* **|** *Arbitrary*] [**-h, --help**] [**-m, --mode** *Abbrev* **|** *AbbrevFileName*] [**-p, --ProcessMode** *WriteSDFile* **|** *CountOnly*] [**-o, --overwrite**] [**-r, --root** rootname] [**-w, --workingdir** dirname] <arguments>...

DESCRIPTION

Generate Sphingophospholipids (SP) structures using compound abbreviations specified on a command line or in a CSV/TSV Text file. All the command line arguments represent either compound abbreviations or file name containing abbreviations. Use mode option to control the type of command line arguments.

A SD file, containing structures for all SP abbreviations along with ontological information, is generated as an output.

SUPPORTED ABBREVIATIONS

Current support for SP structure generation include these main classes and sub classes:

o Sphingoid bases

```
. Sphing-4-enines (Sphingosines)
. Sphinganines
. 4-Hydroxysphinganines (Phytosphingosines)
. Sphingoid base homologs and variants
. Sphingoid base 1-phosphates
. Lysosphingomyelins and lysoglycosphingolipids
```

o Sphingoid bases

```
. Sphing-4-enines (Sphingosines)
. Sphinganines
. 4-Hydroxysphinganines (Phytosphingosines)
. Sphingoid base homologs and variants
. Sphingoid base 1-phosphates
. Lysosphingomyelins and lysoglycosphingolipids
. Sphingoid base analogs
```

o Ceramides

```
. N-acylsphingosines (ceramides)
. N-acylsphinganines (dihydroceramides)
. N-acyl-4-hydroxysphinganines (phytoceramides)
. Ceramide 1-phosphates
```

o Phosphosphingolipids

```
. Ceramide phosphocholines (sphingomyelins)
. Ceramide phosphoethanolamines
. Ceramide phosphoinositols
```

o Neutral glycosphingolipids

```
. Simple Glc series (GlcCer, LacCer, etc)
```

```
. GalNAcb1-3Gala1-4Galb1-4Glc- (Globo series)
. GalNAcb1-4Galb1-4Glc- (Ganglio series)
. Galb1-3GlcNAcb1-3Galb1-4Glc- (Lacto series)
. Galb1-4GlcNAcb1-3Galb1-4Glc- (Neolacto series)
. GalNAcb1-3Gala1-3Galb1-4Glc- (Isoglobo series)
. GlcNAcb1-2Mana1-3Manb1-4Glc- (Mollu series)
. GalNAcb1-4GlcNAcb1-3Manb1-4Glc- (Arthro series)
. Gal- (Gala series)
```

o Acidic glycosphingolipids

```
. Gangliosides
```

OPTIONS

-c, --ChainAbbrevMode *MostLikely|Arbitrary*

Specify what types of acyl chain abbreviations are allowed during processing of complete abbreviations: allow most likely chain abbreviations containing specific double bond geometry specifications; allow any acyl chain abbreviation with valid chain length and double bond geometry specificatios. Possible values: *MostLikely or Arbitrary*. Default value: *MostLikely*.

Arbitrary value of **-c, --ChainAbbrevMode** option is not allowed during processing of abbreviations containing wild cards.

During *MostLikely* value of **-c, --ChainAbbrevMode** option, only the most likely acyl chain abbreviations specified in ChainAbbrev.pm module are allowed. However, during *Arbitrary* value of **-c, --ChainAbbrevMode** option, any acyl chain abbreviations with valid chain length and double bond geometry can be specified. The current release of lipidmapstools support chain lengths from 2 to 50 as specified in ChainAbbev.pm module.

In addition to double bond geometry specifications, valid substituents can be specified for in the acyl chain abbreviations.

-h, --help

Print this help message

-m, --mode *Abbrev|AbbrevFileName*

Controls interpretation of command line arguments. Two different methods are provided: specify compound abbreviations or a file name containing compound abbreviations. Possible values: *Abbrev or AbbrevFileName*. Default: *Abbrev*

In *AbbrevFileName* mode, a single line in CSV/TSV files can contain multiple compound abbreviations. The file extension determines delimiter used to process data lines: comma for CSV and tab for TSV. For files with TXT extension, only one compound abbreviation per line is allowed.

Wild card character, *, is also supported in compound abbreviations.

Examples:

```
Specific structures: Cer(d18:0/0:0) Cer(d18:1(4E)/0:0)
                     Cer(d19:1(4E)/24:4(5Z,8Z,11Z,14Z))
Specific structures: SM(d18:0/16:0) SM(d19:0/24:1(15Z))
Specific possibilities: Cer(*/0:0) Cer(d18:1(4E)/*)
All possibilites: *(*:*/*:*) or *(*/*)
```

With wild card character, +/- can also be used for chain lengths to indicate even and odd lengths at sn1/sn2/sn3 positions; additionally > and < qualifiers are also allowed to specify length requirements. Examples:

```
Odd and even number chains at sn1 and sn2: *(*-:*/*+:*)
Odd and even number chains at sn1 and sn2 with length longer than
```

```
18
       and 22: *(*->18:*/*+>22:*)
```

-p, --ProcessMode *WriteSDFile*|*CountOnly*

Specify how abbreviations are processed: generate structures for specified abbreviations along with generating a SD file or just count the number of structures corresponding to specified abbreviations without generating any SD file. Possible values: *WriteSDFile or CountOnly*. Default: *WriteSDFile*.

It can take substantial amount of time for generating all the structures and writing out a SD file for abbreviations containing wild cards. *CountOnly* value of **--ProcessMode** option can be used to get a quick count of number of structures to be generated without writing out any SD file.

-o, --overwrite

Overwrite existing files

-r, --root *rootname*

New file name is generated using the root: <Root>.sdf. Default for new file names: SPAbbrev.sdf, <AbbrevFilenName>.sdf, or <FirstAbbrevFileName>1To<Count>.sdf.

-w, --workingdir *dirname*

Location of working directory. Default: current directory

EXAMPLES

On some systems, command line scripts may need to be invoked using *perl -s SPStrGen.pl*; however, all the examples assume direct invocation of command line script works.

To generate a SPStructures.sdf file containing a structure specified by a command line SP abbreviation, type:

```
% SPStrGen.pl -r SPStructures -o "Cer(d18:0/0:0)"
```

To generate a SPStructures.sdf file containing structures specified by a command line SP abbreviations, type:

```
% SPStrGen.pl -r SPStructures -o "SM(d18:0/16:0)" "SM(d19:0/24:1(15Z))"
```

To enumerate all possible SP structures and generate a SPStructures.sdf file, type:

```
% SPStrGen.pl -r SPStructures -o "*(*/*)"
```

or

```
% SPStrGen.pl -r SPStructures -o "*(*:*/*:*)"
```

AUTHOR

Manish Sud

CONTRIBUTOR

Eoin Fahy

SEE ALSO

CLStrGen.pl, FAStrGen.pl, GLStrGen.pl, GPStrGen.pl, STStrGen.pl

COPYRIGHT

Copyright (C) 2006-2012. The Regents of the University of California. All Rights Reserved.

LICENSE

Modified BSD License
