# Supplementary material for: The LUX Score: A Metric for Lipidome Homology
Source: PLoS Comput Biol. 2015 Sep 22;11(9):e1004511. doi: 10.1371/journal.pcbi.1004511 (PMC4578897; doi:10.1371/journal.pcbi.1004511)
Supplement: S5 Dataset — Includes scripts, README files and data files for Figs 1, 2, 6, 7 and S6. (ZIP) [file pcbi.1004511.s009.zip › S5_Dataset/Lipidome_Homology_Testing/bin/121010_lipidmapstools/docs/pdf/ChainAbbrev.pdf]

NAME

ChainAbbrev - Methods for processing chain abbreviations

SYNOPSIS

use ChainAbbrev;

use ChainAbbrev qw(:all);

DESCRIPTION

ChainAbbrev module provides these methods:

```
ChainAbbrevNameExists - Is it a supported chain abbreviation
ExpandChainAbbrev - Expand wild cards in chain abbreviation
GetChainAbbrevToNameMap - Get chain name
GetChainLenAbbrevSupportedMap - Get reference to supported chain
                                abbreviations data
GetChainLenAbbrevDbleBondGeometryDataMap - Get reference to supported
                                           double bond geometry data
GetChainLengthAndMultipleBondCount - Get chain length and number of
                                     double and triple bonds
GetChainLenToNamePrefixMap - Get chain name prefix
GetCountToNamePrefixMap - Get count prefix
GetSubstituentsAbbrevToNameMap - Get substituents name
GetSubstituentBondOrder - Get substituent bond order
GetSupportedChainLenList - Get supported chain lengths
IsAlkylChainAbbrev - Is it a alkyl chain abbreviation
IsAlkenylChainAbbrev - Is it a alkenyl chain abbreviation
IsChainAbbrevOkay - Is it a valid chain abbreviation
IsDoubleBondsAbbrevOkay - Is it a valid double bond abbreviation
IsRingsAbbrevOkay - Is it a valid ring abbreviation
IsSubstituentsAbbrevOkay - Is it a valid substituent abbreviation
IsWildCardInChainAbbrev - Does chain abbreviation contains a wild card
ParseChainAbbrev - Parse chain abbreviation
ParseRingAbbrev - Parse ring abbreviation
ParseSubstituentAbbrev - Parse substituent abbreviation
SetupChainSubstituentsName - Set up substituent name
```

METHODS

ChainAbbrevNameExists

```
$Status = ChainAbbrevNameExists($ChainAbbrev);
```

Return 1 or 0 based on whether it's a supported chain name.

ExpandChainAbbrev

```
$AbbrevArrayRef = ExpandChainAbbrev($Abbrev);
```

Return a reference to an array containing complete chain abbreviations. Wild card characters in chain abbreviation name are expanded to generate fully qualified chain abbreviations.

GetChainAbbrevToNameMap

```
$AbbrevNameHashRef = GetChainAbbrevToNameMap();
```

Return a reference to hash with chain abbreviation/name as key/value pair.

GetChainLenAbbrevSupportedMap

```
$ChainLenHashRef = GetChainLenAbbrevSupportedMap();
```

Return a reference to hash with supported chain length as hash key.

GetChainLenAbbrevDbleBondGeometryDataMap

```
$ChainLenDblBondHashRef =
    GetChainLenAbbrevDbleBondGeometryDataMap();
```

Return a reference to hash containing information about chain length, number of double bonds and geometry of double bonds.

GetChainLengthAndMultipleBondCount

```
($ChainLength, $DoubleBondCount, $TripleBondCount) =
    GetChainLengthAndMultipleBondCount($ChainAbbrev);
```

Parse chain abbreviation and return these values: chain length; number of double and triple bonds.

GetChainLenToNamePrefixMap

```
$ChainNameHashRef = GetChainLenToNamePrefixMap();
```

Return a reference to hash with chain length/name prefix as key/value pair.

GetCountToNamePrefixMap

```
$CountHashRef = GetCountToNamePrefixMap();
```

Return a reference to hash with count/name prefix as key/value pair.

GetSubstituentsAbbrevToNameMap

```
$AbbrevNameHashRef = GetSubstituentsAbbrevToNameMap();
```

Return a reference to hash with substituents abbreviation/name as key/value pair.

GetSubstituentBondOrder

```
$BondOrder = GetSubstituentBondOrder($SubstituentAbbrev);
```

Return bond order for a sustituent.

GetSupportedChainLenList

```
$ChainLengthListRef = GetSupportedChainLenList();
```

Return a reference to a sorted list containing supported chain lengths.

IsAlkylChainAbbrev

```
$Status = IsAlkylChainAbbrev($ChainAbbrev);
```

Return 1 or 0 based on whether it's a alkyl chain abbreviation.

IsAlkenylChainAbbrev

```
$Status = IsAlkenylChainAbbrev($ChainAbbrev);
```

Return 1 or 0 based on whether it's a alkenyl chain abbreviation.

IsChainAbbrevOkay

```
$Status = IsChainAbbrevOkay($ChainAbbrev);
```

Return 1 or 0 based on whether chain abbreviation is valid.

IsDoubleBondsAbbrevOkay

```
$Status = IsDoubleBondsAbbrevOkay($ChainAbbrev, $ChainLength,
    $DoubleBondCount, $DoubleBondGeometry);
```

Return 1 or 0 based on whether chain abbreviation contains a valid multiple bond specification.

IsRingsAbbrevOkay

```
$Status = IsRingsAbbrevOkay($ChainAbbrev, $ChainLength, $Rings);
```

Return 1 or 0 based on whether chain abbreviation contains a valid ring specification.

IsSubstituentsAbbrevOkay

```
$Status = IsSubstituentsAbbrevOkay($ChainAbbrev, $ChainLength,
    $DoubleBondCount, $DoubleBondGeometry, $Substituents);
```

Return 1 or 0 based on whether chain abbreviation contains a valid substituents specification.

IsWildCardInChainAbbrev

```
$Status = IsWildCardInChainAbbrev($ChainAbbrev);
```

Return 1 or 0 based on whether chain abbreviation contains any wild card character.

ParseChainAbbrev

```
($ChainLength, $DoubleBondCount, $DoubleBondGeometry) =
    ParseChainAbbrev($ChainAbbrev);
```

Parse chain abbreviation and return these values: chain length, number of double bonds, and geometry of double bonds.

ParseRingAbbrev

```
($Pos, $StereoChemistry) = ParseRingAbbrev($ChainAbbrev);
```

Parse chain abbreviation and return these values: ring position and stereochemistry specificaton at the ring.

ParseSubstituentAbbrev

```
($Pos, $Name, $StereoChemistry) =
    ParseSubstituentAbbrev($SubstituentAbbrev);
```

Parse substituent abbreviation and return these values: position of the substituent on the chain, name, and stereochemistry of the substituent.

SetupChainSubstituentsName

```
$SubstituentsName = SetupChainSubstituentsName(
    $CmpdAbbrevTemplateDataMapRef, $ChainIndex);
```

Return systematic name for substituents after ordering and grouping substituents by their position.

AUTHOR

Manish Sud

CONTRIBUTOR

Eoin Fahy

SEE ALSO

LMAPSStr.pm, ChainStr.pm

COPYRIGHT

Copyright (C) 2006-2012. The Regents of the University of California. All Rights Reserved.

LICENSE

Modified BSD License
