# Supplementary material for: The LUX Score: A Metric for Lipidome Homology
Source: PLoS Comput Biol. 2015 Sep 22;11(9):e1004511. doi: 10.1371/journal.pcbi.1004511 (PMC4578897; doi:10.1371/journal.pcbi.1004511)
Supplement: S5 Dataset — Includes scripts, README files and data files for Figs 1, 2, 6, 7 and S6. (ZIP) [file pcbi.1004511.s009.zip › S5_Dataset/Lipidome_Homology_Testing/bin/121010_lipidmapstools/docs/pdf/ChainStr.pdf]

NAME

ChainStr - Fatty Acyls (FA), Glycerolipids (GL) and Glycerophospholipids (GP) structure data generation methods

SYNOPSIS

use ChainStr;

use ChainStr qw(:all);

DESCRIPTION

ChainStr module provides these methods:

```
AssignSubstituentStereoChemistry - Assign stereochemistry to substituents
GenerateAtomBlockLines - Generate SD file atom data lines
GenerateBondBlockLines - Generate SD file bond data lines
GenerateChainStrData - Generate structure data for chains
GenerateCmpdCountsLine - Generate SD file count data line
IsAnySubstituentSpecifiedWithStereoChemistry - Check stereochemistry of
                                                substituents
SetupTemplateDataMap - Set up template data for a compound abbreviation
```

METHODS

AssignSubstituentStereoChemistry

```
    AssignSubstituentStereoChemistry($CmpdAbbrevTemplateDataMapRef,
        $CmdDataLinesRef);
```

Assign stereochemistry to substituents using structure and stereochemistry data available via $CmpdAbbrevTemplateDataMapRef and $CmdDataLinesRef. And add new lines to existing structure data using $CmdDataLinesRef.

GenerateAtomBlockLines

```
    $AtomDataLines =
GenerateAtomBlockLines($CmpdAbbrevTemplateDataMapRef,
        $Sn1AtomLinesRef, $Sn2AtomLinesRef, $Sn3AtomLinesRef);
```

Return atom data lines suitable for writing to SD file. Atom data for all approrpriate chains is merged into a single string using new line character as delimiter.

GenerateBondBlockLines

```
    $BondDataLines =
GenerateBondBlockLines($CmpdAbbrevTemplateDataMapRef,
        $Sn1BondLinesRef, $Sn2AtomLinesRef, $Sn3AtomLinesRef);
```

Return bond data lines suitable for writing to SD file. Bond data for all approrpriate chains is merged into a single string using new line character as delimiter.

GenerateChainStrData

```
    ($AtomLinesArrayRef, $BondLinesArrayRef) =
GenerateChainStrData($ChainType,
        $CmpdAbbrevTemplateDataMapRef);
```

Return array references containing atom and bond data lines for SD file. Appropriate atom and bond data lines are generated using chain type and abbreviation template data.

GenerateCmpdCountsLine

```
$RetValue = GenerateCmpdCountsLine($CmpdAbbrevTemplateDataMapRef);
```

Return a formatted count data line for SD file.

IsAnySubstituentSpecifiedWithStereoChemistry

```
$Status = IsAnySubstituentSpecifiedWithStereoChemistry(
    $CmpdAbbrevTemplateDataMapRef);
```

Return 1 or 0 based on whether stereochemistry is specifed for any substituent.

SetupTemplateDataMap

```
SetupTemplateDataMap($TemplateType, $AbbrevTemplateDataMapRef,
    $TemplateData);
```

Setup compound abbreviation template data using a supported template.

AUTHOR

Manish Sud

CONTRIBUTOR

Eoin Fahy

SEE ALSO

FAStr.pm, GLStr.pm, GPStr.pm, LMAPSStr.pm, SPStr.pm

COPYRIGHT

Copyright (C) 2006-2012. The Regents of the University of California. All Rights Reserved.

LICENSE

Modified BSD License
