# Supplementary material for: The LUX Score: A Metric for Lipidome Homology
Source: PLoS Comput Biol. 2015 Sep 22;11(9):e1004511. doi: 10.1371/journal.pcbi.1004511 (PMC4578897; doi:10.1371/journal.pcbi.1004511)
Supplement: S5 Dataset — Includes scripts, README files and data files for Figs 1, 2, 6, 7 and S6. (ZIP) [file pcbi.1004511.s009.zip › S5_Dataset/Lipidome_Homology_Testing/bin/121010_lipidmapstools/docs/pdf/GLStr.pdf]

NAME

GLStr - Glycerolipids (GL) structure generation methods

SYNOPSIS

use GLStr;

use GLStr qw(:all);

DESCRIPTION

GLStr module provides these methods:

```
GenerateCmpdOntologyData - Generate ontology data
GenerateCmpdOntologySDDataLines - Generate ontology data lines for SD file
GenerateGLChainStrData - Generate chain structure data
GenerateSDFile - Generate SD file
IsGLAbbrevSupported - Is it a supported GL abbreviation
ParseGLAbrev - Parse GL abbreviation
ProcessGLCmpdAbbrevs - Process GL abbreviation
SetupGLCmpdAbbrevTemplateDataMap - Setup template structure data map
ValidateGLAbbrev - Validate GL abbreviation
```

METHODS

ExpandGLCmpdAbbrevs

```
$ExpandedAbbrevArrayRef = ExpandGLCmpdAbbrevs($CmpdAbbrev);
```

Return a reference to an array containing complete GL abbreviations. Wild card characters in GL abbreviation name are expanded to generate fully qualified GL abbreviations.

GenerateCmpdOntologyData

```
$DataHashRef = GenerateCmpdOntologyData($CmpdDataRef);
```

Return a reference to a hash containing ontology data with hash keys and values corresponding to property names and values.

GenerateCmpdOntologySDDataLines

```
$DataLinesArrayRef = GenerateCmpdOntologySDDataLines($CmpDataRef);
```

Return a reference to an array containing ontology data lines suitable for generate SD file data block.

GenerateGLChainStrData

```
($AtomLinesArrayRef, $BondLinesArrayRef) =
   GenerateGLChainStrData($ChainType, $CmpdDataRef);
```

Return array references containing atom and bond data lines for SD file. Appropriate atom and bond data lines are generated using chain type and abbreviation template data.

GenerateSDFile

```
GenerateSDFile($SDFileName, $CmdAbbrevsRef);
```

Generate a SD file for compound abbreviations. Structure data for specified abbreviation is

generated sequentially and written to SD file.

IsGLAbbrevSupported

```
$Status = IsGLAbbrevSupported($Abbrev, $PrintWarning);
```

Return 1 or 0 based on whether GL abbreviated is supported. For unsupported GL abbreviations, a warning is printed unless PrintWarning flag is set.

ParseGLAbrev

```
($GLType, $ChainsAbbrev, $AbbrevModifier) = ParseGLAbrev($Abbrev);
```

Parse GL abbreviation and return these values: GLType, ChainsAbbrev, AbbrevModifier.

ProcessGLCmpdAbbrevs

```
ProcessGLCmpdAbbrevs($CmpdAbbrevsRef, $AllowArbitraryChainAbbrev,
                     $WriteSDFile, $SDFileName);
```

Process specified GL abbreviations to generate structures and write them out either a SD file or simply report number of valid abbreviations.

SetupGLCmpdAbbrevTemplateDataMap

```
$AbbrevTemplateDataMapRef =
   SetupGLCmpdAbbrevTemplateDataMap($Abbrev);
```

Return a reference to a hash containing template data for compound abbreviation. The template data is used to generate SD file for compound abbreviation.

ValidateGLAbbrev

```
$Status = ValidateGLAbbrev($Abbrev);
```

Return 1 or 0 based on whether a GL abbreviation is valid.

AUTHOR

Manish Sud

CONTRIBUTOR

Eoin Fahy

SEE ALSO

ChainAbbrev.pm, ChainStr.pm, LMAPSStr.pm

COPYRIGHT

Copyright (C) 2006-2012. The Regents of the University of California. All Rights Reserved.

LICENSE

Modified BSD License
