# Supplementary material for: The LUX Score: A Metric for Lipidome Homology
Source: PLoS Comput Biol. 2015 Sep 22;11(9):e1004511. doi: 10.1371/journal.pcbi.1004511 (PMC4578897; doi:10.1371/journal.pcbi.1004511)
Supplement: S5 Dataset — Includes scripts, README files and data files for Figs 1, 2, 6, 7 and S6. (ZIP) [file pcbi.1004511.s009.zip › S5_Dataset/Lipidome_Homology_Testing/bin/121010_lipidmapstools/docs/pdf/GLStrGen.pdf]

NAME

GLStrGen.pl - Generate structures for Glycerolipids (GL)

SYNOPSIS

GLStrGen.pl GLAbbrev|GLAbbrevFileName ...

GLStrGen.pl [**-c, --ChainAbbrevMode** *MostLikely* **|** *Arbitrary*] [**-h, --help**] [**-m, --mode** *Abbrev* **|** *AbbrevFileName*] [**-p, --ProcessMode** *WriteSDFile* **|** *CountOnly*] [**-o, --overwrite**] [**-r, --root** rootname] [**-w, --workingdir** dirname] <arguments>...

DESCRIPTION

Generate Glycerolipids (GL) structures using compound abbreviations specified on a command line or in a CSV/TSV Text file. All the command line arguments represent either compound abbreviations or file name containing abbreviations. Use *-m, --mode* option to control the type of command line arguments.

A SD file, containing structures for all GL abbreviations along with ontological information, is generated as an output.

SUPPORTED ABBREVIATIONS

Current support for GL structure generation include these main classes and sub classes:

o Monoradylglycerols

```
. Monoacylglycerols
. Monoalkylglycerols
. Mono-(1Z-alkenyl)-glycerol
```

o Diradylglycerols

```
. Diacylglycerols
. Alkyl, acylglycerols
. Dialkylglycerols
. 1Z-alkenyl, acylglycerols
```

o Triradylglycerols

```
. Triacylglycerols
. Alkyl, diacylglycerols
. Dialkyl, monoacylglycerols
. 1Z-alkenyl, diacylglycerols
```

OPTIONS

-c, --ChainAbbrevMode *MostLikely|Arbitrary*

Specify what types of acyl chain abbreviations are allowed during processing of complete abbreviations: allow most likely chain abbreviations containing specific double bond geometry specifications; allow any acyl chain abbreviation with valid chain length and double bond geometry specificatios. Possible values: *MostLikely or Arbitrary*. Default value: *MostLikely*.

Arbitrary value of **-c, --ChainAbbrevMode** option is not allowed during processing of abbreviations containing wild cards.

During *MostLikely* value of **-c, --ChainAbbrevMode** option, only the most likely acyl chain abbreviations specified in ChainAbbrev.pm module are allowed. However, during *Arbitrary* value

of **-c, --ChainAbbrevMode** option, any acyl chain abbreviations with valid chain length and double bond geometry can be specified. The current release of lipidmapstools support chain lengths from 2 to 50 as specified in ChainAbbev.pm module.

In addition to double bond geometry specifications, valid substituents can be specified for in the acyl chain abbreviations.

-h, --help

Print this help message

-m, --mode *Abbrev|AbbrevFileName*

Controls interpretation of command line arguments. Two different methods are provided: specify compound abbreviations or a file name containing compound abbreviations. Possible values: *Abbrev or AbbrevFileName*. Default: *Abbrev*

In *AbbrevFileName* mode, a single line in CSV/TSV files can contain multiple compound abbreviations. The file extension determines delimiter used to process data lines: comma for CSV and tab for TSV. For files with TXT extension, only one compound abbreviation per line is allowed.

Wild card character, *, is also supported in compound abbreviations.

Examples:

```
    Specific structures: MG(16:0/0:0/0:0) DG(18:1(11E)/16:0/0:0)
                         TG(16:0/16:0/18:1(9Z))
    Specific structures: MG(O-16:0/0:0/0:0) DG(P-16:0/16:0/0:0)
                         TG(O-20:0/16:0/18:1(9Z))
    Specific possibilities: DG(18:*/16:0/0:0) DG(18:1(*)/16:0/0:0)
                            DG(*:*(9Z)/16:0/0:0)
                            DG(*:*(9Z)/*:*(11E)/0:0)
    All TG possibilities: *(*:*/*:*/*:*) or *(*/*/*)
    All MG, DG and TG possibilities: "MG(*:*/0:0/0:0)" "DG(*:*/*:*/0:0)"
                                     "DG(*.*/0:0/*:*)" "TG(*:*/*:*/*:*)"
```

Along with wild card character, +/- can also be used for chain lengths to indicate even and odd lengths at sn1/sn2/sn3 positions; additionally > and < qualifiers are also allowed to specify length requirements. Examples:

```
    Odd and even number chains at sn1 and sn2: TG(*+:*/*-:*/*:*)
    Odd and even number chains at sn1 and sn2 with length longer than
    10 and 20: TG(*+>10:*/*->20:*/*:*)
```

Default sn2 stereochemistry is R. However, abbreviation format also supports these additional stereochemistry specifications for sn2 position: S; U - unknown; rac - racemic mixture. Examples:

```
    MG(16:0/0:0/0:0)[rac] - racemic mixture
    DG(18:1(11E)/16:0/0:0)[S] - sn2 stereochemistry is S instead of
default R
    TG(16:0/16:0/18:1(9Z))[U] - sn2 stereochemistry is unknown
```

To generate all isomers for specific chains in DG and TG, use of iso designation is also supported. Stereochemistry specification support is not available with isomeric structure generation. Examples:

```
    DG(18:1(11E)/16:0/0:0)[iso2] - Two isomeric structures
    TG(16:0/16:0/18:1(9Z))[iso3] - Three isomeric structures
    TG(16:0/18:0/18:1(9Z))[iso6] - Six isomeric structures
```

Additionally, all isomeric structures can also be generated by explicit specification of chains at different positions:

```
DG(18:1(11E)/16:0/0:0) DG(16:0/18:1(11E)/0:0)
TG(16:0/16:0/18:1(9Z)) TG(16:0/18:1(9Z)/16:0)
TG(18:1(9Z)/16:0/16:0/)
```

Wild card chain abbreviations are supported with sn2 stereochemistry but not with isomer abbreviation.

-p, --ProcessMode *WriteSDFile|CountOnly*

Specify how abbreviations are processed: generate structures for specified abbreviations along with generating a SD file or just count the number of structures corresponding to specified abbreviations without generating any SD file. Possible values: *WriteSDFile or CountOnly*. Default: *WriteSDFile*.

It can take substantial amount of time for generating all the structures and writing out a SD file for abbreviations containing wild cards. *CountOnly* value of **--ProcessMode** option can be used to get a quick count of number of structures to be generated without writing out any SD file.

-o, --overwrite

Overwrite existing files

-r, --root *rootname*

New file name is generated using the root: <Root>.sdf. Default for new file names: GLAbbrev.sdf, <AbbrevFilenName>.sdf, or <FirstAbbrevFileName>1To<Count>.sdf.

-w, --workingdir *dirname*

Location of working directory. Default: current directory

EXAMPLES

On some systems, command line scripts may need to be invoked using *perl -s GLStrGen.pl*; however, all the examples assume direct invocation of command line script works.

To generate a GLStructures.sdf file containing a structure specified by a command line GL abbreviation, type:

```
% GLStrGen.pl -r GLStructures -o "MG(16:0/0:0/0:0)"
```

To generate a GLStructures.sdf file containing structures specified by a command line GL abbreviations, type:

```
% GLStrGen.pl -r GLStructures -o "MG(16:0/0:0/0:0)"
"DG(18:1(11E)/16:0/0:0)" "TG(16:0/16:0/18:1(9Z))"
```

To generate a GLStructures.sdf file containing structures specified by a command line GL abbreviations with specific stereochemistry, type:

```
% GLStrGen.pl -r GLStructures -o "MG(16:0/0:0/0:0)[rac]"
"DG(18:1(11E)/16:0/0:0)[S]" "TG(16:0/16:0/18:1(9Z))[U]"
```

To generate a GLStructures.sdf file containing all isomeric structures specified by a command line GL abbreviations, type:

```
% GLStrGen.pl -r GLStructures -o "DG(18:1(11E)/16:0/0:0)[iso2]"
```

```
"TG(16:0/16:0/18:1(9Z))[iso3]" "TG(16:0/17:0/18:1(9Z))[iso6]"
```

To enumerate all possible GL structures and generate a GLStructures.sdf file, type:

```
% GLStrGen.pl -r GLStructures -o "*(*/*/*)"
```

or

```
% GLStrGen.pl -r GLStructures -o "*(*:*/*:*/*:*)"
```

or

```
% GLStrGen.pl -r GLStructures -o "*(*:*(*)/*:*(*)/*:*(*))"
```

To enumerate all possible Monoradylglycerols structures and generate a MonoGLStructures.sdf file, type:

```
% GLStrGen.pl -r MonoGLStructures -o "MG(*/0:0/0:0)"
```

To enumerate all possible Diradylglycerols structures and generate a DiGLStructures.sdf file, type:

```
% GLStrGen.pl -r DiGLStructures -o "DG(*/*/0:0)"
```

To enumerate all possible Monoradylglycerols structures with one double bond on acyl chain and generate a GLStructures.sdf file, type:

```
% GLStrGen.pl -r MonoGLStructures -o "MG(*:1/0:0/0:0)"
```

To enumerate all possible Monoradylglycerols structures with even chain lengths and generate a GLStructures.sdf file, type:

```
% GLStrGen.pl -r MonoGLStructures -o "MG(*+:*/0:0/0:0)"
```

To enumerate all possible Diradylglycerols structures with odd chains longer than 10 at sn1 and even chains longer than 18 at sn2, and generate a DiGLStructures.sdf file, type:

```
% GLStrGen.pl -r DiGLStructures -o "DG(*->10:*/*+>18:*/0:0)"
```

AUTHOR

Manish Sud

CONTRIBUTOR

Eoin Fahy

SEE ALSO

CLStrGen.pl, FAStrGen.pl, GPStrGen.pl, SPStrGen.pl, STStrGen.pl

COPYRIGHT

Copyright (C) 2006-2012. The Regents of the University of California. All Rights Reserved.

LICENSE

Modified BSD License
