# Supplementary material for: The LUX Score: A Metric for Lipidome Homology
Source: PLoS Comput Biol. 2015 Sep 22;11(9):e1004511. doi: 10.1371/journal.pcbi.1004511 (PMC4578897; doi:10.1371/journal.pcbi.1004511)
Supplement: S5 Dataset — Includes scripts, README files and data files for Figs 1, 2, 6, 7 and S6. (ZIP) [file pcbi.1004511.s009.zip › S5_Dataset/Lipidome_Homology_Testing/bin/121010_lipidmapstools/docs/pdf/GPStr.pdf]

NAME

GPStr - Glycerolipids (GP) structure generation methods

SYNOPSIS

use GPStr;

use GPStr qw(:all);

DESCRIPTION

GPStr module provides these methods:

```
GenerateCmpdOntologyData - Generate ontology data
GenerateCmpdOntologySDDataLines - Generate ontology data lines for SD file
GenerateGPChainStrData - Generate chain structure data
GenerateSDFile - Generate SD file
GetGPTemplatesData - Get templates data
GetGPSupportedHeadGroupMap - Get supported headgroups data
GetGPTemplateID - Get templates ID
IsGPChainsAbbrevSupported - Is it a supported GP abbreviation
ParseGPAbbrev - Parse GP abbreviation
ProcessGPCmpdAbbrevs - Process GP abbreviation
SetupGPCmpdAbbrevTemplateDataMap - Setup template structure data map
ValidateGPAbbrev - Validate GP abbreviation
```

METHODS

GenerateCmpdOntologyData

```
$DataHashRef = GenerateCmpdOntologyData($CmpDataRef);
```

Return a reference to a hash containing ontology data with hash keys and values corresponding to property names and values.

GenerateCmpdOntologySDDataLines

```
$DataLinesArrayRef = GenerateCmpdOntologySDDataLines($CmpdDataRef);
```

Return a reference to an array containing ontology data lines suitable for generate SD file data block.

GenerateGPChainStrData

```
($AtomLinesArrayRef, $BondLinesArrayRef) =
   GenerateGPChainStrData($ChainType, $CmpdDataRef);
```

Return array references containing atom and bond data lines for SD file. Appropriate atom and bond data lines are generated using chain type and abbreviation template data.

GenerateSDFile

```
GenerateSDFile($SDFileName, $CmdAbbrevsRef);
```

Generate a SD file for compound abbreviations. Structure data for specified abbreviation is generated sequentially and written to SD file.

GetGPTemplatesData

```
$TemplatesDataRef = GetGPTemplatesData();
```

Return a reference to a hash containing GP templates data

GetGPSupportedHeadGroupMap

```
$SupportedHeadGroupDataRef = GetGPSupportedHeadGroupMap();
```

Return a reference to a hash containing supported head groups data.

GetGPTemplateID

```
$HeadGroupID = GetGPTemplateID($HeadGroupAbbrev, $ChainsAbbrev);
```

Return a supported template ID for compound abbreviation.

IsGPChainsAbbrevSupported

```
$Status = IsGPChainsAbbrevSupported($Abbrev, $PrintWarning);
```

Return 1 or 0 based on whether GP abbreviated is supported. For unsupported GP abbreviations, a warning is printed unless PrintWarning flag is set.

ParseGPAbbrev

```
($HeadGroup, $ChainsAbbrev, $AbbrevModifier) =
   ParseGPAbbrev($Abbrev);
```

Parse GP abbreviation and return these values: HeadGroup, ChainsAbbrev, AbbrevModifier.

ProcessGPCmpdAbbrevs

```
ProcessGPCmpdAbbrevs($CmpdAbbrevsRef, $AllowArbitraryChainAbbrev,
                     $WriteSDFile, $SDFileName);
```

Process specified GP abbreviations to generate structures and write them out either a SD file or simply report number of valid abbreviations.

SetupGPCmpdAbbrevTemplateDataMap

```
$AbbrevTemplateDataMapRef =
   SetupGPCmpdAbbrevTemplateDataMap($Abbrev);
```

Return a reference to a hash containing template data for compound abbreviation. The template data is used to generate SD file for compound abbreviation.

ValidateGPAbbrev

```
$Status = ValidateGPAbbrev($Abbrev);
```

Return 1 or 0 based on whether a GP abbreviation is valid.

AUTHOR

Manish Sud

CONTRIBUTOR

Eoin Fahy

SEE ALSO

ChainAbbrev.pm, ChainStr.pm, LMAPSStr.pm

COPYRIGHT

Copyright (C) 2006-2012. The Regents of the University of California. All Rights Reserved.

LICENSE

Modified BSD License
