# Supplementary material for: The LUX Score: A Metric for Lipidome Homology
Source: PLoS Comput Biol. 2015 Sep 22;11(9):e1004511. doi: 10.1371/journal.pcbi.1004511 (PMC4578897; doi:10.1371/journal.pcbi.1004511)
Supplement: S5 Dataset — Includes scripts, README files and data files for Figs 1, 2, 6, 7 and S6. (ZIP) [file pcbi.1004511.s009.zip › S5_Dataset/Lipidome_Homology_Testing/bin/121010_lipidmapstools/docs/pdf/LMAPSStr.pdf]

NAME

LMAPSStr - Glycerolipids (GL) structure generation methods

SYNOPSIS

use LMAPSStr;

use LMAPSStr qw(:all);

DESCRIPTION

LMAPSStr module provides these methods:

```
GenerateCmpdAtomLine - Generate SD file atom data line
GenerateCmpdBondLine - Generate SD file bond data line
GenerateCmpdCountsLine - Generate SD file count data line
GenerateCmpdMiscInfoLine - Generate SD file misc data line
ParseCmpdAtomLine - Parse SD file atom data line
ParseCmpdBondLine - Parse SD file bond data line
ParseCmpdCountsLine - Parse SD file count data line
RoundToNextInteger - Round up to next integer
SetupCmpdAbbrevs - Setup lipid abbreviations
SetupSDFileName - Setup SD file name
StandardizeStereochemistrySpec - Standardize stereochemistry
StandardizeStereochemistrySpec - Standardize ring stereochemistry
```

METHODS

GenerateCmpdAtomLine

```
$Line = GenerateCmpdAtomLine($AtomX, $AtomY, $AtomZ, $AtomSymbol);
```

Return a formatted atom data line for SD file.

GenerateCmpdBondLine

```
$Line = GenerateCmpdBondLine($FirstAtomNum, $SecondAtomNum,
        $BondType, [$BondStereo]);
```

Return a formatted bond data line for SD file.

GenerateCmpdCountsLine

```
$Line = GenerateCmpdCountsLine($AtomCount, $BondCount,
        [$ChiralFlag, $PropertyCount, $Version]);
```

Return a formatted count data line for SD file.

GenerateCmpdMiscInfoLine

```
$Line = GenerateCmpdMiscInfoLine();
```

Return a formatted miscellaneous data line for SD file. In addition to a time stamp, LipdMAPS name is used as the program name.

ParseCmpdAtomLine

```
($AtomX, $AtomY, $AtomZ, $AtomSymbol) = ParseCmpdAtomLine($Line);
```

Parse SD file atom data line and return a list with these values: atom coordinates and element symbol.

ParseCmpdBondLine

```
($FirstAtomNum, $SecondAtomNum, $BondType, $BondStereo) =
    ParseCmpdBondLine($Line);
```

Parse SD file atom bond data line and return a list containing these values: bond atom numbers and bond type.

ParseCmpdCountsLine

```
($AtomCount, $BondCount, $ChiralFlag, $PropertyCount, $Version) =
     ParseCmpdCountsLine($Line);
```

Parse SD file count data line and return a list containing these values: atom/bond count and other miscellaneous count information.

RoundToNextInteger

```
$IntegerValue = RoundToNextInteger($Number);
```

Return an integer by rounding the number off to next integer.

SetupCmpdAbbrevs

```
$AbbrebArrayRef = SetupCmpdAbbrevs($CmdLineOptionsRef);
```

Return a reference to an array containing specified compound abbreviations by parsing command line arguments or processing files containing specified abbreviations.

SetupSDFileName

```
$SDFilename = SetupSDFileName($LipidCategory, $CmdLineOptionsRef);
```

Return a SD file name by processing a specified *-r, --root* option or using default values.

StandardizeStereochemistrySpec

```
    $StandardizeSpec =
StandardizeStereochemistrySpec($StereochemistrySpec);
```

Return a standardize stereochemistry specification containg R/S instead of a/b or alpha/beta.

StandardizeRingStereochemistrySpec

```
    $StandardizeSpec =
StandardizeRingStereochemistrySpec($StereochemistrySpec);
```

Return a standardize stereochemistry specification containg alpha/beta instead of a/b or alpha/beta.

AUTHOR

Manish Sud

CONTRIBUTOR

Eoin Fahy

SEE ALSO

ChainAbbrev.pm, ChainStr.pm

COPYRIGHT

Copyright (C) 2006-2012. The Regents of the University of California. All Rights Reserved.

LICENSE

Modified BSD License
