# Supplementary material for: The LUX Score: A Metric for Lipidome Homology
Source: PLoS Comput Biol. 2015 Sep 22;11(9):e1004511. doi: 10.1371/journal.pcbi.1004511 (PMC4578897; doi:10.1371/journal.pcbi.1004511)
Supplement: S5 Dataset — Includes scripts, README files and data files for Figs 1, 2, 6, 7 and S6. (ZIP) [file pcbi.1004511.s009.zip › S5_Dataset/Lipidome_Homology_Testing/bin/121010_lipidmapstools/docs/pdf/LMStrGen.pdf]

NAME

LMStrGen.pl - Generate arbitrary LIPID MAPS structures

SYNOPSIS

LMStrGen.pl LMAbbrev|LMAbbrevFileName ...

LMStrGen.pl [**-h, --help**] [**-o, --overwrite**] [**-r, --root** rootname] [**-w, --workingdir** dirname] <arguments>...

DESCRIPTION

Generate arbitrary LIPID MAPS (LM) structures using compound abbreviations specified on a command line or in a CSV/TSV Text file. All the command line arguments represent either compound abbreviations or file name containing abbreviations. Use mode option to control the type of command line arguments.

A SD file, containing structures for all LM abbreviations along with ontological information, is generated as an output.

SUPPORTED ABBREVIATIONS

Current support for LM structure generation include these headgroups and acyl chains:

```
o DIMAXX - sn1, sn2 and sn3
o DIMA20 - sn1 and sn2
o DIMA22 - sn1 and sn2
o DIMA20Me - sn1 and sn2
o DIMA22Me - sn1 and sn2
o DIMB20 - sn1 and sn2
o DIMB22 - sn1 and sn2
o DIB20Me - sn1 and sn2
o DIB22Me - sn1 and sn2
o PIM1 - sn1 and sn2
o PIM2 - sn1 and sn2
o PIM3 - sn1 and sn2
o PIM4 - sn1 and sn2
o PIM5 - sn1 and sn2
o PIM6 - sn1 and sn2
o DAT - sn1 and sn2
o CoA - sn1
```

OPTIONS

-h, --help

Print this help message

-m, --mode *Abbrev|AbbrevFileName*

Controls interpretation of command line arguments. Two different methods are provided: specify compound abbreviations or a file name containing compound abbreviations. Possible values: *Abbrev or AbbrevFileName*. Default: *Abbrev*

In *AbbrevFileName* mode, a single line in CSV/TSV files can contain multiple compound abbreviations. The file extension determines delimiter used to process data lines: comma for CSV and tab for TSV. For files with TXT extension, only one compound abbreviation per line is allowed.

Wild card character, *, is not supported in compound abbreviations.

Examples:

```
               Specific structures: DIMA20(12:0/13:0) DIMB20(17:1(9Z)/18:0)
                                    CoA(16:0) PIM1(21:0/22:0)
```

-o, --overwrite

Overwrite existing files

-r, --root *rootname*

New file name is generated using the root: <Root>.sdf. Default for new file names: LMAbbrev.sdf, <AbbrevFilenName>.sdf, or <FirstAbbrevFileName>1To<Count>.sdf.

-w, --workingdir *dirname*

Location of working directory. Default: current directory

EXAMPLES

On some systems, command line scripts may need to be invoked using *perl -s LMStrGen.pl*; however, all the examples assume direct invocation of command line script works.

To generate a LMStructures.sdf file containing a structure specified by a command line LM abbreviation, type:

```
    % LMStrGen.pl -r LMStructures -o "DIMA20(12:0/13:0)"
```

To generate a LMStructures.sdf file containing structures specified by a command line LM abbreviations, type:

```
    % LMStrGen.pl -r LMStructures -o "DIMA22(16:0/18:0)"
"DIMA22Me(18:1(11E)/16:0)"
```

AUTHOR

Manish Sud

CONTRIBUTOR

Eoin Fahy

SEE ALSO

CLStrGen.pl, FAStrGen.pl, GLStrGen.pl, SPStrGen.pl, STStrGen.pl

COPYRIGHT

Copyright (C) 2006-2012. The Regents of the University of California. All Rights Reserved.

LICENSE

Modified BSD License
