# Supplementary material for: The LUX Score: A Metric for Lipidome Homology
Source: PLoS Comput Biol. 2015 Sep 22;11(9):e1004511. doi: 10.1371/journal.pcbi.1004511 (PMC4578897; doi:10.1371/journal.pcbi.1004511)
Supplement: S5 Dataset — Includes scripts, README files and data files for Figs 1, 2, 6, 7 and S6. (ZIP) [file pcbi.1004511.s009.zip › S5_Dataset/Lipidome_Homology_Testing/bin/121010_lipidmapstools/docs/pdf/STStr.pdf]

NAME

STStr - Sterol (ST) structure generation methods

SYNOPSIS

use STStr;

use STStr qw(:all);

DESCRIPTION

STStr module provides these methods:

```
ExpandSTCmpdAbbrevs - Expand ST abbreviation
GenerateCmpdOntologyData - Generate ontology data
GenerateCmpdOntologySDDataLines - Generate ontology data lines for
                                  SD file
GenerateSTStrData - Generate structure data
GenerateSDFile - Generate SD file
IsSTAbbrevSupported - Is it a supported ST abbreviation
IsSTSubstituentsNameSupported - Is it a supported ST substituent name
IsSTDoubleBondsAbbrevOkay - Is it a valid ST double bond abbreviation
IsSTSubstituentsAbbrevOkay - Is it a valid ST substituent abbreviation
IsWildCardInSTAbbrev - Does ST abbreviatio contains a wild card
ParseSTAbrev - Parse ST abbreviation
ParseSTDoubleBondAbbrev - Parse ST double bond abbreviation
ParseSTSubstituentAbbrev - Parse ST substituent abbreviation
SetupSTCmpdAbbrevTemplateDataMap - Setup template structure data map
ValidateSTAbbrev - Validate ST abbreviation
```

METHODS

ExpandSTCmpdAbbrevs

```
$ExpandedAbbrevArrayRef = ExpandSTCmpdAbbrevs($CmpdAbbrev);
```

Return a reference to an array containing complete ST abbreviations. Wild card characters in ST abbreviation name are expanded to generate fully qualified ST abbreviations.

GenerateCmpdOntologyData

```
$DataHashRef = GenerateCmpdOntologyData($CmpdDataRef);
```

Return a reference to a hash containing ontology data with hash keys and values corresponding to property names and values.

GenerateCmpdOntologySDDataLines

```
$DataLinesArrayRef =
    GenerateCmpdOntologySDDataLines($CmpDataRef);
```

Return a reference to an array containing ontology data lines suitable for generate SD file data block.

GenerateSTStrData

```
($AtomLinesArrayRef, $BondLinesArrayRef) =
   GenerateSTStrData($CmpdDataRef);
```

Return array references containing atom and bond data lines for SD file. Appropriate atom and bond data lines are generated using abbreviation template data.

GenerateSDFile

```
GenerateSDFile($SDFileName, $CmdAbbrevsRef);
```

Generate a SD file for compound abbreviations. Structure data for specified abbreviation is generated sequentially and written to SD file.

IsSTAbbrevSupported

```
$Status = IsSTAbbrevSupported($Abbrev);
```

Return 1 or 0 based on whether ST abbreviation is supported.

IsSTSubstituentsNameSupported

```
$Status = IsSTSubstituentsNameSupported($SubstituentAbbrev);
```

Return 1 or 0 based on whether ST substituent abbreviation is supported.

IsSTDoubleBondsAbbrevOkay

```
$Status = IsSTDoubleBondsAbbrevOkay($STAbbrev, $STType,
   $SubstituentsAbbrev, $DoubleBondsAbbrev);
```

Return 1 or 0 based on whether ST double bond abbreviation is valid.

IsSTSubstituentsAbbrevOkay

```
$Status = IsSTSubstituentsAbbrevOkay($STAbbrev, $STType,
   $SubstituentsAbbrev, $DoubleBondsAbbrev);
```

Return 1 or 0 based on whether ST substituent abbreviation is valid.

IsWildCardInSTAbbrev

```
$Status = IsSTAbbrevSupported($Abbrev);
```

Return 1 or 0 based on whether ST abbreviation contains wild card.

ParseSTAbbrev

```
($STType,  $SubstituentsAbbrev, $DoubleBondsAbbrev) =
    ParseSTAbrev($Abbrev);
```

Parse ST abbreviation and return these values: STType, SubstituentsAbbrev, and DoubleBondsAbbrev.

ParseSTDoubleBondAbbrev

```
($BondPos1, $BondPos1) = ParseSTDoubleBondAbbrev($Abbrev);
```

Parse ST double bond abbreviation and return these values: BondPos1 and BondPos2.

ParseSTSubstituentAbbrev

```
($SubstituentPos, $SubstituentAbbrev, $StereoChemistry) =
   ParseSTSubstituentAbbrev($Abbrev);
```

Parse ST substituents abbreviation and return these values: SubstituentPos, SubstituentAbbrev,

and SubstituentStereoChemistry.

SetupSTCmpdAbbrevTemplateDataMap

```
$AbbrevTemplateDataMapRef =
   SetupSTCmpdAbbrevTemplateDataMap($Abbrev);
```

Return a reference to a hash containing template data for compound abbreviation. The template data is used to generate SD file for compound abbreviation.

ValidateSTAbbrev

```
$Status = ValidateSTAbbrev($Abbrev);
```

Return 1 or 0 based on whether a ST abbreviation is valid.

AUTHOR

Manish Sud

CONTRIBUTOR

Eoin Fahy

SEE ALSO

ChainStr.pm, LMAPSStr.pm

COPYRIGHT

Copyright (C) 2006-2012. The Regents of the University of California. All Rights Reserved.

LICENSE

Modified BSD License
