# Supplementary material for: The LUX Score: A Metric for Lipidome Homology
Source: PLoS Comput Biol. 2015 Sep 22;11(9):e1004511. doi: 10.1371/journal.pcbi.1004511 (PMC4578897; doi:10.1371/journal.pcbi.1004511)
Supplement: S5 Dataset — Includes scripts, README files and data files for Figs 1, 2, 6, 7 and S6. (ZIP) [file pcbi.1004511.s009.zip › S5_Dataset/Lipidome_Homology_Testing/bin/121010_lipidmapstools/docs/pdf/STStrGen.pdf]

NAME

STStrGen.pl - Generate structures for Sterols (ST)

SYNOPSIS

STStrGen.pl FAAbbrev|FAAbbrevFileName ...

STStrGen.pl [**-h, --help**] [**-o, --overwrite**] [**-r, --root** rootname] [**-w, --workingdir** dirname] <arguments>...

DESCRIPTION

Generate Strerol (ST) structures using compound abbreviations specified on a command line or in a CSV/TSV Text file. All the command line arguments represent either compound abbreviations or file name containing abbreviations. Use mode option to control the type of command line arguments.

A SD file, containing structures for all SP abbreviations along with ontological information, is generated as an output.

SUPPORTED ABBREVIATIONS

Current support for ST structure generation include these main classes and sub classes:

o Sterols

```
. Cholesterol and derivatives
. Ergosterols and C24-methyl derivatives
. Stigmasterols and C24-ethyl derivatives
```

OPTIONS

-h, --help

Print this help message

-m, --mode *Abbrev|AbbrevFileName*

Controls interpretation of command line arguments. Two different methods are provided: specify compound abbreviations or a file name containing compound abbreviations. Possible values: *Abbrev or AbbrevFileName*. Default: *Abbrev*

In *AbbrevFileName* mode, a single line in CSV/TSV files can contain multiple compound abbreviations. The file extension determines delimiter used to process data lines: comma for CSV and tab for TSV. For files with TXT extension, only one compound abbreviation per line is allowed.

Examples:

```
cholesterol: "CHOLESTANE(3,b,OH:5(6))"
5alpha-cholestane: "CHOLESTANE(5,a,H)"
25-hydroxy-cholesterol: "CHOLESTANE(3,b,OH/25,,OH:5(6))"
5,6beta-epoxy-cholesterol: "CHOLESTANE(3,b,OH/5,b,Ep)"
cholestenone: "CHOLESTANE(3,,Ke:4)"

ergost-5-en-3beta-ol: "ERGOSTANE(3,b,OH:5)"

campest-5-en-3beta-ol: "CAMPESTANE(3,b,OH:5)"

stigmast-5,25-dien-3beta-ol: "STIGMASTANE(3,b,OH:5/25(26))"
```

-o, --overwrite

Overwrite existing files

-r, --root *rootname*

New file name is generated using the root: <Root>.sdf. Default for new file names: STAbbrev.sdf, <AbbrevFilenName>.sdf, or <FirstAbbrevFileName>1To<Count>.sdf.

-w, --workingdir *dirname*

Location of working directory. Default: current directory

EXAMPLES

On some systems, command line scripts may need to be invoked using *perl -s STStrGen.pl*; however, all the examples assume direct invocation of command line script works.

To generate a STStructures.sdf file containing a structure specified by a command line abbreviation for cholesterol, type:

```
% STStrGen.pl -r STStructures -o "CHOLESTANE(3,b,OH:5(6))"
```

To generate a STStructures.sdf file containing a structure specified by a command line abbreviation for 5alpha-cholestane, type:

```
% STStrGen.pl -r STStructures -o "CHOLESTANE(5,a,H)"
```

To generate a STStructures.sdf file containing a structure specified by a command line abbreviation for 25-hydroxy-cholesterol, type:

```
% STStrGen.pl -r STStructures -o "CHOLESTANE(3,b,OH/25,,OH:5(6))"
```

To generate a STStructures.sdf file containing a structure specified by a command line abbreviation for 24S-hydroxy-cholesterol, type:

```
% STStrGen.pl -r STStructures -o "CHOLESTANE(3,b,OH/24,a,OH:5(6))"
```

To generate a STStructures.sdf file containing a structure specified by a command line abbreviation for 5,6beta-epoxy-cholesterol, type:

```
% STStrGen.pl -r STStructures -o "CHOLESTANE(3,b,OH/5,b,Ep)"
```

To generate a STStructures.sdf file containing a structure specified by a command line abbreviation for cholestenone, type:

```
% STStrGen.pl -r STStructures -o "CHOLESTANE(3,,Ke:4)"
```

To generate a STStructures.sdf file containing a structure specified by a command line abbreviation for desmostero;, type:

```
% STStrGen.pl -r STStructures -o "CHOLESTANE(3,b,OH:5/24)"
```

AUTHOR

Manish Sud

CONTRIBUTOR

Eoin Fahy

SEE ALSO

CLStrGen.pl, FAStrGen.pl, GLStrGen.pl, GPStrGen.pl, SPStrGen.pl

COPYRIGHT

Copyright (C) 2006-2012. The Regents of the University of California. All Rights Reserved.

LICENSE

Modified BSD License
