# Supplementary material for: The LUX Score: A Metric for Lipidome Homology
Source: PLoS Comput Biol. 2015 Sep 22;11(9):e1004511. doi: 10.1371/journal.pcbi.1004511 (PMC4578897; doi:10.1371/journal.pcbi.1004511)
Supplement: S5 Dataset — Includes scripts, README files and data files for Figs 1, 2, 6, 7 and S6. (ZIP) [file pcbi.1004511.s009.zip › S5_Dataset/Lipidome_Homology_Testing/DM_Result/Dendrograms.pdf]

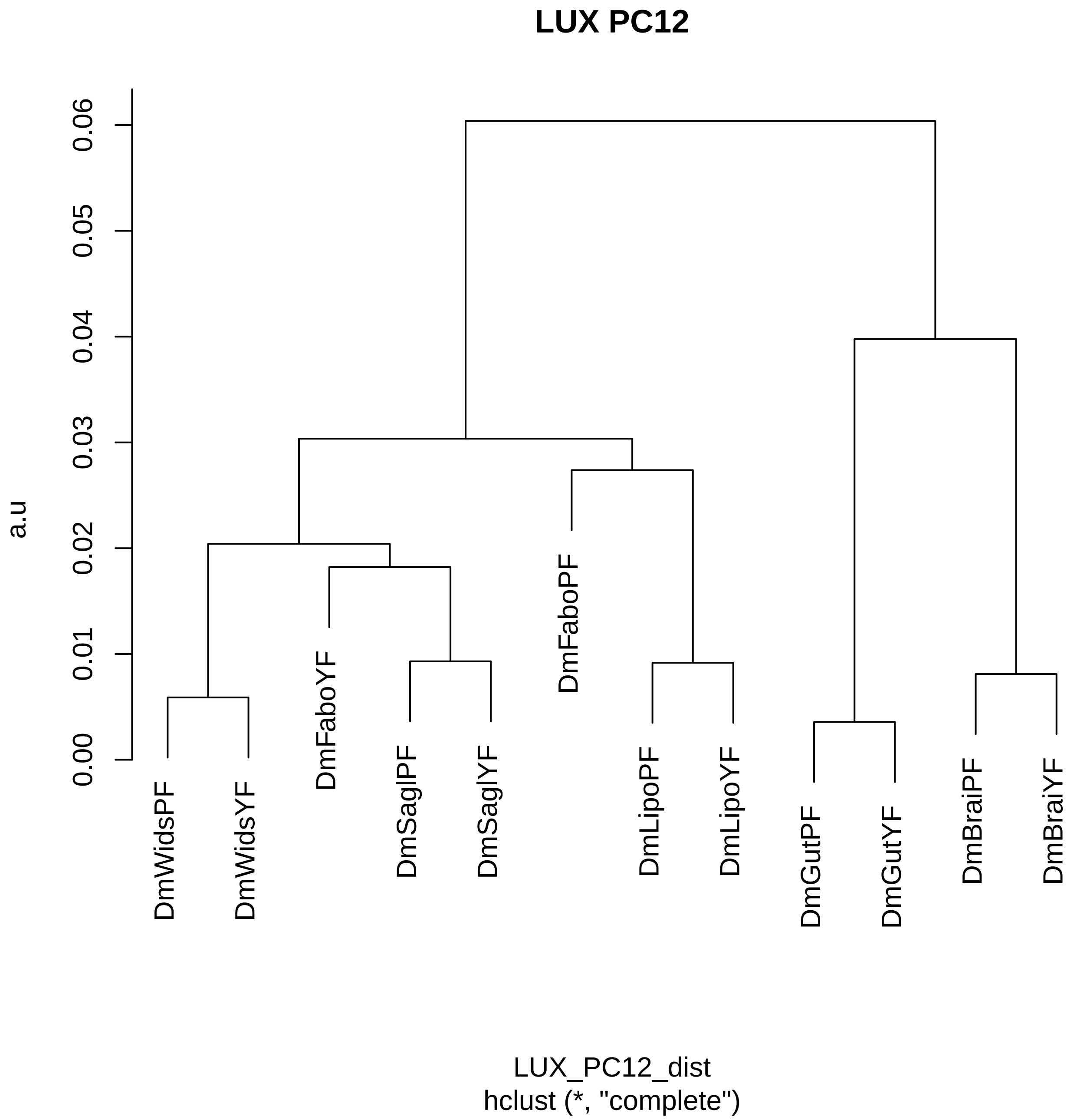
LUX PC12
a.u
0.00
0.01
0.02
0.03
0.04
0.05
0.06
DmWidsPF
DmWidsYF
DmFaboYF
DmSaglPF
DmSaglYF
DmFaboPF
DmLipoPF
DmLipoYF
DmGutPF
DmGutYF
DmBraiPF
DmBraiYF
LUX_PC12_dist
hclust (*, "complete")

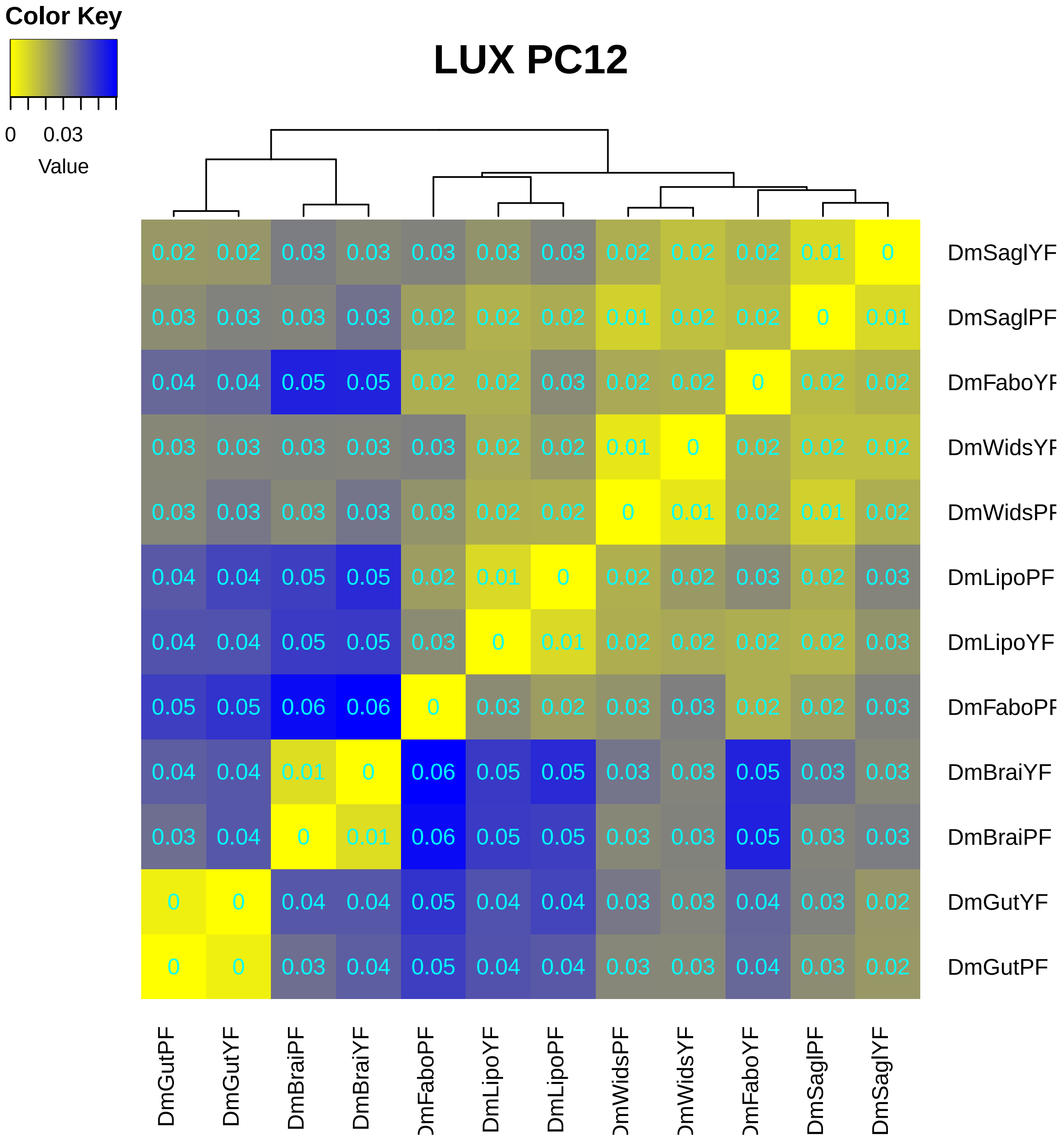
LUX PC12
Color Key
0 0.03
Value
0.02 0.02 0.03 0.03 0.03 0.03 0.03 0.02 0.02 0.02 0.01 0 DmSaglYF
0.03 0.03 0.03 0.03 0.02 0.02 0.02 0.01 0.02 0.02 0 0.01 DmSaglPF
0.04 0.04 0.05 0.05 0.02 0.02 0.03 0.02 0.02 0 0.02 0.02 DmFaboYF
0.03 0.03 0.03 0.03 0.03 0.02 0.02 0.01 0 0.02 0.02 0.02 DmWidsYF
0.03 0.03 0.03 0.03 0.03 0.02 0.02 0 0.01 0.02 0.01 0.02 DmWidsPF
0.04 0.04 0.05 0.05 0.02 0.01 0 0.02 0.02 0.03 0.02 0.03 DmLipoPF
0.04 0.04 0.05 0.05 0.03 0 0.01 0.02 0.02 0.02 0.02 0.03 DmLipoYF
0.05 0.05 0.06 0.06 0 0.03 0.02 0.03 0.03 0.02 0.02 0.03 DmFaboPF
0.04 0.04 0.01 0 0.06 0.05 0.05 0.03 0.03 0.05 0.03 0.03 DmBraiYF
0.03 0.04 0 0.01 0.06 0.05 0.05 0.03 0.03 0.05 0.03 0.03 DmBraiPF
0 0 0.04 0.04 0.05 0.04 0.04 0.03 0.03 0.04 0.03 0.02 DmGutYF
0 0 0.03 0.04 0.05 0.04 0.04 0.03 0.03 0.04 0.03 0.02 DmGutPF
DmGutPF DmGutYF DmBraiPF DmBraiYF DmFaboPF DmLipoYF DmLipoPF DmWidsPF DmWidsYF DmFaboYF DmSaglPF DmSaglYF

LUX PC123

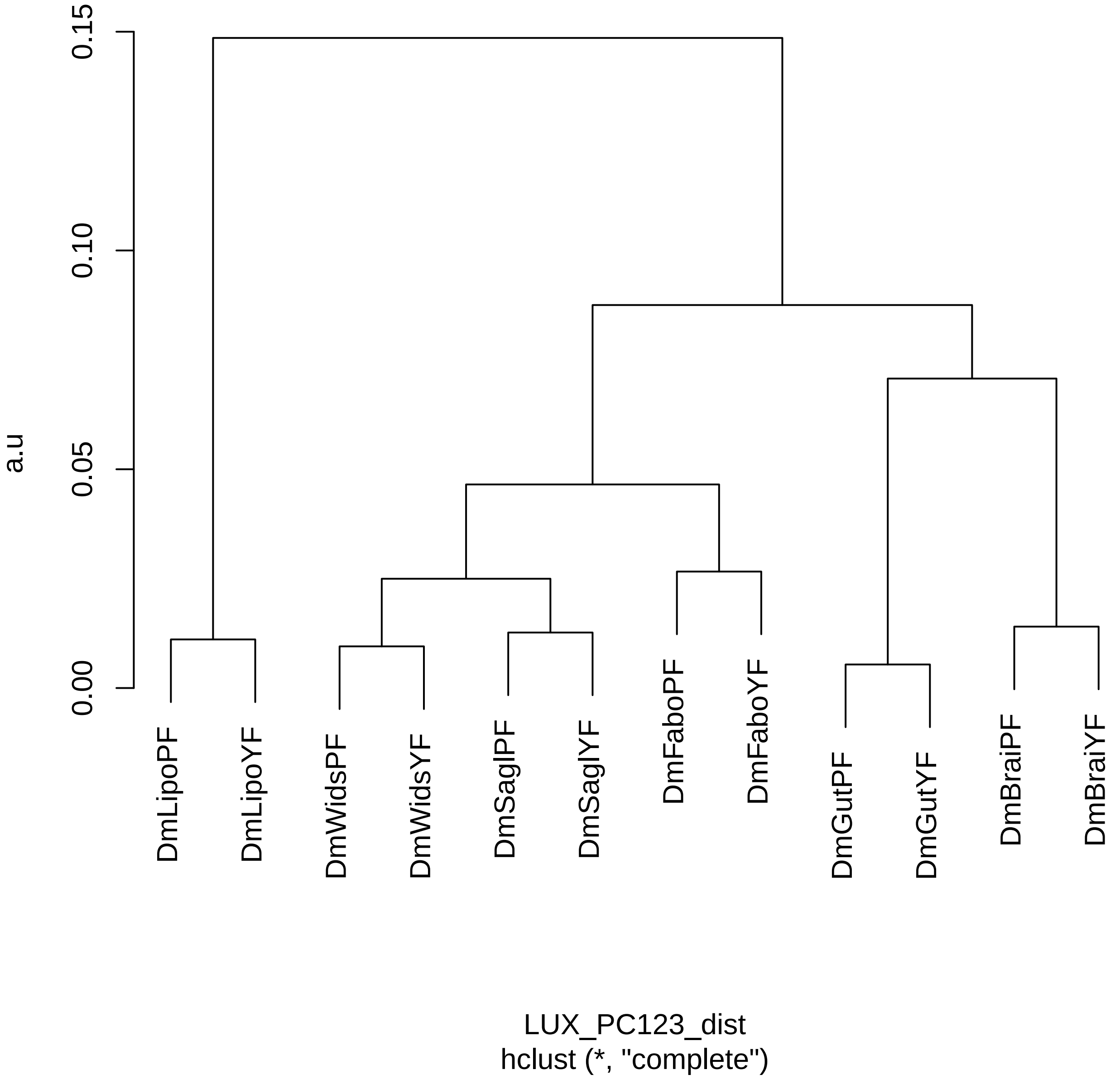
a.u
0.00
0.05
0.10
0.15
DmLipoPF
DmLipoYF
DmWidsPF
DmWidsYF
DmSaglPF
DmSaglYF
DmFaboPF
DmFaboYF
DmGutPF
DmGutYF
DmBraiPF
DmBraiYF
LUX_PC123_dist
hclust (*, "complete")

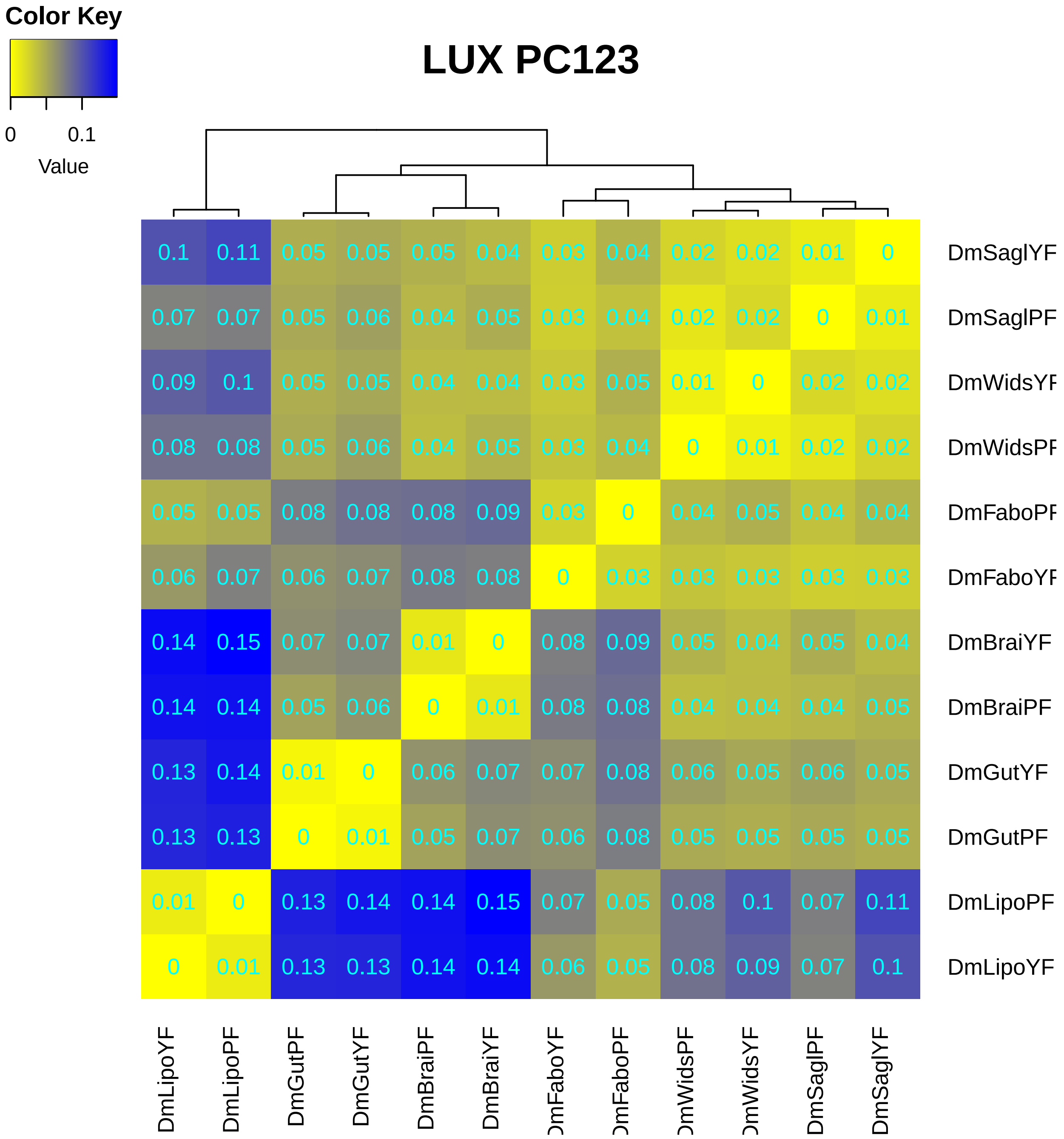

LUX PC123
Color Key
0
0.1
Value
DmSaglYF
DmSaglPF
DmWidsYF
DmWidsPF
DmFaboPF
DmFaboYF
DmBraiYF
DmBraiPF
DmGutYF
DmGutPF
DmLipoPF
DmLipoYF
DmLipoYF
DmLipoPF
DmGutPF
DmGutYF
DmBraiPF
DmBraiYF
DmFaboYF
DmFaboPF
DmWidsPF
DmWidsYF
DmSaglPF
DmSaglYF
0.1 0.11 0.05 0.05 0.05 0.04 0.03 0.04 0.02 0.02 0.01 0
0.07 0.07 0.05 0.06 0.04 0.05 0.03 0.04 0.02 0.02 0 0.01
0.09 0.1 0.05 0.05 0.04 0.04 0.03 0.05 0.01 0 0.02 0.02
0.08 0.08 0.05 0.06 0.04 0.05 0.03 0.04 0 0.01 0.02 0.02
0.05 0.05 0.08 0.08 0.08 0.09 0.03 0 0.04 0.05 0.04 0.04
0.06 0.07 0.06 0.07 0.08 0.08 0 0.03 0.03 0.03 0.03 0.03
0.14 0.15 0.07 0.07 0.01 0 0.08 0.09 0.05 0.04 0.05 0.04
0.14 0.14 0.05 0.06 0 0.01 0.08 0.08 0.04 0.04 0.04 0.05
0.13 0.14 0.01 0 0.06 0.07 0.07 0.08 0.06 0.05 0.06 0.05
0.13 0.13 0 0.01 0.05 0.07 0.06 0.08 0.05 0.05 0.05 0.05
0.01 0 0.13 0.14 0.14 0.15 0.07 0.05 0.08 0.1 0.07 0.11
0 0.01 0.13 0.13 0.14 0.14 0.06 0.05 0.08 0.09 0.07 0.1

LUX PC123

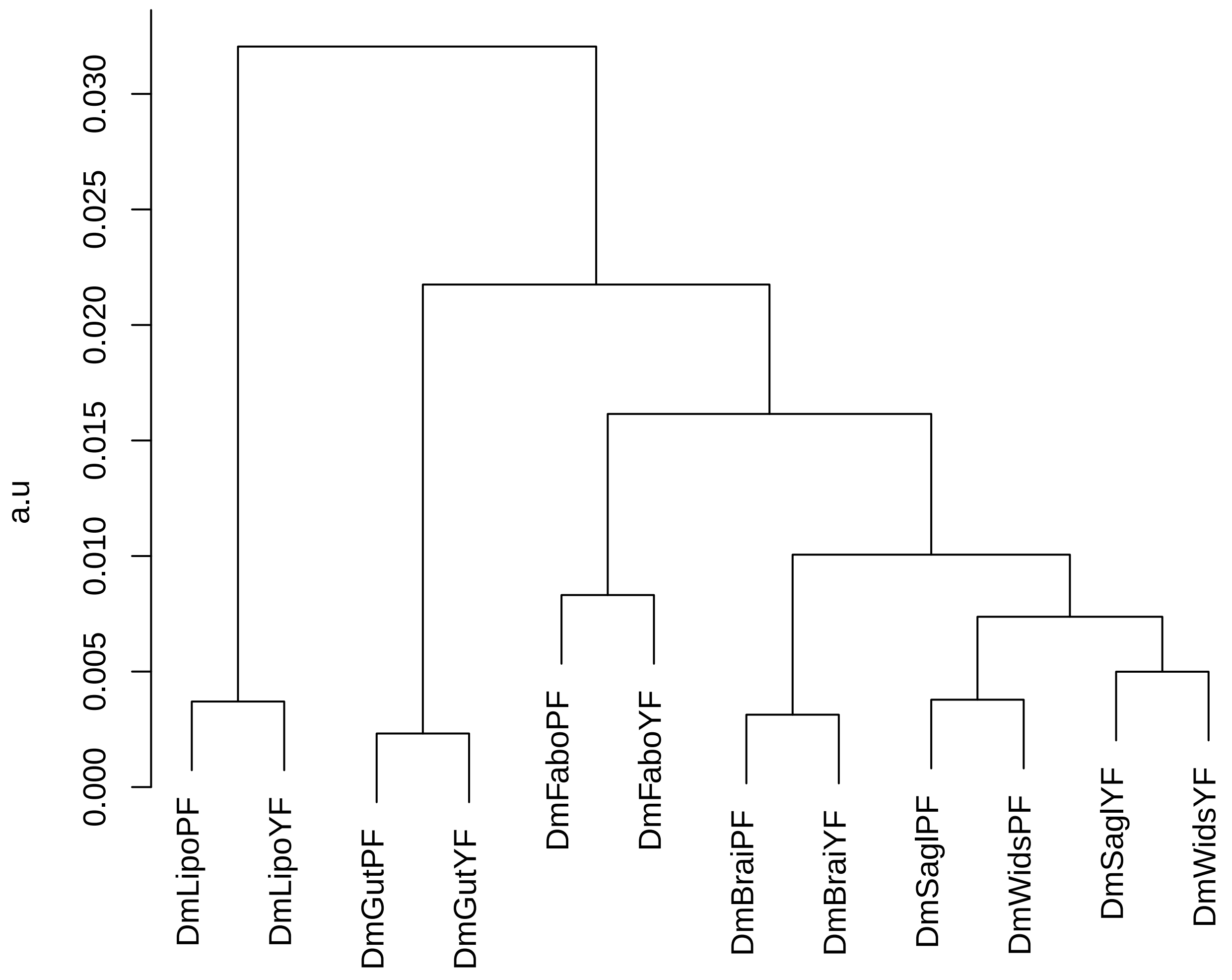

LUX_SS_dist
hclust (*, "complete")

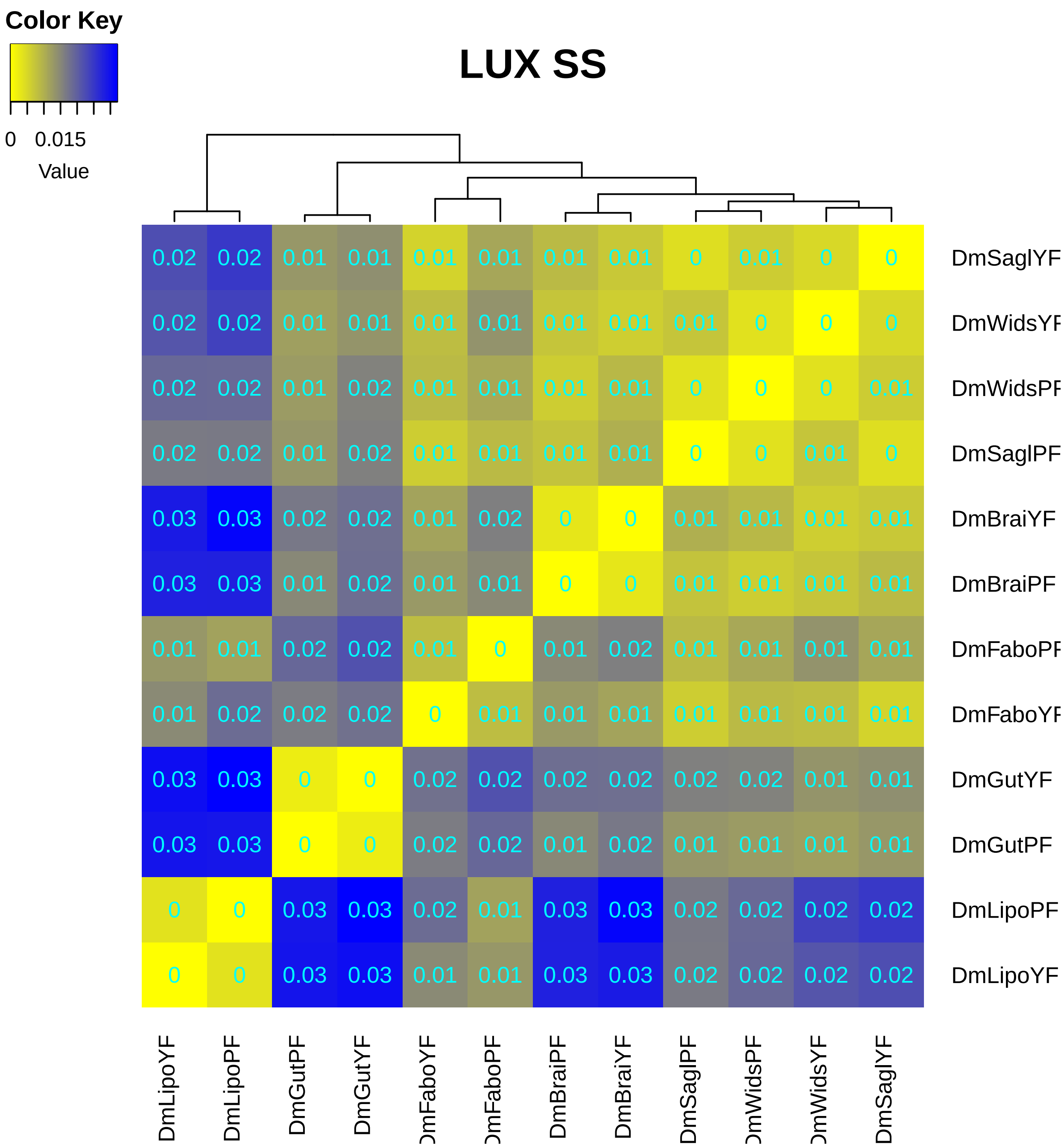

Color Key
0
0.015
Value
LUX SS
DmSaglYF 0.02 0.02 0.01 0.01 0.01 0.01 0.01 0.01 0 0.01 0 0
DmWidsYF 0.02 0.02 0.01 0.01 0.01 0.01 0.01 0.01 0.01 0 0 0
DmWidsPF 0.02 0.02 0.01 0.02 0.01 0.01 0.01 0.01 0 0 0 0.01
DmSaglPF 0.02 0.02 0.01 0.02 0.01 0.01 0.01 0.01 0 0 0.01 0
DmBraiYF 0.03 0.03 0.02 0.02 0.01 0.02 0 0 0.01 0.01 0.01 0.01
DmBraiPF 0.03 0.03 0.01 0.02 0.01 0.01 0 0 0.01 0.01 0.01 0.01
DmFaboPF 0.01 0.01 0.02 0.02 0.01 0 0.01 0.02 0.01 0.01 0.01 0.01
DmFaboYF 0.01 0.02 0.02 0.02 0 0.01 0.01 0.01 0.01 0.01 0.01 0.01
DmGutYF 0.03 0.03 0 0 0.02 0.02 0.02 0.02 0.02 0.02 0.01 0.01
DmGutPF 0.03 0.03 0 0 0.02 0.02 0.01 0.02 0.01 0.01 0.01 0.01
DmLipoPF 0 0 0.03 0.03 0.02 0.01 0.03 0.03 0.02 0.02 0.02 0.02
DmLipoYF 0 0 0.03 0.03 0.01 0.01 0.03 0.03 0.02 0.02 0.02 0.02
DmLipoYF
DmLipoPF
DmGutPF
DmGutYF
DmFaboYF
DmFaboPF
DmBraiPF
DmBraiYF
DmSaglPF
DmWidsPF
DmWidsYF
DmSaglYF

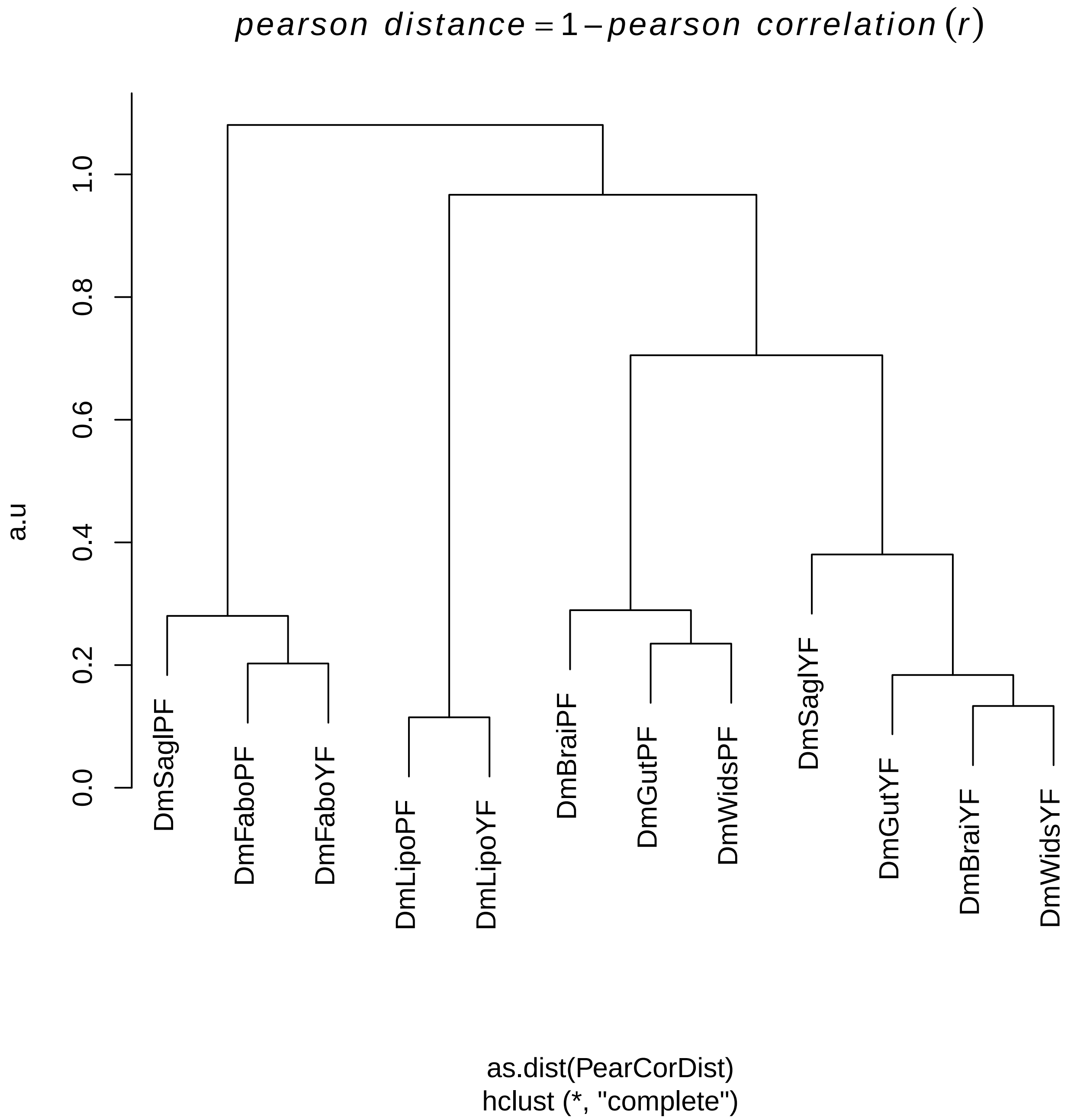

$pearson\ distance = 1 - pearson\ correlation\,(r)$
a.u
0.0
0.2
0.4
0.6
0.8
1.0
DmSaglPF
DmFaboPF
DmFaboYF
DmLipoPF
DmLipoYF
DmBraiPF
DmGutPF
DmWidsPF
DmSaglYF
DmGutYF
DmBraiYF
DmWidsYF
as.dist(PearCorDist)
hclust (*, "complete")

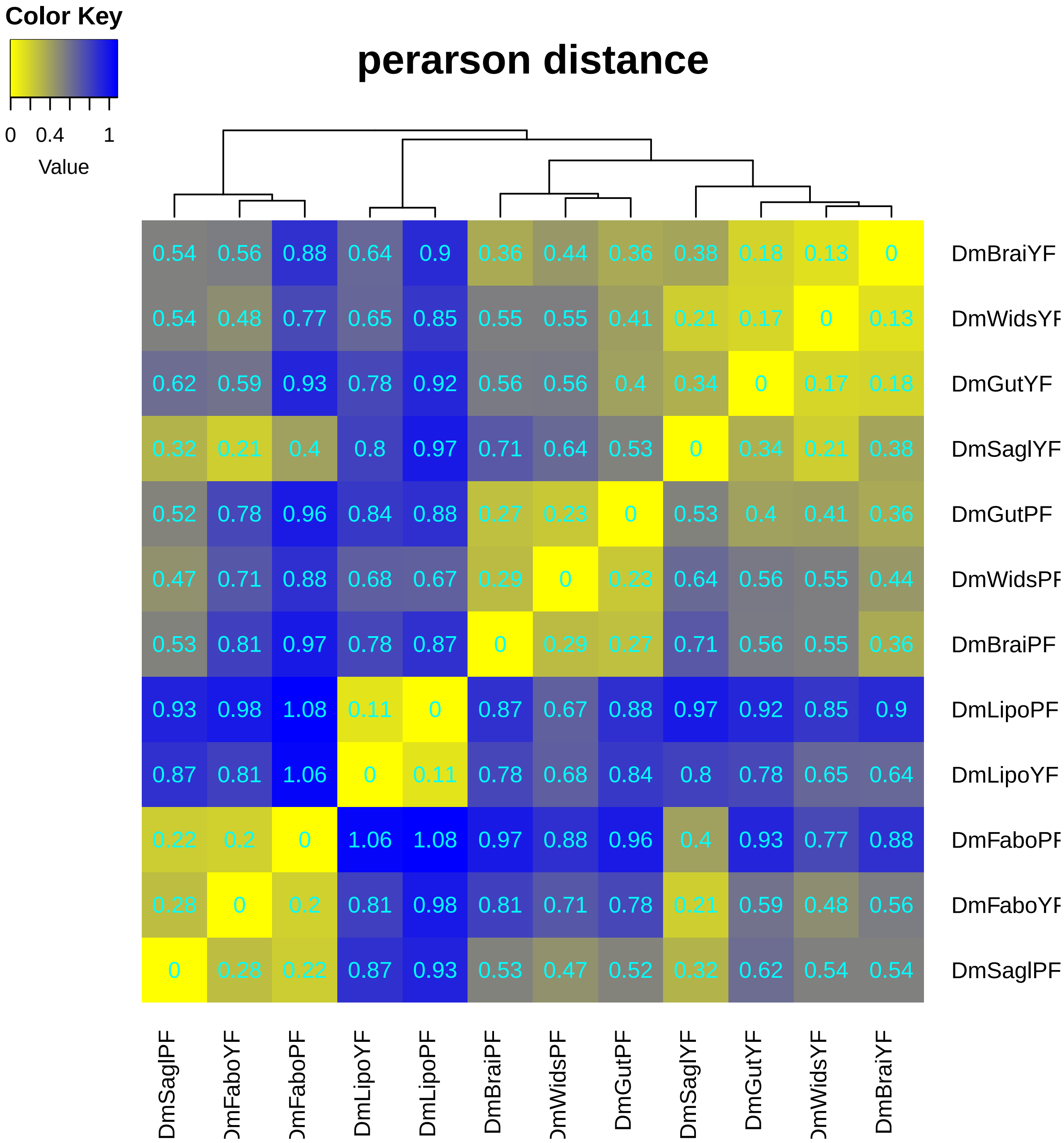

Color Key
0 0.4 1
Value
perarson distance
DmBraiYF
DmWidsYF
DmGutYF
DmSaglYF
DmGutPF
DmWidsPF
DmBraiPF
DmLipoPF
DmLipoYF
DmFaboPF
DmFaboYF
DmSaglPF
0.54 0.56 0.88 0.64 0.9 0.36 0.44 0.36 0.38 0.18 0.13 0
0.54 0.48 0.77 0.65 0.85 0.55 0.55 0.41 0.21 0.17 0 0.13
0.62 0.59 0.93 0.78 0.92 0.56 0.56 0.4 0.34 0 0.17 0.18
0.32 0.21 0.4 0.8 0.97 0.71 0.64 0.53 0 0.34 0.21 0.38
0.52 0.78 0.96 0.84 0.88 0.27 0.23 0 0.53 0.4 0.41 0.36
0.47 0.71 0.88 0.68 0.67 0.29 0 0.23 0.64 0.56 0.55 0.44
0.53 0.81 0.97 0.78 0.87 0 0.29 0.27 0.71 0.56 0.55 0.36
0.93 0.98 1.08 0.11 0 0.87 0.67 0.88 0.97 0.92 0.85 0.9
0.87 0.81 1.06 0 0.11 0.78 0.68 0.84 0.8 0.78 0.65 0.64
0.22 0.2 0 1.06 1.08 0.97 0.88 0.96 0.4 0.93 0.77 0.88
0.28 0 0.2 0.81 0.98 0.81 0.71 0.78 0.21 0.59 0.48 0.56
0 0.28 0.22 0.87 0.93 0.53 0.47 0.52 0.32 0.62 0.54 0.54
DmSaglPF
DmLipoYF
DmLipoPF
DmBraiPF
DmGutPF
DmSaglYF
DmGutYF
DmBraiYF

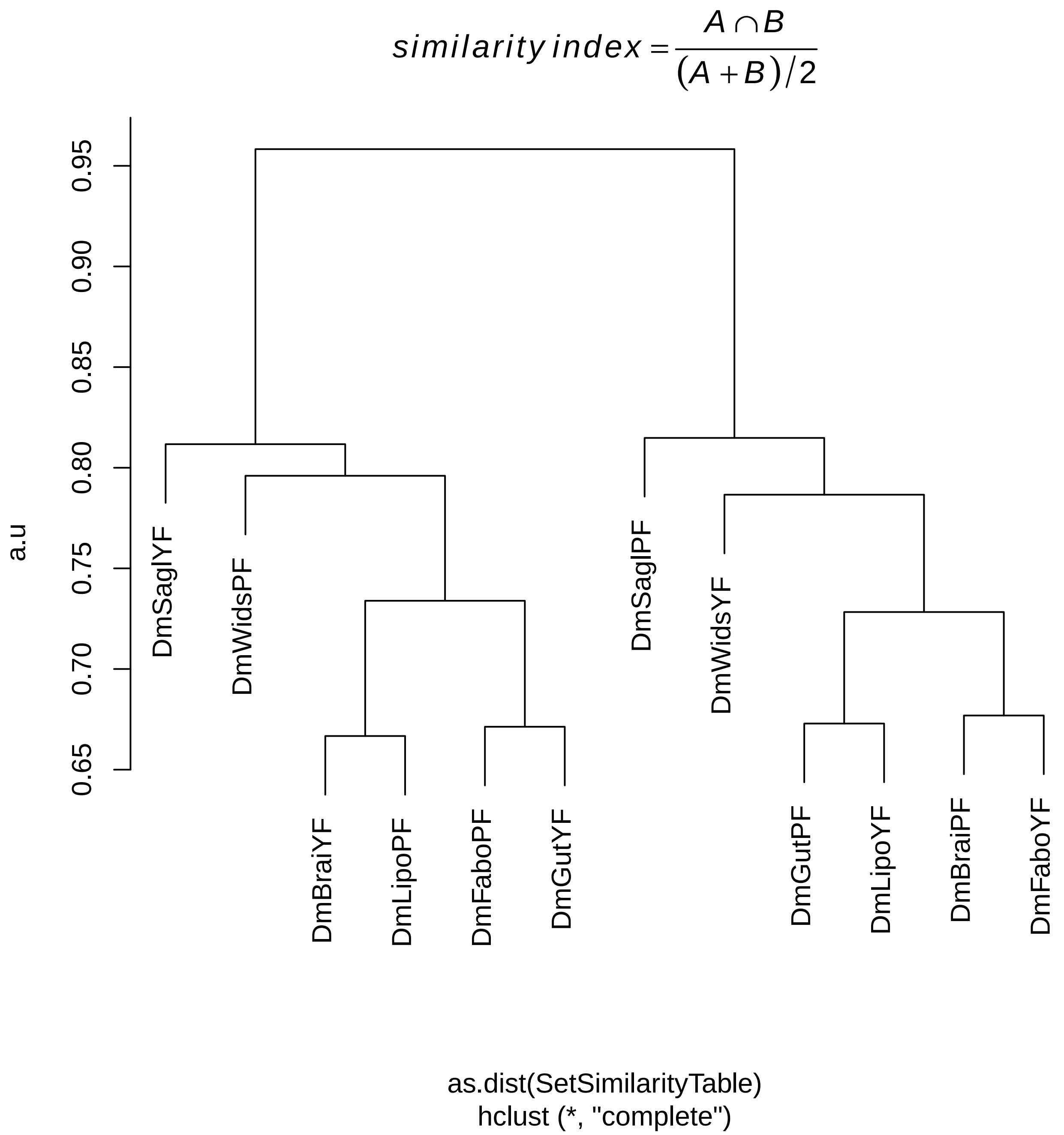

$$similarity\ index = \frac{A \cap B}{(A+B)/2}$$
a.u
0.65
0.70
0.75
0.80
0.85
0.90
0.95
DmSaglYF
DmWidsPF
DmBraiYF
DmLipoPF
DmFaboPF
DmGutYF
DmSaglPF
DmWidsYF
DmGutPF
DmLipoYF
DmBraiPF
DmFaboYF
as.dist(SetSimilarityTable)
hclust (*, "complete")

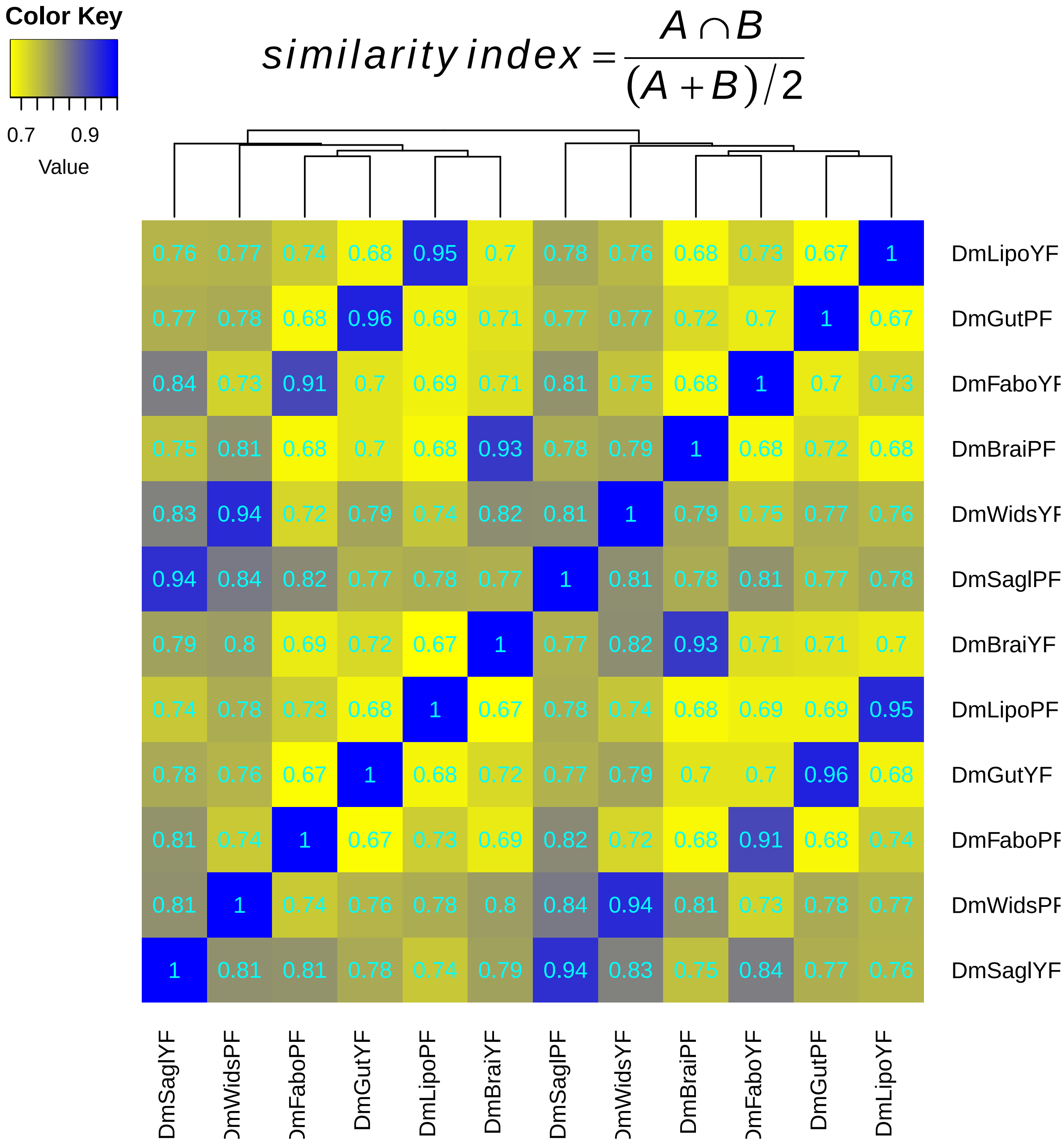
Color Key
0.7 0.9
Value
$$similarity\ index = \frac{A \cap B}{(A+B)/2}$$
DmLipoYF: 0.76 0.77 0.74 0.68 0.95 0.7 0.78 0.76 0.68 0.73 0.67 1
DmGutPF: 0.77 0.78 0.68 0.96 0.69 0.71 0.77 0.77 0.72 0.7 1 0.67
DmFaboYF: 0.84 0.73 0.91 0.7 0.69 0.71 0.81 0.75 0.68 1 0.7 0.73
DmBraiPF: 0.75 0.81 0.68 0.7 0.68 0.93 0.78 0.79 1 0.68 0.72 0.68
DmWidsYF: 0.83 0.94 0.72 0.79 0.74 0.82 0.81 1 0.79 0.75 0.77 0.76
DmSaglPF: 0.94 0.84 0.82 0.77 0.78 0.77 1 0.81 0.78 0.81 0.77 0.78
DmBraiYF: 0.79 0.8 0.69 0.72 0.67 1 0.77 0.82 0.93 0.71 0.71 0.7
DmLipoPF: 0.74 0.78 0.73 0.68 1 0.67 0.78 0.74 0.68 0.69 0.69 0.95
DmGutYF: 0.78 0.76 0.67 1 0.68 0.72 0.77 0.79 0.7 0.7 0.96 0.68
DmFaboPF: 0.81 0.74 1 0.67 0.73 0.69 0.82 0.72 0.68 0.91 0.68 0.74
DmWidsPF: 0.81 1 0.74 0.76 0.78 0.8 0.84 0.94 0.81 0.73 0.78 0.77
DmSaglYF: 1 0.81 0.81 0.78 0.74 0.79 0.94 0.83 0.75 0.84 0.77 0.76
DmSaglYF DmWidsPF DmFaboPF DmGutYF DmLipoPF DmBraiYF DmSaglPF DmWidsYF DmBraiPF DmFaboYF DmGutPF DmLipoYF

$$distance\ index = 1 - \frac{A \cap B}{(A + B)/2}$$

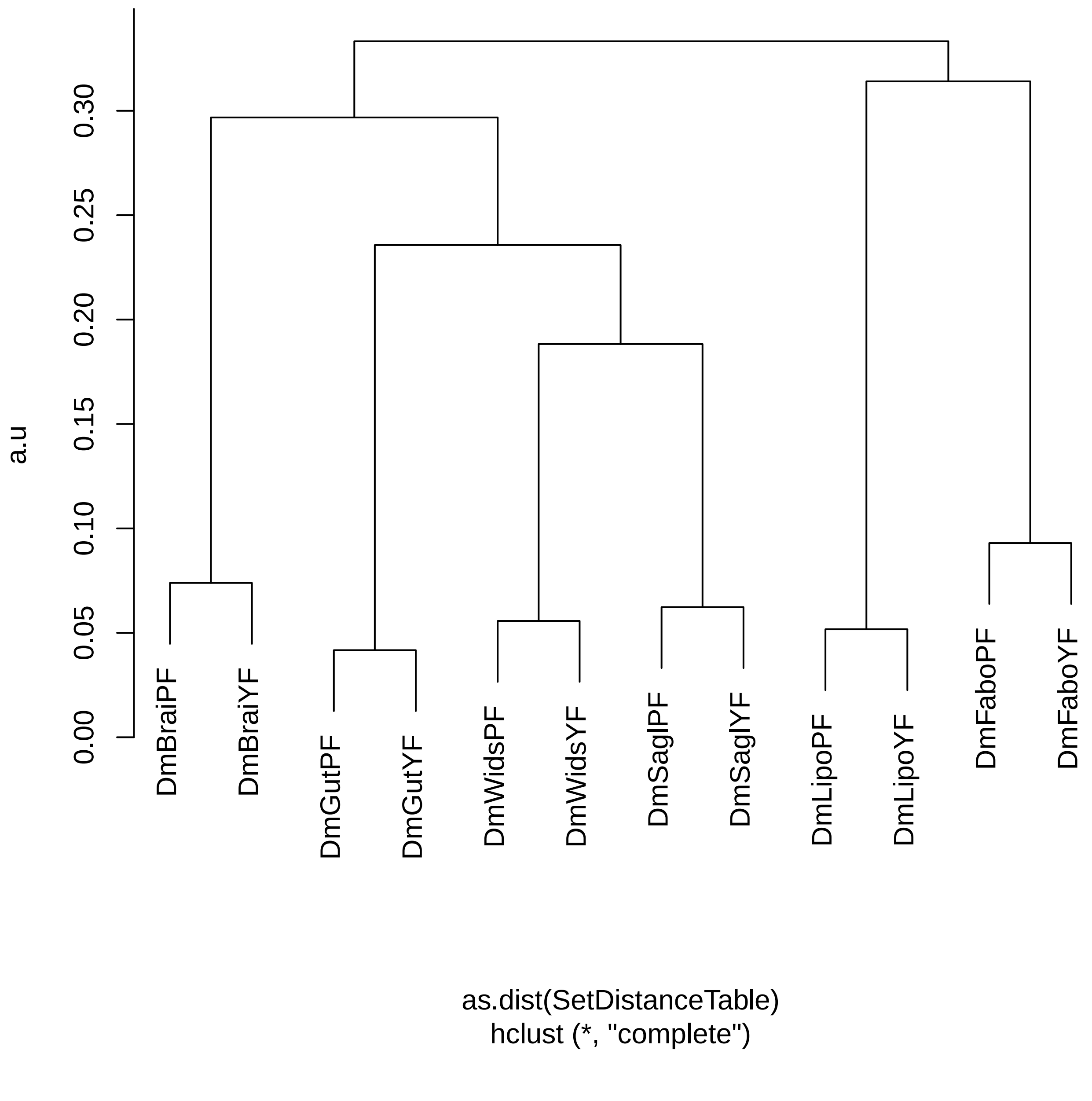

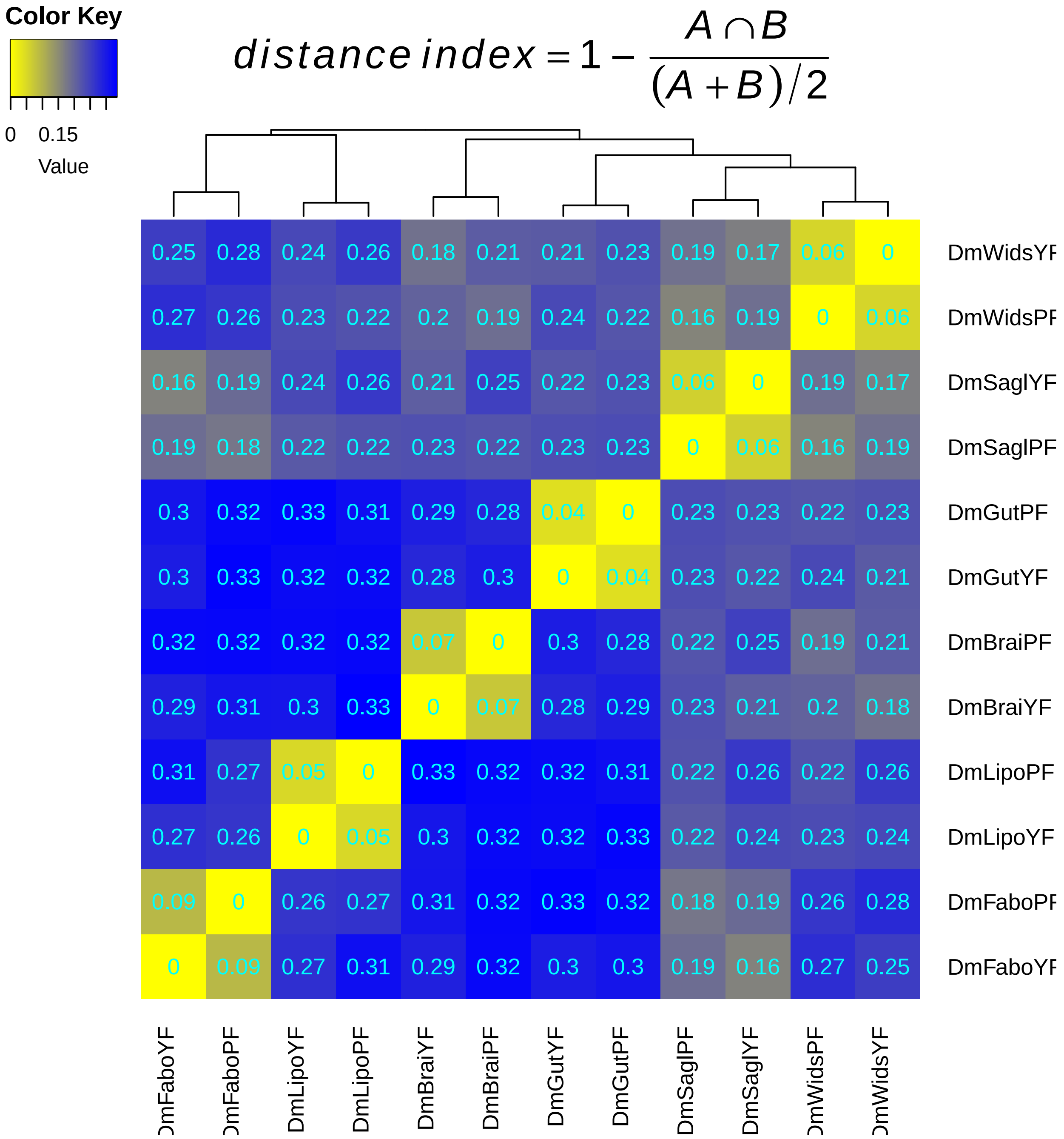
Color Key
0 0.15
Value
$distance\ index = 1 - \frac{A \cap B}{(A+B)/2}$
DmWidsY 0.25 0.28 0.24 0.26 0.18 0.21 0.21 0.23 0.19 0.17 0.06 0
DmWidsP 0.27 0.26 0.23 0.22 0.2 0.19 0.24 0.22 0.16 0.19 0 0.06
DmSaglYF 0.16 0.19 0.24 0.26 0.21 0.25 0.22 0.23 0.06 0 0.19 0.17
DmSaglPF 0.19 0.18 0.22 0.22 0.23 0.22 0.23 0.23 0 0.06 0.16 0.19
DmGutPF 0.3 0.32 0.33 0.31 0.29 0.28 0.04 0 0.23 0.23 0.22 0.23
DmGutYF 0.3 0.33 0.32 0.32 0.28 0.3 0 0.04 0.23 0.22 0.24 0.21
DmBraiPF 0.32 0.32 0.32 0.32 0.07 0 0.3 0.28 0.22 0.25 0.19 0.21
DmBraiYF 0.29 0.31 0.3 0.33 0 0.07 0.28 0.29 0.23 0.21 0.2 0.18
DmLipoPF 0.31 0.27 0.05 0 0.33 0.32 0.32 0.31 0.22 0.26 0.22 0.26
DmLipoYF 0.27 0.26 0 0.05 0.3 0.32 0.32 0.33 0.22 0.24 0.23 0.24
DmFaboP 0.09 0 0.26 0.27 0.31 0.32 0.33 0.32 0.18 0.19 0.26 0.28
DmFaboY 0 0.09 0.27 0.31 0.29 0.32 0.3 0.3 0.19 0.16 0.27 0.25
mFaboYF
mFaboPF
DmLipoYF
DmLipoPF
DmBraiYF
DmBraiPF
DmGutYF
DmGutPF
DmSaglPF
DmSaglYF
mWidsPF
mWidsYF
