# Supplementary material for: The LUX Score: A Metric for Lipidome Homology
Source: PLoS Comput Biol. 2015 Sep 22;11(9):e1004511. doi: 10.1371/journal.pcbi.1004511 (PMC4578897; doi:10.1371/journal.pcbi.1004511)
Supplement: S5 Dataset — Includes scripts, README files and data files for Figs 1, 2, 6, 7 and S6. (ZIP) [file pcbi.1004511.s009.zip › S5_Dataset/Lipidome_Homology_Testing/SC/Dendrograms.pdf]

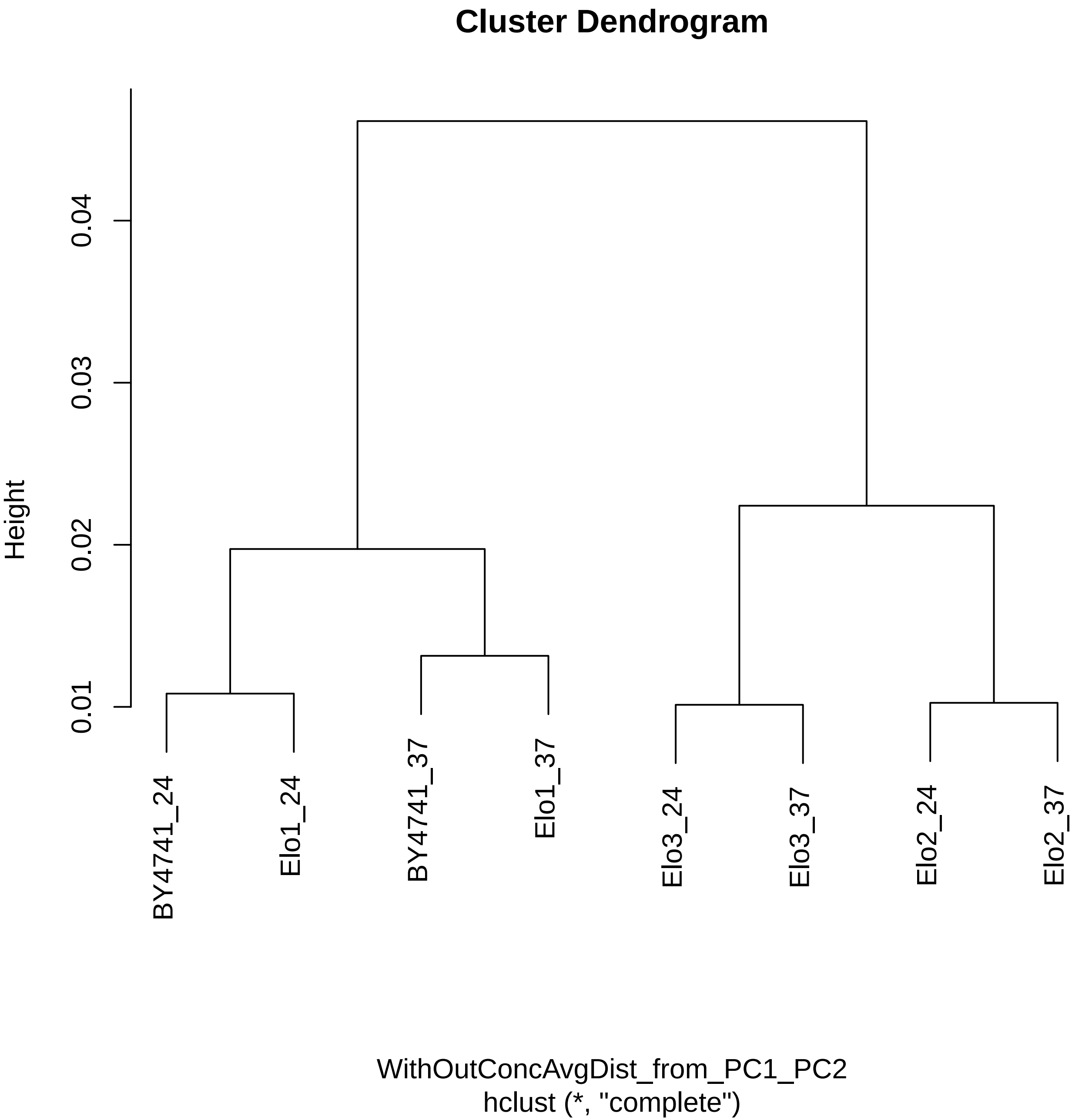
Cluster Dendrogram
Height
0.01
0.02
0.03
0.04
BY4741_24
Elo1_24
BY4741_37
Elo1_37
Elo3_24
Elo3_37
Elo2_24
Elo2_37
WithOutConcAvgDist_from_PC1_PC2
hclust (*, "complete")

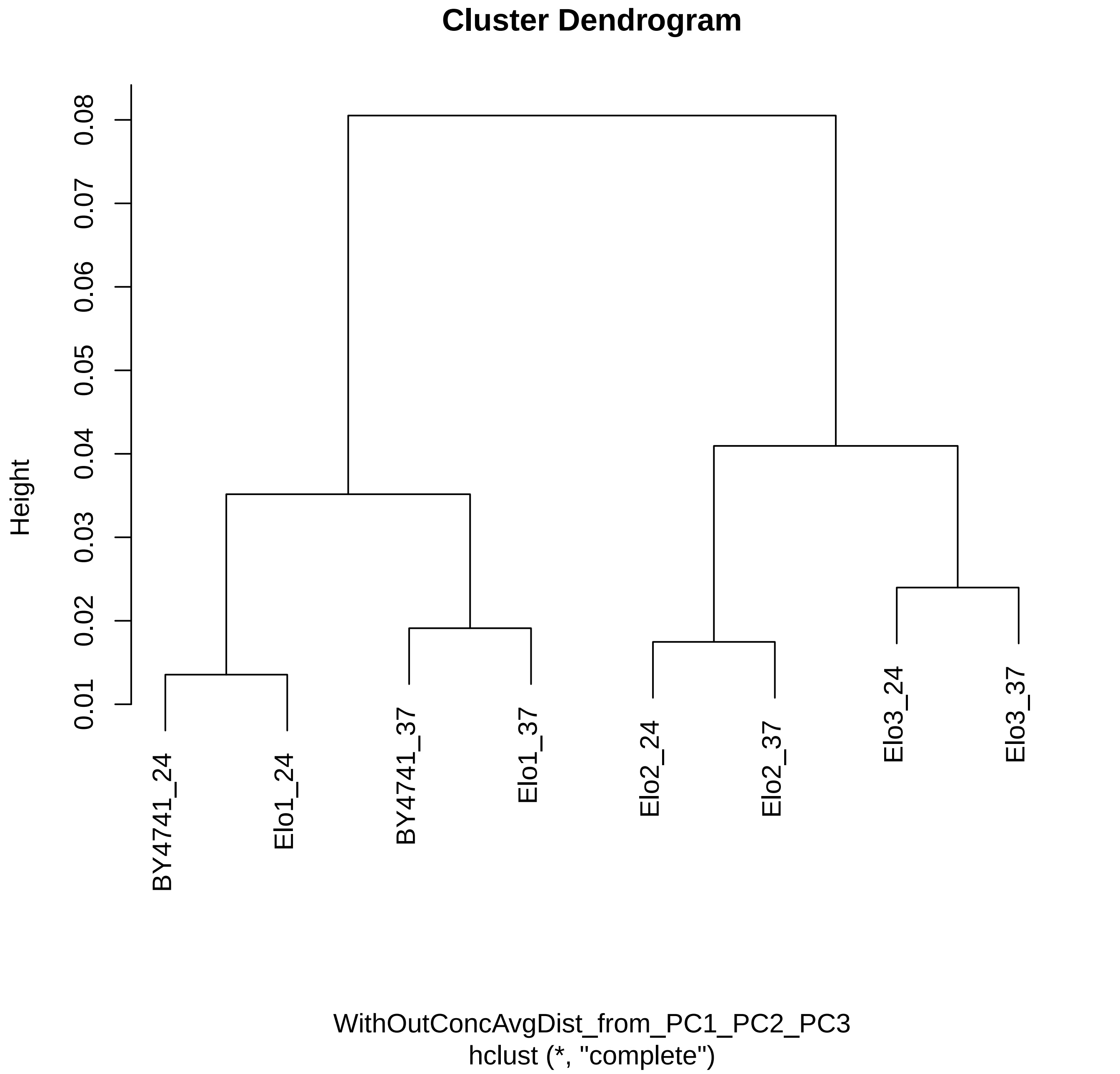
Cluster Dendrogram
Height
0.08
0.07
0.06
0.05
0.04
0.03
0.02
0.01
BY4741_24
Elo1_24
BY4741_37
Elo1_37
Elo2_24
Elo2_37
Elo3_24
Elo3_37
WithOutConcAvgDist_from_PC1_PC2_PC3
hclust (*, "complete")

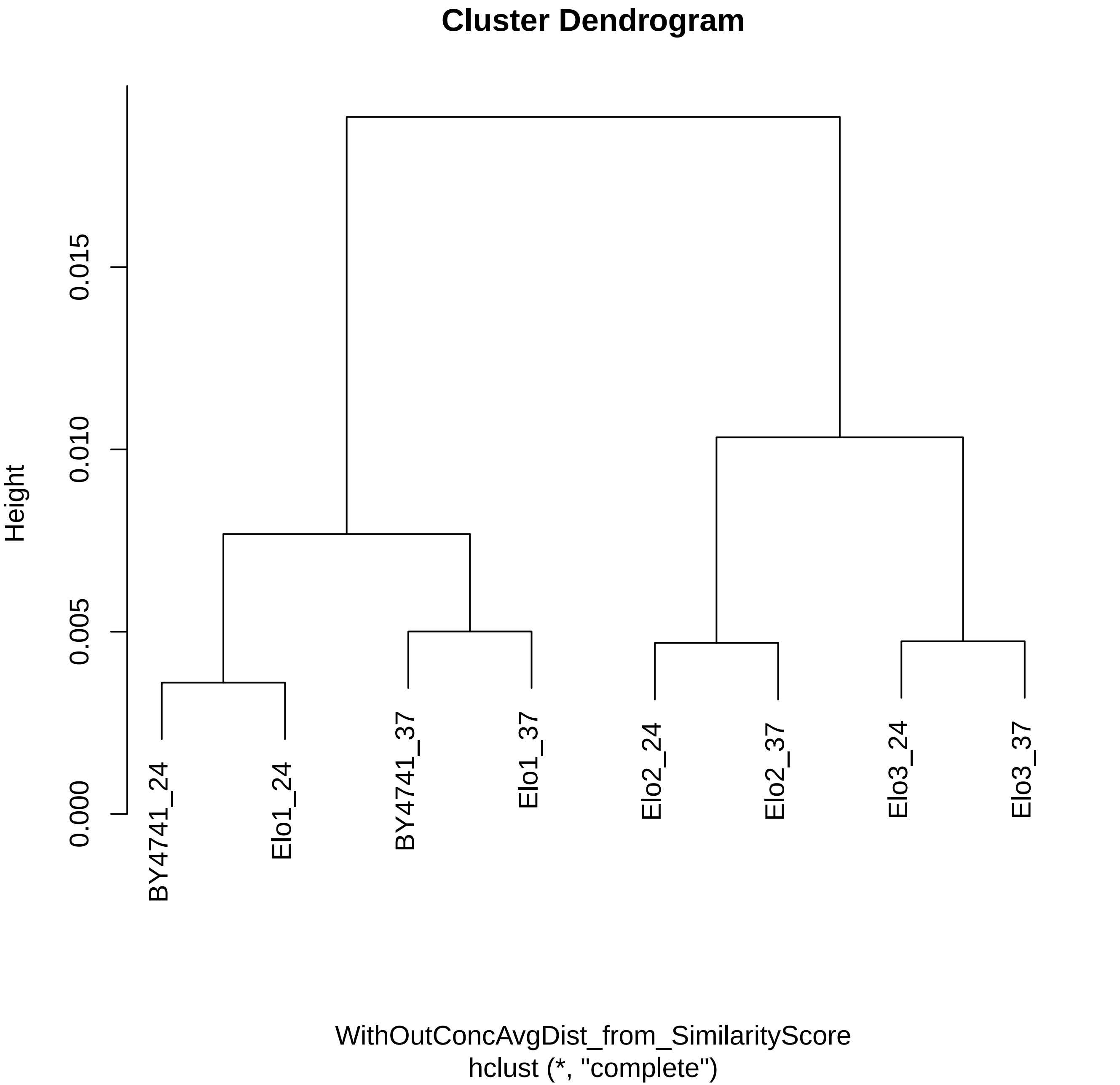
Cluster Dendrogram
Height
0.000
0.005
0.010
0.015
BY4741_24
Elo1_24
BY4741_37
Elo1_37
Elo2_24
Elo2_37
Elo3_24
Elo3_37
WithOutConcAvgDist_from_SimilarityScore
hclust (*, "complete")
