# Supplementary material for: The LUX Score: A Metric for Lipidome Homology
Source: PLoS Comput Biol. 2015 Sep 22;11(9):e1004511. doi: 10.1371/journal.pcbi.1004511 (PMC4578897; doi:10.1371/journal.pcbi.1004511)
Supplement: S5 Dataset — Includes scripts, README files and data files for Figs 1, 2, 6, 7 and S6. (ZIP) [file pcbi.1004511.s009.zip › S5_Dataset/Lipidome_Homology_Testing/SC_Result/Dendrograms.pdf]

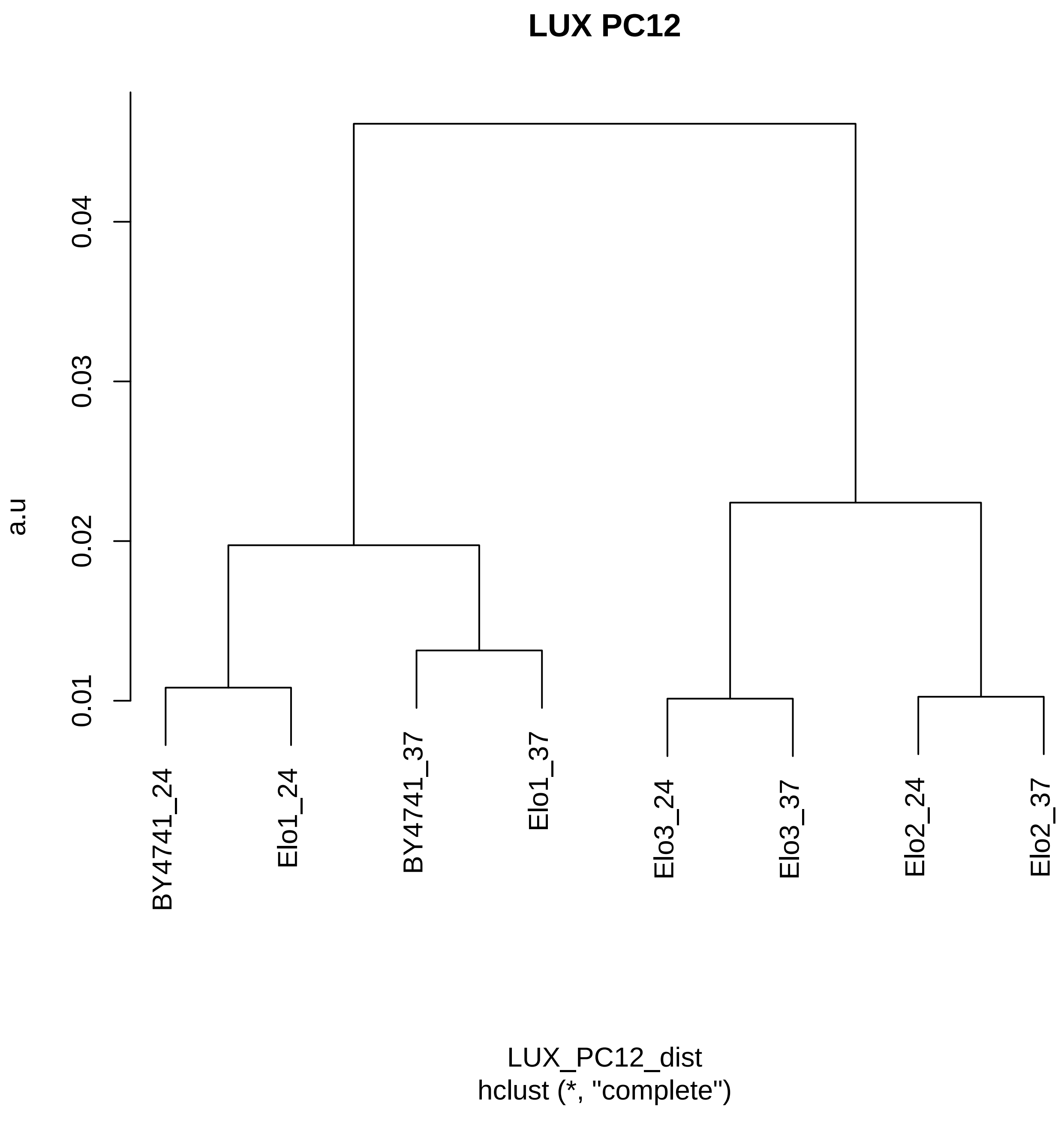
LUX PC12
a.u
0.01
0.02
0.03
0.04
BY4741_24
Elo1_24
BY4741_37
Elo1_37
Elo3_24
Elo3_37
Elo2_24
Elo2_37
LUX_PC12_dist
hclust (*, "complete")

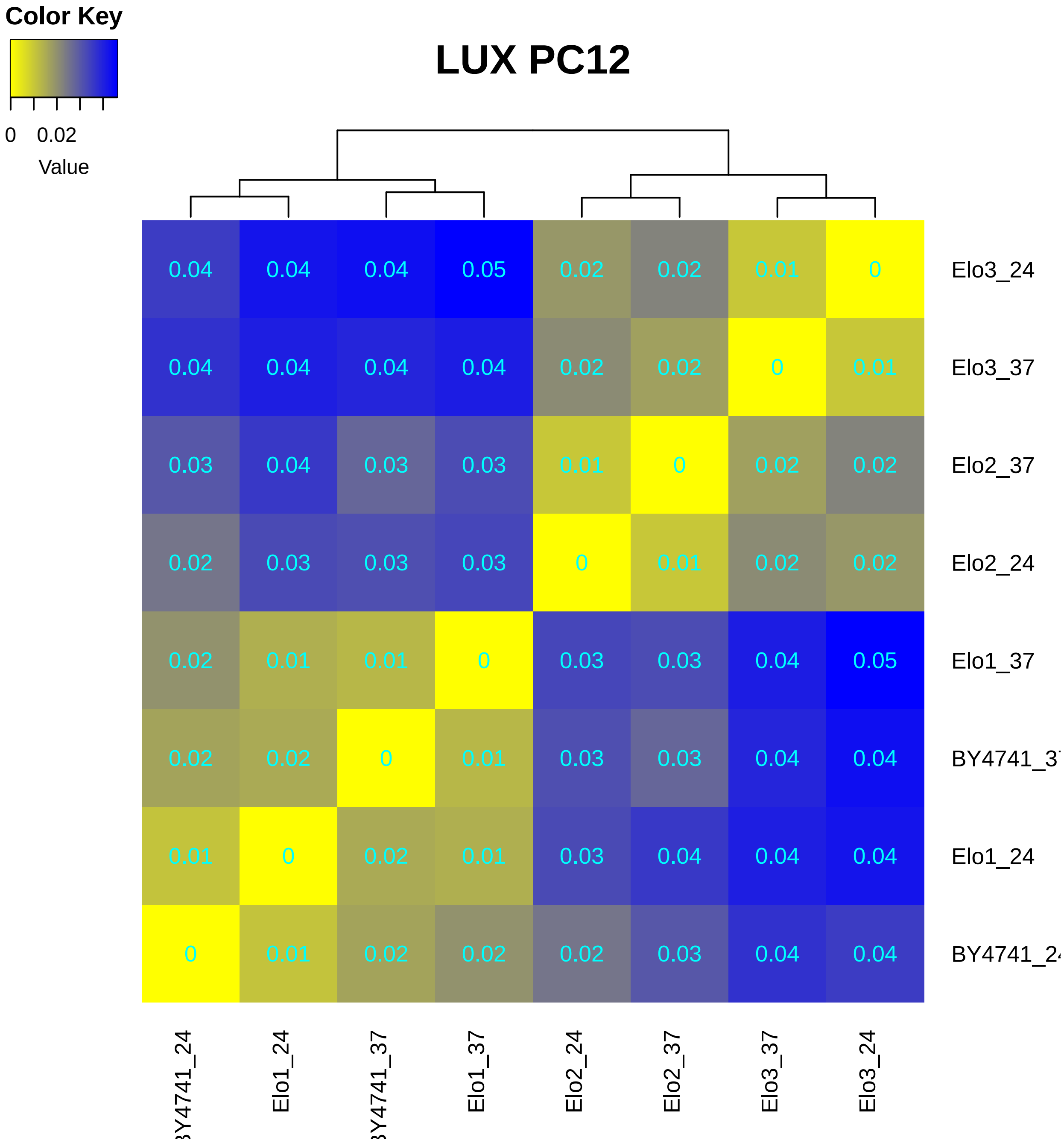
Color Key
0 0.02
Value
LUX PC12
0.04 0.04 0.04 0.05 0.02 0.02 0.01 0 Elo3_24
0.04 0.04 0.04 0.04 0.02 0.02 0 0.01 Elo3_37
0.03 0.04 0.03 0.03 0.01 0 0.02 0.02 Elo2_37
0.02 0.03 0.03 0.03 0 0.01 0.02 0.02 Elo2_24
0.02 0.01 0.01 0 0.03 0.03 0.04 0.05 Elo1_37
0.02 0.02 0 0.01 0.03 0.03 0.04 0.04 BY4741_3
0.01 0 0.02 0.01 0.03 0.04 0.04 0.04 Elo1_24
0 0.01 0.02 0.02 0.02 0.03 0.04 0.04 BY4741_2
3Y4741_24
Elo1_24
3Y4741_37
Elo1_37
Elo2_24
Elo2_37
Elo3_37
Elo3_24

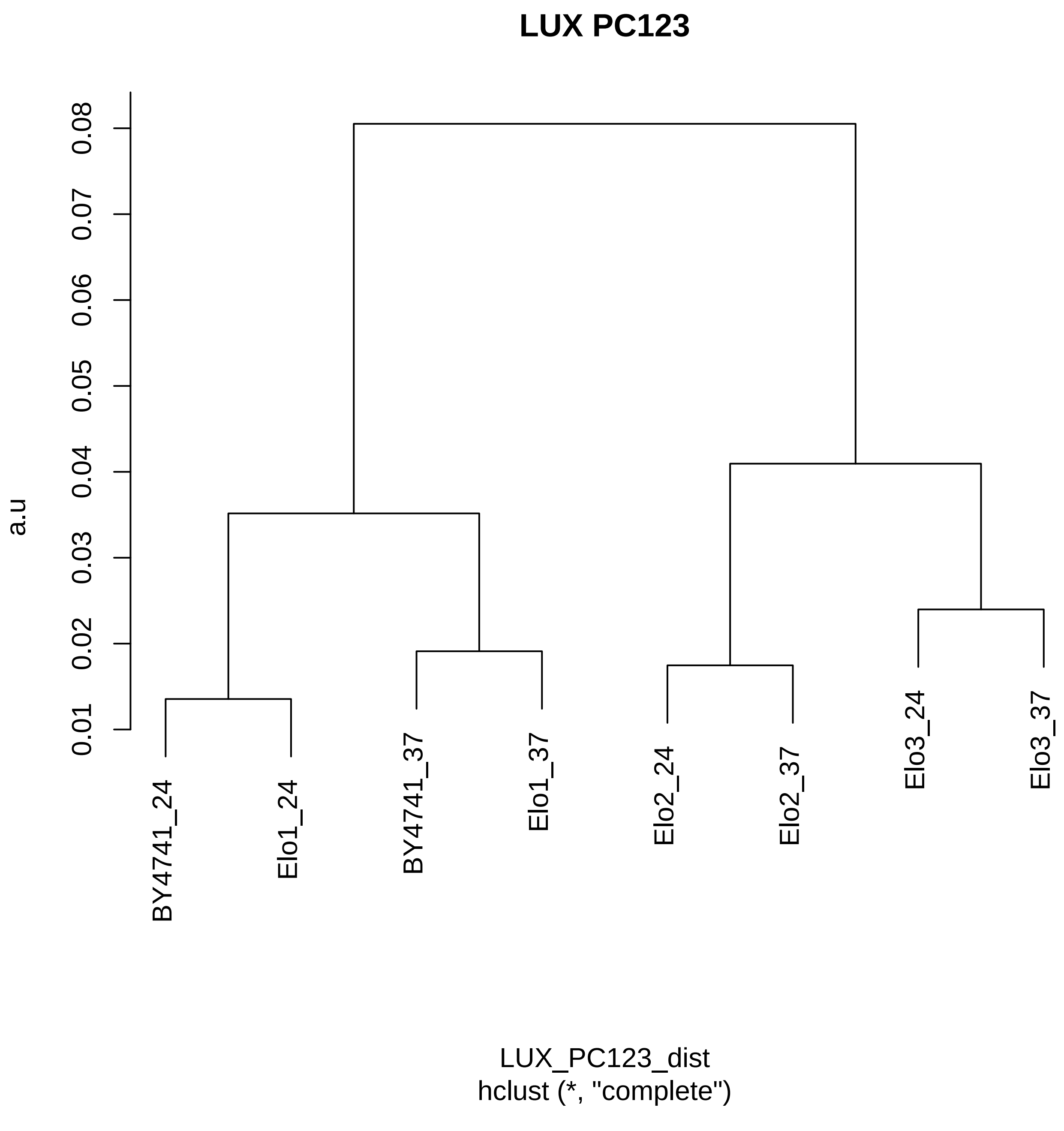
LUX PC123
a.u
0.08
0.07
0.06
0.05
0.04
0.03
0.02
0.01
BY4741_24
Elo1_24
BY4741_37
Elo1_37
Elo2_24
Elo2_37
Elo3_24
Elo3_37
LUX_PC123_dist
hclust (*, "complete")

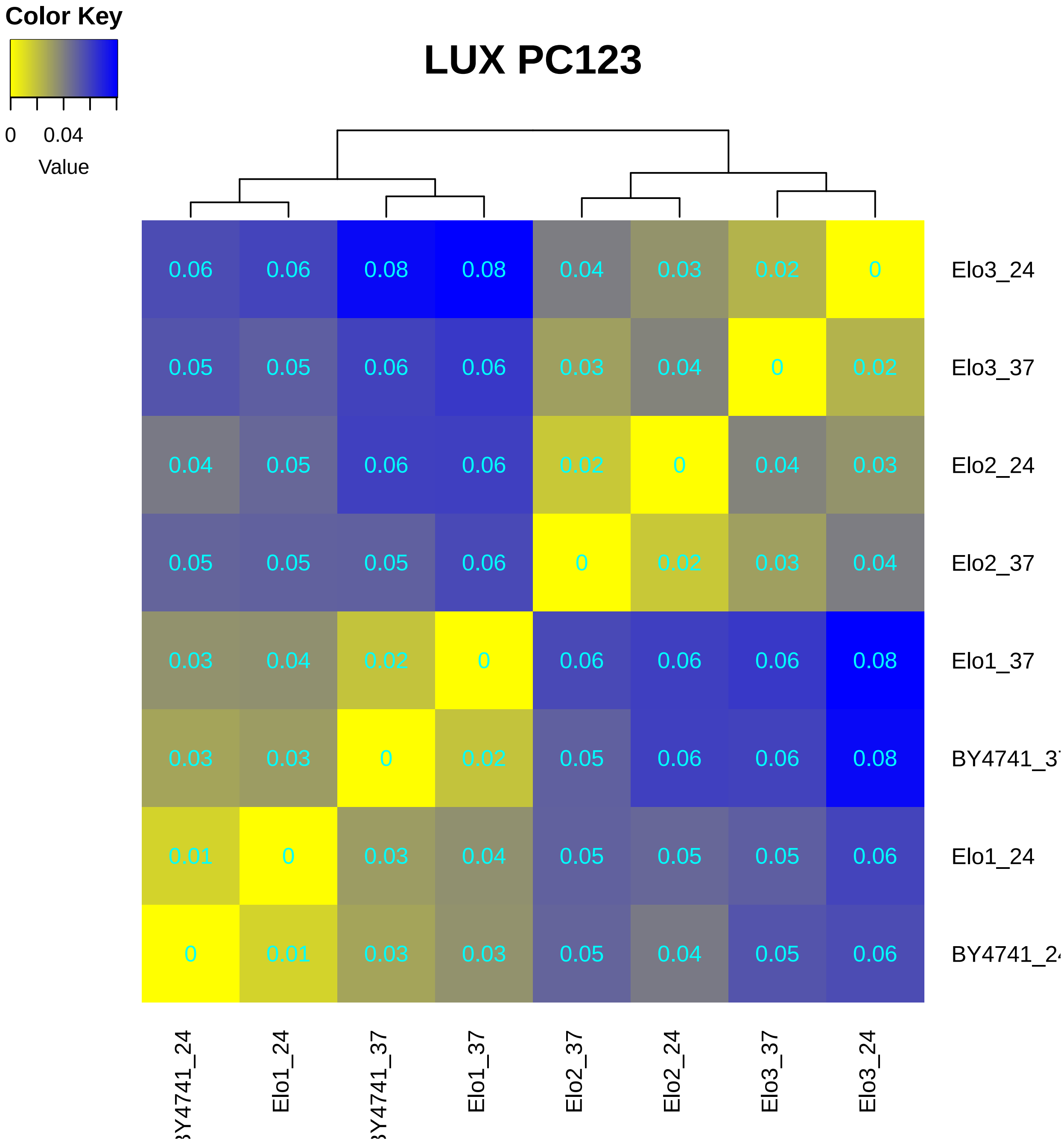
Color Key
0
0.04
Value
LUX PC123
Elo3_24
Elo3_37
Elo2_24
Elo2_37
Elo1_37
BY4741_37
Elo1_24
BY4741_24
BY4741_24
Elo1_24
BY4741_37
Elo1_37
Elo2_37
Elo2_24
Elo3_37
Elo3_24

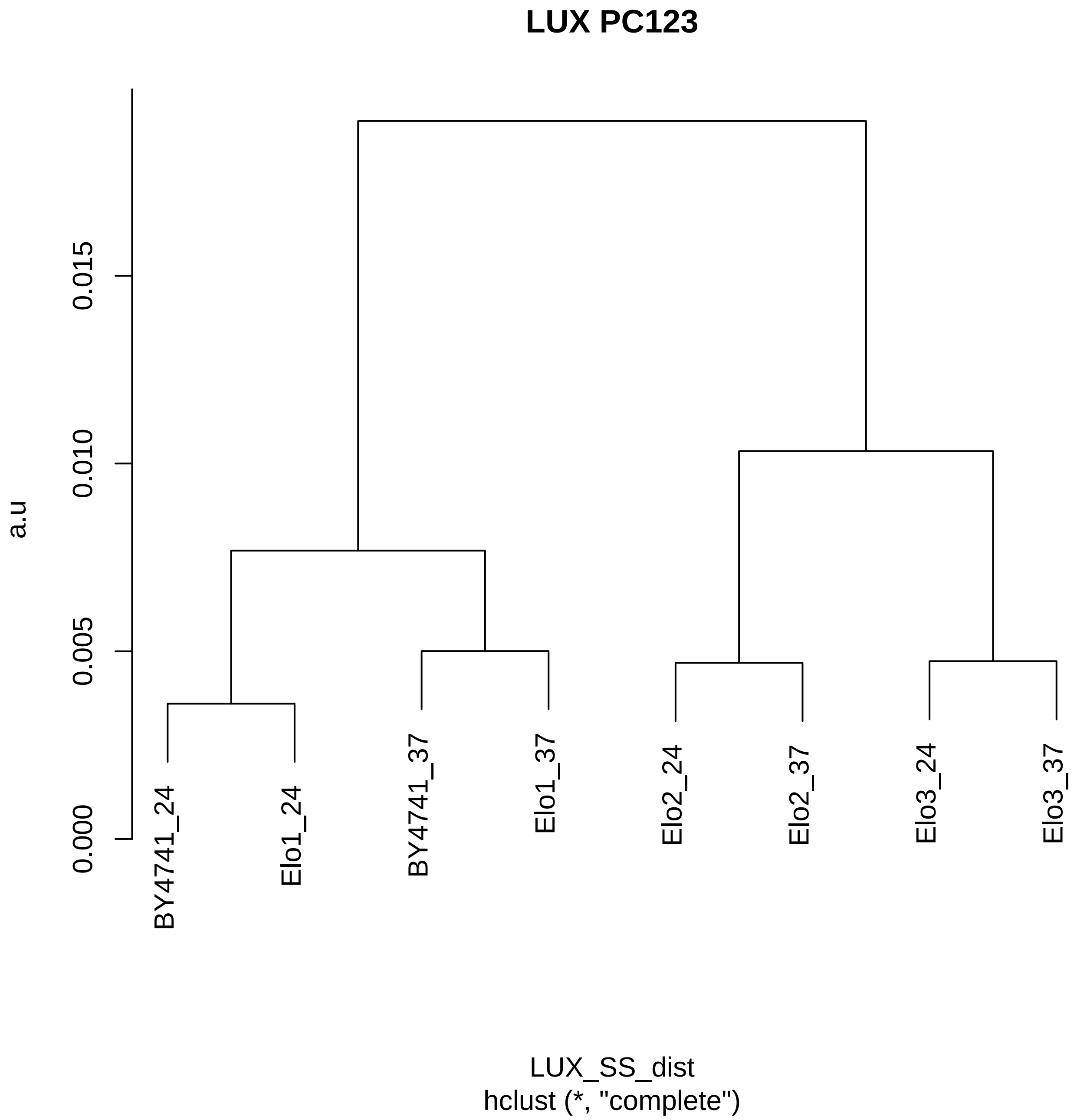
LUX PC123
a.u
0.000
0.005
0.010
0.015
BY4741_24
Elo1_24
BY4741_37
Elo1_37
Elo2_24
Elo2_37
Elo3_24
Elo3_37
LUX_SS_dist
hclust (*, "complete")

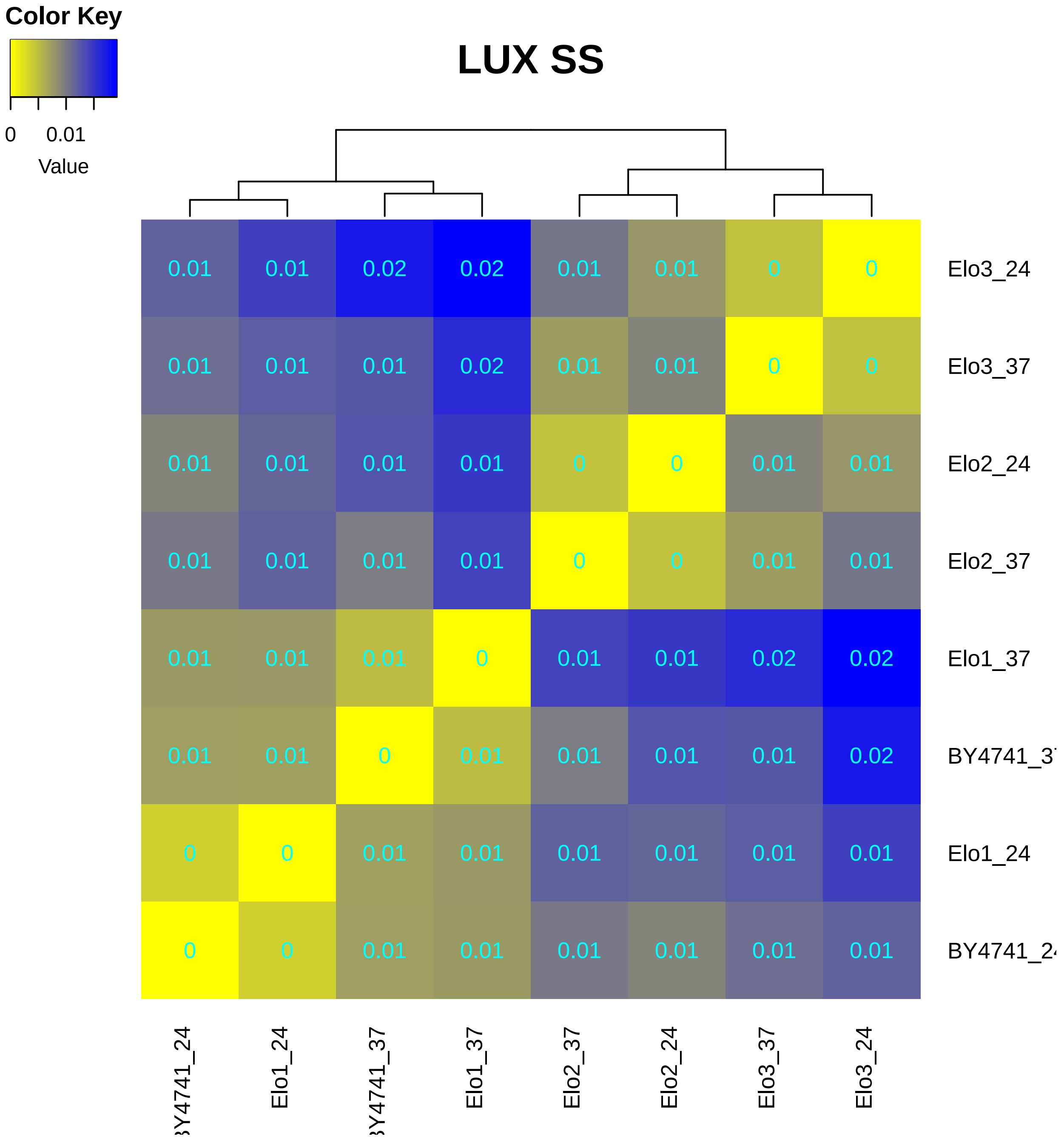
Color Key
0
0.01
Value
LUX SS
Elo3_24
Elo3_37
Elo2_24
Elo2_37
Elo1_37
Elo1_24
Elo1_24
Elo1_37
Elo2_37
Elo2_24
Elo3_37
Elo3_24

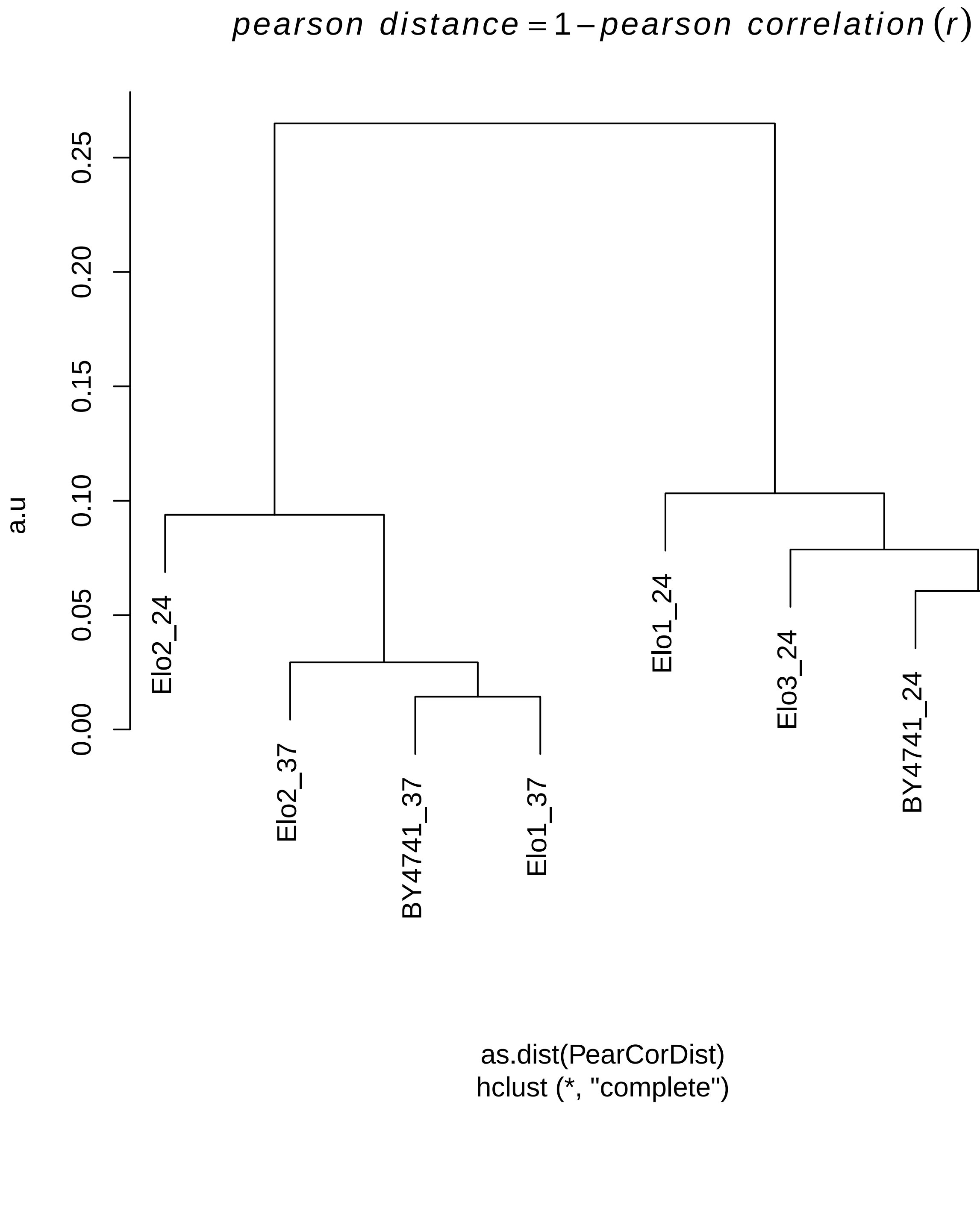

$pearson\ distance = 1 - pearson\ correlation\,(r)$
a.u
0.00
0.05
0.10
0.15
0.20
0.25
Elo2_24
Elo2_37
BY4741_37
Elo1_37
Elo1_24
Elo3_24
BY4741_24
Elo3_37
as.dist(PearCorDist)
hclust (*, "complete")

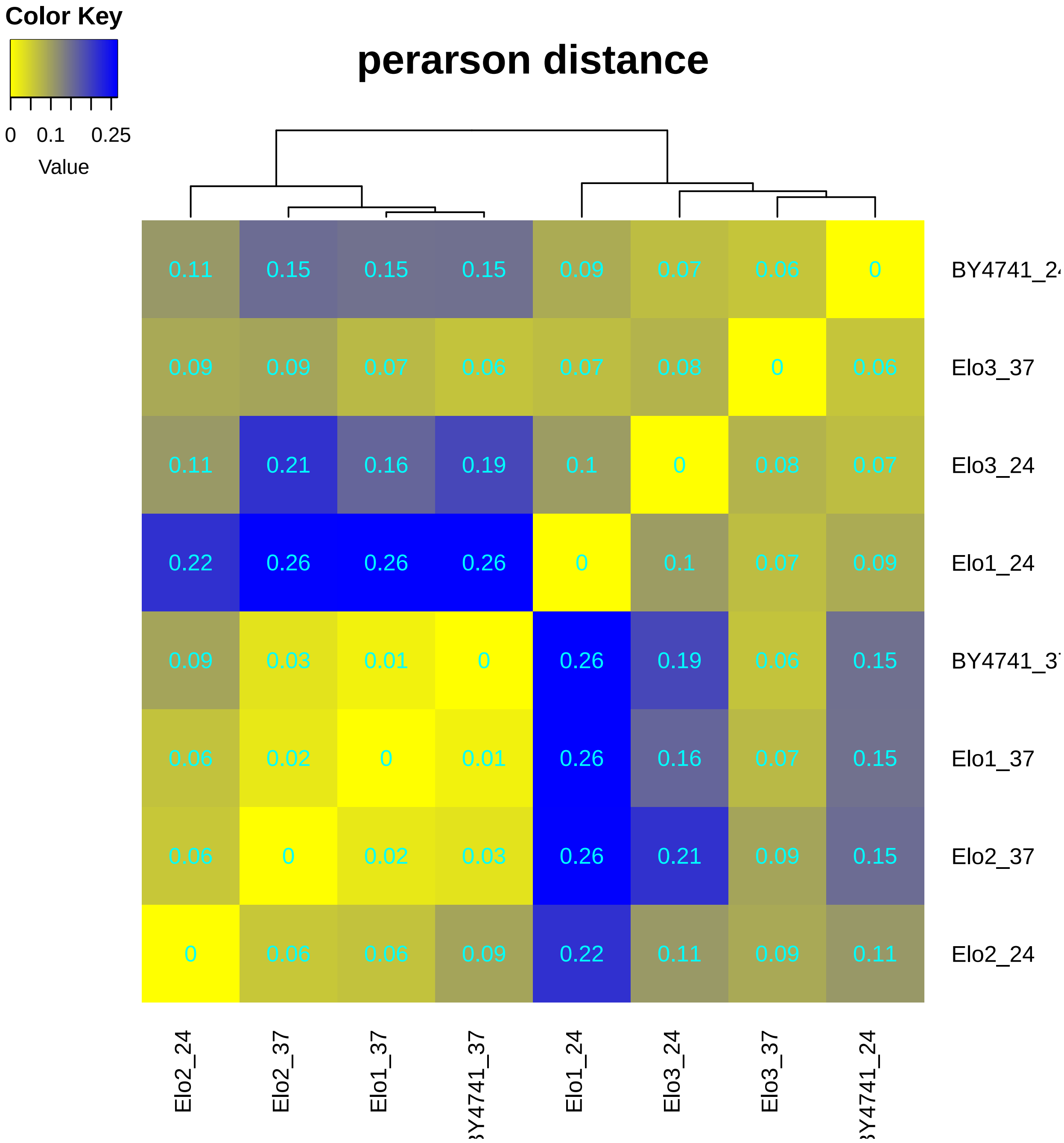
Color Key
0 0.1 0.25
Value
perarson distance
0.11 0.15 0.15 0.15 0.09 0.07 0.06 0
0.09 0.09 0.07 0.06 0.07 0.08 0 0.06
0.11 0.21 0.16 0.19 0.1 0 0.08 0.07
0.22 0.26 0.26 0.26 0 0.1 0.07 0.09
0.09 0.03 0.01 0 0.26 0.19 0.06 0.15
0.06 0.02 0 0.01 0.26 0.16 0.07 0.15
0.06 0 0.02 0.03 0.26 0.21 0.09 0.15
0 0.06 0.06 0.09 0.22 0.11 0.09 0.11
Elo3_37
Elo3_24
Elo1_24
Elo1_37
Elo2_37
Elo2_24
Elo2_24
Elo2_37
Elo1_37
Elo1_24
Elo3_24
Elo3_37

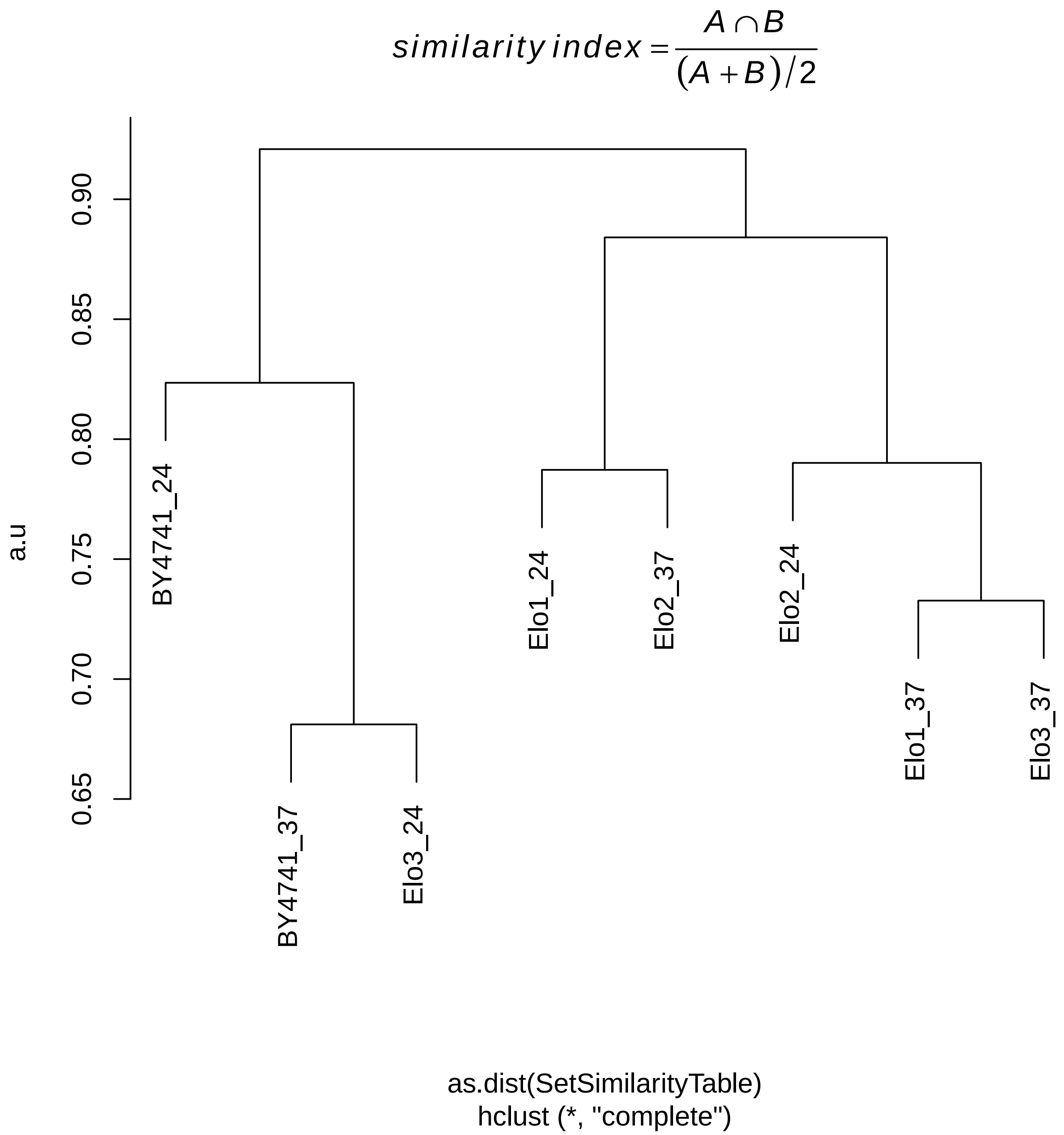
$$similarity\ index = \frac{A \cap B}{(A+B)/2}$$
a.u
0.65
0.70
0.75
0.80
0.85
0.90
BY4741_24
BY4741_37
Elo3_24
Elo1_24
Elo2_37
Elo2_24
Elo1_37
Elo3_37
as.dist(SetSimilarityTable)
hclust (*, "complete")

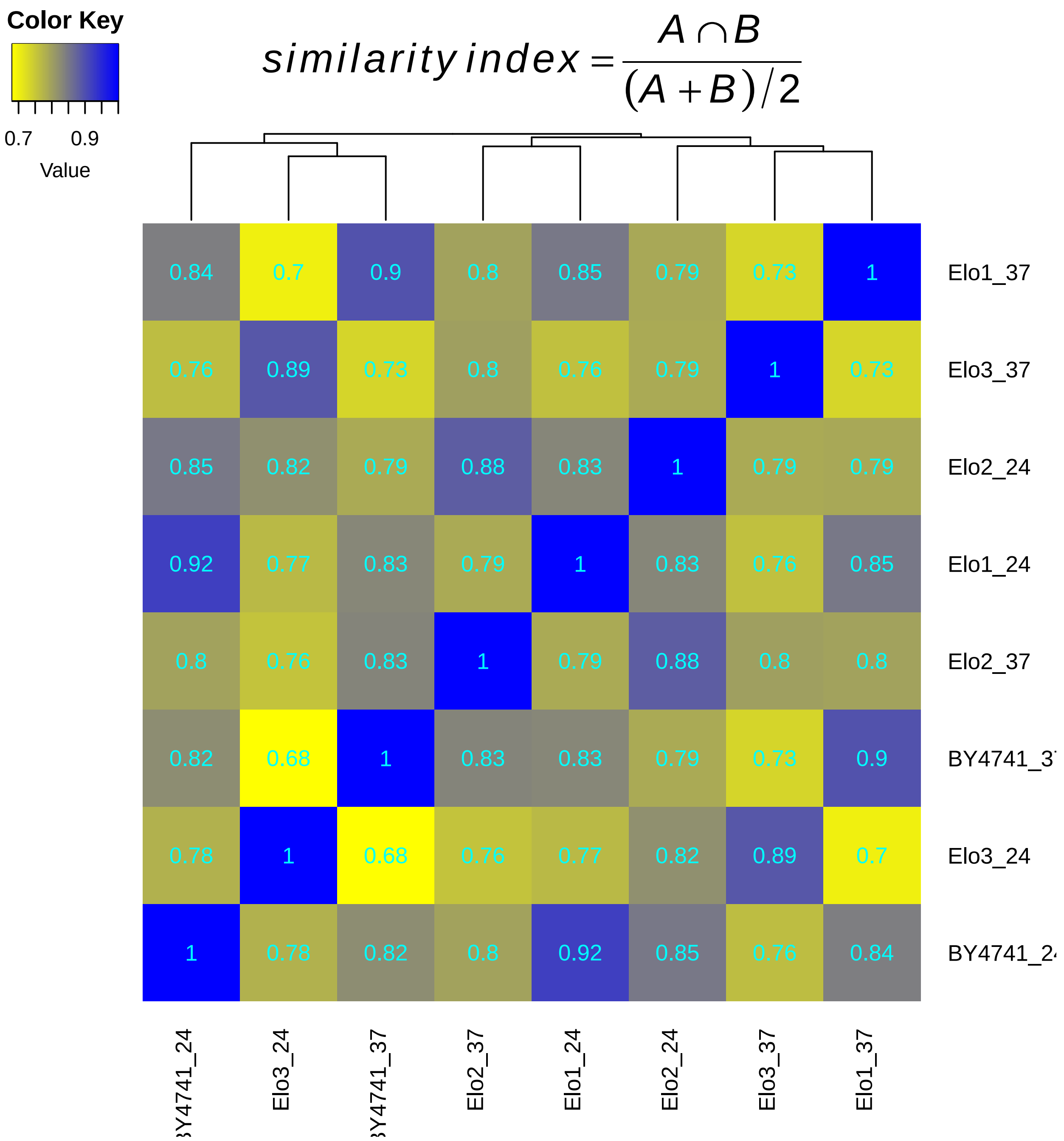
Color Key
0.7
0.9
Value
similarity index = A ∩ B / (A + B)/2
Elo1_37
0.84 0.7 0.9 0.8 0.85 0.79 0.73 1
Elo3_37
0.76 0.89 0.73 0.8 0.76 0.79 1 0.73
Elo2_24
0.85 0.82 0.79 0.88 0.83 1 0.79 0.79
Elo1_24
0.92 0.77 0.83 0.79 1 0.83 0.76 0.85
Elo2_37
0.8 0.76 0.83 1 0.79 0.88 0.8 0.8
BY4741_3
0.82 0.68 1 0.83 0.83 0.79 0.73 0.9
Elo3_24
0.78 1 0.68 0.76 0.77 0.82 0.89 0.7
BY4741_2
1 0.78 0.82 0.8 0.92 0.85 0.76 0.84
Y4741_24
Elo3_24
Y4741_37
Elo2_37
Elo1_24
Elo2_24
Elo3_37
Elo1_37

$$distance\ index = 1 - \frac{A \cap B}{(A+B)/2}$$

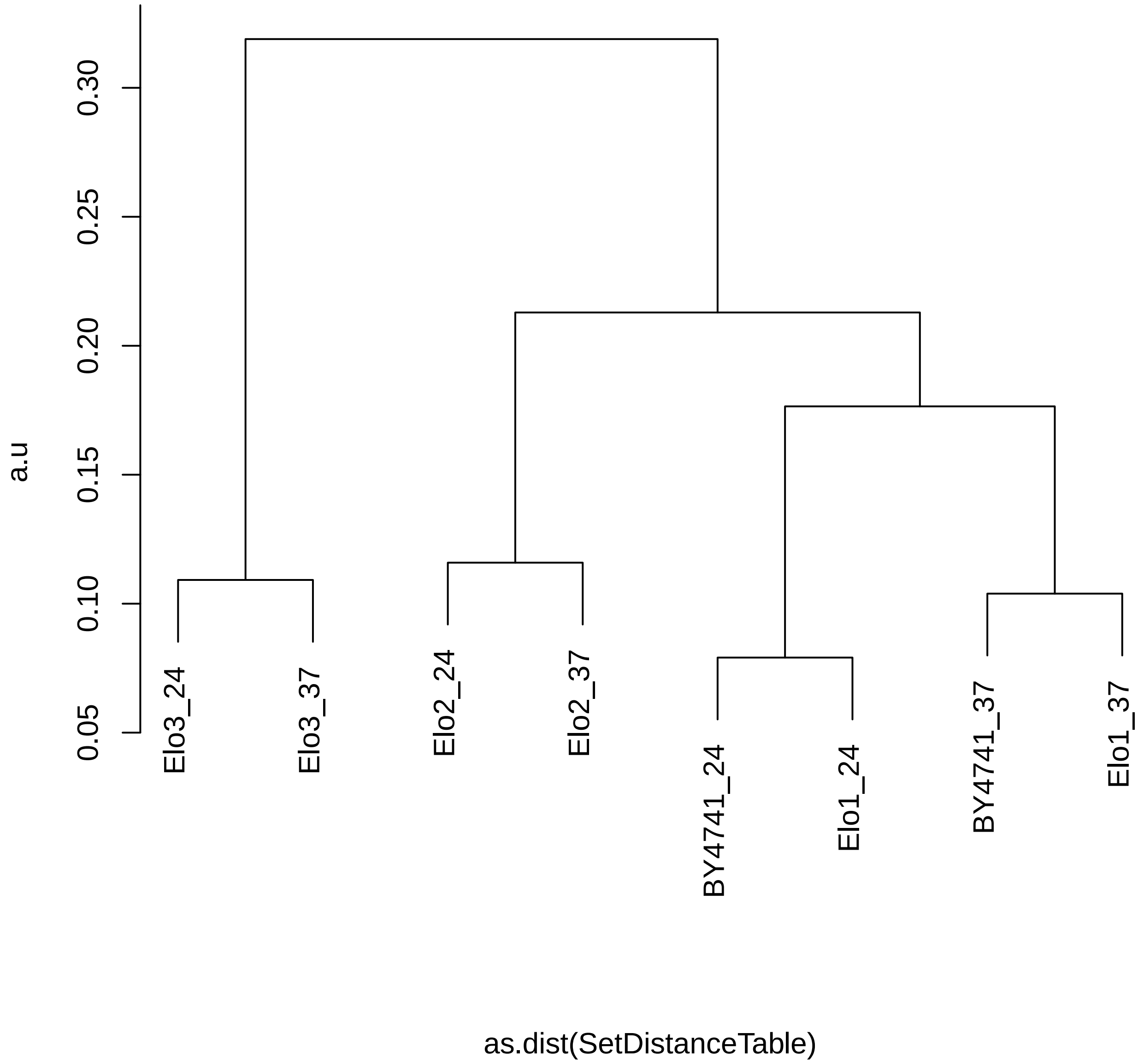

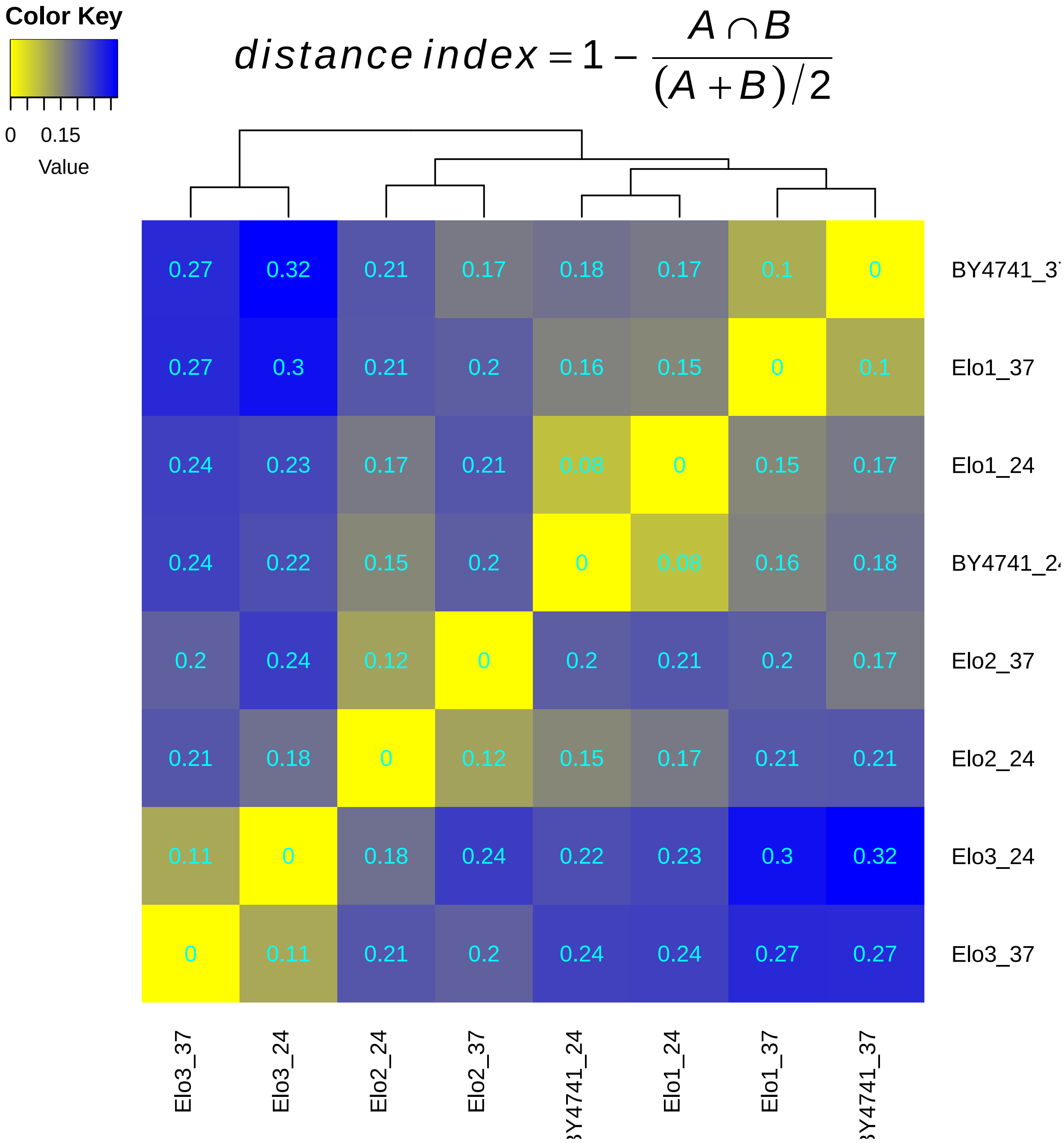

Color Key
0
0.15
Value
$$distance\ index = 1 - \frac{A \cap B}{(A+B)/2}$$
BY4741_3
Elo1_37
Elo1_24
BY4741_2
Elo2_37
Elo2_24
Elo3_24
Elo3_37
Elo3_37
Elo3_24
Elo2_24
Elo2_37
Y4741_24
Elo1_24
Elo1_37
Y4741_37
0.27 0.32 0.21 0.17 0.18 0.17 0.1 0
0.27 0.3 0.21 0.2 0.16 0.15 0 0.1
0.24 0.23 0.17 0.21 0.08 0 0.15 0.17
0.24 0.22 0.15 0.2 0 0.08 0.16 0.18
0.2 0.24 0.12 0 0.2 0.21 0.2 0.17
0.21 0.18 0 0.12 0.15 0.17 0.21 0.21
0.11 0 0.18 0.24 0.22 0.23 0.3 0.32
0 0.11 0.21 0.2 0.24 0.24 0.27 0.27
